# Potential blood biomarkers that can be used as prognosticators of spontaneous intracerebral hemorrhage: A systematic review and meta-analysis

**DOI:** 10.1371/journal.pone.0315333

**Published:** 2025-02-19

**Authors:** Aloysius Bagus Sasongko, Petra Octavian Perdana Wahjoepramono, Danny Halim, Jenifer Kiem Aviani, Achmad Adam, Yeo Tseng Tsai, Eka Julianta Wahjoepramono, Julius July, Tri Hanggono Achmad

**Affiliations:** 1 Department of Neurosurgery, Faculty of Medicine, Pelita Harapan University / Siloam Hospitals, Tangerang, Banten, Indonesia; 2 Post Graduate Program, Faculty of Medicine, Universitas Padjadjaran, Bandung, West Java, Indonesia; 3 Department of Neurosurgery, Faculty of Medicine, Universitas Padjadjaran / Dr. Hasan Sadikin General Hospital, Bandung, West Java, Indonesia; 4 Research Centre for Medical Genetics, Faculty of Medicine, Universitas Padjadjaran, Bandung, West Java, Indonesia; 5 Division of Neurosurgery, Department of Surgery, National University Hospital, Singapore, Singapore; 6 Department of Basic Medical Science, Faculty of Medicine, Universitas Padjadjaran, Bandung, West Java, Indonesia; Pescara General Hospital, ITALY

## Abstract

**Background:**

Predicting nontraumatic spontaneous intracerebral hemorrhage (SICH) patient prognosis has been commonly practiced, particularly when providing informed consent and considering surgical treatment. Biomarkers might provide more real-time evaluation of SICH patients’ condition than clinical prognostic scoring systems. This study aimed to evaluate the reliability of blood biomarkers in predicting prognosis in SICH patients by systematic review and meta-analysis.

**Methods:**

Studies that evaluated the association of blood biomarker(s) with mortality and/or functional outcome in SICH patients up to October 11, 2024, were identified through PubMed, Google Scholars, Scopus databases, and reference lists. Studies that satisfied the inclusion criteria were included in the meta-analyses. Good functional outcome was defined by patient’s Glasgow Outcome Scale (GOS) ≥ 4 or modified Rankin scale mRS ≤ 2. Blood biomarkers were classified into the following categories: angiogenic factors, growth factors, inflammatory biomarkers, coagulation parameters, blood counts, and others. Individual meta-analysis was performed for every evaluation endpoint:7 days, 30 days, 3 months, 6 months, and 1 year. Meta-analyses were performed using Random Effect Mean-Difference with a 95% Confidence Interval for continuous data and visualized as forest plots in RevMan version 5.3 software. Cochrane Tool to Assess Risk of Bias in Cohort Studies was used to assess potential risk of bias of the included studies. GRADE Profiler was used to assess quality of evidence.

**Results:**

Seventy-seven studies fulfilled the inclusion criteria. Surviving SICH patients have significantly lower C-reactive protein (CRP), D-dimer, copeptin, S100β, white blood cell (WBC), monocyte, and glucose than non-surviving patients. SICH patients with good functional outcome have lower D-dimer, Interleukin 6 (IL-6), tumor necrosis factor α (TNF-α), WBC count, neutrophil count, monocyte count, copeptin and significantly higher lymphocyte counts and calcium levels. Out of all blood biomarkers that were evaluated, only S100β and copeptin had very high effect size and high certainty of evidence.

**Conclusion:**

It is interesting to notice that many blood biomarkers significantly associated with SICH patients’ outcomes are related to inflammatory responses. This suggests that modulation of inflammation might be essential to improve SICH patients’ prognosis. We confidently concluded that S100β and copeptin are the most reliable blood biomarkers that can be used as prognosticators in SICH patients. On other biomarkers, in addition to heterogeneities and inconsistencies, several factors might affect the conclusions of current meta-analysis; thus, future studies to increase the certainties of evidence and effect size on other biomarkers are crucial.

## Introduction

Stroke is the second leading cause of death and the primary cause of disability globally [1, 2]. Stroke can be classified into ischemic and hemorrhagic. Although ischemic stroke is the predominant type in the overall stroke incidence, the mortality and morbidity among hemorrhagic stroke patients are higher. Recent global data acknowledged 7.6 million new cases of ischemic stroke annually, with 3.3 million deaths; meanwhile, although hemorrhagic stroke’s approximate annual incidence is only 3.4 million, as many as 3 million die of hemorrhagic stroke annually. This comparison suggests that the severity of hemorrhagic stroke is worse than ischemic stroke [1]. Morbidities, such as motor deficits, decreased cognitive function, urinary incontinence, and dysphagia, are common sequelae in hemorrhagic stroke patients with hemorrhagic stroke [3]. A comparison of epidemiology data on the global burden of stroke in 1990, 2013, and 2022 described the increase in disability-adjusted life-years (DALYs) due to hemorrhagic stroke from 55,953,376 to 68,572,498 years [1, 3].

Hemorrhagic stroke can be subclassified into spontaneous subarachnoid hemorrhage (SAH) and spontaneous intracerebral hemorrhage (SICH) [4]. Despite extensive studies have extended our understanding of SICH pathophysiology, its mortality and morbidity rates persisted, if not worsened [5, 6]. This suggests that the management of SICH patients remains unideal. Managing hemorrhagic stroke patients includes acute treatment, long-term treatment, and rehabilitation [7–9]. In an acute setting, caring physicians must select between surgical and non-surgical management for hemorrhagic stroke patients. In doing so, patient-specific risk stratification is one of the most important steps to selecting the optimum management of hemorrhagic stroke patients. Prognostic scoring systems, such as ICH score and National Institutes of Health Stroke Scale (NIHSS), are designed to assist physicians in this process [10, 11]; although previous studies suggested the reliabilities of these prognostic scoring systems, improvements are still required. Prognostic indicators that can provide real-time values depicting ICH patients’ condition may improve the reliability of the risk stratification system. Multiple studies on the basic science and clinical aspects of hemorrhagic stroke have unraveled various factors that play essential roles in the pathophysiology of SICH [12–14]. The significance of these factors in SICH pathophysiology indicates their potential as biomarkers [15, 16]; furthermore, it also indicates the molecular pathways that might be targeted to improve SICH patients’ outcomes. This meta-analysis evaluates the reliabilities of known admission blood biomarkers as mortality and functional prognostic indicators in hemorrhagic stroke patients.

## Methods

### Literature search and identification

This meta-analysis used the Preferred Reporting Items for Systematic Reviews and Meta-Analyses (PRISMA) statement [17]. PubMed, Scopus, Embase, Ovid, and Google Scholars databases were used to identify publications up to October 11, 2024. The following search terms were applied: spontaneous intracerebral hemorrhage OR hemorrhagic stroke AND biomarkers AND (mortality OR morbidity OR functional outcome OR Glasgow coma scale OR modified Rankin Scale OR disability). Additional studies were acknowledged through screening of the references.

### Inclusion and exclusion criteria

Studies were included if they reported: 1) spontaneous intracerebral hemorrhage (SICH) cases in patients aged ≥18 years, 2) mortality and/or functional outcome, and 3)admission biomarkers or laboratory data related to the outcomes. Studies were excluded if they were: 1) not based on original data, such as reviews, comments, and editorial letters; 2) not including a comparison group; 3) not written in English, 4) unpublished, 5) reporting cases of hemorrhage that were caused by trauma or predisposed by the entity(-ies) that require surgery by itself, such as aneurysm, arteriovenous malformation, and tumor, 6) reporting cases of ischemic stroke, 7) including patients with severe systemic disease, such as uremia, liver cirrhosis, malignancy, chronic heart, kidney, or lung disease, 8) including patients with prior infections within two weeks before admission, 9) including patients who were admitted >72h after onset, 10) not reporting the assessment time (such as in-hospital mortality), 11) not reporting mean or median of the biomarkers, 12) not including sufficient information.

### Data collection and analysis

Four authors (ABS, POPW, DH, JKA) independently reviewed the title and abstract of every article. The full-text article was thoroughly assessed if the abstract met the inclusion criteria. The following information was retrieved: author, country, publication year, number of patients, inclusion and exclusion criteria, stroke etiologies, reported outcomes, methodology for outcome measurement (i.e. mRS score), and biomarkers or laboratory data. Meta-analysis was performed to assess the outcomes at every evaluation endpoint. Cochrane Tool to Assess Risk of Bias in Cohort Studies was used to assess potential risk of bias of the included studies.

### Outcome variables

The assessed outcome variables of this meta-analysis are mortality and good functional outcome. Mortality outcomes depicted the number of ICH patients who survived and died of the ICH incidence. Good functional outcome is concluded when ICH patient’s Glasgow Outcome Scale (GOS) ≥4 or modified Rankin scale (mRS) ≤2; consequently, poor outcome is concluded when ICH patient’s GOS ≤3 or mRS ≥3. Both variables are evaluated at the following endpoints: 7-day, 30-day, 3-month, 6-month, and 1-year.

### Statistical analysis

Meta-analyses were performed using Random Effect Mean-Difference with a 95% Confidence Interval for continuous data and visualized as forest plots in RevMan version 5.3 software (Cochrane Collaboration). P-values that are <0.05 indicate significant differences. The inconsistency index (I^2^) test ranges from 0 to 100% and calculated to evaluate heterogeneity across studies. Values above 50% or p-value <0.05 indicate a significant heterogeneity. The certainty of evidence was assessed using GRADEPro web-service (gradepro.org). The assessment consists of 8 factors: study design (randomized controlled trial or observational study), inconsistency, indirectness, imprecision, publication bias, large effect size, plausible confounding factor, and dose-response gradient. Inconsistency was measured by I^2^, its p-value, and the direction of the studies [18]. Imprecision for continuous variables is graded to extremely serious, very serious, serious, not serious if the study sample size is less than 30%, 40%, 50%, and more than 50% of Optimum Information Size (minimum number of patients recruited to give reliable value with α = 0.05, β = 0.20), respectively [19]. Effect size was calculated using Hedges’ g effect size with the following criterion: Small effect size (0.0 –<0.5), medium effect size (0.5 - <0.8), large effect size (0.8 –<1.4), and very large effect size (>1.4).

## Results

**Fig 1:**
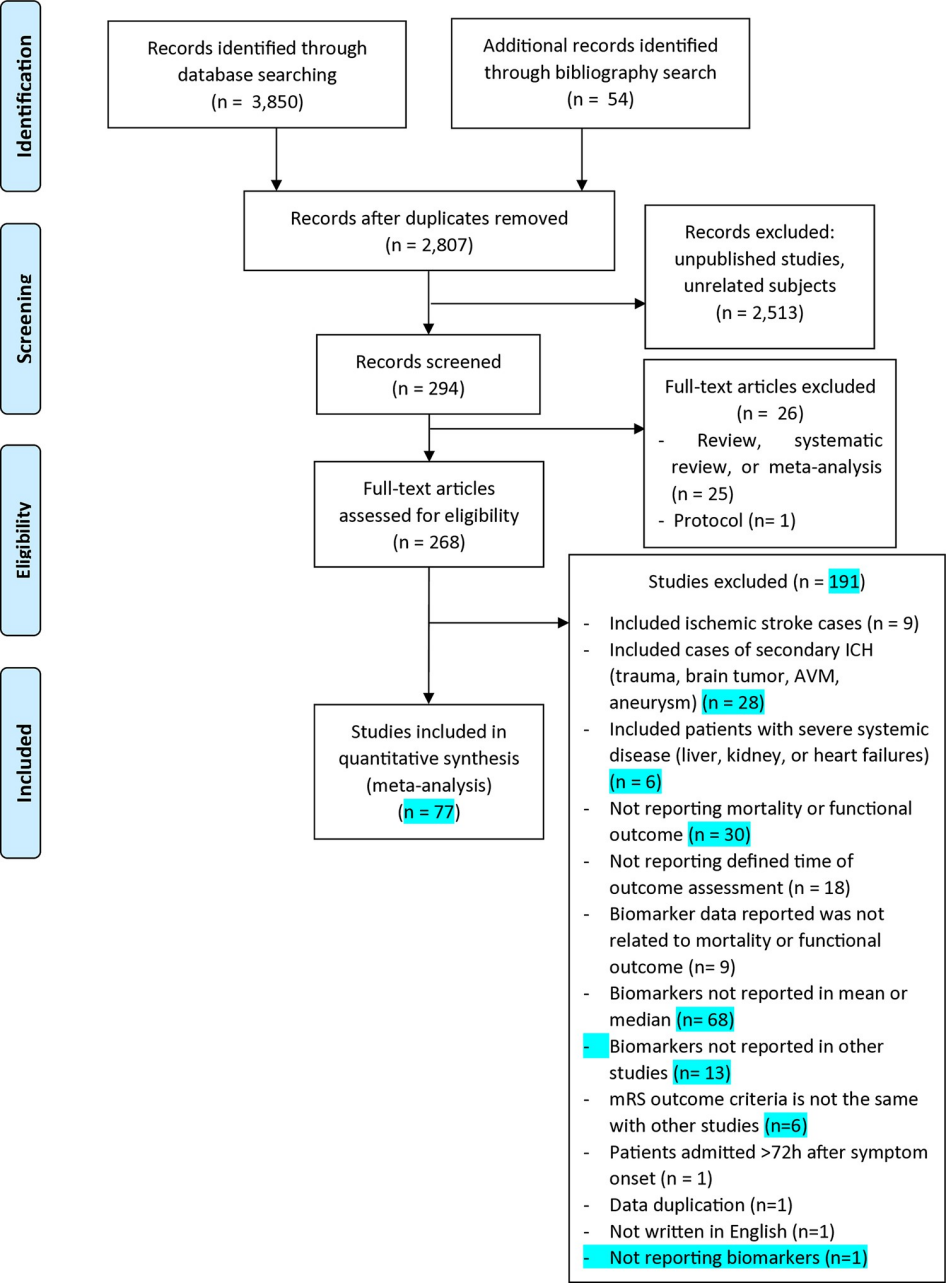
Flow diagram of the study selection.

The literature searches identified 3,850 studies. Additionally, reference screening acknowledged 54 studies **(Fig 1)**. After duplicates, unpublished studies, unrelated subjects, reviews, protocols, and studies on animal models were excluded, a total of 268 full-text articles were assessed. Only 77 articles fulfilled the inclusion criteria, thus included in the meta-analysis [18–93]. The characteristics of full-text articles screened for eligibility are presented in **S1 Table.** The quality of the included studies was written in **S1 Fig** and all studies showed low bias in terms of selection bias, exposure bias, methodological, outcome, follow-up, and intervention biases. All included studies are observational studies, including prospective or retrospective cohort, that did not match patients at the beginning of the study. **S2 Table** summarizes certainty of evidence for mortality and functional outcome. **S3 Table** summarizes the Effect Size and Optimum Information Size for each variable.

### Mortality outcomes

**Fig 2:**
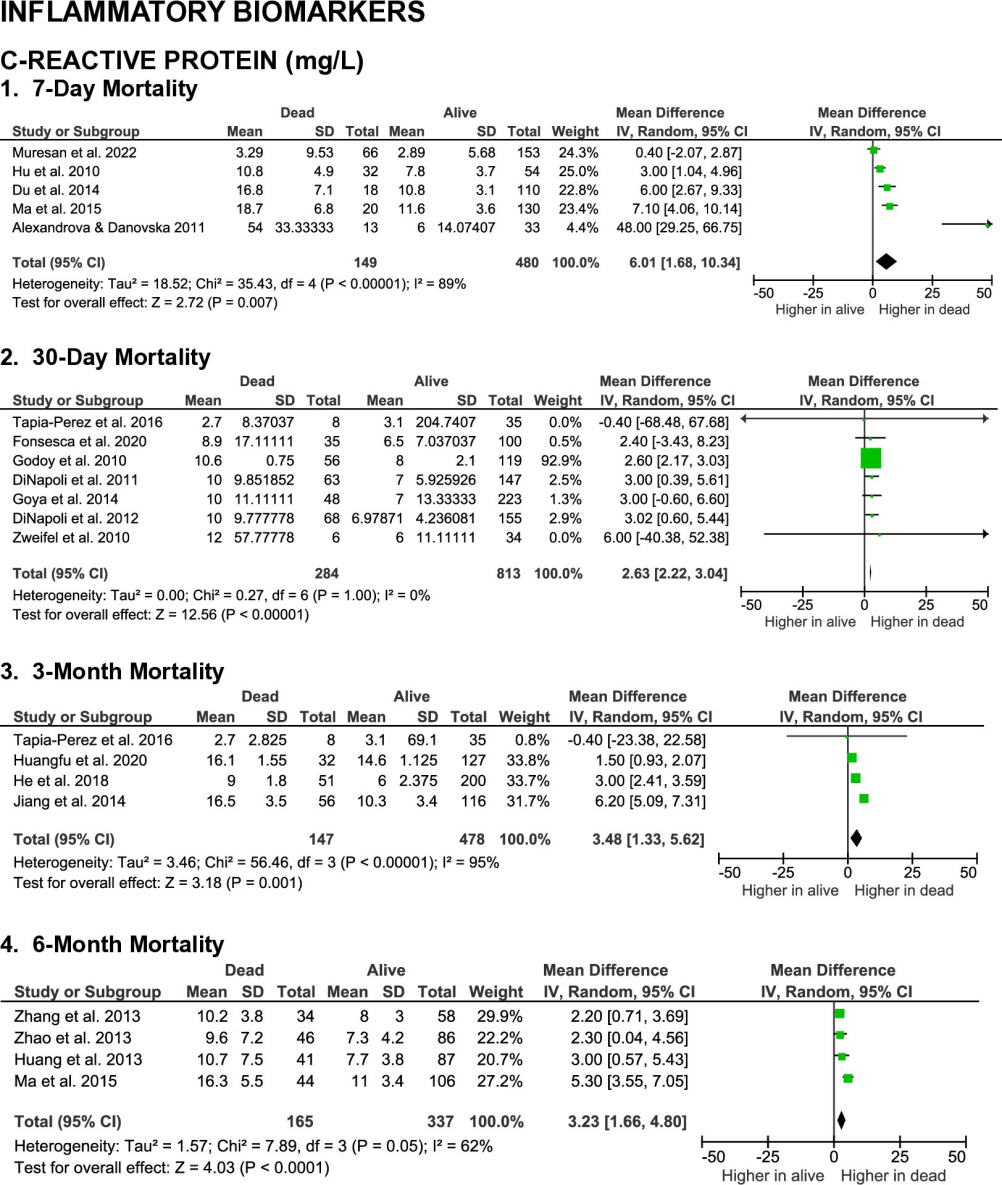
Forest plots on the association of inflammatory serum biomarkers with SICH patients’ mortality.

**Fig 3:**
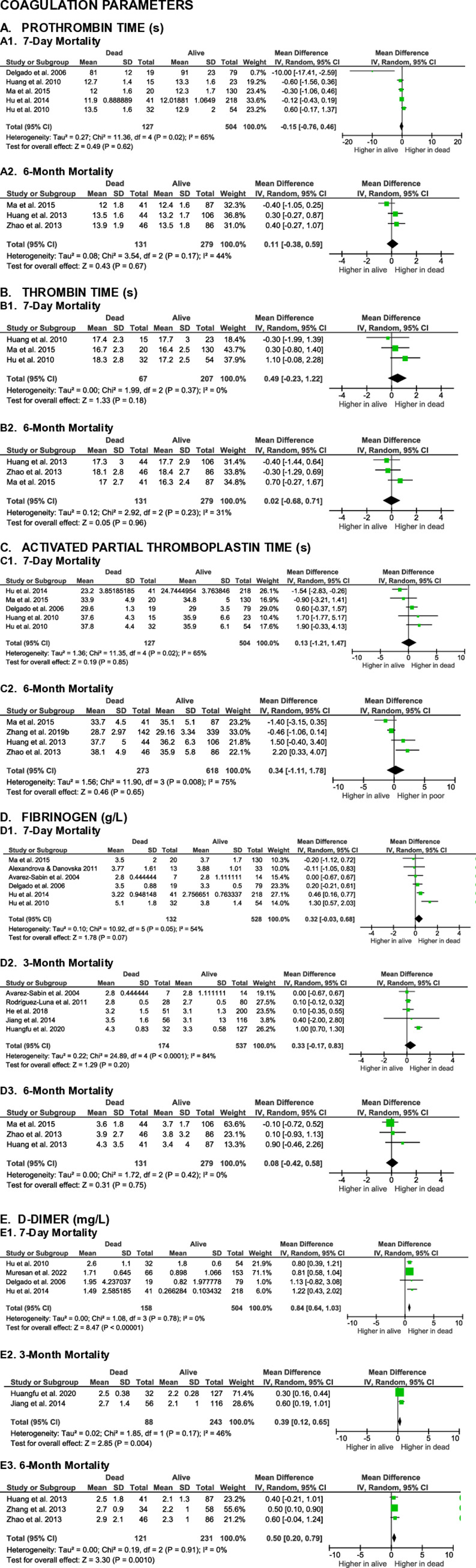
Forest plots of the association of coagulation parameters with SICH patients’ mortality.

**Fig 4:**
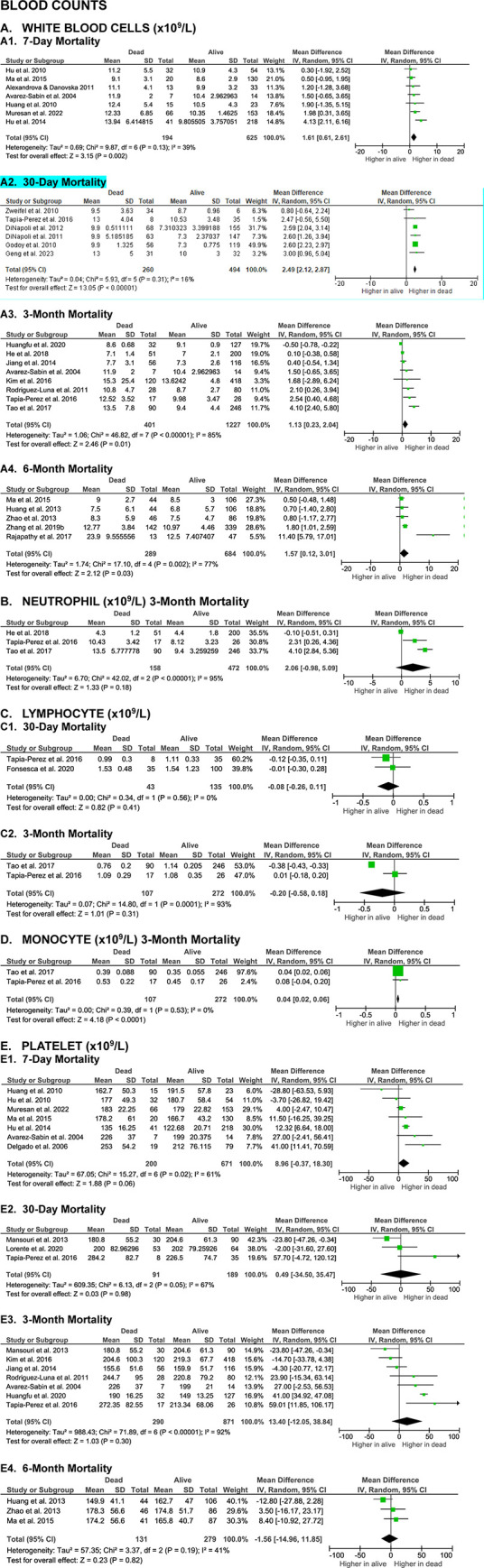
Meta-analyses on the association of blood counts with SICH patients’ mortality.

**Fig 5:**
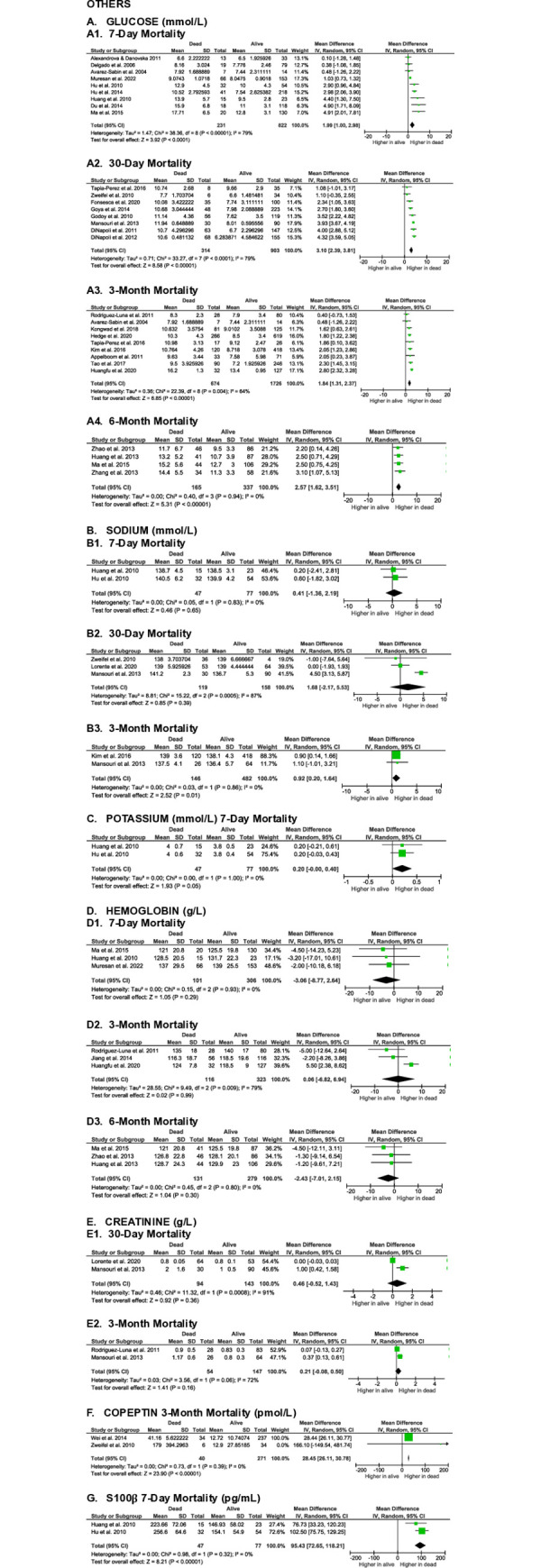
Meta-analyses on the association of other blood serum parameters with SICH patients’ mortality.

**Figs 2** to **5** summarize the results of meta-analyses from studies that evaluated the correlation between blood biomarkers and mortality outcomes in patients with spontaneous intracerebral hemorrhage (SICH) at various time points, including 7-day, 30-day, 3-month, and 6-month post-event. The analyzed blood biomarkers were categorized into subgroups, including inflammatory markers, coagulation parameters, blood cell counts, and others, based on their biological roles and characteristics.

#### Inflammatory biomarkers

A total of 14 studies evaluated the association between C-reactive protein (CRP) levels and mortality in patients with spontaneous intracerebral hemorrhage (SICH) at the 7-day, 30-day, 3-month, and 6-month evaluation endpoints. Significantly higher CRP levels were observed in non-surviving patients at the 7-day (MD 6.01 [1.68, 10.34], z = 2.72, p = 0.007; I^2^ = 89%, p < 0.00001), 30-day (MD 2.63 [2.22, 3.04], z = 12.56, p < 0.00001; I^2^ = 0%, p = 1.00), 3-month (MD 3.48 [1.33, 5.62], z = 3.18, p = 0.001; I^2^ = 95%, p < 0.00001), and 6-month (MD 3.23 [1.66, 4.80], z = 4.03, p < 0.0001; I^2^ = 62%, p = 0.05) time points (**Fig 2**). Despite the medium to large effect sizes, the certainty of evidence were low for the 7-day, 3-month, and 6-month outcomes, and moderate for the 30-day outcome.

#### Coagulation parameters

Prothrombin time (PT), thrombin time (TT), activated partial thromboplastin time (aPTT), and plasma fibrinogen are commonly assessed blood coagulation parameters that have been studied for their association with mortality in patients with spontaneous intracerebral hemorrhage (SICH).

Seven studies evaluated the association between PT and mortality at the 7-day and 6-month endpoints in SICH patients. Meta-analysis results indicated that the differences in PT levels between non-surviving and surviving SICH patients were not statistically significant at 7-day (MD -0.15 [-0.76, 0.46], z = 0.49, p = 0.62; I^2^ = 65%, p = 0.02) or at 6-month (MD 0.11 [-0.38, 0.59], z = 0.43, p = 0.67; I^2^ = 44%, p = 0.17) time points (**Fig 3A**).

Only five studies evaluated the association of TT with mortality in SICH patients. The differences in TT between non-surviving and surviving patients were not significant at both 7-day (MD 0.49 [-0.23, 1.22], z = 1.33, p = 0.18; I^2^ = 0%, p = 0.37) and 6-month (MD 0.02 [-0.68, 0.71], z = 0.05, p = 0.96; I^2^ = 31%, p = 0.23) follow-ups (**Fig 3B**).

Regarding aPTT, eight studies assessed its correlation with mortality in SICH patients. The differences in aPTT between non-surviving and surviving patients were not significant at either the 7-day (MD 0.13 [-1.21, 1.47], z = 0.19, p = 0.85; I^2^ = 65%, p = 0.02) or the 6-month evaluation points (MD 0.34 [-1.11, 1.78], z = 0.46, p = 0.65; I^2^ = 75%, p = 0.008) (**Fig 3C**).

Thirteen studies investigated the association between fibrinogen levels and mortality in SICH patients. Meta-analysis results showed no significant differences in fibrinogen levels between non-surviving and surviving patients at 7-day (MD 0.32 [-0.03, 0.68], z = 1.78, p = 0.07; I^2^ = 54%, p = 0.05), 3-month (MD 0.33 [-0.17, 0.83], z = 1.29, p = 0.20; I^2^ = 84%, p < 0.0001), or 6-month (MD 0.08 [-0.42, 0.58], z = 0.31, p = 0.75; I^2^ = 0%, p = 0.42) follow-up (**Fig 3D**).

Nine studies evaluated the association between D-dimer levels and mortality in SICH patients, regardless of hemorrhage location. Meta-analyses revealed that non-surviving SICH patients had significantly higher D-dimer levels compared to surviving patients at 7-day (MD 0.84 [0.64, 1.03], z = 8.47, p < 0.00001; I^2^ = 0%, p = 0.78), 3-month (MD 0.39 [0.12, 0.65], z = 2.85, p = 0.004; I^2^ = 46%, p = 0.17), and 6-month (MD 0.50 [0.20, 0.79], z = 3.30, p = 0.0010; I^2^ = 0%, p = 0.91) time points (**Fig 3E**).

The certainty of evidence for blood coagulation parameters as mortality biomarkers were very low or low with small effect sizes, except for D-dimer, which demonstrated a large effect size and moderate evidence for predicting 7-day mortality.

#### Blood counts

A meta-analysis of 23 studies revealed significantly higher baseline white blood cells (WBC) counts in non-surviving SICH patients at all evaluated time points, including 7-day (MD 1.61 [0.61, 2.61], z = 3.15, p = 0.002; I^2^ = 39%, p = 0.13), 30-day (MD 2.49 [2.12, 2.87], z = 13.05, p < 0.00001; I^2^ = 16%, p = 0.31), 3-month (MD 1.60 [0.45, 2.76], z = 2.71, p = 0.007; I^2^ = 78%, p = 0.0001), and 6-month (MD 1.57 [0.12, 3.01], z = 2.12, p = 0.03; I^2^ = 77%, p = 0.002) (**Fig 4A**).

For neutrophil counts, a meta-analysis of three studies found that differences between surviving and non-surviving SICH patients at the 3-month endpoint were not statistically significant (MD 2.06 [-0.98, 5.09], z = 1.33, p = 0.18; I^2^ = 95%, p < 0.00001) (**Fig 4B**).

Similarly, three studies on lymphocyte counts showed no significant differences between surviving and non-surviving SICH patients at either the 30-day (MD -0.08 [-0.26, 0.11], z = 0.82, p = 0.41; I^2^ = 0%, p = 0.56) or 3-month (MD -0.20 [-0.58, 0.18], z = 1.01, p = 0.31; I^2^ = 93%, p = 0.0001) time points (**Fig 4C**).

Only two studies evaluated monocyte counts in relation to mortality, and the meta-analysis showed that at 3-month evaluation time, non-surviving SICH patients had significantly higher baseline monocyte counts (MD 0.04 [0.02, 0.06], z = 4.18, p < 0.0001; I^2^ = 0%, p = 0.53) (**Fig 4D**).

Sixteen studies investigated the association between platelet counts and mortality in SICH patients. The meta-analysis found no significant differences in platelet counts between non-surviving and surviving patients at any evaluation points, including 7-day (MD 8.96 [-0.37, 18.30], z = 1.88, p = 0.06; I^2^ = 61%, p = 0.02), 30-day (MD 0.49 [-34.50, 35.47], z = 0.03, p = 0.98; I^2^ = 67%, p = 0.05), 3-month (MD 13.40 [-12.05, 38.84], z = 1.03, p = 0.30; I^2^ = 92%, p < 0.00001), and 6-month (MD -1.56 [-14.96, 11.85], z = 0.23, p = 0.82; I^2^ = 41%, p = 0.19) (**Fig 4E**).

Among the blood count parameters, only WBC counts demonstrated moderate certainty in predicting 30-day mortality, with a large effect size. Other blood count parameters showed very low to low certainty and small effect sizes for predicting short- to long-term mortality.

#### Others

Previous studies have also assessed other routine blood examination values that may be associated with mortality rates in SICH patients, including glucose, sodium, potassium, hemoglobin (Hb), and creatinine (Cr). A meta-analysis of 28 studies revealed significantly higher glucose levels in non-surviving SICH patients across all evaluation time points: 7-day (MD 1.99 [1.00, 2.98], z = 3.92, p < 0.0001; I^2^ = 79%, p < 0.00001), 30-day (MD 3.10 [2.39, 3.81], z = 8.58, p < 0.00001; I^2^ = 79%, p < 0.0001), 3-month (MD 1.84 [1.31, 2.37], z = 6.85, p < 0.00001; I^2^ = 64%, p = 0.004), and 6-month (MD 2.57 [1.62, 3.51], z = 5.31, p < 0.00001; I^2^ = 0%, p = 0.94) (**Fig 5A**). Although these findings were significant, the quality of evidence for glucose levels in predicting mid- to long-term mortality was very low to low, with medium to large effect sizes.

Six studies examined the association of sodium levels with mortality rates in SICH patients. Meta-analyses revealed no significant differences in sodium levels between non-survivors and survivors at the 7-day (MD 0.41 [-1.36, 2.19], z = 0.46, p = 0.65; I^2^ = 0%, p = 0.83), 30-day (MD 1.68 [-2.17, 5.53], z = 0.85, p = 0.39; I^2^ = 87%, p = 0.0005), and 3-month (MD 0.92 [0.20, 1.64], z = 2.52, p = 0.01; I^2^ = 0%, p = 0.86) endpoints (**Fig 5B**).

A meta-analysis of two studies on potassium levels found no significant differences between survivors and non-survivors at the 7-day endpoint (MD 0.20 [0.00, 0.40], z = 1.93, p = 0.05; I^2^ = 0%, p = 1.00) (**Fig 5C**).

Meta-analyses of eight studies assessing the relationship between hemoglobin levels and mortality in SICH patients revealed no statistically significant differences between survivors and non-survivors at the 7-day (MD -3.06 [-8.77, 2.64], z = 1.05, p = 0.29; I^2^ = 0%, p = 0.93), 3-month (MD 0.06 [-6.82, 6.94], z = 0.02, p = 0.99; I^2^ = 79%, p = 0.009), and 6-month (MD -2.43 [-7.01, 2.15], z = 1.04, p = 0.30; I^2^ = 0%, p = 0.80) endpoints (**Fig 5D**).

Similarly, meta-analyses of three studies on creatinine levels found no significant differences between survivors and non-survivors at the 30-day (MD 0.46 [-0.52, 1.43], z = 0.92, p = 0.36; I^2^ = 91%, p = 0.0008) and 3-month (MD 0.21 [-0.08, 0.50], z = 1.41, p = 0.16; I^2^ = 72%, p = 0.06) endpoints (**Fig 5E**). The certainty of sodium, potassium, hemoglobin, and creatinine levels in predicting short- to long-term mortality were very low, with small effect sizes.

Only two studies evaluated the association between copeptin levels and mortality in SICH patients at the 3-month endpoint. The meta-analysis revealed significantly higher copeptin levels in non-survivors compared to survivors (MD 28.45 [26.11, 30.78], z = 23.90, p < 0.00001; I^2^ = 0%, p = 0.39), with a very high effect size and high certainty of evidence (**Fig 5F**).

Similarly, the association between S100 calcium-binding protein beta (S100β) levels and mortality was evaluated in two studies. Meta-analysis showed significantly higher S100β levels in non-survivors compared to survivors at the 7-day evaluation endpoint (MD 95.43 [72.65, 118.21], z = 8.21, p < 0.00001; I^2^ = 0%, p = 0.32). This analysis also demonstrated a very high effect size with high certainty of evidence (**Fig 5G**).

### Functional outcome

**Fig 6:**
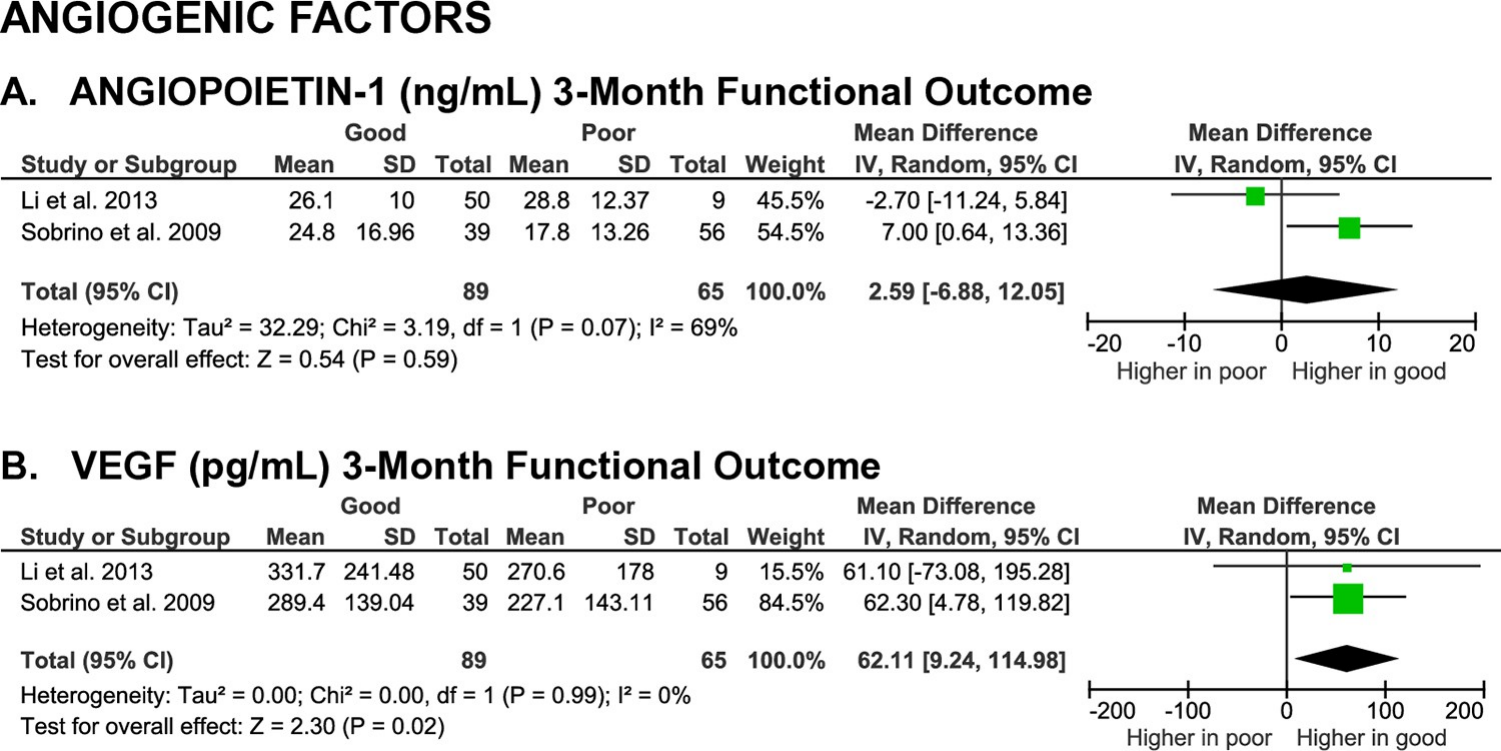
Meta-analyses on the association of angiogenesis growth factors with SICH patients’ functional outcome.

**Fig 7:**
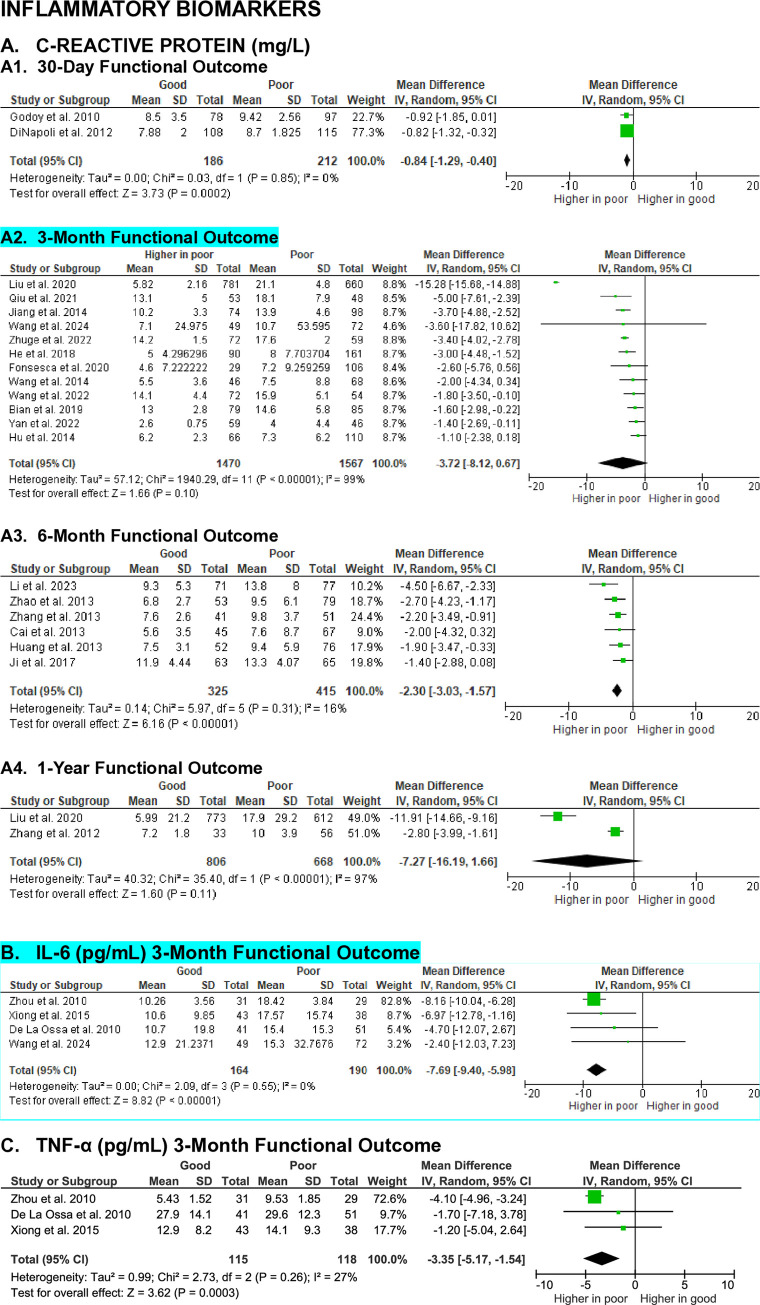
Meta-analyses on the association of inflammatory biomarkers with SICH patients’ functional outcome.

**Fig 8:**
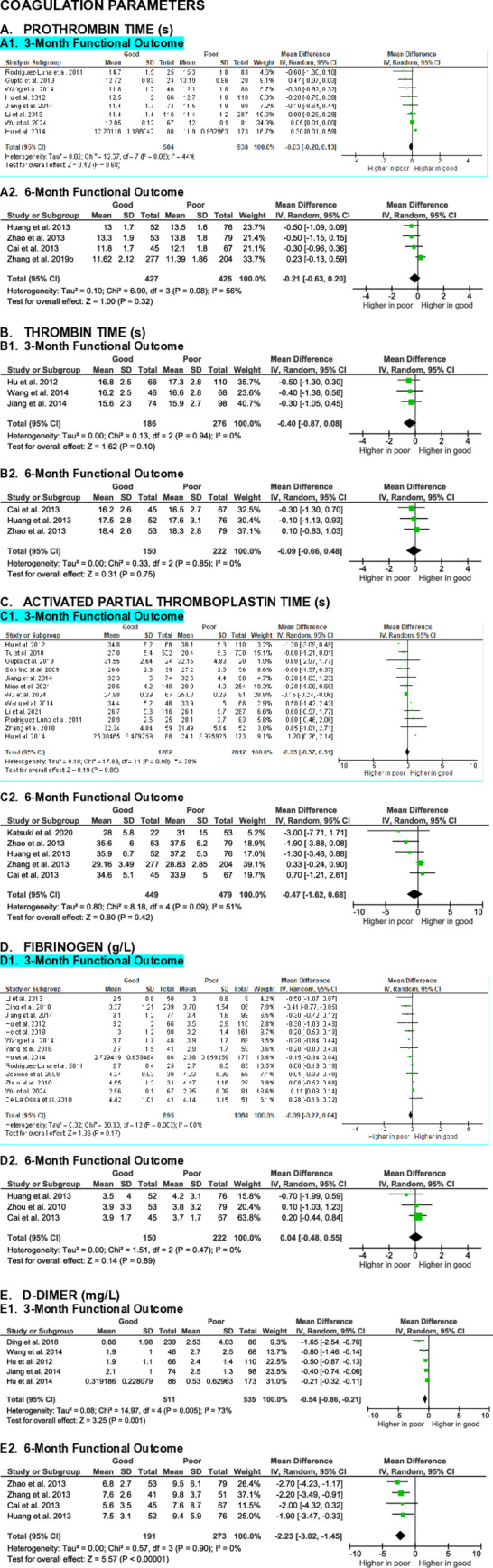
Meta-analyses on the association of coagulation parameters with SICH patients’ functional outcome.

**Fig 9:**
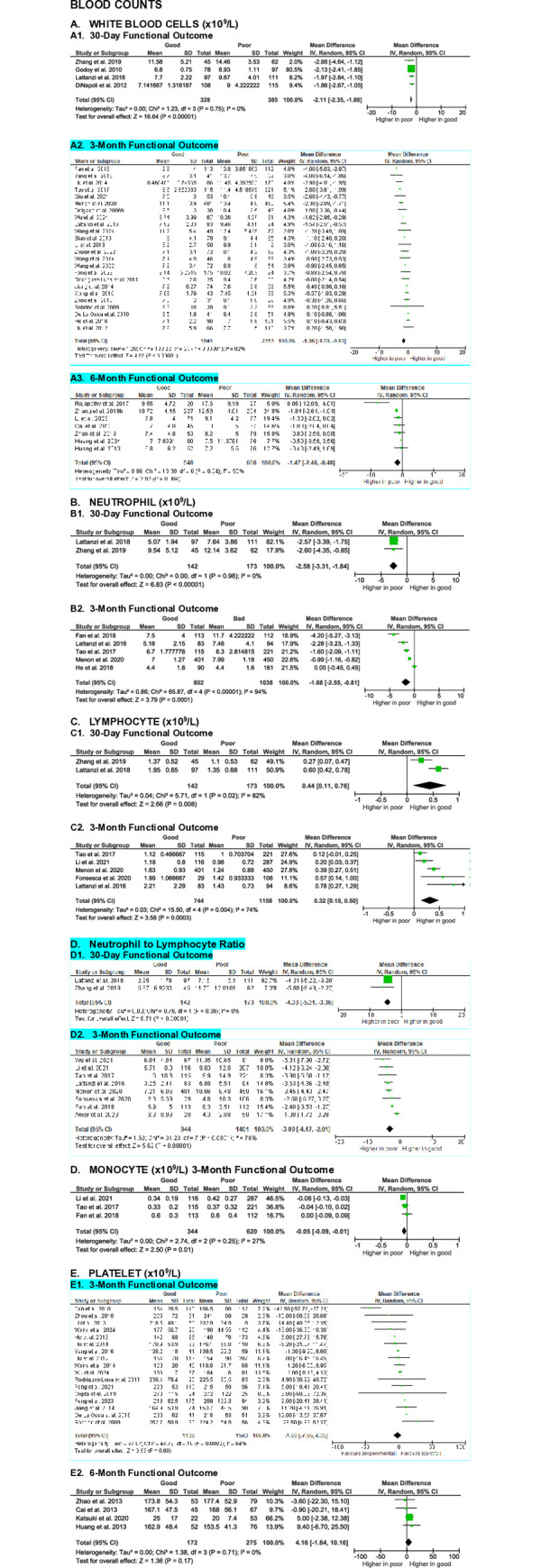
Meta-analyses on the association of blood counts with SICH patients’ functional outcome.

**Fig 10:**
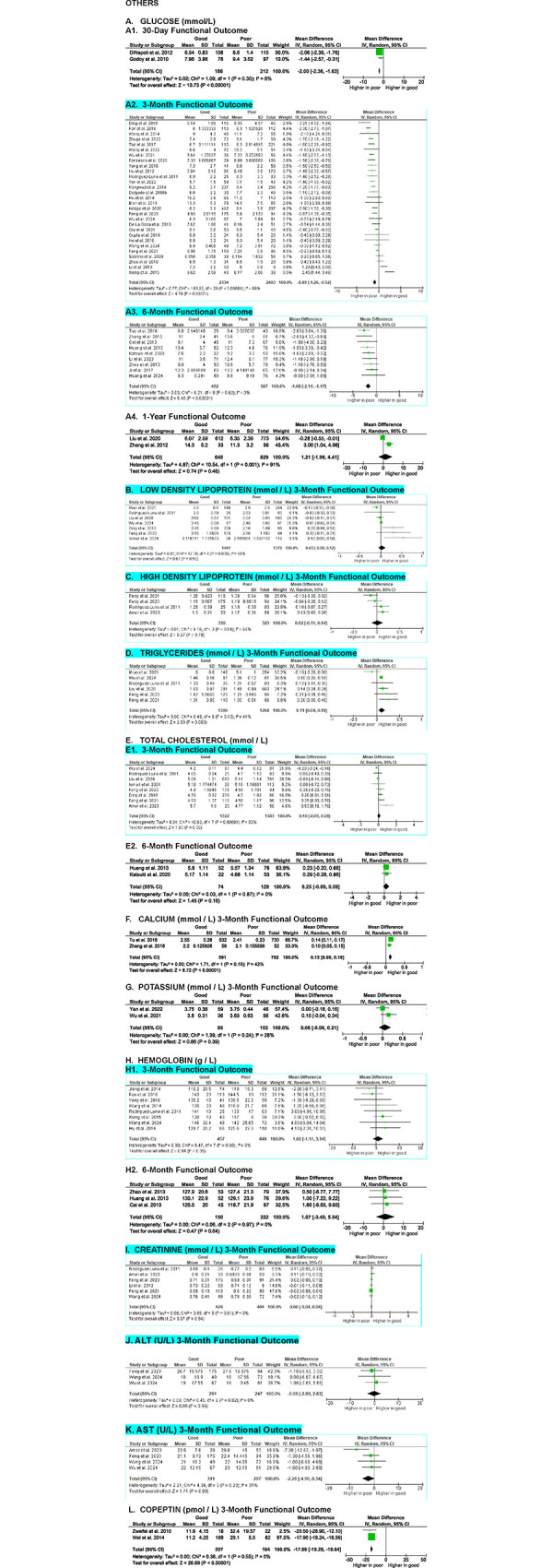
Meta-analyses on the association of other serum parameters with SICH patients’ functional outcome.

**Figs 6–10** present the summarized results of meta-analyses on various biomarkers associated with functional outcome in SICH patients at different evaluation time points, including 30-day, 3-month, 6-month, and 1-year assessments. The analyzed blood biomarkers were categorized into subgroups based on their biological roles or characteristics: angiogenesis factors, inflammatory biomarkers, coagulation parameters, blood counts, and other relevant biomarkers.

**Angiogenic factors.** A meta-analysis of two studies found no statistically significant differences in angiopoietin-1 levels between patients with good and poor outcomes at the 3-month evaluation time (MD 2.59 [-6.88, 12.05], z = 0.54, p = 0.59; I^2^ = 69%, p = 0.07) (**Fig 6A**). The predictive value of angiopoietin-1 for functional outcome was very low due to the small effect size.

Similarly, a meta-analysis of two studies revealed no statistically significant differences in vascular endothelial growth factor (VEGF) levels between patients with good and poor functional outcome at the 3-month evaluation time (MD 62.11 [9.24, 114.98], z = 2.30, p = 0.02; I^2^ = 0%, p = 0.99) (**Fig 6B**). The certainty of evidence was low, and the effect size was small.

#### Inflammatory biomarkers

A total of 21 studies evaluated the association between C-reactive protein (CRP) levels and functional outcome in SICH patients. Meta-analyses showed that at the 30-day (MD -0.84 [-1.29, -0.40], z = 3.73, p = 0.0002; I^2^ = 0%, p = 0.85) and 6-month (MD -2.30 [-3.03, -1.57], z = 6.16, p < 0.00001; I^2^ = 16%, p = 0.31) evaluation endpoints, SICH patients with good functional outcome had significantly lower CRP levels than those with poor functional outcome. However, at the 3-month (MD -3.72 [-8.12, -0.67], z = 1.66, p = 0.10; I^2^ = 99%, p < 0.00001) and 1-year (MD -7.27 [-16.19, 1.66], z = 1.60, p = 0.11; I^2^ = 97%, p < 0.00001) evaluation endpoints, the differences of CRP levels were statistically insignificant (**Fig 7A**). The predictive certainty of admission CRP levels toward short- and long-term functional outcome were low, with effect sizes ranging from small to medium.

A meta-analysis examined the association of interleukin-6 (IL-6) levels with 3-month functional outcome in SICH patients revealed significantly lower IL-6 levels in patients with good functional outcome compared to those with poor outcomes (MD -7.69 [-9.40, -5.98], z = 8.82, p < 0.00001; I^2^ = 0%, p = 0.55) (**Fig 7B**). Although the difference was significant with large effect size, the quality of evidence was moderate.

Similarly, a meta-analysis showed that tumor necrosis factor-alpha (TNF-α) levels in SICH patients with good functional outcome were significantly lower than patients with poor outcome at the 3-month evaluation point (MD -3.35 [-5.17, -1.54], z = 3.62, p = 0.0003; I^2^ = 27%, p = 0.26) (**Fig 7C**). The effect size was large, but the quality of evidence was moderate.

#### Coagulation parameters

Prothrombin time (PT), thrombin time (TT), activated partial thromboplastin time (aPTT), and fibrinogen levels have been evaluated in relation to the functional outcome of SICH patients. A total of 12 studies assessed the association between PT and functional outcome in SICH patients. Meta-analyses of these studies showed no significant differences in PT between patients with good functional outcome and those with poor outcome at the 3-month (MD -0.03 [-0.20, 0.13], z = 0.42, p = 0.68; I^2^ = 44%, p = 0.08) and 6-month (MD -0.21 [-0.63, 0.20], z = 1.00, p = 0.32; I^2^ = 56%, p = 0.08) evaluation endpoints (**Fig 8A**).

Six studies examined the relationship between TT and functional outcome in SICH patients. Meta-analyses indicated insignificance at both the 3-month (MD -0.40 [-0.87, 0.08], z = 1.62, p = 0.10; I^2^ = 0%, p = 0.94) and 6-month (MD -0.09 [-0.66, 0.48], z = 0.31, p = 0.75; I^2^ = 0%, p = 0.85) evaluation endpoints (**Fig 8B**).

Meta-analyses of 17 studies revealed no significant differences in aPTT between patients with good and poor functional outcomes at the 3-month (MD 0.03 [-0.37, 0.31], z = 0.19, p = 0.85; I^2^ = 38%, p = 0.08) and 6-month (MD -0.47 [-1.62, 0.68], z = 0.80, p = 0.42; I^2^ = 51%, p = 0.09) evaluation endpoints (**Fig 8C**).

Fifteen studies analyzed the association between fibrinogen levels and functional outcome in SICH patients. Meta-analyses showed that fibrinogen levels in patients with good functional outcome were not significantly different from those with poor outcome at the 3-month (MD -0.09 [-0.22, 0.04], z = 1.36, p = 0.17; I^2^ = 60%, p = 0.003) and 6-month (MD 0.04 [-0.48, 0.55], z = 0.14, p = 0.89; I^2^ = 0%, p = 0.47) evaluation endpoints (**Fig 8D**).

In contrast, meta-analyses of nine studies evaluating D-dimer levels showed that SICH patients with good functional outcome had significantly lower D-dimer levels compared to those with poor outcome at both the 3-month (MD -0.54 [-0.86, -0.21], z = 3.25, p = 0.001; I^2^ = 73%, p = 0.001) and 6-month (MD -2.23 [-3.02, -1.45], z = 5.57, p < 0.00001; I^2^ = 0%, p = 0.90) evaluation endpoints (**Fig 8E**).

The certainty of evidence of all blood coagulation parameters in predicting functional outcome in SICH patients were very low to low, with small effect sizes across all parameters.

#### Blood counts

Leukocyte, neutrophil, lymphocyte, monocyte, and platelet counts have been evaluated in relation to functional outcome in SICH patients. A meta-analysis of 35 studies on white blood cell (WBC) counts revealed that patients with good functional outcome had significantly lower WBC counts compared to patients with poor outcome at 30-day (MD -2.11 [-2.35, -1.86], z = 16.64, p < 0.00001; I^2^ = 0%, p = 0.75), 3-month (MD -1.36 [-1.90, -0.83], z = 4.99, p < 0.00001; I^2^ = 82%, p < 0.00001), and 6-month (MD -1.47 [-2.46, -0.48], z = 2.92, p = 0.004; I^2^ = 55%, p = 0.04) evaluation endpoints (**Fig 9A**). While the association at 30-day showed high certainty and a very large effect size, the certainty for the 3- and 6-month outcomes were low, with small effect sizes.

Meta-analyses of seven studies evaluating neutrophil counts showed that patients with good functional outcome had significantly lower neutrophil counts compared to those with poor outcome at 30-day (MD -2.58 [-3.31, -1.84], z = 6.83, p < 0.00001; I^2^ = 0%, p = 0.98) and 3-month (MD -1.68 [-2.55, -0.81], z = 3.79, p = 0.0001; I^2^ = 94%, p < 0.00001) evaluation endpoints (**Fig 9B**). The certainty of evidence were low, with medium effect sizes.

Seven studies assessed the relationship between lymphocyte counts and functional outcome. Meta-analyses showed that SICH patients with good functional outcome had significantly higher lymphocyte counts at 30-day (MD 0.44 [0.11, 0.76], z = 2.66, p = 0.008; I^2^ = 82%, p = 0.02) and 3-month (MD 0.32 [0.15, 0.50], z = 3.58, p = 0.0003; I^2^ = 74%, p = 0.004) evaluation endpoints (**Fig 9C**). However, the certainty of evidence were very low, with small to medium effect sizes.

Nine studies examined the association between Neutrophil-to-Lymphocyte Ratio (NLR) and functional outcome. Meta-analyses revealed that patients with poor outcomes had significantly higher NLR at 30-day (MD -4.33 [-5.31, -3.36], z = 8.71, p < 0.00001; I^2^ = 0%, p = 0.38) and 3-month (MD -3.09 [-4.17, -2.01], z = 5.62, p < 0.00001; I^2^ = 78%, p < 0.00001) endpoints (**Fig 9D**). The certainty of evidence were moderate for the 30-day outcome, with a large effect size, and very low for the 3-month outcome, with a small effect size.

Three studies found that patients with good functional outcome had significantly lower monocyte counts than those with poor outcome at the 3-month evaluation (MD -0.05 [-0.09, -0.01], z = 2.50, p = 0.01; I^2^ = 27%, p = 0.25) (**Fig 9E**). Despite being statistically significant, the certainty of evidence was low, with a small effect size.

Last, meta-analyses of 18 studies showed no significant differences on platelet counts between patients with good and poor functional outcome at the 3-month (MD -1.85 [-10.16, 6.47], z = 0.44, p = 0.66; I^2^ = 68%, p = 0.0001) and 6-month (MD 4.16 [-1.84, 10.16], z = 1.36, p = 0.17; I^2^ = 0%, p = 0.71) evaluation endpoints (**Fig 9F**). The certainty of evidence were very low, with small effect sizes.

#### Others

In addition to the previously mentioned biomarkers, several other values commonly measured during routine blood examinations have been associated with functional outcome in SICH patients. These include glucose, LDL, HDL, triglycerides, total cholesterol, calcium, potassium, copeptin, hemoglobin, and creatinine.

A meta-analysis of 41 studies examining glucose levels revealed that SICH patients with good functional outcome had significantly lower glucose levels than those with poor outcomes at 30-day (MD -2.00 [-2.36, -1.63], z = 10.73, p < 0.00001; I^2^ = 8%, p = 0.30), 3-month (MD -0.89 [-1.26, -0.52], z = 4.70, p < 0.0001; I^2^ = 86%, p < 0.00001), and 6-month (MD -1.68 [-2.19, -1.17], z = 6.48, p < 0.00001; I^2^ = 0%, p = 0.62) evaluation endpoints. However, no significant difference was found at the 1-year evaluation endpoint (MD 1.21 [-1.99, 4.41], z = 0.74, p = 0.46; I^2^ = 91%, p = 0.001) (**Fig 10A**). The evidence for predicting 90-day, 6-month, and 1-year outcomes were very low to low, with small effect sizes, while the evidence for 30-day functional outcome was high with a very large effect size.

Seven studies evaluating LDL levels showed no significant difference between patients with good and poor outcomes at the 3-month evaluation (MD 0.03 [-0.06, 0.12], z = 0.67, p = 0.50; I^2^ = 65%, p = 0.008) (**Fig 10B**).

A meta-analysis of four studies assessing HDL levels also revealed no significant differences between patients with good and poor functional outcome at the 3-month evaluation (MD -0.02 [-0.11, 0.14], z = 0.27, p = 0.78; I^2^ = 63%, p = 0.04) (**Fig 10C**).

Similarly, six studies analyzing triglyceride levels found no significant differences between patients with good and poor outcomes at the 3-month evaluation (MD 0.11 [0.04, 0.18], z = 2.99, p = 0.003; I^2^ = 41%, p = 0.13) (**Fig 10D**).

Ten studies on total cholesterol levels also showed no significant differences between patients with good and poor outcomes at 3-month (MD 0.10 [-0.09, 0.28], z = 1.03, p = 0.30; I^2^ = 83%, p < 0.00001) and 6-month (MD 0.25 [-0.09, 0.59], z = 1.45, p = 0.15; I^2^ = 0%, p = 0.87) evaluation endpoints (**Fig 10E**).

Meta-analyses of two studies indicated that patients with good outcomes had significantly higher calcium levels than those with poor outcomes at the 3-month evaluation (MD 0.13 [0.09, 0.16], z = 6.72, p < 0.00001; I^2^ = 42%, p = 0.19) (**Fig 10F**).

Two studies on potassium levels revealed no significant differences between patients with good and poor outcomes (MD 0.06 [-0.08, 0.21], z = 0.86, p = 0.39; I^2^ = 28%, p = 0.24) (**Fig 10G**).

Eleven studies analyzing hemoglobin levels found no significant differences between patients with good and poor outcomes at 3-month (MD 1.02 [-1.11, 3.14], z = 0.94, p = 0.35; I^2^ = 0%, p = 0.60) and 6-month (MD 1.07 [-3.40, 5.54], z = 0.47, p = 0.64; I^2^ = 0%, p = 0.97) evaluation endpoints (**Fig 10H**).

Meta-analyses of six studies on creatinine levels also showed no significant differences between patients with good and poor outcomes at 3-month evaluation times (MD 0.00 [-0.04, 0.04], z = 0.07, p = 0.94; I^2^ = 0%, p = 0.61) (**Fig 10I**).

Three studies revealed no significant differences in alanine aminotransferase (ALT) (MD -0.08 [-2.99, 2.83], z = 0.05, p = 0.96; I^2^ = 0%, p = 0.82) (**Fig 10J**) and aspartate aminotransferase (AST) (MD -2.28 [-4.90, 0.34], z = 1.71, p = 0.09; I^2^ = 31%, p = 0.23) (**Fig 10K)** between patients with good and poor outcomes at the 3-month evaluation points.

The certainty of evidence for the associations between LDL, HDL, triglycerides, total cholesterol, calcium, potassium, hemoglobin, creatinine, ALT, and AST levels and functional outcome were very low to low, with all effect sizes being small.

Last, two studies revealed that patients with good outcome had significantly lower copeptin levels than those with poor outcome at the 3-month evaluation (MD -17.96 [-19.28, -16.64], z = 26.69, p < 0.00001; I^2^ = 0%, p = 0.55) (**Fig 10L**). The certainty of evidence for this finding was high, with a very large effect size.

## Discussion

Despite advancements in the diagnostic and therapeutic management of SICH patients, the optimal approach remains a topic of ongoing debate [94, 95]. Decisions regarding surgical evacuation versus conservative treatment are often influenced by the surgeon’s experience and preferences. One strategy to reduce subjectivity in surgical decision-making for SICH patients involves the use of prognostic scoring systems to stratify risk and guide patient selection for surgical intervention [89, 96]. To date, no consensus has been established on a universally accepted scoring system or specific surgical threshold scores. Nonetheless, based on clinical experience, surgeons and patients’ families tend to favor conservative management when the patient’s risk of 30-day mortality is either very low or extremely high, regardless of the predicted functional outcome.

A commonly referenced prognostic tool is the intracerebral hemorrhage (ICH) score [11], which has generally been considered reliable. However, previous studies suggest that mortality outcomes may not solely correlate with age, Glasgow Coma Scale (GCS) score, hematoma volume, the presence of intraventricular hemorrhage (IVH), or infratentorial bleeding location [97–99]. As a result, ongoing efforts aim to improve the sensitivity and specificity of prognostic scoring systems for SICH patients. Additionally, specific biomarkers derived from blood or other specimens have been proposed as real-time indicators of a patient’s condition. Compared to prognostic models that rely primarily on clinical and radiological characteristics, biomarkers may provide a more accurate reflection of the patient’s prognosis. In this study, we conducted a meta-analysis of multiple studies investigating various blood biomarkers with potential as prognostic indicators in SICH patients.

### All evaluated inflammatory biomarkers are significantly associated with mortality and functional outcome; however, the certainty of evidence ranges from low to moderate

In SICH, the release of blood components into the brain parenchyma triggers immune and inflammatory responses. Both preclinical and clinical studies have highlighted the role of inflammation in disrupting the blood-brain barrier, causing edema, and leading to cell death. Activated microglia, occurring within one hour and persisting up to four weeks after the onset of SICH, release both pro-inflammatory and anti-inflammatory cytokines, indicating that inflammation plays a role in both the acute and chronic phases of SICH. Based on these findings, it is logical to hypothesize that inflammatory biomarker levels are significantly associated with mortality risk and functional outcome in SICH patients.

Supporting this hypothesis, meta-analyses showed CRP levels are significantly higher in non-surviving SICH patients (**Fig 2**). Except for CRP levels at two specific time points, the meta-analyses of inflammatory biomarkers and functional outcome in SICH patients demonstrated that CRP, IL-6, and TNF-α levels were significantly elevated in patients with poor functional outcome compared to those with good outcome (**Fig 7**). The lack of statistical significance in CRP levels at these two time points may be due to high heterogeneity in baseline serum CRP levels within the study population. Despite this, CRP levels generally tend to be higher in patients with poor functional outcome.

IL-6 is secreted during the inflammatory phase by various cells, including monocytes, neurons, and glial cells. Patients with early hematoma growth exhibit higher baseline serum IL-6 levels [100], which has also been correlated with the size of perihematomal hypodensity 3–4 days post-hemorrhage [101]. Based on these findings, it is reasonable to hypothesize that IL-6 levels may be associated with hematoma volume, explaining why IL-6 levels are higher in SICH patients with poorer outcomes compared to those with better outcomes. Nevertheless, the causal relationship between IL-6 levels and outcomes in SICH patients requires further investigation.

Similarly, a possible explanation for the correlation between TNF-α levels and SICH patients’ outcomes lies in the finding that patients with early hematoma growth and larger perihematomal hypodensity tend to have higher serum TNF-α levels [101, 102].

CRP is an acute-phase protein, primarily produced by hepatocytes in response to IL-1, IL-6, and TNF-α. CRP has been extensively studied for its pathophysiological roles in vascular diseases. Briefly, CRP induces inflammatory changes in endothelial and smooth muscle cells, contributing to endothelial dysfunction and the progression of atherosclerosis, which in turn facilitates thrombogenesis through the stimulation of macrophage tissue factor biosynthesis. Elevated CRP levels during the acute phase of stroke reflect the extent and severity of cerebral injury. A systematic review by Di Napoli et al. (2005) evaluated CRP levels at admission as a biomarker for risk and prognosis in ischemic stroke patients, concluding that elevated CRP enhances the predictive power of existing prognostic markers. Higher CRP concentrations were significantly associated with larger brain infarcts [103]. A comparative study by Modrego et al. (2008) demonstrated that baseline CRP levels, measured five hours after onset, predict CT evidence of brain edema in both hemorrhagic and ischemic stroke patients [104].

Although the associations between proinflammatory cytokines and patient outcomes are significant, the quality of evidence for these cytokines ranges from low to moderate. We hypothesize that this may be due to the variability in serum cytokine levels across different studies. Nonetheless, as shown in **Figs 2** and **7**, the studies consistently indicate higher levels of inflammatory cytokines in patients with poor outcomes. A previous systematic review by Montellano et al. (2021) investigated potential biomarkers for mortality and functional prognosis in ischemic stroke patients, concluding that inflammatory biomarkers such as CRP, IL-6, and TNF-α were inconsistently associated with poor outcomes [105]. However, it is important to note that this review included biomarker measurements up to 7-day after stroke onset and did not perform further statistical analysis.

In contrast to that review, our study exclusively included studies that evaluated biomarkers drawn within 24–72 hours after symptom onset, with most studies focusing on patients admitted within 24 hours of symptom onset. The inconsistent findings regarding inflammatory cytokine levels may be attributed to differences in the timing of biomarker evaluation, which could be influenced by the variability in patient admission times. Additionally, the potential impact of underlying comorbidities or inflammatory conditions unrelated to the vascular event cannot be excluded.

Plasma CRP concentrations have been shown to be chronically influenced by a wide range of disease states and physiological factors, such as heart disease, renal insufficiency, diabetes mellitus, obstructive sleep apnea, arterial hypertension, obesity, metabolic syndrome, frequent physical activity, alcohol consumption, high-protein diets, and depressive symptoms. Furthermore, CRP levels may be affected by genetic variations and the biological aging process [103]. Unfortunately, most of the studies included in our analysis did not adjust for these conditions in their analyses or risk stratifications, making it impossible to fully exclude patients with other underlying conditions from the data presented.

Despite these challenges, we believe that inflammatory biomarkers still reflect the severity of haemorrhagic stroke and can therefore be reliably used as prognostic indicators. This hypothesis is supported by the large effect sizes observed in the association between CRP levels and mortality, and between TNF-α levels and functional outcome in SICH patients. However, future studies and clinical applications will require standardized clinical cutoff points and consistent time intervals between blood sampling and stroke onset.

Determining whether elevated inflammatory biomarkers reflect heightened activity of inflammatory cascades leading to poor outcomes or whether excessive inflammation is merely a characteristic of severe SICH is beyond the scope of this study. Nevertheless, since inflammation is initiated and exacerbated by the presence of blood in the brain parenchyma, it is reasonable to consider early hematoma evacuation as a strategy to minimize inflammation [106]. Minimally invasive techniques for evacuating hematomas located more than 1 cm below the cortical surface may be crucial to reduce the risk of damaging healthy cortical areas when accessing deeper brain regions [107].

Consequently, as suggested in previous literature, the potential application of anti-inflammatory agents in SICH management warrants further investigation [108, 109]. Minocycline, an antibiotic that regulates iron metabolism and inhibits microglia activation, has shown neuroprotective effects in both in vitro and in vivo models of intracerebral hemorrhage (ICH), as evidenced by reduced brain damage and cell death [110]. A meta-analysis on the efficacy of Minocycline in patients with acute ischemic stroke (AIS) and SICH indicated that the drug improves functional independence [111]. Additionally, a study in an experimental ischemic stroke mouse model demonstrated that intraperitoneal injection of resolvin D2 nanoparticles, a derivative of docosahexaenoic acid (DHA) with anti-inflammatory properties, reduced inflammatory cytokines such as TNF-α, IL-6, and IL-1β, leading to a decrease in brain damage volume from 46% in the control group to 16% in the treated group [112].

#### Among all blood coagulation parameters evaluated in this study, only D-dimer showed a moderate association with 7-day mortality rates

Blood coagulation is a key physiological process that promotes hemostasis when bleeding occurs [113]. Given this, it is reasonable to hypothesize that blood coagulation factors may be significantly associated with outcomes in SICH patients. Coagulation pathways are divided into intrinsic, extrinsic, and common pathways based on specific interacting factors. The studies included in this meta-analysis investigated five blood coagulation parameters: prothrombin time (PT), thrombin time (TT), activated partial thromboplastin time (aPTT), serum fibrinogen, and serum D-dimer levels. aPTT reflects the intrinsic pathway, while PT, TT, and serum fibrinogen are part of the common pathway, shared by both the intrinsic and extrinsic pathways [114].

The results indicated that PT, TT, aPTT, and fibrinogen levels were not significantly associated with mortality in SICH patients. While serum fibrinogen was significantly associated with 3-month functional outcome, the effect size was small. D-dimer was the only parameter to show a large effect size in relation to 7-day, 30-day, and 3-month mortality, though moderate evidence was only observed for 7-day mortality. D-dimer is a by-product of the blood clotting process, formed when two platelets are bound together via D groups, which subsequently break apart, forming D-dimer. This marker is commonly used in the diagnosis of conditions such as pulmonary embolism and deep vein thrombosis, particularly in cases of low suspicion [115].

We suspect that serum D-dimer and fibrinogen levels in SICH patients may correlate with the size of ruptured blood vessels and hematoma volume. A larger hematoma volume could indicate the rupture of larger vessels, resulting in elevated serum fibrinogen levels. As is known, a larger hematoma volume is considered one of the primary clinical features associated with a worse prognosis [116, 117]. The low level of evidence for D-dimer and fibrinogen in predicting hemorrhagic stroke prognosis may be attributed to the low specificity of these markers. D-dimer and fibrinogen levels are elevated during the acute phase of hemorrhagic stroke, but D-dimer levels are also affected by various thrombotic and coagulation disorders, as well as acute thrombolytic treatment [118]. These comorbidities likely contributed to the high heterogeneity of D-dimer levels across studies.

#### Certain blood cell counts are significantly associated with mortality and functional outcome; however, the quality of evidence supporting these associations is low

An ideal blood biomarker is a value that can be obtained by simple, representative, accurate, and inexpensive measurement(s). This underlaid the importance of studies that evaluated the association of SICH patients’ outcomes with certain blood counts. WBC count is the total count of granulocytes, lymphocytes, and monocytes. WBC plays a major role in immune and inflammatory responses; therefore, increases in WBC counts have been indicated in infectious and inflammatory diseases. In support of the meta-analysis findings on inflammatory biomarkers, the meta-analysis on WBC revealed that WBC counts are higher in SICH patients who acquired good outcomes than those who acquired poor outcomes. On functional outcome, this finding is accompanied with the revelation of higher neutrophils and monocytes counts, and lower lymphocyte count in SICH patients who acquired poor functional outcome compared to those who acquired good functional outcome. Interestingly, only monocytes, not neutrophils and lymphocytes, are significantly higher in non-surviving SICH patients than those who survived. This suggests the increase of WBC in non-surviving patients is primarily due to the increase in monocytes’ number. In relation to their roles in inflammation and hematoma clearance, the increase in monocyte-derived macrophage and microglia in hematoma and perihematomal brain regions of SICH patients have been described [119, 120].

A significantly higher neutrophil count has been observed in patients with poor functional outcome, with a moderate effect size. Neutrophils are among the first leukocytes recruited from peripheral blood to the brain following an intracerebral hemorrhage (ICH). Leukocyte infiltration begins within 8 hours and increases further within 24 hours of ICH onset [121]. Neutrophils primarily act by releasing lactoferrin (LTF), an iron-binding protein that detoxifies hematomas. In vitro studies have shown that LTF reduces the cytotoxicity of lysed red blood cells and FeCl₃ on cultured brain cells. Additionally, systemic administration of LTF has been found to reduce brain edema and mitigate neurological deficits caused by ICH [122].

Despite their essential role, blood-derived inflammatory cells contribute significantly to secondary brain injury following ICH. Neutrophil-induced neurotoxicity involves several pathways, including the secretion of cytotoxic mediators, pro-inflammatory cytokines (such as TNF-α and IL-1β), upregulation of matrix metalloproteinases, excessive production of reactive oxygen species, and macrophage activation. These processes lead to increased capillary permeability, disruption of the blood-brain barrier, cellular swelling, hematoma growth, edema formation, elevated intracranial pressure, and ultimately, displacement of brain tissue, all of which adversely affect stroke recovery [121].

Several clinical studies have identified an early increase in peripheral neutrophils as a predictor of peri-hemorrhagic edema development, a radiological marker of secondary injury following cerebral hematoma, and a risk factor for early neurological deterioration and poor ICH outcomes [107, 123, 124]. Neutrophils are also implicated in the suppression of astrocytic and microglial responses, myelin degradation, and axonal damage in the peri-hematoma region, largely due to neutrophil-derived matrix metalloproteinases. Early inhibition of these metalloproteinases following ICH has been shown to provide neuroprotection, reduce glial activation and neuronal apoptosis, decrease injury volume, and improve neurobehavioral recovery [121].

Lower lymphocyte counts in poor patients had been associated with lymphocyte depletion in the circulation due to perihematomal infiltration. Although the exact mechanism remains unknown, admission lymphopenia had been associated with increased risk of infectious complications leading to poor outcome [125–129]. Furthermore, a previous study revealed the increase in SICH patients’ WBC count is strongly associated with stroke severity, larger baseline hematoma volume, and the presence of intraventricular hemorrhage (IVH) [130, 131]; thus, we speculate that the increase of WBC and their components in SICH patients with poor outcomes is correlated with SICH severity.

The neutrophil-to-lymphocyte ratio (NLR) is a composite index that provides valuable insights into the innate and adaptive branches of the immune system. An early elevation in NLR, driven by an increase in neutrophils and/or a decrease in lymphocytes, may indicate the severity of the hematoma. This, in turn, can serve as a reliable predictor of peri-hemorrhagic edema growth, infection risk, early neurological deterioration, mortality, and poor functional outcome [121]. Recent meta-analyses have demonstrated significantly higher NLR values in patients with adverse outcomes.

Although a significant association of white blood cells and its subset counts with SICH patients’ outcomes were identified, high certainty of evidence was not found. The variety in WBC background levels between individuals that fluctuate by 15% within 1 day might be one factor that has influenced the effect size and certainty of evidence in these studies. The sampling time of baseline WBC count between the included studies varied and ranged from 6h to 72h after symptom onset. Furthermore, possible existences of underlying comorbidities that might have affected blood count parameters could not be excluded [132]; therefore, future studies that consider every underlying comorbidities and determine cut-off points for baseline WBC are highly important.

Due to the roles of platelets as mediators of the coagulation cascade, it is logical to expect higher platelet counts in SICH patients with more severe hemorrhage. Surprisingly, the association between platelet counts in SICH patients and their outcomes is insignificant; thus, it is tempting to evaluate the need to administer coagulation factors and platelet transfusion in SICH patients. Regardless, our findings suggest that platelets and the evaluated coagulation factors are somewhat not significantly associated with SICH pathophysiology.

#### Glucose levels are significantly associated with mortality and functional outcome; however, strong evidence is only observed in analyses conducted at the 30-day evaluation endpoint

In addition to the above-mentioned values, meta-analyses were performed to examine other values that are often evaluated during routine blood examinations, including glucose, sodium, potassium, Hb, creatinine, LDL, HDL, triglyceride, total cholesterol, AST, ALT, and calcium. On both mortality and functional outcome, significantly higher glucose levels were identified in SICH patients with poor outcome compared good outcome. The direct correlation of blood glucose level with SICH patients’ mortality and poor functional outcome is unknown; nonetheless, stress hyperglycemia has been identified in both ischemic and hemorrhagic stroke patients and is suspected to represent the metabolic response to stress [133, 134]. Various hypotheses have been suggested to explain how stress hypoglycemia worsens the outcome of SICH patients, such as the possibility that stress hyperglycemia may represent stroke severity, inflammatory response, and neuro-hormonal disturbances [134–136]. Furthermore, stress hyperglycemia might also exacerbate lactate accumulation, intracellular acidosis, mitochondrial calcium influx, and blood-brain barrier destruction [134]. Further studies on animal models are useful to unravel the exact pathogenesis of stress hyperglycemia in ICH.

*Only S100β and copeptin demonstrated a high level of evidence as reliable prognostic markers for mortality and functional outcome*. This meta-analysis showed only S100β and copeptin are of high certainty to serve as prognosticators for hemorrhagic stroke patients. Our result is in line with Montellano et al. (2021) that concluded natriuretic peptides, copeptin, cortisol, procalcitonin, mannose-binding lectin, and adipocyte fatty acid-binding protein as the most consistent biomarkers associated with poor outcomes [105].

S100β is a member of calcium binding protein found predominately in astroglia and Schwann cells. It is thought as an important neurotropic factor during neurite growth and accumulation of this protein is associated with microtubule network [136]. Normally, S100β is not detectable in serum and its elevation has been associated with neurological damage and diseases, such as ischemic and hemorrhagic stroke, aneurysmal subarachnoid hemorrhage, Alzheimer’s disease, frontotemporal dementia, Down’s syndrome, epilepsy, sleep apnea syndrome, and melanoma [137, 138]. A study by Kanner et al. (2003) revealed that S100β serum elevation is related to blood brain barrier disruption, where S100β secreted by astrocytes into perivascular space leaks to the bloodstream [139]. Previous meta-analysis demonstrated the correlation between S100β elevation and infarct volume [140]. It has also been hypothesized that in the context of infarcted brain tissue, astroglia necrosis and membrane instability in the penumbra region around the ischemia cause cytosolic S100β to leak into the extracellular space, leading to the raising serum S100β concentrations [141]. Based on these facts, we hypothesized that higher S100β levels are related to global ischemic injury after initial intracerebral hemorrhage that subsequently lead to neurological decline and poorer outcome.

Copeptin is the C-terminal part of provasopressin, that might have a role as a sensitive surrogate marker for arginine-vasopressin (AVP) release. Adrenocorticotropic hormone and cortisol are produced by the hypothalamo-pituitary-adrenal axis, and AVP is a strong synergistic element in this process. Based on this fact, it has been hypothesized that copeptin elevation is correlated to the degree of neurohormonal stress response that represents the disease severity. Copeptin has been suggested to the reliable predictor of acute complications (including pneumonia) in stroke patients [105, 141]. Copeptin might depict intracerebral pathophysiology as this small protein is released to the bloodstream and readily bypass the blood-brain barrier. Edema formation is hypothesized to be the main mechanism relating copeptin with poor outcome. Animal studies showed arginine-vasopressin receptor inhibitors tolvaptan and conivaptan attenuated brain edema formation and led to better functional outcome [142, 143].

## Limitations

This meta-analysis detected heterogeneities and inconsistencies, likely due to the limited number of patients in the included studies. Most studies are retrospective or cross-sectional, not prospective or randomized control trials (RCTs); thus, multiple time point evaluations were not performed in the majority of studies. Moreover, confounding factors, such as the location of hemorrhage, comorbidities, admission fatality, presence of intraventricular hemorrhage, presence of hydrocephalus, and hematoma volume, could not be ruled out [144]. Lastly, different ICH management within studies may have influenced the results.

## Conclusion

This study highlights blood biomarkers that can potentially be used as prognosticators of SICH patients. It is interesting to notice that many blood biomarkers significantly associated with SICH patients’ outcomes are related to inflammatory responses. This suggests that modulation of inflammation might be essential to improve SICH patients’ prognosis. Furthermore, out of all blood biomarkers that were evaluated, only studies on S100β and copeptin had very high effect size and high certainty of evidence; thus, we confidently concluded that these two biomarkers are the most reliable blood biomarkers that can be used as prognosticators in SICH patients. Future studies to increase the certainties of evidence and effect size on other biomarkers are important.

## Supporting information

S1 Checklist PRISMA 2009 checklist. (DOC)

S1 Fig Risk of bias of the included studies. (DOCX)

S1 Table Characteristics of the screened studies. (DOCX)

S2 Table GRADE certainty of meta-analysis evidence. (DOCX)

S3 Table Effect size and imprecision of the meta-analysis. (DOCX)

## Abbreviations

Ang-1: Angiopoietin-1
aPTT: Activated Partial Thromboplastin Time
Cr: Creatinine
CRP: C-Reactive Protein
GOS: Glasgow Outcome Scale
Hb: Hemoglobin
HDL: High Density Lipoprotein
I^2^: Inconsistency Index
ICH: Intracerebral Hemorrhage
IL-6: Interleukin-6
IVH: Intraventricular Hemorrhage
LDL: Low Density Lipoprotein
mRS: modified Rankin Scale
PRISMA: Preferred Reporting Items for Systematic Reviews and Meta-Analyses
PT: Prothrombin time
S100β: S100 calcium-binding protein beta
SAH: Subarachnoid Hemorrhage
SICH: Spontaneous Intracerebral Hemorrhage
TF: Tissue Factor
TNF-α: Tumor Necrosis Factor-alpha
TT: Thrombin Time
VEGF: Vascular Endothelial Growth Factor
WBC: White Blood Cells
