# Correction: Potential blood biomarkers that can be used as prognosticators of spontaneous intracerebral hemorrhage: A systematic review and meta-analysis

**DOI:** 10.1371/journal.pone.0332555

**Published:** 2025-09-17

**Authors:** Aloysius Bagus Sasongko, Petra Octavian Perdana Wahjoepramono, Danny Halim, Jenifer Kiem Aviani, Achmad Adam, Yeo Tseng Tsai, Eka Julianta Wahjoepramono, Julius July, Tri Hanggono Achmad

After the publication of this article [1] several typographical and data entry errors were identified. A member of the *PLOS One* Editorial Board assessed the following updates and confirmed that they did not impact the conclusions of [1].

The meta-analysis of coagulation parameters depicted in Fig 3A incorrectly included [2]*,* which did not use the same measurement unit as the other included studies. Please see the correct Fig 3 here with [2] removed from the meta-analysis. Additionally, the second paragraph of the Coagulation Parameters section of the Mortality Outcomes section of the Results is corrected to: “Six studies evaluated the association between PT and mortality at the 7-day and 6-month endpoints in SICH patients. Meta-analysis results indicated that the differences in PT levels between non-surviving and surviving SICH patients were not statistically significant at 7-day (MD -0.12 [-0.52, 0.28], z = 0.59, p = 0.56; I^2^ = 37%, p = 0.19) or at 6-month (MD 0.11 [-0.38, 0.59], z = 0.43, p = 0.67; I² = 44%, p = 0.17) time points (Fig 3A).” This does not impact the interpretation of the analysis.

**Fig 3:**
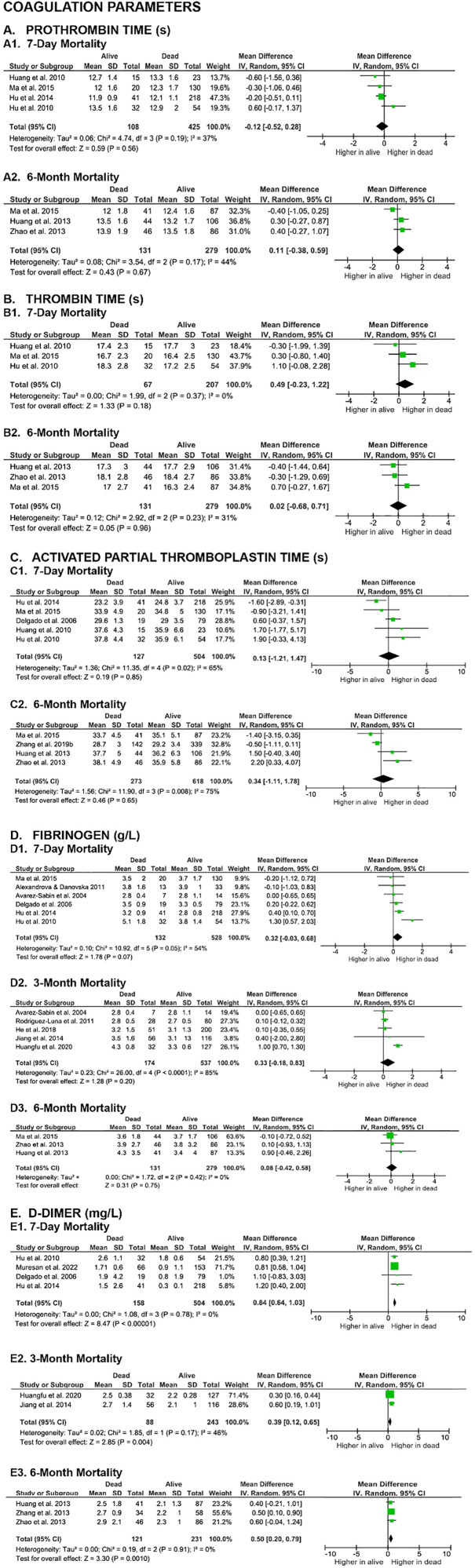
Forest plots of the association of coagulation parameters with SICH patients’ mortality.

There are errors in the reporting of several statistical analyses in the results due to data entry and typographical errors. The following corrections are made:

- The first paragraph of the Blood counts section of the Mortality outcomes section of the Results is updated to: “A meta-analysis of 23 studies revealed significantly higher baseline white blood cells (WBC) counts in non-surviving SICH patients at all evaluated time points, including 7-day (MD 1.61 [0.61, 2.61], z = 3.15, p = 0.002; I2 = 39%, p = 0.13), 30-day (MD 2.49 [2.12, 2.87], z = 13.07, p < 0.00001; I2 = 16%, p = 0.31), 3-month (MD 1.13 [0.23, 2.03], z = 2.46, p = 0.01; I2 = 85%, p < 0.00001), and 6-month (MD 1.57 [0.12, 3.01], z = 2.12, p = 0.03; I2 = 77%, p = 0.002). (Fig 4A).”
- The first sentence of the fifth paragraph of the Coagulation parameters section of the Functional outcome section of the Results is updated to: “In contrast, meta-analyses of nine studies evaluating D-dimer levels showed that SICH patients with good functional outcome had significantly lower D-dimer levels compared to those with poor outcome at both the 3-month (MD -0.54 [-0.86, -0.21], z = 3.25, p = 0.001; I² = 73%, p = 0.005) and 6-month (MD -2.23 [-3.02, -1.45], z = 5.57, p < 0.00001; I² = 0%, p = 0.90) evaluation endpoints (Fig 8E).”
- The sixth paragraph of the Blood counts section of the Functional outcome section of the Results is updated to: “Last, meta-analyses of 18 studies showed no significant differences on platelet counts between patients with good and poor functional outcome at the 3-month (MD -1.60 [-7.55, 4.35], z = 0.53, p = 0.60; I2 = 64%, p = 0.0002) and 6-month (MD 4.16 [-1.84, 10.16], z = 1.36, p = 0.17; I2 = 0%, p = 0.71) evaluation endpoints (Fig 9F). The certainty of evidence were very low, with small effect sizes.”

**Fig 4:**
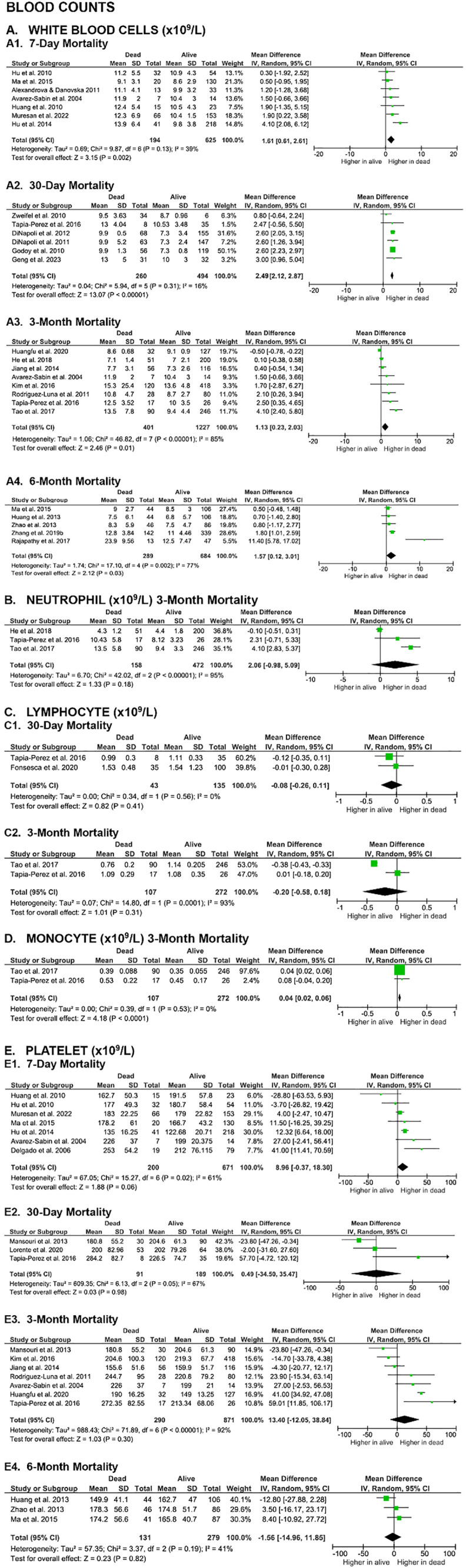
Meta-analyses on the association of blood counts with SICH patients’ mortality.

**Fig 5:**
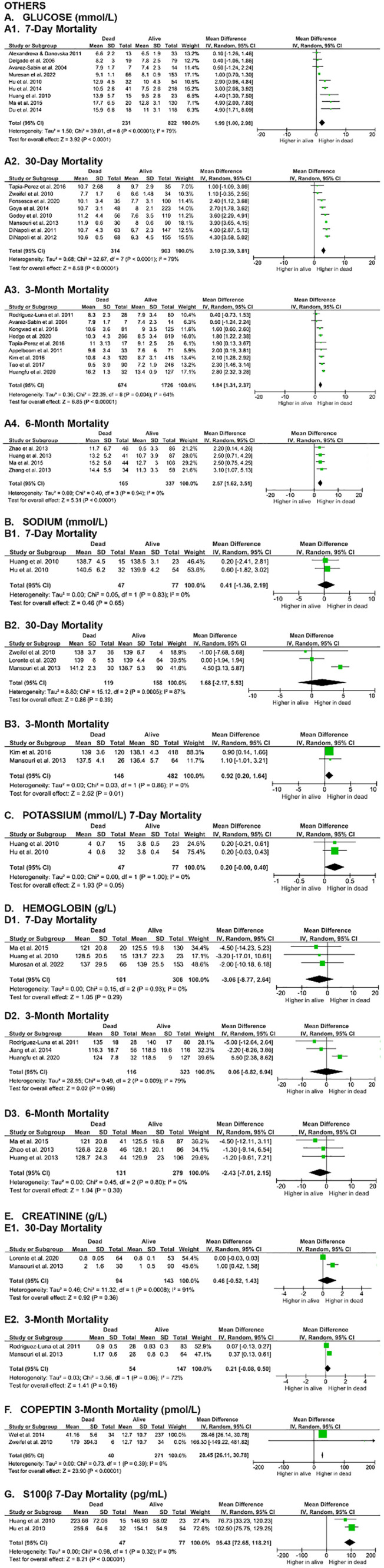
Meta-analyses on the association of other blood serum parameters with SICH patients’ mortality.

There were no errors in the reporting of the above analyses in the figures included in [1], however, panel F was not originally included in Fig 9, and the updated figure can be found here.

The effect of VEGF on 3-month functional outcomes, reported in Fig 6B, were incorrectly interpreted. The second paragraph of the Angiogenic factors section of the Functional outcome section of the Results is updated to: “A meta-analysis of two studies revealed statistically significant differences in vascular endothelial growth factor (VEGF) levels between patients with good and poor functional outcome at the 3-month evaluation time (MD 62.11 [9.24, 114.98], z = 2.30, p = 0.02; I^2^ = 0%, p = 0.99) (Fig 6B). Nevertheless, the certainty of evidence was low, and the effect size was small.”

In the fifth line of the 17th paragraph of the Discussion, the interpretation of the meta-analysis of white blood cells was reported incorrectly, and is updated to: “In support of the meta-analysis findings on inflammatory biomarkers, the meta-analysis on WBC revealed that WBC counts are higher in SICH patients with poor outcomes.”

Due to the suboptimal quality of Figs 4, 5, 8 and 10 affecting readability in [1], the updated figures can be found here with improved resolution.

**Fig 8:**
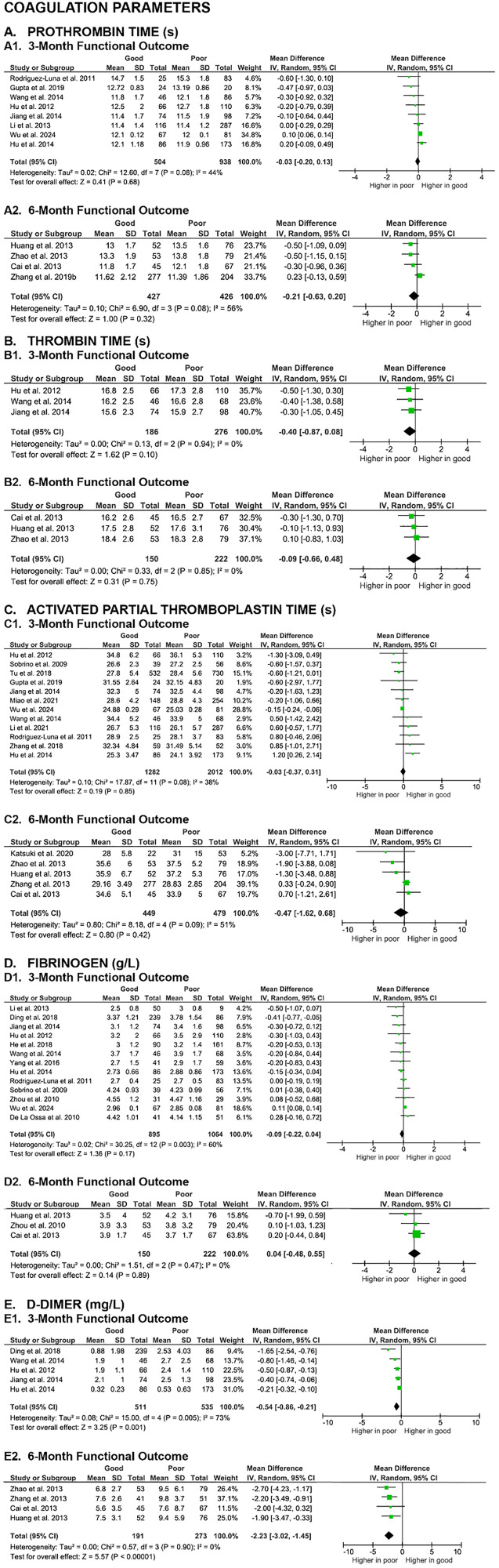
Meta-analyses on the association of coagulation parameters with SICH patients’ functional outcome.

**Fig 9:**
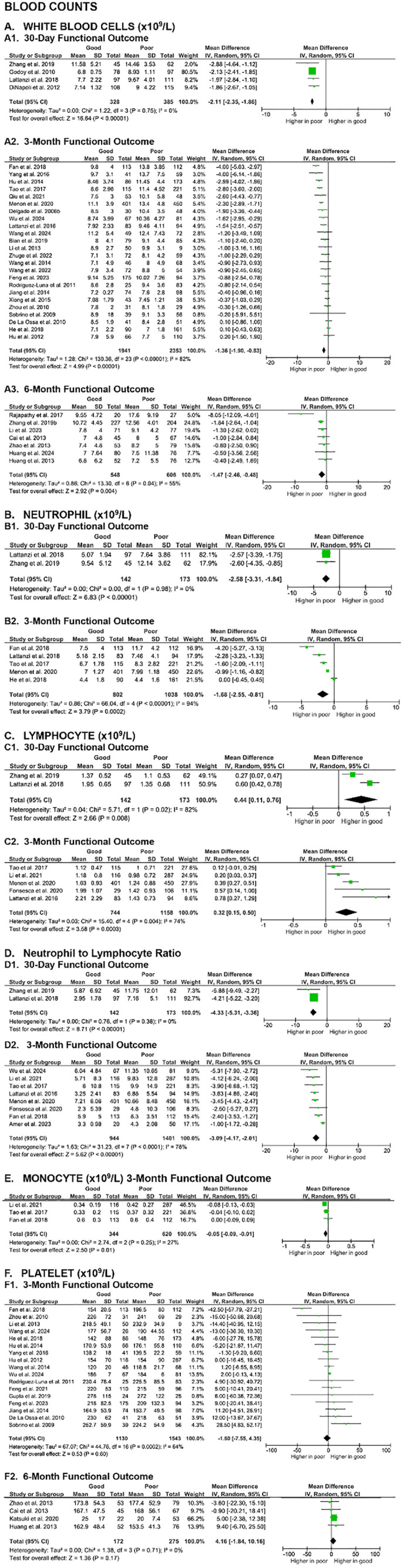
Meta-analyses on the association of blood counts with SICH patients’ functional outcome.

**Fig 10:**
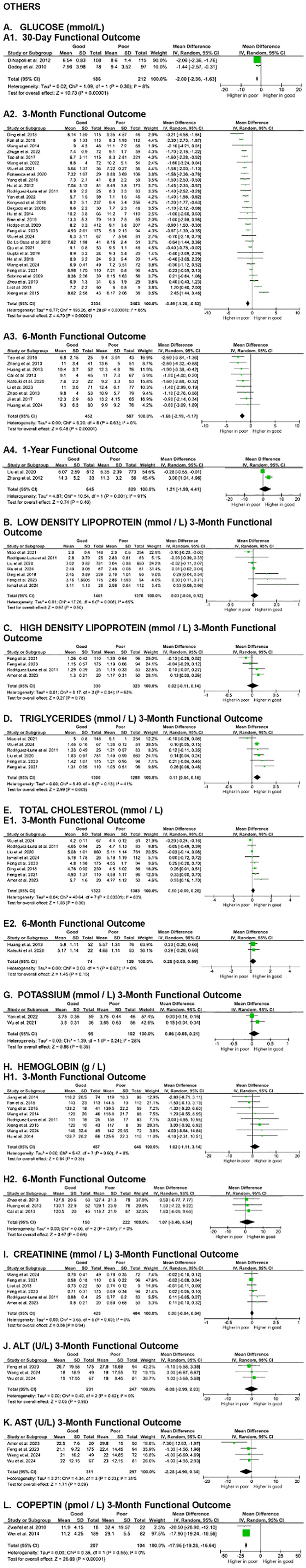
Meta-analyses on the association of other serum parameters with SICH patients’ functional outcome.

The authors apologize for the errors in the published article.
