# Supplementary figures and images for: Potential blood biomarkers that can be used as prognosticators of spontaneous intracerebral hemorrhage: A systematic review and meta-analysis
Source: PLoS One. 2025 Feb 19;20(2):e0315333. doi: 10.1371/journal.pone.0315333 (PMC11838903; doi:10.1371/journal.pone.0315333)

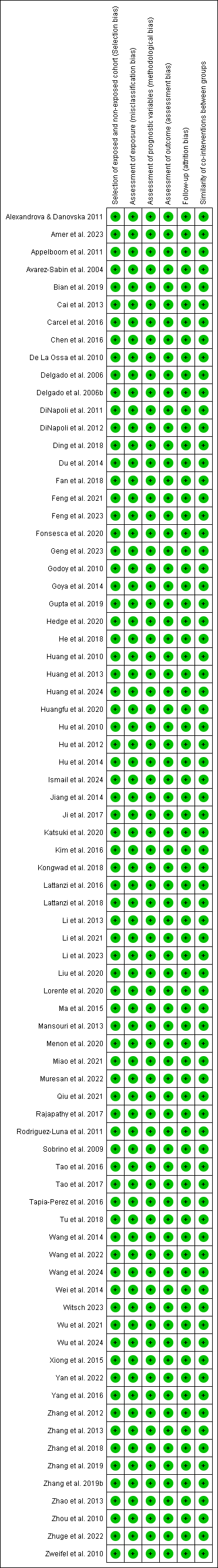

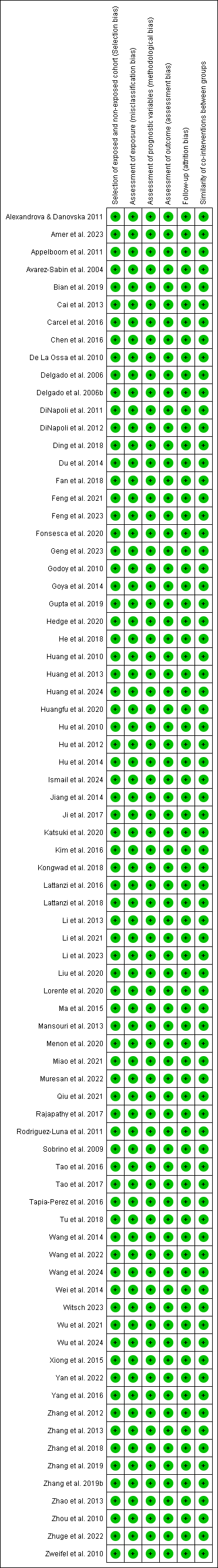

Supplement: S1 Fig — (DOCX) [file pone.0315333.s002.docx]
