# Supplementary material for: Potential blood biomarkers that can be used as prognosticators of spontaneous intracerebral hemorrhage: A systematic review and meta-analysis
Source: PLoS One. 2025 Feb 19;20(2):e0315333. doi: 10.1371/journal.pone.0315333 (PMC11838903; doi:10.1371/journal.pone.0315333)
Supplement: S1 Table — (DOCX) [file pone.0315333.s003.docx]

| **Supplemental Table 1.** Characteristic of the Screened Studies | | | | | | | | | | | |
| --- | --- | --- | --- | --- | --- | --- | --- | --- | --- | --- | --- |
| No | **Name (Year)**  **Country** | **Publication** | **Grouping (no. of patients)** | **Inclusion and Exclusion criteria** | **Etiologic and location of Stroke** | **Reported Outcomes** | **Method of assessment** | **Biomarkers corelation to outcome** | **Cut-off** | **Other confounding Factors** | **Reason for Exclusion** |
| 1 | Abilleira *et al.* (2003)  Spain | Abilleira S, Montaner J, Molina CA, Monasterio J, Castillo J, Alvarez-Sabin J. Matrix metalloproteinase-9 concentration after spontaneous intracerebral hemorrhage. *J Neurosurg* 2003; 99:65-70 | Total: 57  Deep ICH (38)  Lobar ICH (19) | **Inclusion:**  Patients with suspected spontaneous supratentorial ICH, which was diagnosed on the basis of findings on CT scans obtained within the first 24 hours after onset of stroke symptoms  **Exclusion:**  Patients with presence of  known acute or chronic infections, inflammatory or malignant diseases, or immunosuppressive treatment | Deep sICH  Lobar sICH | Neurological worsening  PHE volume >19.1 mL | Decrease in one or more point in CSS  CT scan | MMP-9 | Range | + Baseline ICH volume  + Baseline CSS score  + Smoking  +Chronic alcoholism | Not reporting outcome of interest |
| 2 | Adam *et al.* (2017)  Indonesia | Adam A, Ferry B, Atman D. Korelasi antara nilai S100beta pre dan post kraniotomi evakuasi pendarahan intraserebral spontan dengan luaran. *JNI* 2017; 7(1):8-14 | Total: 40  S100B <0.22 (17)  S100B >0.22 (23) | Inclusion:  + Spontaneous ICH  + Undergone craniotomy evacuation  + Age: 18-80 years  + Operated <24 h after admission  Exclusion:  + Traumatic cause  + Chronic neurodegeneration  + Amyotrophic lateral sclerosis  + Multiple sclerosis  + Melanoma maligna  + Encephalopathy hepaticum  + Bipolar disorder  + Celiac disease | sICH | + In hospital mortality  + Correlation to GOS  + Correlation to ICH volume | + GOS  + Imaging | + S100B | <0.22, >0.22 | Not mentioned | Not reporting defined time for outcome assessment |
| 3 | Agnihotri *et al.* (2018)  USA | Agnihotri S, Czap A, Staff I, Fortunato G, McCullough LD. Peripheral leukocyte counts and outcomes after intracerebral hemorrhage. *J Neuroinflam* 2011; 8:160 | Total: | **Inclusion:**  + Patients with sICH  **Exclusion:**  + SAH  + Hemorrhages secondary to trauma, malformations, coagulopathy, tumor | sICH | + In hospital mortality  + 1 year mortality  + 3-month functional outcome | + Mortality  + Modified Barthel Index | + Leukocyte counts | Not stated | Not mentioned | Not reporting data of biomarker range and number of patients |
| 4 | Alatas *et al.* (2015)  Turkey | Alatas OD, Gurger M, Atescelik M, Yildiz M, Demir FC, et al. Aneuron-specific enolase, S100 Calcium binding protein B, and heat shock protein 70 levels in patients with intracranial hemorrhage. *Medicine* 2015; 94:45. Doi: 10.1097/MD.0000000000002007 | Total: 67  sICH (35)  Healthy (32) | **Inclusion:**  + Older than 18 years  + Presented to mergency Department of Firat University Medical School within 24 h after onset  + Nontraumatic ICH  **Exclusion:**  + Undergoing intracranial procedures  + Using anticoagulant agents  + With malignancy, uremia, liver cirrhosis, SAH, trauma | sICH | + Admission Neurological state  + Admission ICH volume  + Admission functional outcome  + In hospital mortality | + GCS score  + Imaging  + NIHSS  + Mortality | + S100B  + HSP70  + NSE | Not stated | Not mentioned | Not reporting data of biomarker range and number of patients |
| 5 | Alexandrova & Danovska (2011)  Bulgaria | Alexandrova ML, Danovska MP. Serum C-reactive protein and lipid hydroperoxides in predicting short-term clinical outcome after spontaneous intracerebral hemorrhage. *J Clin Neurosci* 2011; 18:247-52. Doi: 10.1016/j.jocn.2010.07.125 | Total: 46 | **Inclusion:**  + Patients with sICh admitted to Department of Neurology Oct 2006-May 2007  **Exclusion:**  + Admission > 48H after ictus  + History of rheumatoid arthritis, concomitant malignant disease, myocardial infarction, chronic kidney insufficiency, hematological disease | sICH (basal ganglia, lobar, subtentorial) | + 7-day mortality  + Clinical outcome at discharge | + Mortality  + Mathew stroke score  + NIHSS  + mRS | Admission biochemistry:  + WBC  + Glucose  +Chloesterol  + Fibrinogen  + TG  + CRP  +ROOH | Range | + Gender  + Comorbidities (arterial hypertension, ischemic heart disease, diabetes mellitus)  + Alcohol abuse  + Smoking  + MSS admission  + Initial MSS  + Initial GCS  + NIHSS score day 7  + Location of sICH  + Hemorrhage volume |  |
| 6 | Avarez-Sabin *et al.* (2004)  Spain | Alvarez-Sabin J, Delgado P, Abilleira S, Molina CA, Arenillas J, et al. Temporal profile of matrix metalloproteinases and their inhibitors after spontaneous intracerebral hemorrhage: Relationship to clinical and radiological outcome. *Stroke* 2004; 35:1316-22. Doi: 10.1161/01.STR.0000126827.69286.90 | Total: 21  Alive 48h = 19  Alive 7d = 16  Allive 3 mo = 14 | **Inclusion:**  + Patients with sICH  + Admitted to emergency department <24H after ictus  **Exclusion:**  + Hemorrhage secondary to vascular malformation, impaired coagulation, head trauma, hemorrhagic infarction, tumor lesion | Supratentorial sICH | + Alive at 48h  + Alive at 7d  + Alive at 3 mo | + mortality | Biomarkers only reported in 3 m.o. mortality:  + Leukocyte  + Platelet  + INR  + Fibrinogen  + Glucose  + MMP-2  + MMP-3  + MMP-9  + TIMP-1  + TIMP-2 | Range | + Gender  + Age  + Previous high blood pressure  + Diabetes  +Liver disease  + Antiplatelet therapy  + Baseline GCS  + Baseline NIHSS  + Blood pressure  + Temperature  + ICH volume  + PE volume |  |
| 7 | Appelboom *et al.* (2011)  New York | Appelboom G, Piaza MA, Hwang BY, carpenter A, Bruce SS, et al. Severity of intraventricular extension correlates with level of admission glucose after intracerebral hemorrhage. *Stroke* 2011; 42:1883-8. Doi: 10.1161/STROKEAHA.110.608166 | Total: 104 | **Inclusion:**  **+** Patients with ICH diagnosed by CT scan  **Exclusion:**  + Age < 18  + Secondary ICH due to malignancy, trauma, hemorrhagic conversion, another primary bleeding event  + Incomplete data | Supratentorial and infratentorial ICH | + Discharge mortality  + 3 m.o. mortality | + Mortality | + Admission glucose | Range | + Diabetes mellitus  + Admission GCS  + Etiology  + Admission hematoma size  + Admission IVH  + Admission HCP  + Admission ICH score  + EVD  + Shunt  + Intrathecal tPA  + Hematoma evacuation |  |
| 8 | Aydin *et al.* (2018)  Turkey | Aydin I, Algin A, Poyraz MK, Yumrutas O. Diagnostic value of serum glial fibrillary acidic protein and S100B serum levels in emergency medicine patients with traumatic vs nontraumatic intracerebral hemorrhage. *Niger J Clin Pract* 2018; 21:1645-50. | Total : 20  Non-traumati (6)  Traumatic (14) | **Inclusion:**  + Computed tomography (CT) scan taken of Emergency Department  + Volunteers who signed informed consent form  + 30 totally healthy individuals were taken as the control group  **Exclusion:**  + Patients without computerized brain tomography  + Spontaneous SAH  + Those who do not agree to participate | Non-traumatic and traumatic ICH | + In hospital mortality | + mortality | + GFAP  + S100B | Range |  | Included traumatic cause of hemorrhage |
| 9 | Behrouz *et al.* 2015 | Behrouz R, Hafeez S, Miller CM. Admission leukocytosis in intracerebral hemorrhage: associated factors and prognostic implications. *Neurocrit Care* 2015: Doi: 10.1007/s12028-015-0128-7 | Total: 128  Leukocytosis (53)  No leukocytosis (75) | **Inclusion:**  + Non-traumatic ICH patients  **Exclusion:**  + Patients related to tumor, aneurysm, or arteriovenous malformation  + Undergone craniotomy for hematoma evacuation  + Chronic leukocytosis  + Taking glucocorticoids prior to presentation | Nontraumatic ICH  Location:  + Basal ganglia  + Brainstem  + Cerebellum  + Lobar  + Thalamus | + Poor outcome  + IVH  + Volume  + Graeb  + HR_0_  + GCS_0_  + T_24_ | mRS (> 5) | + Leukocytosis | WBC <11,000/uL | None | Biomarkers not reported in mean or range median |
| 10 | Behrouz et al. (2015)  USA | Behrouz R, Hafeez S, Mutgi SA, Zakaria A, Miller CM. Hypomagnesemia in intracerebral hemorrhage. *World Neurosurg* 2015. Doi: 10.1016/j.wneu.2015.08.036 | Total: 128  HMG_0_ = 43  NMG_0_ = 85 | **Inclusion:**  Non-traumatic ICH treated at the Ohio State University between 2011-2013  **Exclusion:**  + Secondary Ich to tumor, aneurysm, arteriovenous malformations  + underwent emergency craniotomy for hematoma evacuation | + Supratentorial and infratentorial sICH | At admission:  + ICH volume  + GCS median  + IVH presence  + ICH score  + Functional outcome | + ICH score  + mRS | + Hypomagnasemia | + admission Mg level < 0.7mmol /L | None | Outcomes were measured during admission |
| 11 | Bender *et al.* (2020)  Germany | Bender M, Haferkorn K, Friedrich M, Uhl E, Stein M. Impact of early C-reactive protein/albumin ratio on intra-hospital mortality among patients with spontaneous intracerebral hemorrhage. *J clin Med* 2020; 9:1236. Doi: 10.3390/jcm9041236 |  | **Inclusion:**  + All sICH patients between 2008-2017  **Exclusion :**  + Traumatic ICH  + ICH secondary to vascular malformation, neoplasia  + Present of acute and/or chronic liver failure  + Age <18 years | + sICH | + Intrahospital mortality 30-day mortality | + Mortality | + WBC count  + Hemoglobin  + Hematocrit  + Cholinesterase  + Blood glucose  + Serum lactate  + Troponin I  + Cortisol  + CRP  + Albumin  + CRP/Albumin ratio | Mean (SD) | + Gender  + BMI  + Initial GCS  + APACHE II Socre  + Comorbidities (hypertension, pulmonary disease, arrhyumia, coronary arteri disease, heart failure, cardiosurgical intervention, renal insufficiency, diabetes mellitus, history of ischemic stroke, ICH, history of cancer)  + Treatments | Not reporting defined time for outcome assessment |
| 12 | Bernstein *et a.* (2018)  USA | Bernstein JE, Savla P, Dong F, Zampella B, Wiginton IV JG, Miulli DE, et al. Inflammatory markers and severity of intracerebral hemorrhage. *Cureus* 2018; 10(10): e3529. Doi: 10.7759/cureus.3529 | Total: | **Inclusion:**  + Diagnosis of ICH or IVH without trauma, vascular abnormality, hemorrhagic transformation of ischemic stroke  + Age >18 yrs  + Admitted to ICU minimum 24hrs within | + sICH | + Mortality | Mortality | +TNF alpha  + Homocystein  + CRP  +VEGF | Meand (SD) | + Change in ICH size  + GCS on admission + Midline shift  + ICH score on admission  + Early neurologic decline | Not reporting defined time for outcome assessment |
| 13 | Bernstein et al. (2021)  USA | Bernstein JE, Browne JD, Savla P, Wiginton IV J, Pathana T, Miulli DE, et a. Inflammatory markers in severity of intracerebral hemorrhage II: A follow up study. *Cureus* 2021; 13(1): e12605. Doi: 10.7759/cureus.12605 | Total: 99 | **Inclusion:**  + ICH or IVH patients without trauma, vascular abnormality, hemorrhagic transformation of ischemic stroke  + Age >18 | sICH | Mortality |  | +TNf-a  + Homocysteine  + CRP  + VEGF  +CAR | Correlation | + ICH score | Not reporting data of biomarker range and number of patients |
| 14 | Bhatia et al (2013)  India | Bhatia R, Singh H, Singh S, Padma MV, Prasad K, Tripati M, et a. A prospective study of in-hospital mortality and discharge outcome in spontaneous intracerebral hemorrhage. *Neurol India* 2013; 61(3):244-9. | Total: 214  Survived = 144  Died = 70 | **Inclusion:**  all consecutive patients  of spontaneous ICH admitted under stroke services of the neurology department at All India Institute of Medical Sciences, New Delhi, were recruited  **Exclusion:**  patients with subdural and epidural hematoma,  aneurysmal, arteriovenous malformation (AVM),  anticoagulant or coagulopathy-related hemorrhage and  patients who denied informed consent | sICH supratentorial and infratentorial | + In hospital mortality + functional outcome at discharge | + mortality  + mRS | + Urea  + Creatinine  + Sodium  + Potassium  + Blood sugar | Mean (SD) | + Gender  + Hypertension  + Diabetes  + Dyslipidemia  + Smoking  +Alcohol  + Past stroke  + GCS < 8  + Infection  + NIHSS  + ICH Score  + location of hemoorahge  + Midline shift  + IVH  + Hydrocephalus  + Surgery | Not reporting defined time for outcome assessment |
| 15 | Cai *et a.*  (2020)  China | Cai B, peng L, Wang Z, Zhang M, Peng B. Association between serum lipid and hematoma expansion after Spontaneous intracerebral hemorrhage in Chinese patients. *J Stroke Cerebrovas Dis* 2020. 2020:104793. Doi: 10.1016/j.jstrokecerebrovasdis.2020.104793 | Total = 157  Hematoma growth (45)  No-Hg (112) | **Inclusion:**  (1) age of 18-90 years; (2)  baseline brain computed tomography (CT) performed  within 6 hours postictus of ICH; (3) follow-up CT  obtained within 48 hours after initial CT  **Exclusion**  subarachnoid hemorrhage, subdural hemorrhage, and  hemorrhage due to trauma or brain tumors | sICH | + Hematoma growth | + Imaging | +TC  + LDL-C  +HDL-C  +TG  +LDL-C/TC ratio  +LDL-C/HDL-C ratio | Mean (SD) | + GCS admission  + Hypertension  + Diabetes mellitus  + Smoking  + Antiplatelet | May included cases of aneurysm, vascular malformation, hemorrhagic of ischemic stroke |
| 16 | Carcel *et al.* (2016)  International | Carcel C, Sato S, Zheng D, Heeley E, Arima H, Yang J, et a. Prognostic significance of hyponatremia in acute intracerebral hemorrhage: Pooled analysis of the intensive blood pressure reduction in acute cerebral hemorrhage trial studies. *Neurol Crit Care* 2016; 44(7): 1388-94. Doi: 10.1097/CCM.0000000000001628 | Hyponatremia (349)  Without hyponatremia (2653) | **Inclusion:**  + Spontaneous ICH within 6hrs of onset  + Elevated systolic blood pressure (150-220)  **Exclusion:**  structural cerebral cause for the ICH, assessed as having a very high likelihood of death within the next 24 hours (e.g., massive ICH with midline  shift of hemisphere or deep coma at presentation defned as GCS score of 3–5) or if early surgery to evacuate the ICH was planned | sICH  + Lobar  + Deep  + Brain stem  + cerebellum | + 90-day mortality  + 90 day major disability | +Mortality  + mRS 3-5 | +Hyponatremia | Sodium <135 mEq/L | + Gender  + comorbidities (ICH, ischemic stroke, acute coronary syndrome, hypertension, diabetes mellitus)  + medications  + NIHSS  + GCS Score  + Location of ICH  + IVH |  |
| 17 | Castelazzi et a. 2009  Italy | Castellazzi M, Tamborino C, De Santis G, Garofano F, Lupato A, Ramponi V, et al. Timing of serum active MMP-9 and MMP-2 levels in acute and subacute phases after spontaneous intracerebral hemorrhage. *Act Neurochirurgica Suppl* 2010; 106:137-40. Doi: 10.1007/978-3-211-98811-4_24 | Total: 28 | **Inclusion:**  acute supratentorial  SICH proven by an admission non-enhanced computed tomography (CT) scan carried out within 24 h of onset  **Exclusion:**  (1) occurrence of infratentorial hemorrhage; (2) hematoma  related to tumor, trauma, coagulopathy, aneurysms, vascular  malformations; (3) hemorrhagic transformation of brain  infarction; (4) intraventricular extension of hemorrhage; (5)  surgical hematoma evacuation performed after the frst noncontrast CT scans; (6) age <20 years; or (7) evidence of pregnancy | sICH  Supratentorial | +hematoma volume 24h, 48h, 7 d  + Edema volume 24h, 48h, 7d | + imaging | + MMP-9  + MMP-2 | Correlation | Not stated | Not reporting data of biomarker range and number of patients |
| 18 | Castillo et al. (2002)  Spain | Castillo J, Davalos A, Alvarez-Sabin J, Pumar JM, Leira R, et al. Molecular signatures of brain injury after intracerebral hemorrhage. *Neurology* 2002; 58:624-9. | Total: 124  Good outcome = 71  Poor outcome = 53 | **Inclusion:**  time from the onset of symptoms to admission of <24 hours (in those with strokes present on awakening, time of onset was taken as the time when the patient was last  seen normal); age <80 years; and absence of stupor or coma  **Exclusion:**  + Head trauma  + congenital or acquired coagulation abnormalities  + Head Ct scan showing tumor, arteriovenous malformation  + Surgical treatment of ICH  + Death befor 3-mo monitoring period had ended | sICH supratentorial | + 3-months functional outcome | + Canadian Stroke Scale (CSS)  Good >7  Poor < 7 | + glutamate  + IL-4  +IL-6  + IL-10  + TNF-a  + ICAM-1  + VCAM-1  + serum glucose | Mean (SD) | History of hypertension  + CSS on admission + Initial volume of ICH  + Body temperature  + Blood pressure | Not reporting outcome of interest |
| 19 | Chan et al. (2012)  Hong Kong | Chan CP, Jiang H, Leung L, Wan W, Cheng N, Ip W, et a. Multiple artherosclerosis-related biomarkers associated with short- and long-term mortality after stroke. *Clin Biochem* 2012; 45: 1308-15. Doi: 10.1016/j.clinbiochem.2012.06.014 | Total: 92 | **Inclusion:**  + age >18 years  + Hemorrhagic or ischemic stroke  **Exclusion:**  + Trauma  + Meningitis  + Encephalitis  + Hypertensive encephalopathy  + Intracranial tumor  + Seizure, bell palsy  + Migraine  + Metabolic disturbances  + Post-cardiac arrest  + Endocrine disorders  = Renal failure  + Psychiatric syndrome | Hemorrhagic or ischemic stroke | + 6-month mortality  + 5-year mortality | Mortality | + Lymphocyte  + Monocyte  +Neutrophil  +Platelet  +WBC  + Crp  + Lipocarlin-2  MMP9 mRNA  + MOP | Mean (SD) | + Age  + Gender  + Smoking  + Hypertension  + DM  + Ischemic heart disease  + Hyperlipidemia  + post-stroke mRS + GCS  +NIHSS | Included ischemic stroke |
| 20 | Chang et al. (2018)  USA | Chang JJ, Katsanos AH, Khorchid Y, Dillard K, Kerro A, Burgess LG, Goyal N, Alexandrov AW, et al. Higher low-density lipoprotein cholesterol levels are associated with decreased mortality in patients with intracerebral hemorrhage. *Artherosclerosis* 2018; 269: 14-20. Doi: 10.1016/j.atherosclerosis.2017.12.008 | Tota= 672 | **Inclusion:**  Acute, spontaneous, non-traumatic ICH  + Age >18  **Exclusion:**  + Traumatic, metastatic lesion, venous sinus thromobosis, ICH from vascular lesions  + ICH due to supratherapeutic intrantiona normalized ratio  + Thrombocytopenia | sICH | + Hematoma volume  + Hematoma expansion  + In-hospital mortality | Imaging  Mortality | + Glucose  +Platelet  + LDL  +HDL  +HbA1c  + Creatinine | Correlation | + History of stroke  + Smoking  + Antiplatelet  + Anticoagulant  + Hypertension  + Diabetes mellitus  + Admission INR  + Admission NIHSS | Not reporting data of biomarker range and number of patients |
| 21 | Chen et al. (2011)  China | Chen YC, Chen CM, Liu JL, Chen ST, Cheng ML, Chiu DT. Oxidative markers in spontaneous intracerebral hemorrhage: leukocyte 8-hydroxy-2'-deoxyguanosine as an independent predictor of the 30-day outcome. *J Neurosurg*. 2011;115(6):1184-1190. doi:10.3171/2011.7.JNS11718 | **Total**  64 patients ICH  114 control | **Inclusion:**  + Patients with ICH  + Hospitalized within 3 days of onset  + Have spontaneous ICH  **Exclusion:**  + SAH  + Traumatic hemorrhage, brain tumor, vascular anomaly  + Abnormal platelet count  + Prolonged prothrombin time  + Prolonged activated partial thromboplastin time | sICH non traumatic, tumor, vascular aetiology | 30-day functional outcome | mRS  good 0-3  poor 4-6 | Not reported in correlation to mRS | Mean (SD) | Not stated | No biomarker data for the reported outcomes |
| 22 | Chen et al. (2016)  Taiwan | Chen CW, Wu EH, Huang J, Chang WT, Ao KH, Cheng TJ, et al. Dynamic evolution of D-Dimer level in cerebrospinal fluid predicts poor outcome in patients with spontaneous intracerebral hemorrhage combined with intraventricular hemorrhage. *J Clin Neurosci* 2016. Doi: 10.1016/j.jocn.2015.10.036 | Total = 43  Survivor = 32  Non-survivor = 11 | **Inclusion:**  + Patients with ICH + IVH + EVD placement  **Exclusion:**  + Intracranial aneurysm  + Intracranial tumor  + Arteriovenous malformation  + Cerebellar hemorrhage  + Trauma  + Coagulopathy | sICH + IVH | 30-day mortality | Mortality | + CSF D-dimer level day 0-7 | Mean (SD) | + Gender  + Age  + Initial GCS  + Graeb score  + ICH location  + IVH severity |  |
| 23 | Chen et al. (2019)  China | Chen S, Chen XC, Lou XH, Qian SQ, Ruan ZW. Determination of serum neutrophil gelatinase-associated lipocalin as a prognostic biomarker of acute spontaneous intracerebral hemorrhage. *Clinica Chimica Acta* 2019. Doi: 10.1016/j.cca.2019.02.009 | Total – 106 | **Inclusion:**  + Spontaneous ICH  + Age > 18  + Available serum NGAL level at admission  **Exclusion:**  + Underlying vascular lesion  + Traumatic hemorrhage  + ICH resulting from venous sinus thrombosis, impaired coagulation, antiplatelet pretreatment, anticoagulant therapy, head trauma, hemorrhagic infarction, tumoral bleedings, acute or chronic infections  + Previous ischemic or hemorrhagic stroke  + Underwent surgical procedure | sICH  Supratentorial + Infratentorial | 90-day Functional outcome | mRS  + Good (<2)  + Bad (>2) | + Blood glucose  + CRP  + WBC  + Platelet  + Serum NGAL | Mean (SD)  >417.6 | + Gender  + Age  + BMI  + Smoking  + Hypertension  + DIabete mellitus  + Hyperlipidemia  + Heart failure  + Chronic kidney disease  + Statin  + Infratentrial hemorrhage  + Subarachnoidal extension  + IVH  + Hemorrhage growth  + GCS  + NIHSS  + ICH  + Hematoma vol | Included cases of chronic severe systemic disease |
| 24 | Chu et al. (2019)  China | Chu H, Huang C, Dong J, Yang X, Xiang J, Dong Q, Tang Y. Lactate dehydrogenase predicts early hematoma expansion and poor outcomes in intracerebral hemorrhage patients. *Transl Stroke Res* 2019. Doi: 10.1007/s12975-019-0686-7 | Proteomic = 52  Derivation cohort = 162  Validation cohort = 366 | **Inclusion:**  + underwent NCCT scan within 6h after ICH onset  + Follow-up 24h  **Exclusion:**  + Secondary ICH (cerebral aneurysm, Moyamoya, arteriovenous malformation, tumor, trauma, hemorrhagic transformation)  + Undergoing surgical evacuation befor NCCT  + Primary IVH  + Baseline ICH vol < 1mL  + Historical mRS > 1 | sICH  Deep  Lobar  Infratentorial | 90-day functional outcome | mRS  + Good (< 3)  + Poor (>3)  mRS per category | + LDH | < 149  150-184  185-219  >220 | Number of patients not mentioned | Not reporting continous scale of biomarkers |
| 25 | De La Ossa et a. (2010) | Pérez de la Ossa N, Sobrino T, Silva Y, et al. Iron-related brain damage in patients with intracerebral hemorrhage. Stroke. 2010;41(4):810-813. doi:10.1161/STROKEAHA.109.570168 | Total = 92  Good = 41  Poorl = 51 | **Inclusion**  + Admitted within 12 h  **Exclusion:**  + Previous disability  + Severe alcohol consumption  + Inflammatory or infectious liver, renal  + Hematologic disease or cancer  + Secondari ICH  + Coma | sICH  Infraten + supratentorial | 90-day functional outcome | mRS  + Good (0-2)  + Poor (3-6) | + Serum glucose  + Platelet count  + Fibrinogen  + Leukocyte  + Ferritin  + IL-6  + TNF-a | Mean (sd) | + Hypertension  + Diabetes  + Smoking  + Alcohl  + NIHSS + GCS scale  + ICH volume  + Edema volume  + ICH growth 72h  + IVH |  |
| 26 | Delgado et al. (2006)  Spain | Delgado P, Alvarez-Sabin J, Abilleira S, Santamarina E, Purroy F, et al. Plasma D-Dimer predicts poor outcome after intracerebral hemorrhage. *Neurology* 2006; 67:94-8. | Total = | + ICH within 24h  **Exclusion:**  + Vascular malformation  + impaired coagulation or oral anticoagulant intake  + head trauma  + Hemorrhagic infarction  + Tumoral bleedings  + underwent a surgical procedure  + History of DVT | sICH supratentorial | + 7-day mortality  + neurologic deterioration 48h | + Mortality  + NIHSS | + fibrinogen  + Prothrombin time  + TTPa+  + Platelet  + glucose  + D-dimer  + Leukocyte | Mean (SD)  Med (IQR) | + Gender  + Age  + Location (lobar)  + NIHSS + GCS  + Blood pressure  + Ich vol  + PE volume  + SAH  + IVH |  |
| 27 | Delgado et al. (2006)b  Spain | Delgago P, Sabin JA, Santamarina E, Molina CA, Quintana M, Montaner J. Plasma S100B level after acute spontaneous intracerebral hemorrhage. *Stroke* 2006; 37:2837-9. Doi: 10.1161/01.STR.0000245085.58807.ad | Total = 78 | **Inclusion:**  + ICH within 24h  **Exclusion:**  + Vascular malformation  + impaired coagulation or oral anticoagulant intake  + head trauma  + Hemorrhagic infarction  + Tumoral bleedings  + underwent a surgical procedure | sICH supratentorial | 3 month functional outcome | mRS  + Good (0-2)  + Bad (3-6) | + Leukocyte count  + Glucose  + S100B | Mean (SD)  Med (IQR) | + Gender  + Location  + NIHSS  +GCS  + Blood pressure  + PE volume  + SAE  + IVE |  |
| 28 | Di Napoli et al. (2011)  Argentina | Di Napoli M, Godoy DA, Campi V, del Valle M, Pinero G, Mirofsky M, et al. C-reactive protein level measurement improves mortality prediction when added to the spontaneous intracerebral hemorrhage score. *Stroke* 2011; 42: 1230-6. Doi: 10.1161/STROKEAHA.110.604983 | Total = 210  Alive = 147  Dead = 63 | **Inclusion:**  + sICh patients admitted to ICU 2005-2009  + Absence of trauma  + Absence of structural lesion  **Exclusion:**  + History of acute or chronic infections  + Evidence of acquired in hospital infection | sICH supratentorial + infratentorial | 30-day mortality | Mortality | + glucose  + WBC  + CRP | Median (IQR) | + Gender  + GCS score  + ICH score  + Arterial hypertension  + Diabetes mellitus  + Alcohol  + Smoking  + Cholesterol  + Antiplatelet  + Blood pressure  + Location  + Hematoma volume  + Midline shift  + Hydrocephalus  + Surgery  + ICH vol (30, >30)  + IVH |  |
| 29 | Di Napoli et al. (2012)  International | Di Napoli M, Godoy DA, Campi V, Masotti L, Smith CJ, Jones ADR, et al. C-reactive protein in intracerebral hemorrhage: Time course, tissue localization, and prognosis. *Neurology* 2012; 79:690-9. | Total = 223 | **Inclusion:**  + sICh patients admitted to ICU 2010 international center  + Absence of trauma  + Absence of structural lesion  **Exclusion:**  + History of acute or chronic infections  + Evidence of acquired in hospital infection | sICH supratentorial and infratentorial | 30-day mortality  30-day poor outcome | + Mortality  + GOS (2-3, death = poor; good (4-5) | + CRP admission + CRP 24h  + CRP 48h  + CRP 72 h  + Blood glucose  + WBC | Median (IQR) | + Blood pressure  + Partial pressure  + Arterial hypertension  + Diabetes mellitus  + Alcohol abuse  + Smoking  + Hypercholesterolemia  + Anticoagulant  + Location  + IVH  + Midline shift  + Hydrocephalus  + Surgery |  |
| 30 | Di Napoli et al. (2013)  International | Di Napoli M, Parry-Jones AR, Smith CJ, Hopkins SJ, Slevin M, Masotti L, et al. C-reactive protein predicts hematoma growth in intracerebral hemorrhage. *Stroke* 2014; 45:59-65. Doi: 10.1161/STROEAHA.113.001721 | CRP < 10 = 213  CRP > 10 = 186 | **Inclusion:**  + patients between January 2009 to Dec 2011  + Age > 18  + Primary or vitamin K antagonist assocated sICH  + Absence of secondary causes : brain tumor, vascular malformation, trauma | sICH supratentorial + infratentorial | 30-day mortality    Early neurological worsening | Mortality | CRP | < 10mg/L  >10ng/L | Nt related to outcome | Biomarkers not reported in mean or median |
| 31 | Ding et al. (2018) | Ding W, Gu Z, Song D, Liu J, Zheng G, Tu C. Development and validation of the hypertensive intracerebral hemorrhage prognosis models. *Medicine* 2018; 97(39): e12446. Doi: 10.1097/MD.00000000000012446 | Good outcome – 239  Poor outcome = 86 | **Inclusion:**  + Admited to ICU between 2012-2016  +Baseline nonenchance CT performed within 6hrs after symptoms onset  **Exclusion:**  + Secondary ICH due to arteriovenous malformation, aneurysm, trauma, tumor, infraction, primary IVH, anticoagulant associated ICH | sICH supratentorial infratentorial | 3-month functional outcome | mRS  Poor = 4-6  Good = 0-3 | + Glucose  + Magnesium  + Cholesterol  + LDL  +D-dimer  + Fibrinogen  + GFR | Mean (SD)  GFR < 60mL/min | + Gender  + Age  + Alcohol  + smoking  + Baseline GCS score  + Hypoxemia  + surgery  + GCS motor response  + Pupillary light reflex  + |  |
| 32 | Dong et al. (2011)  China | Dong XQ, Huang M, Yu WH, Zhang ZY, Zhu Q, Che ZH, et al. Change in plasma copeptin level after acute spontaneous basal gangia hemorrhage. *Peptides* 2011; 32: 253-7. Doi: 10.1016/j.peptides.2010.11.021 | All patients = 86  Survival = 54  Non-survival = 32 | **Inclusion;**  + Patients with spontaneous basa ganglia hemorrhage in 2006-2008  **Exclusion:**  **+** Existing previous neurological disease, head trauma, use of antiplatelet or anticoagulant medication  + Presence of other prior systemic diseases | sICH basal ganglia | 7-day mortality | Mortality | + WBC  + Hb  + Platelet  + Glucose  + Sodium  + Potassium  + Pts  + Thrombin time  + Partial thromboplastin time  + Plasma copeptin  + CRP  + Fibrinogen  + D-Dimer | Median (IQR) | + Gender  + Age  + Hypertension  + Diabetes mellitus  + IVH  + Hematoma volume  + Hydrocephalus  + Hematoma growth  + Rebleeding  + Seizure  + Pneumonia  + DVT  + Mechanincal ventilation  + Admissin time  + Time to surgery  + Surgery | Data duplication with Hu 2006 |
| 33 | Du et al. (2013)  China | Du Q, Yang DB, Shen YF, Yu WH, Zhang ZY, Zhu Q, et a. Plasma leptin level predicts hematoma growth and early neurologica deterioration after acute intracerebral hemorrhage. *Peptides* 2013; 45:35-9. Doi: 10.1016/j.peptides.2013.04.017 | Total = 102  HG presence = 32  HG absence = 70  END presence = 24  END absence = 78 | **Inclusion:**  + Patients with acute spontaneous basal ganglia hemorrhage  + Admitted to ICU within 6h from symptoms onset between April 2010 and October 2012  **Exclusion:**  + Patients under anticoagulant treatment  + GCS <9  + Without follow-up CT scan  + Died < 24h  + Underwent surgical procedure | sICH basal ganglia | Hematoma growth 24h  Early neurological deterioration | + Ct scan  + NIHSS > 4 within 24h | + Glucose  + Hb  + Leukocyte  + Platelet  + Prothrombin time  + Activated partial thromboplastine time  + CRP  + Fibrinogen  + Leptin | Mean (SD) | + BMI  + Gender  + Age  + Antiplatelt  + Statin  + Hypertension  + Diabetes mellitus  + GCS score  + NIHSS score  + Hematoma volume  + IVE  + Plasma sampling time  + blood pressure | Not reporting outcome of interest |
| 34 | Du et al. (2014)  China | Du Q, Yu WH, Dong XQ, Yang DB, Shen YF, Li HW, et al. Plasma 8-iso -prostaglandin F2a concentrations and outcomes after acute intracerebral hemorrhage. *Clinica Chimca Acta* 2014. Doi: 10.1016/j.cca.2014.07.014 | Total = | **Inclusion:**  + Patients with sICh basal ganglia 2010-2013  + Admitted within the first 24h  **Exclusion:**  + Previous stroke  + Severe head trauma  + use of antiplatelet or anticoagulant  + Presence of other prior systemic diseases  + Undergoing surgical procedure  + Unavailable biomarker | sICH basal ganglia | Hematoma growth 24h  Early neurological deterioration  7-day mortality | + Ct scan  + NIHSS > 4 within 24h  + Mortality | Correlation Plasma 8-iso-prostaglandin F2a level  + Glucose  + cRP  + 8-iso-prostaglandin F2a level | Correlation  Meand (SD) for mortality | + Gender  + Age  + Hypertension  + Diabetes mellitus  + NIHSS score  + Hematoma volume  + IVH  + END  + Blood pressure |  |
| 35 | Eldawoody et al. (2016)  Egypt | Eldawoody HAF, Mattar MAB, Mesbah A, Zaher A, Elsherif M. Can brain natriuretic peptide, S100b, and interleukin-6 prognosticate the neurological consequences in Egyptian patients presented with supratentorial intracerebral hemorrhage? *Surg Neurol Int* 2020; 11:460. Doi: 10.25259/SNI_784_2020 | Total = 50  sICH only = 40  ICH + IVH = 10 | **Inclusion:**  + sICH patients | sICH | sICH with IVH vs without IVH | Imaging | + Pro-BNP  + IL-6  + S100B | Med (IQR) | + NIHSS  + GCS  + LOS + BI  + mRS | Not reporting outcome of interest |
| 36 | Elhechmi et al. (2017)  Tunisia | Elhechmi YZ, Hassouna M, Cherif MA, Kaddour RB, Sedghiani I, Jerbi Z. Prognostic value of serum C-Reactive Protein in spontaneous intracerebral hemorrhage: When should we take the sample? *J Stroke Cerebrovas Dis*  2016; 11:129. Doi: 10.1016/j.jstrokecerebrovasdis.2016.11.129 | Total = 91  Dead = 34  Survive= 57 | **Inclusion:**  + Patients admitted for Ich from 2010-2015  +  **Exclusion:**  + Automimune disease  + Neoplasm  + Missing ICH score  + Missing initial and H24 CRP | sICH supratentorial and infratentorial | 30-day mortality | Mortality | + CRP initial  + CRP 24h  + Glycemia  + Creatinine  + WBC  + WBC 24h  + Hb  + Platelet | Med (IQR) | + Hypertension + Stroke  + ICH  + Diabetes mellitus  + Renal failure  + Anticoagulant therapy  + Antiplatelet therapy  + GCS score  + IVH  Infratentorial origin  + Age | Included cases of previous stroke, potential ICH from ischemia stroke |
| 37 | Elkhatib et al. (2019)  Egypt | Elkhatib THM, Shehta N, Bessar AA. Hematoma expansion predictors: Laboratory and radiological risk factors in patients with acute intracerebral hemorrhage: A prospective observational study. *J Stroke Cerebrovas Dis* 2019; 28(8): 2177-86. Doi: 10.1016/j.jstrokecerebrovasdis.2019.04.038 | Total – 134 | **Inclusion:**  + Patients with primary ICH  + Admitted to ICU within 12 hrs of symptoms onset from 2017 to 2018  **Exclusion:**  + secondary causes of ICH including aneurysm, vascular malformation, dissection, tumor, head trauma, infarction, hemorrhagic transformation  + Patients underwent surgical evacuation or craniectomy | sICH supratentorial and infratentorial | + 24hrs hematoma expansion | + Imaging | + RDW  + Platelt  + Leukocyte  + Chlesterol  + Trigyceride  + LDL  +HDL + PT  + INR  + Creatinen  + AST  + ALT  + Albumin  + ESR  +Calcium | Mean (SD) | + Time to initial CT  + Ich location  + ICH shape  + ICH density  + Ventricular extension  + CTA spot sign | Not reporting outcome of interest |
| 38 | Fan et al. (2018)  China | Fan Z, Hao L, Chuanyuan T, Jun Z, Xin H, Sen L, et al. Neutrophil and platelet to lymphocyte ratios in associating with blood glucose admission predict the functional outcomes of patients with primary brainstem hemorrhage. *World Neurosurg* 2018; 116:e100-7. Doi: 10.1016/j.wneu.2018.04.098 | Total = 225  Favorable = 113  Poor = 112 | **Inclusion:**  + patients with primary brain stem hemorrhage 2012-2016  + Proper PHB symptoms within 24h  + All diagnostic or laboratory within 24h  + Age > 18years  **Exclusion:**  + Acute or chronic infection or postinfection within 2 weeks  + History of neoplasm, uremia, liver cirrhosis, autoimmune disase, chornic heart disease, severe renal dysfunction, chronic lung disease  + History of using immunosuppressive drugs or anticoagulant  + Trauma, brain tumor, aneurysm and severe central nerve system  + Apoleptic stroke within 6 mo  + previous surgical treatment | sICH brain stem | 90-day Functional outcome | GOS  + Poor = <3  + Good > 3 | + Hb  + Platelet  + WBC  + Neutrophil  + Leukocyte  + Monocyte  + NLR + PLR  + Glucose | Median (IQR) | + Gender  + Age  + Blood pressure  + Tim from onset to admission  + GCS score  + Hematoma size  + SAH  + IVH  + Hydrocephalus |  |
| 39 | Fang et al. (2005)  Taiwan | Fang HY, Ko WJ, Lin CY. Plasma interleukin 11 levels correlate with outcome of spontaneous intracerebral hemorrhage. *Surg Neurol* 2005; 64:511-8. Doi: 10.1016/j.surneu.2005.03.018 | Total = 43  Survival = 33  Nonsurvival = 10 | **Inclusion:**  + sICH patients admitted to surgical ICU between 2002-2003  + ICH noted on brain CT scan on presentation to the emergency department  + Patient age between 40-85  + time between symptom onset and admission < 6hr  + No other previous systemic diseases including uremia, liver cirrhosis, malignancy, chronic heart or lung disease  **Exclusion:**  + history of head trauma or previous troke  + use of antiplatelet or anticoagulant medication  + Arteriovenous malformations of the brain or ruptured cerebral aneurysm | sICH supratentorial + infratentorial | Discharge mortality | Mortality | + WBC  + Hb  + Platelet  + Glucose  + Sodium  + Potassium | Mean (SD) | + Hypertension  + Diabetes mellitus  + Smoking  + Alcoholism  + GCS score  + ICH Score  + Hematoma volume  + Edema score  + IVH  + Location | Not reporting defined time for outcome assessment |
| 40 | Fang et al. (2007)  Taiwan | Fang HY, Ko WJ, Lin CY. Inducible heat shock protein 70, interleukin-18, and tumor necrosis factor alpha correlate with outcomes in spontaneous intracerebral hemorrhage. *J clin Neurosci* 2007; 14:435-41. Doi: 10.1016/j.jocn.2005.12.022 | Total = 43  Survivor = 33  Non-survivor = 10 | **Inclusion:**  + ICH patients between 2002-2003  ++ ICH noted on brain CT scan on presentation to the emergency department  + Patient age between 40-85  + time between symptom onset and admission < 6hr  + No other previous systemic diseases including uremia, liver cirrhosis, malignancy, chronic heart or lung disease  **Exclusion:**  + history of head trauma or previous troke  + use of antiplatelet or anticoagulant medication  + Arteriovenous malformations of the brain or ruptured cerebral aneurysm | sICH supratentorial infratentorial | Discharge mortality | Mortality | + TNF-a  + IL-18  + HSP-70  + WBC  + Hb  + Platelet  + Glucose  + Sodium  + Potassium | Bar plot | + Hypertension  + Diabetes mellitus  + Smoking  + Alcoholism  + GCS score  + ICH Score  + Hematoma volume  + Edema score  + IVH  + Location | Not reporting defined time for outcome assessment |
| 41 | Ferrete-Araujo et al. (2019) | Ferrete-Araujo AM Rodriguez AR, Egea-Guerrero JJ, Vilches-Arenas A, Godoy DA, Murillo-Cabezas F. Brain injury biomarker behavior in spontaneous intracerebral hemorrhage. *Word Neurosurg* 2019. Doi: 10.1016/j.wneu.2019.08.090 | Total = 36 | **Inclusion:**  + Age > 14 years  + sICH clinically diagnosed, confirmed by CT scan  + primary IVH  + Hospital admission within 48 hrs  **Exclusion:**  **+ Pregnancy**  **+** ICH secondary to aneurysm rupture, vascular malformation, tumor, trauma, cerebral herniation, moyamoya | sICH supratentorial dan infratentorial | Admission Neurological severity  Admission Functional outcome | + GCS  + APACHE II  + ICH  + MICH  + Graeb  + Ct volume  + GOS  + mRS | + S100b  + NSE | Correlation | Not stated | Not reporting data of biomarker range and number of patients |
| 42 | Foerch et al. (2006)  Germany | Foerch C, Curdt I, Yan B, Dvorak F, Hermans M, Berkefeld J, et al. Serum glial fibrillary acidic protein as a biomarker for intracerebral hemorrhage in patients with acute stroke. *J neurol Neurosurg Psychiatry* 2006; 77:181-4. Doi: 10.1136/jnnp.2005.074823 |  | **Inclusion:**  onset of an acute and persisting focal eurological deficit caused by ICH or cerebral ischaemia and admission of the patient to our centre within six hours after symptom onset.  **Exclusion:** Patients with persistent negative findings on repeated brain imaging were excluded. Further exclusion criteria were  previous brain injury, a history of intracerebral haemorrhage or cerebral ischaemia, or any other pre-existing central nervous system disease. | Hemoragghic and ischemia | Hematoma volume  Biomarker dynamics | Imaging | GFAP | 2.9ng/L cut-off  Dynamic  Correlation | Not stated | Not reporting outcome of interest |
| 43 | Fonseca et al. (2020)  Belgia | Fonseca S, Cosata F, Seabra M, Dias R, Soares A, Dias c, et al. Systemic inflammation status at admission affects the outcome of intracerebral hemorrhage by increasing erimhematomal edema but not the hematoma growth. *Acta neurologica Belgica* 2020. Doi; 10.1007/s13760-019—1269-2 | Total = 135 | **Inclusion:**  + ICH patients, CT 24h after onset  **Exclusion:**  + Trauma  + Neoplastic or vascular lesion  + Hemorrhagic transformation of a cerebral infarct  + Massive hemorrhage with very poor prognosis leading to the withdrawal of usual ICU management  + Unavailable CT | sICH  Lobar, deep, infratentorial | + 30-day mortality  + 90- day functional outcome  + Hematoma expansion  + Infection 48h  + Significant midline shift | +Mortality  + mRS  Good (0-2)  Bad (3-6)  + Volume > 6mL or 33%  + Shift >2.5mm | + NRL  + PLR + Neutrophilia  + Lymphocyte  + CRP  +Glucose | Med (IQR) | Not stated |  |
| 44 | Gareev et al. (2019)  China | Gareev I, Yang G, Sun J, Beylerli O, Chen X, Zhang D, et al. Circulating microRNAs as potential noninvasive biomarkers of spontaneous intracerebral hemorrhage. *World Neurosurg* 2019. Doi: 10.1016/j.wneu.2019.09.016 | Total = 156  Patient ICH: 106  Healthy: 50 | **Inclusion:**  + ICH patients 2016-2017 admitted to Medical university in China  **Exclusion:**  + Cardiovascular disease  + Immune disease  + Previous surgery  + Injuries  +Organ failure  + Tumors  + Secondary ICH  + primary IVH  + infection | sICH supratentorial and infratentorial | + Relative biomarker expression | qPCR | + miR-145  + miR-181b  + miR-223  + miR-155 | Expression | None | Not reporting outcome of interest |
| 45 | Garrett et al. (2009)  USA | Garrett MC, Komotar RJ, Starke RM, Doshi D, Otten ML, Connolly ES. Elevated troponin levels are predictive of mortality in surgical intracerebral hemorrhage patients. *Neurocrit Care* 2010; 12:199-203. Doi: 10.1007/s12028-009-9245-5 | Dead = 20  Alive = 26  Rehab = 33  Home = 21 | **Inclusion:**  Patients were admitted to the NICU either from our emergency department or at the discretion of the admitting neurologist or neurosurgeon as transfers from outside hospitals. | Traumatic and non-traumatic hemorrhagic stroke | In-hospital mortality | Mortality | Troponin | Correlation | +Etiology  + Location + IVH  + SAH + Midline shift  +hydrocephalus | Biomarkers not reported in mean or range median |
| 46 | Garton et al. (2017)  USA | Garton ALA, Gupta VP, Christophe BR, Connolly ES. Biomarkers of functional outcome in intracerebral hemorrhage: interplay between clinical metrics, CD163, and ferritin. *J Stroke Cerebrovas Dis* 2017; 26(8): 1712-2. Doi: 10.1016/j.jstrokecerebrovasdis.017.03.035 | Total =41 | **Inclusion:**  + Nontraumatic Ich in university medical center 2009-2019  + Serum sample collectod on both day 1 and 7  + Serum samples were <5yrs old  + Follow-up data at 3 and 12 montsh | Nontraumatic ICH | 3-month and 12-month Functional outcome | mRS (Good = 0-3) | +CD163  + Ferritin  + Hepcidin | Mean (SD) | + Age  + Gender  + Hematoma volume  + Initial APACHE II  + Initial GCS  + Initial ICH  + Initial NIHSS | Unstated aetiologies May included secondary ICH causes. |
| 47 | Gerner et al. (2018)  Germany | Gerner ST, Auerbeck K, Sprügel MI, et al. Peak Troponin I Levels Are Associated with Functional Outcome in Intracerebral Hemorrhage. Cerebrovasc Dis. 2018;46(1-2):72-81. doi:10.1159/000492395 | **Total**  745 patients | **Inclusion:**  + Non traumatic ICH  **Exclusion:**  + Patients with secondary ICH due to hemorrhagic transformation of secondary ICH, tumor, aneurysm, AV malformation | Non traumatic ICH | + Functional outcome 3-mo  + Functional outcome 12-mo | mRS score  + 0-3 good  + 4-6 poor | Troponin | Cut off  <0.040, 0.040 – 0.500, >0.500 | Not stated | Not reporting data of biomarker range |
| 48 | Giede-Jeppe et al. (2017)  Germany | Giede-Jepe A, Bobinger T, Gerner ST, Sembil JA, Sprugel MI, Beuscher VD, et a. Neutrophil-to-Lymphocyte ration is an independent predictor ofor in-hospital mortality in spontaneous intracerebral hemorrhage. *Cerebrovas Dis* 2017; 44:26-34. Doi: 10.1158/000368996 | Total = 855 | **Inclusion**  + Spontaneous Ich patients admitted between 2006-2014  **Exclusion:**  + Secondary ICH etiologies: trauma, tumor, arteriovenous malformation, central venous thrombosis, SAH, thrombolysis  + Patients receiving permanent immunomodulatory treatments  + Patients with hematological, autoimmune, infectious disease | sICH supratentorial infratentorial | 3-month mortality  3-month functional outcome | Mortality  mRS  -Good (0-3)  -Bad (4-6) | NLR on admission | Cut-off 2.606  Cut-off  8.508 | Not mentioned | Not reporting data of biomarker range and number of patients |
| 49 | Giede-Jeppe et al. (2016)  Germany | Giede-Jeppe A, Bobinger T, Gerner ST, Madzar D, Sembil J, Lucking H, et al. Lymphocytopenia is an independent predictor of unfavorable functional outcome in spontaneous intraferebral hemorrhage. *Stroke* 2016; 47:1239-1246. Doi: 10.1161/STROKEAHA.116.013003 |  | **Inclusion**  + Spontaneous Ich patients admitted between 2006-2014  **Exclusion:**  + Secondary ICH etiologies: trauma, tumor, arteriovenous malformation, central venous thrombosis, SAH, thrombolysis  + Patients receiving permanent immunomodulatory treatments  + Patients with hematological, autoimmune, infectious disease | sICH supratentorial infratentorial | 3-month functional outcome  In-hospital mortality | mRS  -Good (0-3)  -Bad (4-6) | Lymphocytopenia on admission  Lymphocytopenia on day 5 | Cut-off 1x10^10^/L | Not mentioned | Not reporting data of biomarker range and number of patients |
| 50 | Gong et al. (2020)  China | Gong X, Lu Z, Feng X, Yu C, Xue M, Yu L, et al. Elevated neutrophil-to-lymphocyte ratio predicts depression after intracerebral hemorrhage. *Neuropsychiatrk Dis Treat* 2020; 16:2153-9. | Total 372 | **Inclusion:**  (1) were  diagnosed with ICH verifed by CT scans within 24h from symptom onset; (2) aged ≥18 years  **Exclusion:**  (1) had secondary hemorrhage as  a result of tumor, trauma, vascular malformation, aneurysm, hemorrhagic transformation of cerebral infarct and blood coagulation abnormalities; (2) had concurrent disease that might affect the value of NLR, such as active  infection (fever, cough, or diarrhea), chronic inﬂammatory disease or malignancy; (3) had severe cognitive impairment, a history of major depression or other psychiatric disorders; (4) with severe aphasia so that could  not complete the psychological measurement; (5) had  severe heart, renal or liver diseases | sICH supratentorial infratentorial | Depression after ICH | HAMD >7 | + WBC  + ANC  +ALC  + NLR | Median (IQR) | +Age  +Gender  + Married  + HTN  + DM  + coronary heart disease  +Atrial fibrillation  + Prior stroke  + Smoking  + Drinking  + hematoma volume  + Lcation  + NIHSS  + GCS  +mRS  + BI  +MMSE | Not reporting outcome of interest |
| 51 | Goya et al. (2014)  Japan | Goya Y, Shibazaki K, Sakai K, Aoki J, Uemura J, Saji N, et al. Brain natriuretic peptide upon admission as a biological marker of short-term mortality after intracerebral hemorrhage. *Eur Neurol* 2014; 71:203-7. Doi: 10.1159/000356198 | Total 271  Survive 223  Dead 48 | **Inclusion:**  Patients admitted to stroke center within 24h of ICH onset 2007-2011  **Exclusion:**  ICH related to aneurysms, vascular malformation, dissection, tumor, hemorrhagic transformation of ischemic stroke and traumatic ICH were excluded | sICH supratentorial infratentorial | 30-day mortality | Mortality | +Hb  + HBA1c  + Glucose  + CRP  + BNP | Mean (SD) | + Age  + Gender  + Prior CAD  + Prior ICH  + Prior ischemic stroke  + HTN  + DM  + Hyperlipidemia  + AF  + Antiplatelet  + Blood pressure |  |
| 52 | Goyal et al. (2018)  USA | Goyal N, Tsivgoulis G, Malhotra K, Houck AL, Khorchid YM, Pandhi A, et al. Serum magnesium levels and outcomes in patients with acute spontaneous intracerebral hemorrhage. *J Am Heart Assoc*  2018; 7:e008698. Doi: 10.1161/JAHA.118.008698 | 672 | **Inclusion:**  + Acute sICH <24h  + Age 18 years or more  + Available admission Mg level  **Exclusion:**  + Nonspontaneous cause of ICH including trauma, metastatic hemorrhagic cerebral lesion, venous sinus thrombosis, vascular lesion  + Anticoagulation/ coagulopathy  + Thrombocytopenia | sICH supratentorial and infratentorial | Discharge functional outcome  In hospital mortality | mRS  +favorable (0-1)  + Independence (0-2) | Serum Mg | Mean (SD) | Not related | Not reporting defined time for outcome assessment |
| 53 | Graham et al. (2012)  Hong Kong | Graham CA, Chan RWY, Chan DYS, Chan CPY, Wong LKS, Rainer TH. Matrix metalloproteinase 9 mRNA: an early prognostic amerker for patients with acutes stroke. *Clin Biochem* 2011. Doi: 10.1016/j.clinbiochem.2011.12.006 | Total = 126 | **Inclusion:**  + Age 18 and older  + Presenting to Emergency Department of Prince of Wales Hospita, Hongkong within 24h of onset with either hemorrhagic or ischemic stroke | Hemorrhagic and ischemic stroke cases | 6-month functional outcome  6-mo mortality | mRS  + Good (0-2)  + Bad (3-5)  + Died (6) | MMP-9 mRNA | Fold change expression | No data for other cofounding | Not reporting standard measurement for biomarker |
| 54 | Gu et al. (2013)  China | Gu SJ, Xuan HF, Lu M, Chen XZ, Dong WF, Yang XF, et al. Admission plasma visfatin level strongly correlates with hematoma growth and early neurologic deterioration in patients with acute spontaneous basal ganglia hemorrhage. *Clin Chim Act* 2013; 425:85-9. Doi: 10.1016/j.cca.2013.07.025 | Total – 85 | **Inclusion:** + sICH basal ganglia withi 6h  **Exclusion:**  + Patients with inflammatory diseases  + Infectious disease  + Renal or liver problems  + under anticoagulant treatment  + GCS <9  + Without a follo-up CT  + Dying <24h  + Undergoing surgical procedure | sICH basal ganglia | Hematoma growth  Early neurologic deterioration | +24h CT scan  24h NIHSS increase of >4 points | + Hb  + Leukocyte  + Platelet  + Prothrombin time  + Partial thromboplastin time  + CRP  + Fibrinogen  + Visfatin  + Glucse | Mean (SD) | + Gender  + BMI  + Antiplatelet  + Statin  + HTN  + DM  + GCS  + NIHSS  + Volume  + IVH  + Blood pressure | Not reporting outcome of interest |
| 55 | Gupta et al. (2012)  India | Gupta DK, Atam V, Garg RK. Prognostic value of routine hamatological and biochemical parameters on 30 day fatality in patients with acute hypertensive intracerebral hemorrhage. *Neurol Asia* 2012; 17(1): 13-20. | Total = 111 | **Inclusion:**  + Primary sICH patients 2008 to 2009  + Hypertensive ICH presented within 72h  **Exclusion:**  + Previous history of stroke  + Alcoholic  + Having episodes of jaundice | Hypertensive sICH | Discharge mortality | Mortality | + Hematocrit  + Leukocyte  + Glucose  + Sodium  + Potassium  + Urea  + Creatinine  + Bilirubin  + AST  + ALT + Alanin transaminase  + ALP  + Albumin  + Erythrocyte sedimentation | Mean (SD) | + Blood pressure  + GCS score | Not reporting defined time for outcome assessment |
| 56 | Gupta et al. (2019)  India | Gupta M, Verma R, Parihar A, Garg RK, Singh MK, Malhotra HS. Perihematomal edema as predictor of outcome in spontaneous intracerebral hemorrhage. *J Neuroscie Rural Pract* 2015; 5(1): 48-54. Doi: 10.4103/0976-3147.127873 | Total = | **Inclusion**  + Newly diagnosed sICH imaged within 24-72h rom onset  **Exclusion:**  Intraventricular extension, infratentorial location,  history of anticoagulation, bleeding disorders,  hemorrhage related to intracranial neoplasm, presence of arterio‑venous (AV) malformations, critically ill at admission, presence of end organ damage (congestive cardiac failure (CCF), respiratory failure, and others), and, need for cerebrospinal fluid shunting procedure or cranionotomy or surgical evacuation of intracerebral hematoma. | sICH supratentorial infratentorial | 3-month functional outcome | mRS  + Good (0-2)  + Bad (3-6) | + PT  + aPTT  + Platelet  + Glucose | Mean (SD) | + Age  + Gender  + HTN  + DM  + Alcohol  + Smoking  + Headache  + Vomiting  + seizure  + GCS mean  + Blood pressure  + Admission mRS |  |
| 57 | Halstead et al. (2020)  USA | Halstead MR, Mould WA, Sheth KN, Rosand J, Thompson R, et al. Haptoglobin is associated with increased early perihematomal edema progression in spontaneous intracranial hemorrhage. *Int J Stroke* 2020; 15(8): 899-908. Doi: 10.1177/1747493020912602 | Total - 166 | **Inclusion:**  + Patients with acute sICH 2010-2017  + Patients from MISTIE III trial  + Not exposed to surgical intervention  + Age >18 year  + Presence of initial and follow-up CT within 96h | sICH | +Haptogobin genotype  + Discharge mortality  + Functional recovery on discharge | +sequencing  + Mortality  + mRS (>3) | Haptoglobin | Hp1-1  HP1-2  Hp2-2 |  | Not reporting outcome of interest |
| 58 | Han et al. (2018)  China | Han M, Ding S, Zhang Y, Lin Z, Li K. Serum copper homeostasis in hypertensive intracerebral hemorrhage and its clinical significance. *Biol trace Element Res* 2018. Doi: 10.1007/s12011-017-1227-4 | Healthy (32)  Hypertension (12)  ICH (85) | **Exclusion:**  + Amyloid angiopathy  + Brain tumor  +Aneurysm  + Arterivenous malformation  + Previous disability  + severe alcohol consumption + Cancer  + Immunodeficiency syndrome  + Chronic immune disorders | sICH | Functional outcome at discharge  Discharge mortality | mRS  -Good (0-2)  -Bad (3-6)  Mortaity | + Leukocyte  + Glucose  +CRP | Median (IQR) | + Age  + Gender  + Blood pressure  + GCS  + ICH | Not reporting defined time for outcome assessment |
| 59 | Hays et al. (2006)  USA | Hays A, Diringer MN. Elevated troponin levels are associated with higher mortality following intracerebral hemorrhage. *Neurology* 2006; 66:1330-4 | Total = | **Inclusion:**  + patients admitted to NNICU 2001-2005  + CT performed within 24h of presentation  **Excludion:**  + Head trauma within 2 weeks  + History of ischemic stroke  +Treatment with thrombolytic agents within 2 weeks  + History of myocardia infarction  + Primary IVH  + SAH  + Tumor  + Vascular malformation  + Bacterial meningitis  + Surgical complication  + Reversible vascular malformation | sICH supratentorial infratentorial | In hospital mortality | Mortality | Cardiac troponin | <0.1  -0.1-0.4  >0.4 | + Age  +Ethnicity  + HTN  + DM  + Atrial fibrillation  + prior ICH  +CAD  + Blood pressure  + GCS  + Management  + Location  + IVH  + MLS  + SAH | Not reporting defined time for outcome assessment |
| 60 | He et al. (2018)  China | He D, Zhang Y, Zhang B, Jian W, Deng X, Yang Y, et al. Serum procalcitonin levels are associated with clinical outcome in intracerebral hemorrhage. *Cell Mol Neurobil* 2018; 38:727-33. | Total = 251 | **Inclusion:**  + ICH patients diagnosed on CT evaluated within 24h of onset from 2015-2016  **Exclusion:**  + Age <18 years  + Pre-onset mRS >3  + hemorrhagic conversion of acute brain infarction, trauma, hematologial malignancy, hemophilia, intracranial tumor, aneurysm, moyamoya disease, vascular malformation  + Active or chronic inflammatory disorder  + Sever renal disease hepatic disease | sICH  Lobar  Basal ganglia  Brainstem  Cerebellar | 3-month functional outcome  3-month mortality | mRS  +Good (0-2)  +Bad (3-6)  Mortality | +Leukocyte  + Neutrophil  + HsCRP  + Fibrinogen  + FBG  +Procalcitonin | Mean (SD) | + Age  + Gender  + HTN  + DM  + Hyperlipidemia  + Smoking  Stroke  + CHD  + Blood pressure  + BMI  + Pneumonia  + ICH volume  + IVH  + Perihematomal edema  + Location |  |
| 61 | Hedge et al. (2020)  India | Hedge A, Menon G, Kumar V, Prasad GL, Kongwad LI, Nair R, et al. Clinical profile and predictors of outcome in spontaneous intracerebral hemorrhage from a tertiary care centre in South India. *Stroke Res Treat* 2020. Doi: 10.1155/2020/2192709 | Total = 905  Alive = 619  Dead = 266 | **Inclusion:** + Age >18  + Evidence of sICH  **Exclusion:**  + post-traumatic hematoma  + intracranial space lesion  + Hemorrhagic transformation  + Vascular malformation  +Aneurysm | sICH infratentorial supratentorial | 3-month functional outcome  3-month mortality | mRS  -Good (0-3)  -bad (4-5)  Mortality | Blood glucose | Mean (SD) | +HTN  + DM  + Smoking  + Alcohol  + Antipatelet  + Heart rate  + Blood pressure  + GCS  + ICH score  + Volume  + Location  + IVH  + Hydrocephalus  + Hematoma growth  + Surgery |  |
| 62 | Herdatono et al. (2018)  Indnesia | Herdatono TK, Retnaningsih, Husni A. Hubungan kadar glial fibrillary acidid protein (GFAP) serum dengan luaran klinis neurologis pasien stroke hemoragik. *Neurona* 2018; 36(1):27-35 | Total = | **Inclusion:**  + Hemorrhagic stroke proven with CT scan result  + Primary sICH  + Onset <72 h  **Exclusion**  + Undergone surgeries  + History of autoimmune diseases  + Trauma in last 3 months  + history of brain tumour  + History of dementia Alzheimer  + Infection before onset  + ICH score >3 | sICH supratentorial and infratentorial | 30-day functional outcome  7-day neurological state | mRS  -Good (0-2)  -Bad (3-6)  NIHSS  -Good (score difference d3 and d7 >4) | GFAP serum | Mean (SD) | + gender  +Age  +BMI  + Hypertension  + DM  + GCS  + Location  + Volume  + IVH | Not written in English |
| 63 | Hu et al. (2010)  China | Hu YY, Dong XQ, Yu WH, Zhang ZY. Change in plasma S100B level after acute spontaneous basal ganglia hemorrhage. *Shock* 2010; 33(2): 134-40. Doi: 10.1097/SHK.0b013e3181ad5c88 | Total = 86  Survival (54)  Nonsurvival (32) | **Inclusion:**  + Patients with sICh basal ganglia 2006=2008  **Exclusion;**  + Existing previous neurological disease  + Head trauma  + use of antiplatelet or anticoagulant medication + Presence of previous systemic disease | sICH basal ganglia | 7-day mortality | Mortality | + WBC  + Hb  + Platelet  + Glucose  + Sodium  + Potassium + Prothrombin time  + Thrombin  + Partial thromboplastine time  + S100B  + CRP  +Fibrinogen  + D-dimer | Mean (SD) | + Blood pressure  + Heart rate  + Temperature  + Gender  + Age  + Hypertension  + Diabetes mellitus  + GCS score  + IVH  + ICH volume  + Hydrocephalus  + hematoma growth  + rebleeding  + Seizure  + Pneumoneia  + DVT  + Mechanical ventilation  + Admission time  + Surgery |  |
| 64 | Hu et al. (2014)  China | Hu X, Fang Y, Ye F, Lin S, Li H, You C, Liu M. Effects of plasma D-dimer levels on early mortality and long-term functional outcome after spontaneous intracerebral hemorrhage. *J clin Neurosci* 2014. Doi: 10.1016/j.jocn.2013.11.030. | Total = | **Inclusion:** + Adult patients  + Admitted with sICH within 24 h of onset between 2010-2011  **Exclusion:**  + Trauma  + Arteriovenous malformation  +Aneurysm  + Brain tumor  + Hemorrhagic transformation  + Coagulopathy  + Renal or liver disease | sICH supratentorial + infratentorial | 7-day mortality  3-month functional outcome | Mortality  mRS score  -Good (0-2)  -Badn (3-6) | + WBC  + Glucose  + Platelet  + Prothrombin time + APTT  + Fibrinogen  + D-dimer | Median (IQR) | + Age  + Gender  + Hypertension  + Diabetes mellitus  + Alcohol  + Smoking  + Location  + Midline shift  + SAH  + IVH  + Hematoma volume  + IVH score  + GCS score  + Surgery |  |
| 65 | Hu et al. (2019)  China | Hu Zj, Wang XC, Zhu LC, Yao Ym, Chen TT, Xu J, et al. Circulating Klotho is linked to prognosis of acute intracerebral hemorrhage. *Clinica Chimica Acta* 2019; 497: 114-9. Doi: 10.1016/j.cca.2019.07.023 | Total = 96  Patient: 96  Control healthy: 96 | **Inclusion:**  + First ever ICH witin 24h between 2014=2017  **Exclusion:**  + Age <18 years  + Bleedingfrom underlying vascular lesions, traumatic force, venous sinous thrombosis, coagulation, infarction, tumors  + Undergoing surgical procedure  + Coexsisting autoimmune disease  + Sever infection  + Pregnancy  + Malignancy | sICH supratentorial infratentorial | 90-day functional outcome | mRS  -Good (0-2)  -Bad (3-6) | + Klotho  + Serum D-dimer  + CRP  + WBC  + Platelet | Klotho:  <224  229-298  299-379  >393  Other:  Mean (SD) | + Blood pressure  + Age  + BMI  + smoking  + Alcohol  + HTN + DM  + Hyperlipidemia  + Heart failure  + Coronary artery disease  + Chronic kidney disease  + Statin  + GCS  +NIHSS  +ICH score  + Hematoma volume  + Location + IVH  + SAH | Included cases of chronic sever systemic disease |
| 66 | Huang et al. (2010)  China | Huang M, Dong XQ, Hu YY, Yu WH, Zhang ZY. High S100B levels in cerebrospinal fluid and peripheral blood of patients with acute basal ganglial hemorrhage are associated with poor outcome. *World J Emerg Med* 2010; 1(1): 22-31. | Total = 38  Survival = 23  Non-survival = 15 | **Inclusion:** + sICH patients 2007-2008  + ICH on CT scan  + Age between 40-80 years  + Admission time <6h  + No other previous systemic diseases  + Need and acceptance of surgical therapy  + Time to surgery within 12 hrs  **Exclusion:**  + Head trauma  + Previous stroke  + Use of antiplatelet or anticoagulant medication  + History of arteriouvenous malformation  + History of ruptured cerebral aneurysm | sICH basal ganglia | 7-day mortality | Mortality | + WBC  + Platelet  + Glucose  + Sodium  + Potassium  + Prothrombin time  + Thrombin time  + Partial thromboplastin time  + S100B plasma  + S100B CSF | Mean (SD) | +Blood pressure  + Heart rate  + Temperature  + respiratory rate  + Gender  + Age  + Hypertension  + Diabetes mellitus  + GCS score  + IVH  + Hydrocephalus  + Hemorrhage growth  + Rebleeding  + Seizure  + Pneumonia  + DVT + Mechanical ventilation  + Admission time  + Time to surgery  + Surgery |  |
| 67 | Huang et al. (2020)  China | Huang H, Huang G, Gu J, Chen K, Huang Y, Xu H. Relationship of serum uric acid to hematoma volume and prognosis in patients with acute supratentorial intracerebral hemorrhage. *World Neurosurg* 2020. Doi: 10.1016/j.wneu.2020.08.018. In Press | Total = 325 | **Inclusion:**  + Acute supratentorial ICH 2016-2017  + Hospitalized within 12h after onset symptoms  + CT examination completed within 24h  + Age > 18years  + Lab examination performed at admission  **Exclusion:**  + ICH caused by head trauma  + Treatment with antiplatelet, anticoagulants, UA lowering drugs, steroid, immunosuppressive agents  + CT image too blurred  + No serum UA  + History of systemic disease | sICH supratentorial | 30-day functional outcome | mRS  -Good (0-3)  -Bad (4-6) | +Leukocyte  + Neutrophil  + Lymphocyte  + Monocyte  + Platelet  + Uric acid  + Glucose  + Creatinine  + prothrombin time | Med (IQR) | + Gender  + Age  + smoking  + Alcohol  + Hypertension  +Diabetes mellitus  + Blood pressure  + surgery | Not excluding secondary ICH caused by |
| 68 | Hviid et al. (2019) | Hviid CVB, Gyldenholm T, Lauridsen SV, Hjort N, Hvas AM, Parkner T. Plasma neurofilament light chain is associated with mortality after spontaneous intracerebral hemorrhage. *Clin Chem Lab Med*  2019. Doi: 10.1515/cclm-2019-0532 | Total = 37  Non-survivor 7  Survivor = 30  Control = 108 | **Inclusion:**  + Patients admitted to ICH of Departments of Neurosurgery and Neurology 2014-2016  + Age > 18 years  + CT within 6h  **Exclusion:** + Pre-existing bleeding disorder  + Treatment with any antithrombotic drugs  + On going antibiotics  + Pregnancy  + Active cancer or chemotherapeutic treatment within last 3 months  + Liver cirrhosis  + Ischemic or hemorrhagic cerebral infarction +Structural cause of ICH | sICH supratentorial and infratentorial | 30-day mortality | Mortality | Plasma neurofilament light chain  -Admission  -24h | Dot plot | Admission hematoma volume | Biomarkers not reported elsewhere |
| 69 | Inoue et al. (2013) | Inoue Y, Miyashita F, Toyoda K, Minematsu K. Low serum calcium levels contribute to larger hematoma volume in acute intracerebral hemorrhage. *Stroke* 2013; 44:2004-6. Doi: 10.1161/STROKEAHA.113.001187/-/DCI | Total = 273 | **Inclusion:**  + Nontraumatic ICH admitted within 24h from onset  + | Nontraumatic ICH | 30-day functional outcome  Admission neurological state  Hematoma volume | mRS:  -Good (0-2)  -Bad (3-6)  NIHSS 24h  Imaging | Calcium level | Quartile | Not stated | Unstated aetiologies May included secondary ICH causes |
| 70 | James et al. (2009)  USA | James ML, Blessing R, Phillips-bute BG, Bennet E, Laskowitz DT. S100B and brain natriuretic peptide predict functional neurological outcome after intracerebral hemorrhage. *Biomarkers*  2009; 14(6): 388-94. Doi: 10.1080/13547500903015784 | Total: 28 | **Inclusion:**  Supratentorial ICH 1 January 2000 and 31 December  2003  **Exclusion:**  Age <18  Confirmed pregnancy  Known or suspected barin tumor  Known or suspected CNS vascular malformation  Presence of subarachnoid blood  Masive head trauma  Time of presentation >24h  Multiple organ dysfunction at time of admission | Supratentorial ICH | Functional toucome | Barthe Index  mRS | S100B  BNP | Odds ratio,  Regression | Data not shown | Not reporting data of biomarker range and number of patients |
| 71 | Ji et al. (2017)  China | Ji WJ, Chou XM, Wu GQ, Shen YF, Yang XG, Wang ZF, et al. Association between serum periostin concentrations and outcome after acute spontaneous intracerebral hemorrhage. *Clin Chim Act*2017; 474:23-7. Doi: 10.1016/j.cca.2017.09.002 | Patient: 118  Control: | **Inclusion:**  + Patients period of Jan 2013-Apr 2016  + Basal ganglia sICH  + Presented 24h  **Exclusion:**  + Surgical procedure  + Prior ICH  + Ischemic stroke  + Severe head trauma  + Use of antiplatelet or anticoagulant  + Presence of other systemic disease  + Malignancy | Basal ganglisa sICH | 6-month functional outcome | mRS  + Good (0-2)  +Bad (3-6) | + Blood glucose  + CRP  + Periostin | Med (IQR) | + Age  + Gender  + Hypertension  + DM  + Hypercholesterlemia  + Location  + NIHSS score  + Hematoma volume  + IVH  + Early deterioration  + Blood pressure |  |
| 72 | Jiang et al. (2014)  China | Jiang YX, Zhang GH, Wang ZM, Yang H. Serum YKL-40 levels as a prognostic factor in patients with intracerebral hemorrhage. Clin Biochem. 2014;47(18):302-306. doi:10.1016/j.clinbiochem.2014.09.015 |  | **Inclusion:**  + sICH basal ganglia  + Admitted within 6h January 2010-March 2013  **Exclusion:**  + Use of antiplatelet or anticoagulant  + Previous neurological disase  + Head trauma  + Presence of systemic diseases  + Malignancy  + Infection  + Patients undergoing surgical procedures | sICh basal ganglia | 3-mo mortality  3-mo functional outcome | Mortality  mRS  + Good (0-2)  + Bad (3-6) | + CRP  + D-dimer  + WBC  + Hb  + Platelet  + Prothrombin time  + Thrombin tinme  + pTP time  + Fibrinogen  + YKL-40 | Mean (SD) | + Age  + Gender  + HT  + DM  + NIHSS  + Volume  + IVH  + Hematoma growth  + Blood pressure |  |
| 73 | Juntilla et al. (2017)  Finland | Junttila E, Koskenkari J, Ala-Kokko T. Hypophoshatemia after nontraumatic intracranial hemorrhage. *Anaesthesiol Scandinav* 2017. Doi: 10.1111/aas.12903 | Total = 100 | **Inclusion:**  + ICU admission non-traumatic ICH  + Dec 2007-Dec 2009  **Exclusion:**  + Renal insufficiency  + Age < 18 years  + Admissin delay >38h  + AVM bleeding  + Decision to withdraw from active treatment  + Decision to transfer to the ward before recruitment  + Tumor bleeding | sICH supratentorial infratentorial | ICU mortality  1 year mortality  1 year functional outcome | Mortality  GOS  + Good (4-6)  + Bad (1-3) | Phosphate | Cut off 0.65mmol | Not stated | Included aneurysmal bleeding cases |
| 74 | Kalita et al. (2015)  India | Kalita J, Bastia J, Bhoin SK, Misra UK. Systemic inflammatory response syndrome predicts severity of stroke and outcome. *J Stroke Cerebrovas Dis 2015*; 24(7): 1640-8. Doi: 10.1016/j.jstrokecerebrovasdis.2015.03.057 | Total: | **Inclusion:**  + Ischemic and sICH during 2011-2012  + Admitted within 48h  **Exclusion:**  + AVM  + Aneurysmal bleed  + Pregnancy  + Coagulopathy  + Bleeding diathesis  + Immunosuppresion  + Organ transplantation  + Malignancy  + Septicemia before ICH  + Lack of consent  + Antiplatelet and anticoagulant  + Corticosteroid drugs  + age < 15 years | sICH and ischemic stroke | Presence of SIRS |  | + Hb  + Pletelet  + CRP  + Blood sugar  + BUN  + S creatinine  + MCV  + S prtein  + S alumbim  + S uric acid  + S cholesterol  + S LDL  + S ALP  + S bilirubin | Mean (SD) |  | Not reporting outcome of interest |
| 75 | Kang et al. (2019)  China | Kang K, Lu J, Ju Y, Wang W, Shen Y, Wang A, et al. association of pre- and post- stroke glycemic status with clinical outcome in spontaneous intracerebral hemorrhage. *Sci Rep* 2019; 9:19054. Doi: 10.1038/s41598-019-55610-z | Total: | **Inclusion** + first-ever acute-onset ICH  + Age >18  + Arriving within 72 h of onset  **Exclusion:** + Past history of ICH  + Age <18 years  + Arriving >72 h  + Comorbidities  + Secondary ICH (AVM, aneurysm, Anticoagulatn therapy, tumor, trauma, Cerebral venous thrombosis, transformation of ischemic stroke) | sICh supratentorial infratentorial | Baseline hematoma volume  Baseline NIHSS  Bseline GCS score |  | HbAc1 |  |  | Not reporting outcome of interest |
| 76 | Kayhanian *et al.* (2017)  UK | Kayhanian S, Weerasuriya CK, Rai U, Young AMH. Prognostic value of peripheral leukocyt counts and plasma glucose in intracerebral hemorrhage. *J Clin Neurosci* 2017. Doi: 10.1016/j.jocn.2017.03.032 | Total | **Inclusion:**  + Admited between 2009-2011  **Exclusion:**  + ischemic stroke  + SAH  + Sub dural hemorrhage | All ICH | In-hospital mortality | Mortality | + Glucose  + Leukocyte  + Neutrophil  + Lymphocyte  + Basophil  + Eusinophil  + monocyte | Med (IQR) | + age  + Gender  + CCI  + Smoking  + Premorbidn mRS | Not reporting defined time for outcome assessment |
| 77 | Kim et al. (2016)  South Korea | Kim YJ, Han MH, Kim CH, Kim JM, Cheong JH, Ryu JI. Increased short-term mortality in patients with spontaneous intracerebral hemorrhage and its association with admission glucose levels and leukocytosis. *World Neurosurg* 2016. Doi: 10.1016/j.wneu.2016.11.087. | Total: 538 | **Inclusion:**  + Non-traumatic sICH  + Age >18 years  + Admitted between 2008-2015  **Exclusion:**  + Infratentorial ICH  + Secondary ICH (malignancy, hemorrhagic conversion, bleeding disorder, vascular malformation)  + CT scan >72 h  + Missing results | Supratentorial sICH | 3-mo mortality | Mortality | + Sodium + Potassium + chloride  + AST  + ALT + Glucose  + WBC  + Platelet | Mean (SD) | + Age  + Gender  + Location  + hematoma volume  + Surgery  + DM  + HTN  + Alcohol  + Smoking  + Antithrombotic  + Previous stroke history |  |
| 78 | Kimura et al. (2007)  Japan | Kimura K, Iguchi Y, Inoue T, Shibazaki K, Matsumoto N, Kobayashi K, Yamshita S. Hyperglycemia independently increases the risk of early death in acute spontaneous intracerebral hemorrhage. *J Neurosurg Sci* 2007; 255: 90-4. Doi: 10.1016/j.jns.2007.02.005 | Total = | **Inclusion:**  + sICH admitted to center 2004-2006  + Admitted 24h of onset  **Exclusion:**  + Vascular malformation | Supratentorial sICH | 14-day mortality | Mortality | + HBA1c  + Cholseterol  + Triclyceride  + CRP  + Leukocyte  + Erythrocyte  + Platelet  + Albumin  + GOT  + GPT  + Sodium  + Potassium  + Glucose  + Fibrinogen  + ICH volume | Mean (SD) | + Age  + Gender  + Use of antiplatelet  + Blood pressure  + NIHSS score | No information of other secondary ICH exclusion (aneurysm, tumor, trauma) |
| 79 | Kongwad *et al.* (2018) India | Kognwad LI, Hedge A, Menon G, Nair R. Influence of admission blood glucose in predicting outcome in patients with spontaneous intracerebral hematoma. *Front Neurol* 2018; 9:725 Doi: 10.3389/fneur.2018.00725 | Total = 455 | **Inclusion:**  + sICH patients admitted from Feb 2015 – Jul 2017  **Exclusion:**  + Trauma  + AVM  + Aneurysm  + Coagulation abnormalities | sICH supratentorial infratentorial | Discharge functional outcome  3-mo functional outcome  90-day mortality | mRS  + Good (0-3)  + Poor (4-6) | + Blood glucose | Mean (SD) | + Age  + GCS  + Volume  + IVE  + Hydrocephalus |  |
| 80 | Kumar et al. (2009)  USA | Kumar MA, Rost NS, Snider RW, Chanderraj R, Greenberg SM, et al. Anemia and hematoma volume in acute intracerebral hemorrhage. *Crit Care Med* 2009; 37(4): 1442-7. Doi: 10.1097/CCM. 0b013e31819ced3a | Total = 694 | **Inclusion:**  + Primary ICH  + Admitted between 1999 to 31 Oct 2005  **Exclusion:**  + Age < 18 years  + Secondary ICH (trauma, ischemic transformation, tumor, AVM, thrombosis, vasculitis, etc.) | sICH  Supratentorial  I | 30-day mortality  Presence of IVH  ICH volume | Mortality | + Anemia  + Glucose  + WBC | Hb <12.1-13.1  Mean (SD) | + Gender  + Antiplatelet  + IVH  + ICH volume  + Age  + Lobar location + Blood pressure | Biomarkers not reported in mean or range median |
| 81 | Kuramatsu et al. (2014)  Japan | Kuramatsu JB, Bobinger T, Volbers B, Staykov D, Lucking H, Kolska SP, et al. Hyponatremia is an independent predictor of in-hospital mortality in spontaneous intracerebral hemorrhage. *Stroke*  2014; 45:1285-91. Doi:10.1161/STROKEAHA.113.0041360/DC1 | Total = 422 | **Inclusion:**  + sICH patients admitted during 2006-2010  **Exclusion:**  + Secondary ICH (anticoagulation, tumor, trauma, AVM, thrombolysis) | sICH supratentorial infratentorial | In-hospital mortality  90-day functional outcome  1-year functional outcome | mRS scores | Hyponatremia | Na<135mEq/L | Not mentioned | Biomarkers not reported in mean or range median |
| 82 | Landreneau et al. (2018)  USA | Landerenau MJ, Mullen MT, Messe SR, Cucchiara B, Sheth KN, McCullough LD, et al. CCL2 and CXCL10 are associated with poor outcome after intracerebral hemorrhage. *Ann Clin Transl Neurol* 2018; 5(8): 962-70. Do: 10.1002/acn3.595 | Total = 128 | **Inclusion:**  + Enrolled in Univerisyt of Penssylvania 2008-2013  + Age > 18  + Presented within 24h with sICH  **Exclusion:**  + AVM  AVF  + Aneurysm  + VST  + Trauma  + Malignancy  + Immunosupprssion or autoimmune  + Disability mRS>2 | sICH | 90-day functional outcome | mRS  + Good 0-3  + Poor 4-6 | CCL2  G-CSF  GM-CSF  CX3CL1  IL-10  CCL7  CCL22  IL-1ra  IL-1beta  IL-4  IL-6  IL-8  CXCL10  TNF |  | Not mentioned | Biomarker data reported was not related to mortality or functional outcome |
| 83 | Lattanzi et al. (2016)  Italy | Lattanzi S, Cagnetti C, Provinciali L, Silvestrini M. Neutrophil-to-lymphocyte ratio predicts the outcome of acute intracerebral hemorrhage. *Stroke* 2016; 47:1654-7. Doi: 10.1161/STROKEAHA>116.013627 | Total = 177 | **Inclusion:**  + Hospitalized between Jan 2008 – Sept 2015  + Stroke caused by sICH  + CT within 24h | sICH acute | 90-day functional outcome  + Good (0-3)  + Poor (4-6) | mRS  + Good 0-3  + Poor 4-6 | WBC  Neutrophil  Lymphocyte  NLR | Odds Ratio  Data range in supplemental | + HTN  + DM  + Hyperlipidemia  + AF  + Coronary artery diseiase  + Prior stroke  + Smoker  + Anticoagulatns  + Blood pressure  + IVH  + Volume  + Locations |  |
| 84 | Lattanzi et al. (2017)  Italy | Lattanzi S, Cagnetti C, Provinciali L, Silvestrini M. Neutrophil-to-lymphocyte ratio and neurological deterioration following acute cerebral hemorrhage. *Oncotarge*t 2017; 8(34): 5789-94. | Total = 192 | **Inclusion**:  + Patients from Jan 2008 = July 2016  + CT within 24h  + sICH  **Exclusion:**  + Patients presenting with IVH  + Hemorrhage secondary to brain tumor  + Dural venous sinous thromobosis  + AVM  + Aneurysm  + Immunomodulatory treatment | sICH supratentorial infratentoril | Neurological deterioration  7-day | 4 point or greater increase in NIHSS score, or >2 point decrease GCS or death | + WBC  + ALC  + ANC + NLR | Box plot | + age  + Sex  + clinical history HT, DM, hyperlipidemia, AF, Coronary artery disease, TIA, smoker  + Anticoagulant  + Blood pressure  + NIHSS admission  _ Volume  + Location | Not reporting outcome of interest |
| 85 | Lattanzi et al. (2018)  Italy | Lattanzi S, Cagnetti C, Rinaldi C, Angelocola S, Provinciali L, Silvestrini M. Neutrophil-to-lymphocyte ratio improves outcome prediction of acute intracerebral hemorrhage. *J Neurol Sci* 2018; 387: 98-102. Doi: 10.1016/j.jns.2018.01.038 | Total = 208 | **Inclusion:**  + Patients admitted between jan 2008 – march 2017  + Acute sICH  + CT neuro 24 h  **Inclusion:**  + Secondary ICH  + Isolated IVH  + Receiving immunomoculatory or immunosuppressive treatment | sICH supratentorial infratentorial | 30-day functional outcome | mRS  + Good (0-3)  + Poor (4-6) | + WBC  + Neutrophil count  + Lymphocyte  + NLR | Meand (SD) | + Age  + Sex  + HTN  + DM  + Hyperlipidemia  + AF  + Coronary artery disease  + TIA + Current smoker  + Antiplatelet  + Anticoagulant  + Blood pressure  + NIHSS score  + Volume  + Location  + IVH |  |
| 86 | Lehmann et al. (2020)  Germany | Lehmann F, Schenk LM, Schneider M, et al. Predictive Relevance of Baseline Lactate and Glucose Levels in Patients with Spontaneous Deep-Seated Intracerebral Hemorrhage. Brain Sci. 2021;11(5):633. Published 2021 May 14. doi:10.3390/brainsci11050633 | **Total** 102 | **Inclusion:**  + Spontaneous deep-seated ICH  **Exclusion** + Lobar ICH  + Underlying source of bleeding (aneurysm, AVM, trauma  + Not treated in the NICU for at least 3 days  + In disastrous clinical situation  + Withdrawal of life-sustaining treatment | sICH deep | 90-day functional outcome  90-day mortality | mRS  0-4 good  5-6 poor  Mortality | Serum lactate  Glucose | Cut-off lactate 1.6 mmol/L  Cut-off glucose 133 mg/dL  ROC curve | Not stated | Biomarkers not reported in mean or median |
| 87 | Lei et al. (2020)  China | Lei C, Geng J, Zhong L. The association between plasma HMGB1 and sRAGE and clinical outcome in intracerebral hemorrhage. *J Neuroimmunol* 2020. Doi: 10.1016/j.jenuroim.2020.577266 | Total = | **Inclusion**  + Patients within 72 h of acute ICH  + Admitted between 1 Sept 2017 to 31 Sept 2018  + | sICH | Functional outcome 90-day | mRS  + Good (0-2)  + Poor (3-6) | + CRP  + Leukocyts  + Neutropil | Mean (SD) | + Age  + Gender  + HTN  + DM  + Heart disease  + Cholsterol  + Smoking | All cause of ICH, not excluding secondary cause of ICH and traumatic ICH |
| 88 | Leira et al. (2004)  Italy | Leira R, Dávalos A, Silva Y, Gil-Peralta A, Tejada J, Garcia M, Castillo J, et a. Early neurologic deterioration in intracerebral hemorrhage. *Neurology* 2004; 63:461-7. | Total = 266 | **Inclusion**  + Spontaneous supratentorial ICH  + Admitted between May 1999 to April 2001  + Onset <12 h  + Absence of stupor or coma  **Exclusion:**  + Hematoma secondary to injury, coagulation abnormalities  + AVM, Aneurysm, Tumor | Supratentorial sICH | Early neurological deterioration | Decrease > 1 points CSS 48h , including dead | + Hb  + Leukocyte  + Neutrohpil  + Fibrinogen  + ESR  + CPK | Mean (SD0 | + Age  + Gender  + Infection  + Inflammatory disease  + Temperature | Not reporting outcome of interest |
| 89 | Li et al. (2008)  China | Li W. Serum lipid levels and 3-month prognosis in Chinese patients with acute stroke. *Adv Ther* 2008; 25(40: 329-41. Doi: 10.1007/s12325-008-0045-7 | Total = 693 | **Inclusion:**  + confirmed CT stroke  + | Ischemic and hemorrhagic stroke | 3-month functional outcome  90-day mortality | mRS  + Good (0-3)  + Bad (4-6) | + TC  + TG  + HDL-C  + LDL-C  + Glucose  + Albumin | Median (IQR) |  | Included patients with ischemic stroke |
| 90 | Li et al. (2012)  China | Li YF, Luo J, Li Q, Jing YJ, Wang RY, Li RS. A new simple model for prediction of hospital mortality in patients with intracerebral hemorrhage. *CNS Neurosci Therap* 2012; 18:482-6 | Total = | **Inclusion:**  + Retrospective ICH patient Jan 2008-Aug 2009  + Age 14-95 years | All cause ICH | In hospital mortality | Mortality | + Glucose  + TP  + ALB  + Na  + K  + Urea  + Scr  + LDL-C  + HDL-C  + TC  + TG  + WBC  + RBC + HGB | Mean (SD) | Age  + Gender  + GCS Score  + Blood pressure  + Heart rate | Not excluding secondary cause of ICH |
| 91 | Li et al. (2013)  China | Li N, Liu YF, Ma L, Worthmann H, Wang YL, Wang YJ, et al. Association of molecular markers with perihematomal edema and clinical outcome in intracerebral hemorrhage. *Stroke* 2013; 44:658-63. Doi: 10.1161/STROKEAHA.112.673590 | Total = 59 | **Inclusion**  **+** patients with sICh admitted between Jan 201 to Dec 2011  + Symptom onset on admission <24h  + Age <80 years  + Absence of coma  **Exclusion:**  + Secondary ICH from aneurysm, VM, hemorrhagic infaction, tumor, impaired coagulation  + istory of acute or chronic infection  + Malignant disease  + Immunosuppresive treatment | sICH supratentorial  (Cerebral) | 90-day functional outcome | mRS  + Good (0-2)  + Bad (3-6) | + Leukocyte  + Platelet  + Glucose  + Creatinine  + INR  + Fibrinogen  + MMP-3  + MMP-9  + VEGF  + Ang-1 | Mean (SD) | + Sex  + Age  + BMI  + HTN  + DM  + History of stroke  + Tobaco  + Alcohol  + Antiplatelet  + Initial GCS score  + NIHSS  + SBP  + DBP  + Hematoma volume  + PHE volume  + Deep location  + IVE |  |
| 92 | Li et al. (2019)  China | LI HJ, Han NN, Nan Y, Zhang K, Li G, Chen H. Plsma osteopontin acts as a prognostic marker in acute intracerebral hemorrhage patients. *Clin Chim Act* 2019. Doi: 10.1016/j.cca.2019.10.018 | Total = | **Inclusion:**  + Patients admitted within Sep 2015-Aug 2018  + Age >18 years  + mRS 0-2 prior to onset  + Basal ganglia hemorrhage on CT  + CT within 24h after stroke onset  **Exclusion:**  + Underlying vascular lesions  + Coagulative dysfunction  + Hemorrhagic infarction  + tumor  + Infection  + Neurologicla disease | sICH basal ganglia | Early neurological deterioration (END)  90-day functional outcome | Increase of > 4 points in NIHSS score or death at 24h from symptoms onset  mRS  + Good (0-2)  + Bad (3-6) | Plasma osteopontin  + Blood glucose  + CRP  + WBC count | Dot plot  Cut-off (1337.1) | + Gender  + Age  + Smoking  + Alcohol  + HTN + DM  + Hyperlipidemia  + Congestive heart failure  + Coronary artery disease  + Chronic kidney disase  + Antiplatelet  + NIHSS score  + Hematoma volume  _ Location  + IVH  + Blood pressure | Not reporting outcome of interest |
| 93 | Liotta et al. (2017) | Liotta EM, Prabhakaran S, Sangha RS, Bush RA, Long AE, Trevick SA, et al. Magnesium, hemostasis, and outcomes in patients with intracerebral hemorrhage. *Neurology* 2017; 89: 813-9 | Total = | **Inclusion:**  + patients with sICH between Nov 2006-March 2016  + CT within 48h follow-up  **Exclusion:**  + Patients with ICH attributed to trauma, hemorrhagic conversion, structural lesion, VM | sICH supratentorial infratentorial | 90-day functional outcome  Hemostasis | mRS | Magnesium | Model |  | Not reporting data of biomarker range and number of patients |
| 94 | Liu et al. (2020)  China | Liu Q, Zhao W, Xing Y, Hong Y, Zhou G. Low triglyceride levels are associated with unfavorable outcomes in patients with spontaneous intracerebral hemorrhage. *Neurocrit Care* 2020. Doi: 10.1007/s12028-020-01023-0 | Total = 1451 | **Inclusion:**  + Patiants with ICH between 2005-2018  + Symptoms lasting 24h or longer  + Admitted to hospital within 72 h  + Age > 18years  **Exclusion:**  + Diagnosed with hemorrhagic transformation after ischemic stroke  + SAH  + Traumatic hemorrhage  + Secondary hemorrhage by AVM, VST, Aneurysma  + Supratherapeutic INR  + mRS >2 | sICH supratentorial infratentorial | Functional outcome at discharge, 3 mo, 12 mo | mRS  + Good (0-2)  + Bad (3-6) | + WBC  + Hb  + TG  + TC  + HDL  + LDL  + Glucose  + CRP  + Albumin  + Creatinine | Mean (SD) | + Gender  + Age  + Educational level  + Hematoma location + GCS score  + Ich volume  _ IVH  + NIHSS + BI  + mRS  + Smoking  + Alcohol  + Hypertension  + DM  + AF  + SBP  + DBP |  |
| 95 | Lorente et al. (2020)  Spain | Lorente L, Martin MM, Ramos L, Argueso M, Caceres JJ, Sola-Violan J, et al. High serum tissue inhibitor of matrix metalloproteinase-1 levels and mortality in patients with spontaneous intracerebral hemorrhage. *World Neurosurg* 2020; 134:e476-80. Doi: 10.1016/j.wneu.2019.10.106 | Total = | **Inclusion:**  + Patients with severe supratentorial sICH  + GCS <9  **Exclusion**  + Hemorrhage infratentorial  + Traumatic  + Infarction transformation  + Age <18 years  + Pregnancy  + Malignant disease  + Inflammatory disease | Supratentorial sICH | 30-day mortality | Mortality | + Creatinine  + Sodium  + Glycemia  + Lactic acid  + INR  + Fibrinogen  + Platelet  + aPTT+ +MMP-9  +TIMP-1 | Mean (SD) | + ge  + Gender  + Site  + Volume  + IVH  + Hydrocephalus  + Midline shift  + herniation | Included secondary causes of SICH aneurysm, arteriovenous malformatin |
| 96 | Lorente et al. (2020)  Spain | Lorente L, Martin MM, Gonzalez-Rivero AF, Perez-Cejas A, Sabatel R, et al. Serum substance P levels and early mortality of spontaneous intracerebral hemorrhage patients. *J Strok Cerebrovas Dise* 20020; 29(7): 104893 | Total = | **Inclusion:**  + Patients with severe primary supratentorial ICH  + GCS score < 9  **Exclusion:**  + Traumatic hemorrhage  + Hemorrhage secondary to aneurysm  + AVM  + Anticoagulat treatment or fibrinolytic  + Age <18 years  + Pregnancy  + Malignan disease  + Asthma  + Ulcerative colitis  + Chron’s disase | Supratentorial ICH | 30-day mortality | Mortality | + Creatinine  = Sodium  + APTT  + Fibrinogen  + INR  + Platelet  + Substance P | Median (IQR) | + Age  + Gener  + SIH volume  + Site  + IVH  + Transtentorial herniation  + midline shift  + Hydrocephalus  + Ich score  + GCS score  + APACHE II socre  = Temperature  + Pa/FI O_2_ |  |
| 97 | Lorente et al. (2019)  Spain | Lorente L, Martin MM, Abreu-Gonzalez P, ramos L, Argueso M, Sole-Viola J, et al. The serum melatonin levels and mortality of patients with spontaneous intracerebral hemorrhage. *Brain Sci* 2019; 9:263. Doi: 10.3390/brainsci9100263 | Total = 100 | **Inclusion:**  + Patients with severe supratentorial sICH  + GCS <9  **Exclusion**  + Hemorrhage infratentorial  + Traumatic  + Infarction transformation  + Age <18 years  + Pregnancy  + Malignant disease  + Inflammatory disease | Supratentorial ICH | 30-day mortality | Moratlity | + Creatinine  = Sodium  + APTT  + Fibrinogen  + INR  + Platelet  + Melatonin | Median (IQR) | + Age  + Gener  + SIH volume  + Site  + IVH  + Transtentorial herniation  + midline shift  + Hydrocephalus  + Ich score  + GCS score  + APACHE II socre  = Temperature  + Pa/FI O_2_ | Included secondary causes of SICH aneurysm, arteriovenous malformatin |
| 98 | Lu et al. (2021) | Lu P, cui L, Wang Y, Kang K, Gu H, Li Z, et al. Relationship between glycosylated hemoglobin and short-term mortality of spontaneous intracerebral hemorrhage. *Front Neurol* 2021; 12:648907 | Total : 21,11I | **Inclusion:**  + Patients admitted from Aug 2015-Jul 2019  + Age >18 years  + Within 7 days of symptom onset  **Exclusion:**  + History of previous troke  + History of previous liver abnormalities  + Bleeding history or tendency  + Lack of data | Non-traumatic sICh | In-hospital mortality | Moratlity | HbA1c | Cut-off:  <5.1%  5.1-5.6  5.6-6.1  >6.1 | Not mentioned | Not reporting defined time for outcome assessment |
| 99 | Ma et al. (2015)  China | Ma BQ, Zhang M, Ba L . Plasma pituitary adenylate cyclase-activating polypeptide concentrations and mortality after acute spontaneous basal ganglia hemorrhage. *Clin Chim Act* 2015; 439:102-6. Doi: 10.1016/j.cca.2014.10.010 | Total = 150 | **Inclusion:**  + Patients with asICH within first 24h Jan 2013-Jul 2013  **Exclusion:**  + Previous stroke  + Severe head trauma  + Use of antiplatelet or anticoagulant  + Presence of systemic disease  + Surgical procedure | Acute sICH basal ganglia | 7-day mortality  6- mo mortality | Mortality | + Glucose  + CRP  + WBC  + Hb  + Platelet  + Ptb  + Thrombin time  = pTT  + Fibrinogen  + PACAP | Mean (SD) | + Gener  Age  + HTN  + DM  + NIHSS + Hematoma  + IVH  + hemorrhage growth  + END  + SAP  + DAP |  |
| 100 | Mansouri et al. (2013)  Iran | Mansouri B, Heidari K, Asadollahi S, Nazari M, Assarzadegan F, Amini A, et al. Mortality and functional disability after spontaneous intracranial hemorrhage: the predictive impact of overall admission factory. *Neurol Sci*  2013; 34:1933-9. Doi: 10.1007/s10072-013-1410-0 | Total = 120 | **Incsluion:**  + Non traumatic, non-aneurysmatic ICH presented between January and July 2012  + CT within 24h  **Exclusion:**  + Secondary etiologies of cerebral hemorrhag e(trauma, aneurysm, structural lesion, tumor)  + Referred from outlying centers | sICh supratentorial infratentorial | 30-day mortality  3-mo mortality | Mortality | +Platelet  + pTB time  + Glucose  + Sodium  + Creatinine | Mean (SD) | + Age  + Sex  + HTN  + DM  + Hyperlilidemia  + CVA  + Smoker  + Alcoho  + Antiplatelet  + IVH  + Midline shift  + Hydrocephalus |  |
| 101 | Mao et al. (2019)  China | Mao J, Jiang W, Liu G, Jiang B. Serum calcium levels at admission is associated with the outcomes in patients with hypertensive intracerebral hemorrhage. *British J Neurosurg* 2019. Doi: 10.1080/02688697.2019.1571162 | Total = 658 | **Inclusion:**  + Spontaneous hypertensive ICH  + CT within 72h  + Total serum calcium measured on admission  + Had history of HTN  **Exclusion:**  + Traumatic  + Tumor intracranial  + Vscular malformation rupture  + Hemorrhagic conversion  + Complicated with systemic disase | sICH hypertensive | 30-day mortality  6-mo mRS | Mortality  mRS (range) | Calcium concentration | T1: <2.25  T2: 2.25-2.75  T3: >2.75 | Not related to outcome | - Biomarkers not reported in mean or range median |
| 102 | Marti-Fabregas *e t a.* (2010)  Spain | Marti-Fabregas J, Borrell M, silva , Delgado-Mederos R, Martinez-Ramirez S, de Juan-Delago M, et al. Hemostatic proteins and their association with hematoma growth in patients with acute intracerebral hemorrhage.*Stroke* 2010; 41:2976-8 | Total = | **Inclusion:**  + Patients with spontaneous supratentorial ICH diagnosed within the first 6h after the onset of symptoms  + Follow-up CT obtained within 24 to 72 h after onset symptom  **Exclusion:** + Patients treated with emergent surgical evacuation  + Died before a fello-up CT  + ICH attributable to anticoagulants  + Bleeding diathesis  + tmor and arterio-venous malformation | sICH supratentorial | Hemostasis within 24-72 h | MRI | + Fibrinogen  + Factor XIII  + Functional thrombin  + Plamiogen  + a2-Antiplasmin  + Tissue plasminogen activator  + D-dimer  + Thrmbomodulin  + Trhombin-antithrombin complex  + Plasmin-antiplasmin complex | Mean (SD) | Not stated | Not reporting outcome of interest |
| 103 | Mehdiratta et al. (2008)  USA | Mehdiratta M, Kumar S, Hackney D, Schlaug G, Selim M. Association between serum ferritin level and perihematomal edema volume in patients with spontaneous intracerebral hemorrhage. *Stroke* 2008; 39:1165-70. Doi: 10.1161/STROKEAHA.107.501213 | Total = | **Inclusion:**  +Admitted between 2006-2007  + Acute sICH with follow-p CT at day 3-4  **Exclusion:**  + Anticoagulant  + AVM  + TUmor  + Trauma  + Transformation  + Initial or subsequent IVH and infratentorial hemorrhage  + Died within 3-4 days | Suprantentorial sICH | Hematoma volume  Absolute edema  Relative edema | Imaging | Serum ferritin | Dot plot | Not mentioned | Not reporting outcome of interest |
| 104 | Menon et al. (2020)  USA | Menon G, Johnson SE, Hedge A, Rathod S, Nayak R, Nair R. Neutrophil to lymphocyte ratio – a novel prognostic marker following spontaneous intracerebral hemorrhage. *Clin Neurol Neurosurg* 2021; 200:106339 | Total = 851 | **Inclusion:**  + patients admitted between Jan 2015-Dec 2018 for acute hemorrhagic stroke  + Age >18 years  **Exclusion:**  + Traumatic ICH  + ICH secondary to vascular lesions, aneurysm, lesions  + Coagulopathy | sICH  Supratentorial  Infratentorial | 90-day functional outcome  Discharge functional outcome  Mortality | mRS  + Good (0-3)  + Bad (4-6) | + NLR + Neutrophil  + Lymphocite  + TC | Man (SD)  Cut-off 8.2 | + Age  + GCS <8  + Volume  + IVH  + Hydrocephalus |  |
| 105 | Miao et al. (2013)  China | Miao W, Zhang Y, Li H. Bispectral index predicts deaths within 2 weeks in coma patients, a better predictor than serum neuron-specific enolase or S100 protein. *J Anesth* 2013; 27:855-61. Doi: 10.1007/s00540-013-1654-0 | Total = 90 | **Inclusion:**  + Coma patients with sever brain injury  **Exclusion:**  + severe cardiac, respiratory, renal disease  + Died of extracerebral lesion  + Received sedatives or muscular relaxants | Brain injury | BIS value | BIS index | S100 protein  NSE | Mean (SD) | +Cause of brain injury  + | Not reporting outcome of interest |
| 106 | Miao et al. (2021)  China | Miao Y, Zhang Z, Feng X, Sun W. IL-33 as a novel serum prognostic marker of intracerebral hemorrhage. *Oxidat Med Cell Longetiv* 2021. Doi: 10.1155/2021/5597790 | Total = 713 | **Inclusion:**  + Patients admitted between Jan 2018 to Dec 2020  +Hospitalized within 24 h of onset  + CT diagnosed ICH  **Exclusion:**  + Secondary ICH by trauma, tumor, AVM  + mRS > 3  + Needed surgical intervention  + Subtentorial hemorrhage  + Sever liver and kidney dysfunction | Supratentorial sICH | 90-day functional outcome | mRS  + Good (0-2)  + Bad (3-6) | +TG  + LDL-C  + HbA1c  + FBG  + APTT  + INR + IL-33 | Mean (SD) | +Age  + Sex  + HT  + DM  + Hyperlipidemia  + CHD  + SBP  + DBP  + NIHSS score  + GCS score  + Hematoma volume |  |
| 107 | Morotti et al. (2016)  USA | MOrotti A, Charidimou A, Phuah CL, Jessel MJ, Schwab K, et al. Association between serum calcium level and extent of bleeding in patients with intracerebral hemorrhage. *JAMA Neurol* 2016; 73(11): 1285-90. Doi: 10.1001/jamaneurol.2016.2252 | Total = 2103 | **Inclusion:**  + Patients admitted between 1994-2015  + Diagnosis of sICH by NCCT performed within 72h  + Total serum measurement on admission  **Exclusion:**  + traumatic ICH  + Tumor, AVM  + Primary IVH  + Hemorrhagic conversion | sICH supratentorial infratentorial | 30-day mortality | Mortality | Serum calcium | Cut-off <8.4 mg/dL | Not related to outcome | Biomarkers not reported in mean or range median |
| 108 | Muller et al. (2019)  Germany | Muller M, Tapia-Perez JH, Yildiz C, Rashidi A, Luchtmann M. Alterations in inflammatory markers and clinical outcome after spontaneous intracerebral hemorrhage – Preliminary results. *J Stroke Cerebrovas Dis* 2020; 29(8): 104861 | Total =27 | **Inclusion:**  + sICH patients between Jul 2013-Feb 2016  + Age > 18 years  + Admission witin 24h onset  + CT diagnosis  **Exclusion:** + Pupils dilated and not reactive to light on both sides  + Previous brain damage  + Neurological disability  + Immunological disorder  + ICH volume <10mL  + Secondary ICH (trauma, tumor, postoperative)  + Multiple ICH  + Subdural or epidural hematoma  + Pregnancy |  | 30-day mortality  90-day mortality  30-day functional outcome  90-day functional outcome | Mortality  mRS  + Good (0-2)  + Bad (3-6) |  |  |  | Biomarker data reported was not related to mortality or functional outcome |
| 109 | Palm et al. (2013)  Germany | Palm F, Henschke N, Wolf J, Zimmer K, Safer A, Schroder RJ, et al. Intracerebral hemorrhage in a population based stroke registry (LuSSt): Incidence, aetiology, functional outcome, and mortality. *J Neurol* 2013; 260:2541-50. Doi: 10.1007/s00415-013-7013-0 | Total = 152 | **Inclusion:**  + Primary ICH Jan 206-Dec 2010  + All-causes of sICH | All cause sICH | 1-year mortality  1-year functional outcome | mRS  + Good (0-2)  + Bad (3-6) | +Leukocyte  + Glucose  + CRP | Med (IQR) | + Medical history  + Anticoagulation  + Hematoma site  +Hematoma volume  +IVH  +Midline shift  + Etiology | Included causes of secondary ICH (AVM, tumor, etc.) |
| 110 | Qian et al. (2018)  China | Qian SQ, He SR, Li BB, Qian J, Zheng XD. Serum S100A12 and 30-day mortality after acute interacerebral hemorrhage. *Clin Chim Act* 2018; 477:1-6. Doi: 10.1016/j.cca.2017.11.032 | Total = 234 | **Inclusion:**  + Patients with acute sICH between 2013-2016  + Admitted within the first 24h after onset  + Free of conditions such as infection, immunosupressan, autoimmune, uremia, liver cirrhosis, malignancy, chronic heart or lung disease  **Exclusion:**  + Neurological disease  + Ischemic stroke  + Prior hemorrhagic stroke  + Sever head trauma  + Antiplatelet  + Surgical procedure | sICH basal ganglia | 30-day mortality | Mortality | + Glucose  + CRP  + WBC  + Serum S100A12 | Mean (SD) | + Age  + HTN  + DM  + NIHSS score  + Hematoma vlume  + IVH  + END | Not reporting data of biomarker range and number of patients |
| 111 | Qiu et al. (2016)  China | Qiu SZ Wang HX, Shen J, Zheng GR, Chen B, Huang JJ, Gao JB. The prognostic value of serum signal peptide-Cub-Egf domain-containing protein-1 concentrations in acute intracerebral hemorrhage. *Clin Chim Act* 2016; 461:103-9. | Total = 128 | **Inclusion:**  + Patients sICH admitted within 24h of onset  **Exclude**  + Acute or chronic infections in the 4 weeks befor ICH  + Previous ischemic or hemorrhagic score  + Severe head trauma  + Use of antiplatelet or anticoagulant  + Malignancy | sICH infratentorial supratentorial | + Hematoma growth  + END  + 7-day mortality  + 6-mo mortality  + 6-mo functional outcome | Imaging  NIHSS > 4 points within 24h of symptoms onset  Mortality  mRS  + Good (0-2)  + Bad (3-6) | Serum SCUBE1 level | Box plot | Not mentioned | Biomarkers not reported in other studies |
| 112 | Rajapathy et al. (2017)  Malaysia | Rajapathy SK, Idris Z, Kandasamy R, Sii Hieng AW, Abdullah JM. Inflammatory biomarkers and their value in predicting survival and outcome among patients with spontaneous intracerebral haemorrhage. *Malays J Med Sci* 2017’24(3): 51-65. Doi: 10.21315/mjms2017.24.3.7 | Total = 60 | **Inclusion:**  + Age between 30-75 years  + Prsented with sICH secondary to uncontrolled hypertension  + GCS admission 9-14/15  ICH size 10-30 cm  **Exclusion:**  + Infratentorial bleed  + Intraventricular bleed  + Brainstem or thalamic bleed  + Pregnancy  + Incomplete study duration  + Positive infection  + comorbidity | sICH lobar with GCS >9 | 6-mo functional outcome  6-mo mortality | GOS  + Good (4-5)  + Bad (2-3) | + WBC admission  + WBC 72h  + CRP admission  + CRP 72h | Mdian (IQR)  CRP positive | + Age  + Gender  + Clot  + GCS median |  |
| 113 | Rajendran et al. (2020)  India | Rajendran SR, Periyasamy S, Manjuladevi MT, George N. Evaluation of serum ferritin as a prognostic marker in acute hemorrhagic stroke. *J Neurosci Rural Pract* 2020; 11:72-7. Doi; 10.1055/s-0039-1700597 | Total = 50 | **Inclusion:**  + First stroke episode  + Primary supratentorial hemorrhage  **Exclusion:**  + Secondary ICH  + Ischemic stroke  +Anemia  + Sever alcohol consumption  + Chronic liver disease  + Chronic kidney disease  + Hematological malignancies | Supratentorial sICH | 7-day mortality  7-day functional outcome | Mortality  mRS  + Good (0-2)  + Bad (3-5) | Ferritin | Median (IQR) | + Age  + SBP  + DBP  + PP  + MAP  + GCS  + ICH volume | Biomarker is not reported in other studies |
| 114 | Rendevski et al. (2018)  Macedonia | Rendevski V, Aleksovski B, Stojanov D, Rendevska AM, Aleksovski V, Gjorgoski I. Modeling prognostic factors of poor neurological outcome in conservatively trated patients with intracerebral hemorrhage: A focus on TNF-a. *Clin Neurol Neurosurg* 2018; 172: 51-8. Doi: 10.1016/j.clineuro.2018.06.027 | Total = 50 | **Inclusion:**  + Patients diagnosed with acute, primary, supratentorial intracerebral hemorrhage  + Without coma  + Hospitalized during 24h after onset  **Exclusion:**  + Neurodegenerative disorders  + Pulmonary  + Renal and hepatic disease  + coagulopathies  AVM  + Aneurysm  + Intratumoral hemorrhage  + Coagulant therapy  + surgical intervention  + IVH  + SAH  + Hematoma expansion | sICH supratentorial | 3-mo functional outcome | CSS score  + Good >7  + Poor <7 | + Glutamate  + TNF-a | Correlation to CSS score | + Gender  + SBP  + DBP  + DM  + Initial ICH volume  + ICH 5d  + Edema 5d  + CSS score on evaluation | Biomarkers not reported in mean or range median |
| 115 | Rendevski et a. (2018)  Macedonia | Rendevski V, Aleksovski B, Stojanov D, Aleksovski V, Rendevska AM, Kolevska M, et al. Peripheral glutamate and TNF-a levels in patients with intracerebral hemorrhage: Their prognostic values and interactions toward the formation of the edemal volume. *Neurolgia I Neurochirugia Polska* 2017. Doi: 10.1016/j.pjnns.2017.10.003 | Total = 50 | **Inclusion:**  + Patients diagnosed with acute, primary, supratentorial intracerebral hemorrhage  + Without coma  + Hospitalized during 24h after onset  **Exclusion:**  + Neurodegenerative disorders  + Pulmonary  + Renal and hepatic disease  + coagulopathies  AVM  + Aneurysm  + Intratumoral hemorrhage  + Coagulant therapy  + surgical intervention  + IVH  + SAH  + Hematoma expansion | Acute primary supratentorial ICH | Comparison with control |  | + Glutamate  + TNF-a | Med (IQR) | + Gender  + SBP  + DBP  + DM  + Initial ICH volume  + ICH 5d  + Edema 5d  + CSS score on evaluation | Not reporting outcome of interest |
| 116 | Richard et al. (2015)  France | Richard S, Lagerstedt L, Burkhard PR, Debouverie M, Turck N, Sanchez JC. E-selectin and vascular cell adhesion molecule-1 as biomarkers of 3-month outcome in cerebrovascular diseases. *J Inflamm* 2015; 12:61. Doi 10.1186/s12950-015-0106-z | Total = 100 | **Inclusion:**  + Admitted within 36h of onset of stroke or transient ischemic attack  **Exclusion:**  + Pregnancy  + Cancer  + Liver cirrhosis  + Renal failure  + Myocardial infarction  + Ongoing treatment with neuroleptics or lithium | Hemorrhagic and ischemic stroke | 3-mo functional outcome | mRS  + Good (0-2)  + Bad (3-6) | + E selectin  + VCAM-1  + CRP  + IL-6  + NT-proBNP  + S100B | Mean (SD) | + Age  + Sex  + NIHSS + SBP  + DBP  + Hypertension  + DM  + Dyslipidemia  + Tobacco  + Alcohol  + AF  + Coronary disease | Included cases of ischemic stroke |
| 117 | Rodriguez-yanez et a. 2012  Spain | Rodriguez-Yanez M, Brea D, Arias S, Blanco M, Pumar JM, Castillo J, Sobrino T. Increased expression of Toll-like receptors 2 and 4 is associated with poor outcome in intracerebral hemorrhage. *J Neuroimmunol* 2012; 247:75-80. Doi: 10.1016/j.jneuroim.2012.03.019 | Total = 185 | **Inclusion:**  + Patients recruited between June 2008-June 2010  + First ever hemispheric non-traumatic ICH <24h  **Exclusion:**  + Chronic inflammatory disease  + Sever hepatic  + Renal disease  + Hematological disease  + Cancer  + Infectious disease  + Anti-inflammatory treatment | sICH supratentorial infratentorial | 3-mo functional outcome | mRS  + Good (0-2)  + Bad (3-6) | + TLR2 neutrophil  + TLR 2 monocyte  + TLR4 neutrophil  + TLR4 monocyte  + Fibrinogen  + Leukocyte  + Platelet  + Hb  + Glucose | Mean (SD) | + Age  + Sex  + HTN  + DM  + DYslipidemia  + Alcohol  + Smoking  + AF  + Statin  +Anticoagulant  + SBP  + DBP | Not excluding cases of secondary ICH caused by tumor or arterious malformations |
| 118 | Ray-O’Reilly et al. (2017)  California | Roy-O’’Reilly M, Zhu L, Atadja L, Torres G, Aronowski J, McCullough L, Edwards NJ. Soluble CD163 in intracerebral hemorrhage: biomarker for perihematomal edema. *Ann Clin Transl Neurol* 2017; 4(11):793-800. Doi: 10.1002/acn3.485 | Total = 51  PHE high = 21  PHE low = 30 | **Inclusion:** + Primary IHC  + Homogenous ICH characteristics  + Deep supratentorial hemorrhage  + Hypertensive  **Exclusion:**  + Coagulopathy  + Patients with secondary ICH | sICH supratentorial deep hypertensive | 90-day mortality  90-day functional outcome | mRS | PHE high  PHE low | Imaging | Not related to outcome | Not reporting relation of biomarker to outcome |
| 119 | Sakamoto et a. (2013)  Japan | Sakamoto Y, Koga M, Yamagami H, Okuda S, Okada Y, Kimura K, et al. Systolic blood pressure after intravenous antihypertensive treatment and clinical outcomes in hyperacute intracerebral hemorrhage. *Stroke* 2013; 44:1486-51. Doi: 10.1161/STROKEAHA.113.001212 | Total = 211 | **Inclusion:**  + Acute sptontaneous supratentorial Ich patients with hypertension  + Treated within 3 h from onset  + Age > 20years  + GCS >5  + CT <2.5 h  + Hematoma volume <60mmL  + Absence of extensive IVH  **Exclusion:**  + Ich because of cerebral neoplasm  + AVM  + Aneurysm  + Trauma  + Bleeding diathesis  + Coagulopathy  + Surgical intervention  + Pregnancy  + Parturition within the previous 30 days or active lactation  + PTT >1.7  + Warfarin intake  + Platelet count <50,000 | Spontaneous supratentorial hypertensive ICH | 3-mo functional outcome  Neurological deterioration 72 h | mRS  + Good (0-3)  + Bad (4-6)  Increase >4 points of NIHSS or >2 points of GCS | + Albumin  + Leukocyte count  + Glucose  + Cholesterol  + Creatinine | Med (IQR) | + Sex  + Age  + history of stroke  + Antithromobotic medication  + Cirrhosis  + HTN  + DM  + Hyperlipidaemia  + Smoking  + Alcohol  + SBP  + HR  + NIHSS + Hematoma volume  + Location | Included cases of chronic severe systemic disease |
| 120 | Sallinen 2019  Finand | Sallinen H, Wu TY, Meretoja A, Putaala J, Tatlisumak T, Strbian D. Effect of baseline hypocalcaemia on volume of intracerebral haemorrhage in patients presenting within 72 hours from symptom onset. *J Neurol Sci* 2019; 403: 24-9. Doi: 10.1016/j.jns.2019.05.033 | Total = 1013 | **Inclusion:**  + patients presented between 2005-2010  + Anticoagulant only warfarin  **Exclusion:**  + Trauma  + Tumour | sICH hypertensive, amyloid, anticoagulation | 90-day mortality | Mortality | Serum calcium | Cut-off 1.16  <1.16 hypo  1.16-1.30 normo | Not related to outcome | Biomarkers not reported in mean or range median |
| 121 | Sasmita et a. (2019)  Indonesia | Sasmita PK, Setyopranoto I, Wibowo S, Sadewa AH. Correlation between serum S100b protein level with neurological deficit in patients with acute intracerebral hemorrhage. *Bali Med J* 2019; 8(1): 63-8. | Total = 46 | **Inclusion:**  + ICH noted on CT scan  + Age > 30 years  + Admission time < 48h  + No other previous systemic disease + Give inform consent  **Exclusion:**  + History of head trauma  + History of previous stroke or TIA or subarachnoid hemorrhage | sICH supratentorial infratentorial | Survival on admission | Mortality | S100beta  + Glucose  + WBC | Med (Range) | + BP  + HR + Respiration rate  + Temperature | Not reporting outcome of interest |
| 122 | Silva et al. (2004)  Spain | Silva Y, Leira R, Tejada J, Lainez JM, Castillo J, Davalos A. Molecular signatures of vascular injury are assoiated with early growth of intracerebral hemorrhage. *Stroke* 2005; 36:86-91. Doi: 10.1161/01.STR.0000149615.51204.0b | Total = 266 | **Inclusion:** + Primary hemispheric ICH  + Admitted within 12 h  **Exclusion:** + Age <18 years  + Coma with expected death within 48h  + Hemorrhage secondary to brain tumor, trauma, drug abuse, coagulation diseorder, anticoagulant therapy, vascular malformation  + Patienst without stored frozen blood samples  + CT not available at 48h | sICH supratentorial | Early growth of hemorrhage | CT scan | + Glucose  + Leukocyte  + platelet  + Fibronogen  + Prothrombin time  + aPTT | Mean (SD) | + Age  + Gender  + History of hypertension  + Alcohol  + Smoking  + DM  + Hepatopathy  + Previous troke+ Infection  + Cognitive decline | Not reporting outcome of interest |
| 123 | Sobrino et all (2009) | Sobrino T, Arias S, Rodriguez-Gonzalez R, Brea D, Silva Y, de a Ossa NP, et al. High serum levels of growth factors are associated with good outcome in intracerebral hemorrhage. *J cerebral Blood Flow Metabol* 2009; 29:1968-74. | Total = 95  Good = 39  Bad = 56 | **Inclusion:**  + Patients admitted between Feb 2005-March 2007  + Primary ICH <12 h of symptoms onset  + Previously independent of daily activities  + Not in anticoagulant treatment  **Exclusion:**  + Chronic inflammatory disease  + Severe hepatice  + Renal disease  + Hematological disease  + Cancer  + Infectious disease in 15 days | sICH | 90-day functional outcome | mRS  + good 0-2  + Bad (3-6) | + Glucose  + Fibrinogen + Leukocyte  + Platelet  + INR  + PTT  Biomarkers at baseline, 24h, 72h  + VEGF  + GCSF  + Ang-1 | Mean (SD) | + Age  + Geder  + HTN  Diabetes  + AF  + Smoking  + Alcohol  + Previous stroke  + ICH volume  + IVH  + Lobar  +NIHSS |  |
| 124 | Sun et al (2013)  China | Sun W, Pan W, Kranz PG, Hailey CE, Williamson RA, et al. Predictors of late neurological deterioration after spontaneous intracerebral hemorrhage. *Neurocrit Care* 2013. Doi: 10.1007/s12028-013-9894-2 | Total = 149 | **Inclusion:**  + Had an admission diagnosis of sICH supratentorial  + Admission within 24h  **Exclusion:**  + ICH secondary to trauma, hemorrhagic conversion, tumor, AVM, coagulopathy  + Aneurysmal SAH  + Institution of comfort measure, death  + Craniotomy prior to 48h  + Missing GCS data | Supratentorial sICH | Late neurological deterioration 7-day | NIHSS >4 | + WBC  + Platelet  + Glucose | Med (IQR) | + Age  + Gender  + Race  + HTN  + DM  + Hyperlipidemia  + Stroke  + Coronaropathy  + AF+ SBP  + DBP  + Temp  + GCS  + Location  + IVH  + Hematoma volume  + MLS | Not reporting outcome of interest |
| 125 | Sun et al. (2017)  China | Sun Y, You S, Zhong C, Huang Z, Hu L, Zhang X, et al. Neutrophil to lymphocyte ratio and the hematoma volume and stroke severity in acute intracerebral hemorrhage patients. *Am J Emrg Med* 2017; 35:429-33. Doi: 10.1016/j.ajem.2016.11.037 | Total = | **Inclusion:**  + Admitted 2011-2014  + CT confirned stroke  **Exclusion:**  + Trauma  + Brain tumor  + Hemorrhagci transformation of IS  + Vascular cerebral malformation  + Surgery  _+ Time from onset to admission >7 days  + no MRS score  + no lab data | sICH supratentorial infratentorial | Neutrophil to lymphocyte ratio | Lab data | + TG  + TC  + LDL-C  + HDL-C  + FPG  + HTN  + DM  + Stroke  + Vol  + Location | Med (IQR) | + Age  + Gender  + Race  + HTN  + DM  + Hyperlipidemia  + Stroke  + Coronaropathy  + AF+ SBP  + DBP  + Temp  + GCS  + Location  + IVH  + Hematoma volume  + MLS | Not reporting outcome of interest |
| 126 | Svenson et al. (2017)  Sweden | Svensson EH, Soderholm M, Abul-Kasim K, Engstrom G. Tumor necrosis factor receptor 1 and 2 are associated with risk of intracerebral hemorrhage. *Stroke* 2017; 48:2710-5. Doi: 10.1161/STROKEAHA.117.017849 | Total = | **Inclusion:**  + Age 45-73  + Examination during 1991-1996  + CT diagnosed  **Exclusion:**  + Secondary causes (trauma, AVM, hemorrhagic infarction) | sICH supratentorial infratentorial | 3-mo functional outcome | mRS + Good (0-2)  + Bad (3-6) | TNFR1  TNFR2 | Odds ratio | Not related to outcomes | Not reporting data of biomarker range and number of patients |
| 127 | Tao et al. (2016)  China | Tao C, Hu X, Wang J, You C. Effect of admission hyperglycemia on 6-month functional outcome in patients with spontaneous cerebellar hemorrhage. *Med Sci Monit* 2017; 23:1200-7. Doi: 10.12659/MSM/900202 | Total = 77  Good = 27  Bad = 50 | **Inclusion:**  + Patients with acute spontaneous cerebellar hemorrhage  + Admitted within 24h after onset  + Admitted between Sep 2010 and April 2015  **Exclusion:**  + head trauma  + Coagulopathy  + Warfarin therapy  + Cerebral venous thrombosis  + Hemorrhagic transformation  + Ischemic stroke  + AVM  + Aneurysm  + Tmor | sICH infratentorial cerebellum | 6-mo functional outcome | mRS  + Good (0-2)  + Bad (3-6) | Blood glucose | Med (IQR) | + Age  + Gender  + HTN  + DM  + Drinking  + Smoking  + SBP  + DBP  + GCS score  + Hemorrhage location  + SAH  + IVH  + FVC  + BSC  + Hydrocephalus  + Hematoma size  + Surgery |  |
| 128 | Tao et al. (2017)  China | Tao C, Hu X, Wang J, Ma J, Li H, You C. Admission neutrophil count and neutrophil to lymphocyte ratio predict 90-day outcome in intracerebral hemorrhage. Biomark Med. 2017;11(1):33-42. doi:10.2217/bmm-2016-0187 | Total = 336 | **Inclusion:**  + Diagnosis of ICH confirmed by CT or MRI  + Admission within 24h from onset  + Age >18 years  **Exclusion** + Concurrent disease which may affect the value of NLR  + Systemic inflammatory disease  + neoplasm  + Autoimmune disease  Dementia  + mRS before onset >3  + Conccurrence with coagulation disorders  + Secondary hemorrhage due to trauma, tumor, aneurysm, AVM, hemorrhagic transformation  + History of severe renal dysfunction | sICH supratentorial (deep, lobar) infratentorial | 90-day mortality    90-day functional outcome | Mortality  mRS  + Good (0-2)  + Bad (3-6) | + Glucose  + WBC  + Neutrophil  + Lymphocyte  + Monocyte  + NLR | Median (IQR) | + Age  + Gender  + HTN  + DM  + Alcohol  + Smoking  + Antiplatelet  + SBP  + DBP  + GCS Score  + Ich location + Midline shift  + SAH  + IVH  + ICH volume |  |
| 129 | Tapia-Perez et al. (2016)  Germany | Tapia-Perez JH, Karagianis D, Zilke R, Koufuglou V, Bondar I, Schenider T. Assessment of systemic cellular inflammatory response after spontaneous intracerebral hemorrhage. Clin Neurol Neurosurg 2016; 150: 72-9. Doi: 10.1016/j.clineuro.2016.07.010 | Total = 43  Died = 8  Survivor = 35 | **Inclusion:**  + Patients admitted from Dec 2010 to Oct 2011  **Exclusion:**  + History of cerebral tumor  + hemorrhage after ischemic stroke | ICH supratentorial infratentorial | 30-day mortality  90-day mortality | Mortality | + Glucose  + Platelet  + Leukocyte  + Neutrophil  + Lymphocyte  + Monocyte  + IL-6  + CRP  + Hematocrit | Mean (SD) | + Age  + Sex  + HTN  + Infarction  + DM  + Prior ICH  + Antiplatelet drugs  + Anticoagulatin  + Initial GCS <9  + NIHSS <15  Blood pressure  + ICH volume  + IVH  + Hydrocephalus  + Supratentorial location |  |
| 130 | Trifan et al. (2020)  USA | Trifan G, Testai FD. Systemic immune-inflamamation (SII) index predicts poor outcome after spontaneous supratentorial intracerebral hemorrhage. J Stroke cerebrovas dis 2020; 29(9): 105057. Doi: 10.1016/j.jstrokecerebrovasdis.2020.105057 | Total = 239 | **Inclusion:**  + Patients admitted from 2007 to 2015 with diagnosis of Ich  + Age >18 years  + Available peripheral laboratory data on admission  + mRS at hostpital discharge  **Exclusion:**  + Secondary ICH  + Surgery  + Brainstem or cerebellar hematoma | sICH supratentorial | Discharge functional outcome | mRS  + Good (-0-3)  + Bad (4-6) | + Lymphocyte  + Neutrophil  + Platelet  + SII index | Med (IQR) | + Age  + Gender  + GCS  + Ischemic  + ICH  + HTN  + DM  + CKD  + Ich volume  + IVH  + Location | Not defining certain time of outcome |
| 131 | Tu et al. (2018)  China | Tu L Liu X, Li T, Yag X, Ren Y, Zhang Q, et al. Admission serum calcium level as a prognostic marker for intracerebral hemorrhage. Neurocrit Care 2018. Doi: 10.1007/s12028-018-0574-0 | Total = 1262  Poor = 730  Good = 532 | **Inclusion:**  + Patients admitted between Jan 2012 to Jone 2017  + CT within 12 h  + Patients died due to ICH  **Exclusion:**  + Trauma, intraceranial tumor, primary IVH, secondary ICH  + Hepatic cirrhosis | sICH supratentorial infratentorial | 90-day functional outcome | mRS  + Good (0-2)  + Bad (3-6) | Serum calcium level  + RBG  + APTT  + INR | Mean (SD) | + Age  + Sex  + SBP  + DBP  + anti hypertensive drugs  + CHD  + MI  + Ischemic stroke  = History of hemorrhagic stroke  + Anticoagulate  + Antiplatelet  + Location + hematoma extension |  |
| 132 | Unden et al. (200)  Sweden | Unden J, Strandberg K, Malm J, Campbell E, Rosengren L, Stenflo J, et al. Explorative investigation of biomarkers of brain damage and coagulation system activation in clinical stroke differentiation. J Neurol 2009; 256:72-77. Doi: 10.1007/s00415-009-0054-8 | Total = 97 | **Exclusion:**  + patients with SAH and with previous stroke  + Transcient ischemic attack during study period | ICH | Comparison between Ich and infarct |  | + GFPA  + APC-PCI  + NSE  + S100-B |  |  | Not reporting outcome of interest |
| 133 | Wang et al. (2011)  China | Wang KW, Cho CL, Chen HJ Liang CL, Liliang PC, Tsai YD, et al. Molecular biomarker of inflammatory response is assocated with rebleeding in spontaneous intracerebral hemorrhage. Eur Neurol 2011; 66:332-7. Doi: 10.1159/000332027 | Total = 59  Rebleeding = 39  No-rebleed = 20 | Exclusion:  **+** History of stroke  + Infection within 3 mo  + Concurrent major cardiac, renal, hepatic, autoimmune, cancerous disease  + Previous head trauma |  | Rebleeding vs no-rebleeding  30- day functional outcome | mRS  + Good (0-3) + Bad (4-6) | + IL-10  + ICAM-1  + C3  + Glucose | Med (IQR) |  | Included patients with rupture of cerebral aneurysm, AVM, undefined etiiologies |
| 134 | Wang et al. (2016)  China | Wang F, Hu S, Ding Y, Ju X, Wang L, Lu Q, Wu X. Neutrophil-to-lymphocyte ratio and 30-day mortality in patients with acute intracerebral hemorrhage. J Stroke Cerebrovas Dis 2015; 25(1): 182-7. Doi: 10.1016/j.jstrokcerebrovasdis.2015.09.013 | Total = 224 | **Inclusion:**  **+** Patients with sICH hospitalized between Jan 2012 and Jan 2014  + Age >18  **Exclusion:**  + Admitted more than 24h  + Hematologic disorders  + Immunosupressant drug users  + Trauma  + Oral anticoagulation drugs  + History of infection within 2 weeks befor ICH  + History of stroke within 6 months  + History of malignancy | sICH supratentorial infratentorial | In-hospital mortality | Mortality | + NLR 0  + NLR discharge | Mean (SD) | + age  + Sex  + HTN  + Supratentorial  + IVH  + Pneumonia  + Surgery  + ICH volume  + Arterial pressure  + GCS score | Not reporting defined time for outcome assessment |
| 135 | Wang et al. (2018)  China | Wang F, Xu F, Quan Y, Wang L, Xia JJ, Jiang TT, et al. Early increase of neutrophil-to-lymphocyte ratio predicts 30-day mortality in patients with spontaneous intracerebral hemorrhage. CNS Neurosci Ther 2019; 25:30-5. Doi: 10.1111/cns.12977 | Total = 275 | **Inclusion:**  + Primary sICH verified by CT scans  + Age > 18 years  **Exclusion:**  + Hspital admission >24h after disease onset  + Comorbidity with hematologic disorders of istory of malignancy  + Use of immunosupresant  + History of infection within 2 weeks  + Stroke within past 6 mo | sICH supratentorial infratentorial | 30-day mortality | Mortality | + NLR T1-T3 | Dot plot | + Age  + Sex  + HTN  + DM  + Smoking  + Alcohol  + AF  + Cerebrovascular history  + Dyslipidaemia  + Site of bleeding  + ICH volume  + Bood pressure | Not excluding secondary causes of ICH by AVM, aneurysm, tumor |
| 136 | Wang et al (2018)b  China | Wang F, Wang L, Jiang T, Xia J, Xu F, Shen L, et al. Neutrophil-to-lymphocyte ratio is an independent predictor of 30-day mortality of intracerebral hemorrhage patients: a validation cohort study. Neurotox Res 2018. Doi: 10.1007/s12640-018-9890-6 | Total = | **Inclusion:**  + Adult ICH patients Jan 2016- Jan 2017  + Primary sICH verified by CT scans  + Age > 18 years  **Exclusion:**  + Hspital admission >24h after disease onset  + Comorbidity with hematologic disorders of istory of malignancy  + Use of immunosupresant  + History of infection within 2 weeks  + Stroke within past 6 mo | sICH supratentorial infratentorial | 30-day mortality | Mortality | + WBC  + Neutrophil  + Lymphoctye  + NLR + CRP  + Fibrinogen | Mean (SD) | + Age  + Sex  + HTN  + DM  + Smoking  + Alcohol  + AF  + Cerebrovascular history  + Dyslipidaemia  + Site of bleeding  + ICH volume  + Bood pressure | Not excluding secondary causes of ICH by AVM, aneurysm, tumor |
| 137 | Wang et al. (2018)c  China | Wang L. Huangfu X, Tao B, Zhong GJ, Le ZD. Serum tenacin-C predicts severity and outcome of acute intracerebral hemorrhage. Clinica Chimica Act 2018; 481:69-74. Doi: 10.1016/j.cca.2018.02.033 | Total = | **Inclusion:**  + Patients with basal ganglial hemorrhage admitted within 6h from symptoms between Jul 2014 to Dec 2016.  **Exclusion:**  + Antiplatelet or anticoagulant  + Underwent surgical procedure  + Autoimmune disease, uremia, liver cirrhosis, malignancy, chronic heart or lung disease  + Previous neurological disease: ischemic or hemorrhagic stroke and head trauma | Basal ganglia hemorrhage | + Hematoma growth + END  + 90-day mortality  + 90-day functional outcome | + Imaging  + NIHSS > 4 within 24 h  + Mortality  + mRS | + Hb  + Platelet  + Fibrinogen  + D dimer  + Glucose  + CRP  + WBC  + TNC | Odds ratio | + Sex  + Age  + BMI + Smoking  + Statin + HTN  + DM  = NIHSS score  + Volume  + IVH | Not reporting data of biomarker range and number of patients |
| 138 | Wang et al. (2020)  China | Wang J, Wang W, Liu Y, Zhao X. Associations between levels of high-sensitivity C-Reactive protein and outcome after intracerebral hemorrhage. Front Neurol 2019; 11:535068. Doi: 10.3389/fneur.2020.535068 | Total = 92 | **Inclusion:**  + Patients age> 18 years  + Diagnosed with acute symptomatic ICH by CT scan  + Presented within 24h  + CT within 48h  **Exclusion:**  +Secondary ICH (aneurysm, AVM, infarction, tumor, coagulation)  + Infection  + Surgery | sICH supratentorial infratentorial | 3-mo functional outcome  1-year functional outcome | mRS  + Good (0-2)  + Bad (3-6) | Hs-CRP | Cut-off 3mg/L | Not related to outcome | Biomarkers not reported in mean or range median |
| 139 | Wang et al. (2020)b  China | Wang g, He CJ, Liang XS, ZHoug YF, Chen SH. Potential role of serum substance P as a favorable biomarker of functional outcome in acute spontaneous intracerebral hemorrhage. *Clin Chim Act* 2020; 510:111-6 Doi: 10.1016/j.cca.2020.07.014 | Total = 149 | **Inclusion:**  + Ich patients primary confirmed by CT within 24h Jan 2015 to Jan 2017  + Age >18 year  **Exclusion:**  + Secondary ICH ( AVM, Aneurysm, VST, coagulation, infarction, tumor)  + Severe disease (heart failure, myocardial infarction, unstable angina) + Infection within 4 weeks  + Undergone surgical procedure | sICH supratentorial infratentorial | 90-day functional outcome | mRS  + Good (0-2)  + Bad (3-6) | + Triglyceride  + Choleterol  + LDL  + HDL  + Glucose  + Leukcyte  + CRP  + Susbstance P >449 | Mean (SD)  Dot plot (SP) | + Age  + Sex  + BMI  + Smoking  + Alcohol  + HTN  + DM  + Hyperlipidemia  + Heart failure  + Artery disease  + CKD  + Hematoma volume  + NIHSS  + IVH  + SAH |  |
| 140 | Wei et al. (2014)  China | Wei ZJ, Ou YQ, Li X, Li H. The 90-day prognostic value of copeptin in acute intracerebral hemorrhage. Neurol Sci. 2014;35(11):1673-1679. doi:10.1007/s10072-014-1809-2 | Total = 271 | **Inclusion:**  + Patients admitted within 2010-2012  + Primary ICH  + Admission within 24h  **Exclusion:**  + Previous neurological disease  + Head trauma  + Use of antplatelet or anticoagulant  + Autoimmune  + Systemic disease (uremia, liver cirrhosis, malignancy, heart or lung disease) | sICH supratentorial infratentorial | 3-mo functional outcome  3-mo mortality | mRS  + Good (0-2)  + Bad (3-6) | Plasma copeptin | Box plot | Not stated |  |
| 141 | Whiteley et al. (2009)  UK | Whiteley W, Jackson C, Lewis S, Lowe G, Rumley A, et al. Inflammatory markers and poor outcome after stroke: A prospective cohort study and systematic review of Interleukin-6. Plos ed 2009; 6(9): 31000145 | Total = 844 | **Inclusion:**  + Patients admitted between April 2002-May 2005  **Exclusion:**  + Clinical symptoms >24h + SAH | Ischemic and hemorrhagic stroke | Discharge functional outcome  In hospital mortality | mRS  + Good (0-2)  + Bad (3-6) | + IL-6  + CRP  + Fibrinogen  + WBC + Glucose  + Cholesterol | Med (IQR) | + Age  + Gender  NIHSS  + OSCP ischemic stroke syndrome number  + HTN  + DM  _ Cardiac failure  + AF  + Prior stroke  + Smoking | Included cases of ischemic stroke |
| 142 | Wu et al. (2021)  China | Wu XY, Zhuang YK, Cai Y, Dong XQ, Wang KY, et al. Serum glucose and potassium ratio as a predictive factor for prognosis of acute intracerebral hemorrhage. J Int Med Res 2021; 49(40 1-12. DOi: 10.1177/0300060521100969 | Total = | **Inclusion:**  + Patients admitted between Nov 2018 to Dec 2019  + Primary sICH  + Symptomp <24h  **Exclusion:**  + Age <18 years  + Secondary ICH (trauma, AVM, infarction, tumor)  + Previous troke  + Severe disease such infection within 4weeks  + Autoimmune disease  + Malignancy  + Pregnancy | sICH supratentorial infratentorial | 90-day functional outcome | mRS  + Good (0-2)  + Bad (3-6) | + Glucose  + Pottasium  + Glucose/pottasium ratio | Med (IQR) | + Age  + Gender  + HTN  + DM  + Smoking  + Alcohol  + Site  + IVH  + NIHSS  + GCS  + Hematoma volume  + SAP  + DAP |  |
| 143 | Xiong et al. (2015)  China | Xiong XY, Chen J, Zhu WY, Zhao T, Zhong Q, Zhou K, et la. Serum hepcidin concentrations correlate with serum iron level and outcome in patients with intracerebral hemorrhage. Neurol Sci 2015; 36: 1843-9. Doi: 10.1007/s10072-0150226602 | Total = 86 | **Inclusion**  + Patients with primary ICH  + Admitted within 12h from ictus  **Exclusion**  + Patient age <18 years and 80 years  + Underwent surgery after admission  + In coma or died on admission or within 48h  + Secondary Ich (brain tumor, trauma, drug, coagulopathy, AVM)  + Inflammatory conditions + Iron deficiency anemia  + Hepatopathy  + Took glucocorticosteroids or immunodepressants  + Did not comply with the study protocol | sICH supratentorial | 90-day functional outcome | mRS + Good (0-2) + Bad (3-6) | + Leukocyte  + Glucose  = Platelet  + Hb  + Hepcidin  + Iron  + Il-6  + TNF-a | Med (IQR) | + Age  + Gender  + HTN  + DM + Smoking  + Alcohol  + SBP  + DBP  + NIHSS  + NIHSS 30 day  + GCS baseline  + Basal ganglia  + Edema volume  + ICH growth  + IVH  + Mass effect |  |
| 144 | Yan et al. (2016)  China | Yan XJ, Yu GF, JIe YQ, Fan XF, Huang Q, Dai WM. Role of galectin-3 in plasma as a predictive biomarker of outcome after acute intracerebral hemorrhage. J Neurol Sci 2016; 368:121-7. Doi: 10,1016/j.jns2016.06.071 | Total = | **Inclusion**  + Patients admitted between Sep 2011 to Sep 2014  + Primary sICH 24h from onset  **Exclusion:**  **+** Prior ischemic or hemorrhagic stroke  + Head trauma  _ Use of antiplatelet or anticoagulant medication  + Systemic disease including autoimmune, uremia, liver cirrhosis, malignancy, chronic heart or lung disease  + Infection within 4 weeks  Surgical procedure | Acute sICH basal ganglia | 7- day mortality  6-mo mortality | Mortality | + glucose  + CRP  + Galectin 3 | Odds ratio | + Gender  + Age  + HTN  + DM  + NIHSS  + Volume  + IVH  + END + Admission time  + SAP  + DAP | Biomarkers not reported in mean or median |
| 145 | Yang et al. (2016)  China | Yang G, Hu R, Zhang C, Qian C, Lu QQ, Yung WH, et al. A combination of serum iron, ferritin and transferrin predicts outcome in patients with intracerebral hemorrhage. *Sci Rep* 2016; 6:21970. Doi: 10.1038/srep21970 | Total = 104 | **Inclusion:**  + Age 40-80 years  + Admitted within 24h after onset  **Exclusion:**  **+** Secondary ICH (anticoagulant, aneurysm, AVM, tumor, head trauma, transformation)  + Underlying disease such as anemia and sever liver or renal disease  + Pregnancy | sICH supratentorial infratentorial | 90-day functional outcome | mRS  + Good (0-2)  + Bad (3-6) | + Erytrocyte  + Hb  + Leukocyt  + platelet  + glucose  + Fibrinogen | Mean (SD0 | +Age + Sex  + HTN  + DM  + Heart disease  + History of stroke  + Alcohol  + Smoke habit  + SBP  + DBP  + GCS  + NIHSS  + Ich score  + Location  + Surgery |  |
| 146 | Yang et al. (2016)b  China | Yang G, Shao GF. Elevated serum IL-11, TNF a and VEGF expressions contribute to the patophysiiology of hypertensive intracerebral hemorrhage (HICH). Neurol Sci 2016; 37: 1253-9. Doi: 10.1007/s10072-016-2576-z | Total = | **Inclusion:**  + Newly diagnosed HICH patients during Feb 2013 to Feb 2015  + CT or MRI confirmed cerebral hemorrhage  + Without infectious disease or immune system disease, cancer  + Normal heart, liver, kidney function  + Hospital survivors without combined ischemic stroke or hernia | Hypertensive ICH | Comparison of biomarker between stroke severities | Control, mild, moderate, severe | + IL-11  + TNF-a  + VEGF | Mean (SD) | Not stated | Not reporting outcome of interest |
| 147 | You et al. (2016) | You S, Han Q, Xu J, Zhong C, Zhang Y, Lu H, et al. Serum calcium and phosphate levels and short- and long-term outcomes in acute intracerebral hemorrhage patients. J Stroke Cerebrovas Dis 2016; 25(4): 914-20. Doi: 10,1016/j.jstrokcerebrovasdis.2015.12.023 | Total = | **Inclusion:**  + Patients with ICH admitted within 7 days from symptom onset on Nov 2011 to March 2014  **Exclusion:**  + Secondary ICH (trauma, tumor, transformation, AVM)  + Surgery  + Missing data | sICH supratentorial infratentorial | Discharge functional outcome  3-mo functional outcome | mRS  + Excellent (0-1) | + Serum calcium  + Phosphorus | Cut-off 2.32  Cutoff 1.19 | Not stated | Biomarkers not reported in mean or median |
| 148 | Yu et al. (2014)  China | Yu WH, Wang WH, Dong XQ, Du Q, Yang DB, Shen YF, et al. Prognostic significance of plasma copeptin detection compared with multiple biomarkers in intracerebral hemorrhage. Clin Chim Act 2014; 433: 174-8. Doi: 10.1016/j.cca.2014.03.014 | Total = 118  Control = 118 | **Inclusion:**  + Acute sICH basal ganglia  + Admitted within 6h from symptoms between Jan 2010 and Jan 2013  **Exclusion:**  + Use of antiplatelet or anticoagulant  + Existing previous neurological disease and head trauma  + Presence of other systemic disease including uremia, liver cirrhosis, malignancy, chronic heart disease, chronic lung disease  + undergoing surgical procedure | sICH supratentorial infratentorial | 6-mo functional outcome  6-mo mortality | mRS  + good (0-2)  + Bad (3-6) | + Copeptine  + NSE + S100B  +MBP  + GFAP  + Tau  + pNF-H  + UCH-L1 | Mean (SD) | Not stated |  |
| 149 | Yu et al. (2016)  China | Yu S, Arima H, Heeley E, Delcourt C, Krause M, Peng B et al. White blood cell count and clinical outcomes after intracerebral hemorrhage: the INTERACT2 trial. J Neurol Sci 2016; 361: 112-116. Doi: 10.1016/j.jns.2015.12.033 | Total = 2630 | **Inclusion**  + Patients with sICH within 6h of onset  + Elevated sBP  + Age > 18years  **Exclusion:**  + Contraindication to intensive BP lowering (sever carotid, vertebral, cerebral arterial stenosis, Moya-Moya, etc.)  + Indication to intensive BP lowering  + Evidence of secondary ICH  + Likelihood that the patient will die within next 24h (GCS 3-5)  + Known existing dementia or pre-stroke disability | sICH supratentorial infratentorial | 90-day functional outcome | mRS | WBC | Cut-off  < 6.22  6.24-7.89  7.90-10.17  >10.20 | Not stated | Biomarkers not reported in mean or median |
| 150 | Zhang et al. (2012)  China | Zhang X, Lu XM, Huang LF, Ye H. Copeptin is associated with one-year mortality and functional outcome in patients with acute spontaneous basal ganglia hemorrhage. *Peptides* 2021; 33:336-41. Doi: 10.1016/j.peptides.2012.01.011 | Total = 89 | **Inclusion:**  + Patients with spontaneous basal ganglia hemorrhage Jul 2007-Dec 2009  + Admitted within 6h from onset  **Exclusion**  + Existing previous neurological disease  + Head trauma  + use of antiplatelet  + Presence of other prior systemic disease including uremia, liver cirrhosis, malignancy, chronic heart or lung disease  + Surgical procedure | sICH basal ganglia | 1 year functional outcome  1 year mortality | mRS  + Good (0-2)  + Bad (3-6) | + Glucose  + CRP  + D-dimer  + Copeptin | Mena (SD) | + Gender  + Age  + HTN  + DM  + NIHSS  + Volume  + IVH  + Hemorrhage growth + END  + SBP  + DAP |  |
| 151 | Zhang et al. (2012)b  China | Zhang A, Li J, Li X, Song L, Li H. The prognostic value of copeptin for acute intracerebral hemorrhage patients. *Exp Ther med* 2013; 5: 467-70 . Doi:10.3892/etm.2012.804 | Total = 120 | **Inclusion:**  + Patients with acute intracerebral hemorrhage | ICH acute | 90-day functional outcome  90-day mortality | mRS  + Good (0-2)  + Bad (3-6) | + Glucose  + WBC  + Copeptin | Mean (SD) | + Hematoma volume  + GCS score  + Hemphil score | No information on exclusion criteria |
| 152 | Zhang et al. (2013)  China | Zhang X, Lu XM, Huang LF, Li X. Prognostic value of leptin: 6-month outcome in patients with intracerebral hemorrhage. *Peptides* 2013; 43: 133-6. Doi: 10,1016/j.peptides.2013.03.010 | Total = 92 | **Inclusion**  + Patienst with sICH basal ganglia during Jan 2010-March 2012  + Admitted within 6h of symtomps onset  **Exclusion:**  + Existing previous neurological disease  + Head trauma  + Antiplatelet or anticoagulant  + Presence of other prior systemic disease including uremia, liver cirrhosis, malignangcy, and chronic heart disease  + Underwent surgical procedure | sICH basal ganglia | 6-mo mortality  6-mo functional outcome | Mortality  mRS  + Good (0-2)  + Bad (3-6) | + Glucose  + CRP  + D-dimer  + Leptin | Mean (SD) | + Gender  + Age  + BMI  + HTN  + DM  + NIHSS score  + Hematoma volume  + IVH  + Hemorrhage growth  + END  + SAP  + DAP |  |
| 153 | Zhang et al. (2015)  China | Zhang ZL, Liu YG, Huang QB, Wang hW, Song Y, XU ZK, Li F. Nuclear factor-kB activation in perihematomal brain tissue correlates with outcome in patients with intracerebral hemorrhage. *J Neuroinflam* 2015; 12:53. Doi: 10.1186/s12974-015-0277-9 | Total = 45 | **Inclusion:**  + Patients with sICh basal ganglia  + Admitted between Oct 2011 to Aug 2013  + Time from symptom onset 6-12 h  + hematoma volume 30-90 mL  + Hematoma evacuation conducted along non-functional cortex  **Exclusion**  + rebleeding  + Secondary ICH (head trauma, aneurysm, vascular malformation, hemorrhagic infarction, cerebral vein and sinus thrombosis, tumor, anticoagulant, blood thinners, coagulopathy-related hemorrhage)  + history of bleeding, inflammation, trauma, surgery  + Use of drugs that affect the immune system  + presence of underlying disease within previous month | sICH supratentorial basal ganglia | 6-mo functional outcome | mRS  + Good (0-3)  + Bad (4-6) | NF-kB activation | Mean (SD) | + Gender  + Age  + GCS score on admission + Smoking  + Alcohol  + HTN  + DM  + Coronary heart disease  + COPD + hematoma volume  + IVH  + HC  + Midline shift  + Brain edema  + MV  + Pneumonia | Biomarker not reported in other studies |
| 154 | Zhang et al. (2018)  China | Zhang YB, Zheng SF, Yao PS, Chen GR, Li GH, Li Sc, et al. Lower ionized calcium predicts hematoma expansion and poor outcome in patients with hypertensive intracerebral hemorrhage. *World Neurosurg* 2018; E1-5. Doi: 10.1016/j.wneu.2018.06.223 | Total = | **Inclusion:**  + Patients with hypertensive ICH between Jan 2015-Dec 2017  + CT within 6h of onset, follow-up within 24h  **Exclusion:**  + HICH >30mL  + Evacuation of hematoma without preoperative follow-up CT scan  + Hemorrhage by brain infarction, AVM, tumor  + Antiplatelt or anticoagulant Ich | Hypertensive ICH | 3-mo functional outcome | mRS  + Good (0-3)  + Bad (4-6) | + Total calcium  + ionized calcium  + PLT  + PT  + aPTT  + INR | Med (IQR) | + Age  + Sex  + Smoking  = DM  + SBP  + DBP  + location |  |
| 155 | Zhang et al. (2018)b  China | Zhang F, Qian J, tao C, Wang Y, Lin S, You C, Yang W. Neutrophil to lymphocyte ratio predicts island sign in patients with intracranial hemorrhage. *Medicine* 2018; 97(44): e13057. | Total = | **Inclusion:**  + patients with Ich admitted between Sep 2014 to Oct 2016  + Ich by CT 6h  + ab within 24h  + age >18 years  **Exclusion** + Secondary ICH  + Infection in 2 weeks  + Patients with systemic diseses including neoplasm, COPD, autoimmune, heart disease, uremia, severe renal dysnfunction, immunosuppressant  _ Hematoma evacuation before follow-up CT | sICH infratentorial supratentorial | Hematoma expansion  Island sign | Imaging | + PT  + aPTT  + INR  + WBC + ANC  + AMC  + NLR | Mena (SD) | + Sex  + Age  + SAP  + DAP  + Mean arterial pressure  + HTN  + DM  + Ischemic stroke  + GCS score  + Hematoma size  + HC  + IVH  + Loacation | Not reporting outcome of interest |
| 156 | Zhang et al. (2018)c  China | Zhang W, Shen Y. Platelet-to-lymphocyte ratio as a new predictive index of neurological outcomes in patients with acute intracranial hemorrhage: A retrospective study. *Med Sci Monit* 2018; 24:4413-20. Doi: 10.12659/MSM/910845 | Total = 107 | **Inclusion:**  + Patients with acute ICH admitted to ICU after intracranial surgery from Jan 2016 to June 2017  + Age >18 years  + GCS > 4 and <12 on admission  + emergency intracranial surgery within 24h after onset  **Exclusion:**  + Pregnant  + Lacked sufficient info  + Aneurysmal SAH | All cause sICH | GCS at hospital discharge  Functional outcome at 6-mo | GCS + 3-8  + 9-12 _ 13-15  mRS  + Good (0-2)  + Bad (3-6) | + Platelet  + Lymphocyte  + PLR  + CRP | Mean (SD) | + Age  + Gender  + Alcohol  + Smoking  + Cardiac  + Kidney | Included cases of traumatic hemorrhage and systemic comorbidities |
| 157 | Zhang et al. (2019)  China | Zhang F, Ren Y, Shi Y, Fu W, Tao C, et al. Predictive ability of admission neutrophil to lymphocyte ratio on short-term outcome in patients with spontaneous cerebrellar hemorrhage. *Medicine* 2018; 98(25): e16120 | Total = 107 | **Inclusion:**  **+** Patients with sICH cerebellar  + Blood examination within 24h  + Age > 18 years  **Exclusion:**  + Secondary ICH to aneurysm, AVM, trauma, brain tumor  + Chronic infection  + Systemic inflammation by severe systemic diseases including autoimmune disease, lung cancer, chronic heart disease, COPD, uremia, severe renal dysfunction, pneumonia, acute or chronic infectious disease  + Medication such as immunosuppressant, immunotherapy, anticoagulants  + Ischemia or hemorrhagic stroke happened within 6 mo | sICH cerebellar | 30-day functional outcome | GOS  + Good (4-5)  + Bad (0-3) | + WBC  + ANC + ALC + AMC + NLR | Mean (SD) | + Gender  + Age  + SBP  + DBP  + HTN  + DM  + Ischemic stroke  + Smoker  + Alcohol  + GCS on admission  + Hematoma size  + SAH  + IVH  + hematoma site  + Brainstem compression  + 4^th^ ventricle compression + HC |  |
| 158 | Zhang et al. (2019)b  China | Zhang F, Ren Y, Fu W, et al. Predictive Accuracy of Neutrophil-to-Lymphocyte Ratio on Long-Term Outcome in Patients with Spontaneous Intracerebral Hemorrhage. World Neurosurg. 2019;125:e651-e657. doi:10.1016/j.wneu.2019.01.143 | Total = | **Inclusion:**  + Patients with sICh from Oct 2013 to May 2017  + CT within 6H  + Follow up CT during 24h  **Exclusion:**  + Secondary Ich of tumor, aneurysm, trauma, AVM, moya-moya disease  + Hematoma evacuation or surgical approach was performed | sICH supratentorial infratentorial | 6-mo functional outcome  6-mo mortality | GOS  + Good (4-5)  + Bad (0-3) | + WBC + ANC  + AMC  + NLR  = PT  + aPTT + INR | Mean (SD) | + Sex  + Age  + SBP  + DBP  + HTN  + DM  + Ischemic stroke  + Smoker  + Alcohol  + GCs on admission  + Hematoma size  + SAH  + IVH  + HC + Supratentorial |  |
| 159 | Zhang et al. (2020)  China | Zhang GH, Ye ZH, Guan HJ, Guo M, Zhou XX, Xu YY. Impact of serum omentin-1 concentrations on functional outcome among acute intracerebral hemorrhage patients. *Clinica Chimica Acta* 2020; 503:169-74 | Total = | **Inclusion:**  + Primari sICH  + Admitted within 24h after symptom onset of stroke  **Exclusion:**  + Age< 18 y  + Bleeding due to underlyin vascular lesion, AVM, infarction, tumor  + Surgical procedure  + Other disease such as autoimmune, severe infection, pregnancy or known malignancy | sICh supratentorial infratentorial | 90-day functional outcome | mRS  + Good (0-2)  + Poor (3-6) | + Glucose  + CRP  + WBC  + Omentin-1 | Med (IQR)  Cut-off 147.9 | + Gender  + Age  + BMI  + Smoking  + Drinking  + HTN  + DM  + Hyperlipidemia  + Heart failure  + Artery disease  + CKD  + Antiplatelet  + Anticoagulat  + GCS  + ICH  + Hematoma volume  + SAH  = IVH  + SAP  + DAP | Included patients with prior systemic disease |
| 160 | Zhou et al. (2010)  China | Zhou Y, Xion KL, Lin S, Zhong Q, Lu FL, Liang H, et al. Elevation of high-mobility group protein box-1 in serum correlates with severity of acute intracerebral hemorrhage. *Mediat Inflam* 2010. Doi: 10.1155/2010.142458 | Total = | **Inclusion:**  + patients with aICh from Jan 2009 to March 2010  **Exclusion:**  + Age<18 years  + Surgery after admission  + In coma or died within 48h  + hemorrhage due to brain tumor, trauma, drug abuse, coagulation, vascular malformation  + Obvisous inflammatory conditions (infectious disease, SE, thematoid arthritis) within 6 mo  + Acute myocardia infarction  + Acute ischemia of liver  + Autoimmune disase + GLucocorticosteroids or immunodepressants | sICh infatentorial supratentorial | 3-mo functional outcome | mRS  + Good (0-2)  + Poor (3-6) | + Glucose  + Platelet  + Leukocyte  + Fibrinogen + Il-6  + TNF-a | Med (IQR) | +Age  + Sex  + HTN  + Diabetes + Smoking  + Alcohol  + SBP  + DBP  + gCS  + NIHSS  + Ich volume  + Edema volume  + ICH growth at 72 h  + ICH growth 7days  + IVH  + Mass effect |  |
| 161 | Zhou et a. (2016)  China | Zhou S, Bao J, Wang y, Pan S. S100b as biomarker for differential diagnosis of intracerebral hemorrhage and ischemic stroke. *Neurol Res* 2016. Doi: 10.1080/01616412.2016.1152675 | Total = | **Inclusion:**  + Patients from June 104 to Jul 2015 admitted to emergency department for acute stroke symptoms within the first 6h  **Exclusion:**  + Previous acute stroke within 3 mo  + Previous brain injury, head truma, other neurological disease | ICH and IS | Comparison of Ich and IS |  | S100b | Mean(SD) | + Age  + Gender  + HTN  + Diabetes  + NIHSSS  + Crdio-embolic  + Small vessel  + Large vessen  + Infarction size  + Posterior circulation  + Hemorrhage location | Not reporting outcome of interest |
| 162 | Zhou et al. (2020)  China | Zhou Q, Zhang D, Chen X, Yang Z, Liu Z, Wei B, et al. Plasma D-dimer predicts poor outcome and mortality after spontaneous intracerebral hemorrhage. *Brain Behav* 2020. Doi: 10.1002/brb3.1946 | Total = 1,322 | **Inclusion:**  + Age > 18 years  + Presented with sICH between Jan 2016 and Dec 2016  **Exclusion:**  + Ich to trauma, hemorrhagic conversion of ischemic stroke, structural lesion, vascular malformation | sICH supratentorial infratentorial | 90-day functional outcome  90-day mortality | mRS  + Good (0-2)  + Bad (3-6) | Glusoe  INR  PTaPTT  Fibrinogen  D=dimer | Odds ratio | + Age  + Sex  + HTN  + DM  + BP  + Hear rate  + Temp  + GCS + NIHSS = Anticoagulant  = Antiplatelet  + Initial hematoma volum  + IVH | Not reporting data of biomarker range and number of patients |
| 163 | Zweifel et al. (2010)  Switzerland | Zweifel C, Katan M, Schuetz P, Siegemund M, Morgenthaler NG, Merlo A, et al. Copeptin is associated with mortality and outcome in patients with acute intracerebral hemorrhage. BMC Neurol 2010; 10:34 | **Total =** 40 | **Inclusion:**  + Admitted within Nov 2006-nov 2007  + Admitted with sICH within 72h of symptom  **Exclusion:**  + Trauma  + SAH | sICH hypertensive and coagulopathy (9) | 30-day mortality  90-day functional outcome | Mortality  mRS  + good (0-2)  + Bad (3-6) | + Sodium  + Osmolarity  + Glucose  + WBC  + CRP  + Copeptin | Median (IQR) | + Age  + Gender  + GCS  + Charlson index  + Temperature  + hematoma volume  + HTN  + Antiplatelets  + ICH score  + Location  + IVH  + Surgery |  |
| 164 | Alsamani et al. (2022)  China | Alsamani R, Limin Z, Jianwei W, et al.. Predictive value of the apolipoprotein B/A1 ratio in intracerebral hemorrhage outcomes. *J Clin Lab Anal*. 2022;36:e24562. doi: 10.1002/jcla.24562 | **Total: 2**16 | **Inclusion:**  + ICH patients > 18 years  **Exclusion:**  + Patients with ICH attributed to trauma, tumor, hemorrhagic transformation of ischemic stroke, aneurysm, cerebral vascular malformation  + Patients with hepatic renal disease  + Impaired coagulation  + Sepsis  + Patients with ICH induced by cerebral amyloid angiopathy  + Presentation since clinical onset of stroke >24h  + Did not take initial NCCT within 24h  Did not take follow-up NCCT within 48h  + No follow-up  + No apo A1 apoB record | sICH  All location | 1-year mortality  1-year functional outcome | Mortality  mRS  + good (0-2)  + Bad (3-6) | ApoB/apoA1 ratio | Cutoff 0.80 |  | Not reporting data of biomarker range and number of patients |
| 165 | Babu et al. (2022)  India | Babu S, Pulicken M, Thazhathuveedu AK. Peripheral Blood Neutrophil-to-lymphocyte Ratio as a Predictor of Functional Outcomes in Patients with Hemorrhagic Stroke. Indian J Crit Care Med 2022;26(1):18–22 | **Total** 158 patients | **Inclusion:**  + Patients >18 years  + Presented to emergency department within 24h of onset of stroke symptoms  + Evidence of hemorrhagic stroke in CT brain  **Exclusion:**  + Patients with a history of trauma, fever, prior stroke, or current anticoagulant medications | Not stated | 90-day functional outcome | mRS  + good (0-2)  + Bad (3-6) | Neutrophil to lymphocyte ratio | ROC Curve |  | Aetiology of hemorrhagic stroke was not stated |
| 166 | Bader et al. (2022)  USA | Bader ER, Pana TA, Barlas RS, Metcalf AK, Potter JF, Myint PK. Elevated inflammatory biomarkers and poor outcomes in intracerebral hemorrhage. *J Neurol*. 2022;269(12):6330-6341. doi:10.1007/s00415-022-11284-8 | **Total** 1,714 patients | **Inclusion:**  + Primary intracerebral haemorrhage cases  **Exclusion:**  + First CRP measurement after discharge  + Firsth WCC measurement after discharge  + Implausible white cell count  + Missing OCSP, mRS, CRP, pre-ICH mRS data | Not stated | 90-day mortality  90-day functional outcome | Mortality  mRS  + good (0-2)  + Bad (3-6) | Elevated inflammatory biomarker | Number of patients with elevated inflammatory biomarker | + Age  + Sex  + Comorbidities | Not reporting data of biomarker range |
| 167 | Bender et al. (2021)  USA | Bender M, Naumann T, Uhl E, Stein M. Early Serum Biomarkers for Intensive Care Unit Treatment within the First 24 Hours in Patients with Intracerebral Hemorrhage. *J Neurol Surg A Cent Eur Neurosurg*. 2021;82(2):138-146. doi:10.1055/s-0040-1716516 | **Total**  329 patients | **Inclusion:**  + Age > 18 years  + Diagnosis of sICH  + Presented to ED Feb 2008 – Jan 2017  + Cardiopulmonary biomarker TNI and/or CV were determined on admission  **Exclusion:**  + Patients with acute cardiac decompensation to coronary artery disease  + Acute pulmonary decompensation  + Long term cortisol treatment  + ICH due to vascular malformation, malignancy, trauma | sICH primary  All location | Intrahospital mortality | Mortality | CRP  WBC count  Hemoglobin  Hematocrit  Cholinesterase  Glucose  Lactate  Albumin  Troponin I  Cortisol | Mean (+SD) | + Cardiopulmonary parameter  + Treatment  + Surgery  + ICH volume  + IVH  + Hydrocephalus  + Age  + Sex  + BMI  + Admission GCS  + Chronic arterial hypertension  + Pulmonarydisease  + Renal insufficiency | Not reporting defined time for outcome assessment |
| 168 | Bender et al. (2022)  USA | Bender M, Haferkorn K, Nagl J, Uhl E, Stein M. Serum Lactate as Serum Biomarker for Cardiopulmonary Parameters within the First 24 Hours after a Spontaneous Intracerebral Hemorrhage. *Diagnostics (Basel)*. 2022;12(10):2414. Published 2022 Oct 5. doi:10.3390/diagnostics12102414 | **Total**  **354** | **Inclusion**  + Patients with sICH  + Age > 18 years  + Treated for 24h at ICU  **Exclusion:**  + Acute cardiac decompensatin  + Cardiopulmonary reanimation  + Acute pulmonary decompensation  + Liver failure  + ICH due to trauma, neoplasia, vascular malformation | sICH non traumatic, neoplasia, vascular malformation | mRS at discharge  Mortality | mRS  + 0-4 favorable  + 5-6 unfavorable | Lactate positive  Lactate negative |  |  | Not reporting data of biomarker range |
| 169 | Bender et al. (2023)  USA | Bender M, Haferkorn K, Tajmiri-Gondai S, Stein M, Uhl E. Serum Urea-to-Albumin Ratio Is an Independent Predictor of Intra-Hospital Mortality in Neurosurgical Intensive Care Unit Patients with Spontaneous Intracerebral Hemorrhage. *J Clin Med*. 2023;12(10):3538. Published 2023 May 18. doi:10.3390/jcm12103538 | **Total**  354 | **Inclusion**  + Patients with sICH  + Age > 18 years  + Treated for 24h at ICU  **Exclusion:**  + Acute cardiac decompensatin  + Cardiopulmonary reanimation  + Acute pulmonary decompensation  + Liver failure  + ICH due to trauma, neoplasia, vascular malformation | sICH non traumatic, neoplasia, vascular malformation | Intrahospital mortality | Mortality | + WBC  + Hemoglobin  + Hematocrit  + Cholinesterase  + Glucose  + Lactate  + Cortisol  + CRP  + Creatinine  + Urea  + Albumin  + Prothrombin time  + Partial thromboplastin time  + Antithrombin III | Mean (SD) | + Age  + Sex  + BMI  + Initial GCS  + Hypertension  + COPD  + Cardiac arrhytmia  + Heart fallure  + DM  + Location  + Medical treatment  + Surgery  + IVH  + Hydrocephalus | Not reporting defined time for outcome assessment |
| 170 | Bian et al. (2019)  China | Bian L, Mao LG, Sun Y, et al. Serum lipoprotein-associated phospholipase A2 as a promising prognostic biomarker in association with 90-day outcome of acute intracerebral hemorrhage. *Clin Chim Acta*. 2019;495:429-435. doi:10.1016/j.cca.2019.05.017 | **Total**  164 patients | **Inclusion:**  + Acute spontaneous basal ganglia hemorrhage  + Admission within 24h  **Exclusion:**  + <18 years  + Undrlying vascular lesion  + Coagulative dysfuction  + Hemorrhagic infarction  + Tumoral bleeding  + Infection within 4 weeks  + Neurological diseases  + Underwet surgical procedure | sICH basal ganglia | 90-day functional outcome  NIHSS score  Hematoma volume | mRS  0-2 good  3-6 poor | Lp-PLA2 level  Glucose  CRP  WBC | Cut off 303.6 ng/mL  Median (range) | + Age  + Sex  + Smoking  + Alcohol  +Hypertension  + DM  + Hyperlipidemia  + Hematoma vlume  + IVH |  |
| 171 | Brea et al. (2009)  Spain | Brea D, Sobrino T, Blanco M, et al. Temporal profile and clinical significance of serum neuron-specific enolase and S100 in ischemic and hemorrhagic stroke. *Clin Chem Lab Med*. 2009;47(12):1513-1518. doi:10.1515/CCLM.2009.337 | **Total**  224 ischemic  44 hemorrhagic | **Inclusion:**  + First time stroke  + Onset of stroke <12h  + Previously independent  **Exclusion:**  + Chronic inflammatory disease  + Severe hepatic or renal disase  + Hematological disease  + Cancer  + Infectious disease within 15 days prior to admission | Ischemic and hemorrhagic stroke | 90-day functional outcome | mRS  0-2 good  3-6 poor | NSE serum at admission, 24h, 72h  S100 serum at admission, 24h, 72h | Median (range) | Not stated | Not reporting etiology of hemorrhagic stroke |
| 172 | Cai et al. (2013)  China | Cai JY, Lu C, Chen MH, et al. Predictive value of phosphorylated axonal neurofilament subunit H for clinical outcome in patients with acute intracerebral hemorrhage. *Clin Chim Acta*. 2013;424:182-186. doi:10.1016/j.cca.2013.06.019 | **Total** | **Inclusion** + Spontaneous basal ganglia hemorrhage  Exclusion:  + Prev neurologica disease  + Head trauma  + Use of antiplatelet or anticoagulant medication  + Prior systemic disease  + Surgical hematoma evacuation  + Unavailable pNF-H | Spontaneous basal ganglia stroke | 6-month functional outcome  Early neurological deterioration | mRS  0-2 good  3-6 poor  Increase > 4 points NIHSS score within 24h | + Glucose  + CRP  + D-Dimer  + WBC  + Hemoglobin  + Platelet count  + Prothrombin time  + Thrombin time  + Partial thrmoboplastin time  + Fibrinogen  + pNFH | Mean (SD) | + Age  + Sex  + Hypertension  + Diabetes mellitus  + NIHSS score  + Hematoma volume  + IVH  + Hemorrhage growth  + END |  |
| 173 | Chen et al. (2018)  China | Chen B, Shen J, Zheng GR, et al. Serum cyclophilin A concentrations and prognosis of acute intracerebral hemorrhage. *Clin Chim Acta*. 2018;486:162-167. doi:10.1016/j.cca.2018.08.002 | **Total**  141 | **Inclusion**  + Patients with first acute spontaneous basal ganglia hemorrhage  **Exclusion** + Admission > 24h  + Unavailable follow-up CT  + Infection within recent a month  + Secondary cause ICH (tumor, vascular malformation, trauma)  + Other neurological disease  + Use of antiplatelet or anticoagulant medication  + Surgical procedure  + Other concurrent comorbidities: autoimmune diasease, uremia, liver cirrhosis, malignancy, chronic heart, lung disase | sICH no traumatic, vascular, neoplasia | 6-mo functional outcome | mRS  0-2 good  3-6 poor | Serum cyclophilin A level | Box plot | Not stated | Biomarker is not reported in other studies |
| 174 | Chen et al. (2023)  China | Chen K, Huang W, Wang J, et al. Increased serum fibroblast growth factor 21 levels are associated with adverse clinical outcomes after intracerebral hemorrhage. Front Neurosci. 2023;17:1117057. Published 2023 May 5. doi:10.3389/fnins.2023.1117057 | **Total** | **Inclusion:**  + Patients with stroke  + Presented within 24h of onset  **Exclusion:**  + Ischemics troke  + Transient ischemic attack  + Aneurysm  + traumatic hemorrhage  + Hemorhagic transformation  + Neoplasia  + No laboratory data  + No follow-up | sICH all location | 90-day functional outcome | mRS score  0-2 good  3-6 poor | FGF21 level | Quartile and mRS distribution | Not stated | Not reporting data of biomarker range |
| 175 | Chu et al. (2023)  China | Chu H, Huang C, Zhou Z, Tang Y, Dong Q, Guo Q. Inflammatory score predicts early hematoma expansion and poor outcomes in patients with intracerebral hemorrhage. Int J Surg. 2023;109(3):266-276. Published 2023 Mar 1. doi:10.1097/JS9.0000000000000191 | **Total**  301 development  154 validation | **Inclusion**  + Age > 18 years  + Admitted to stroke unit within 24h  + NCCT performed within 24h  **Exclusion:**  + Secondary ICH (cerebral aneurysm, Moyamoya, arteriovenous malformation, tumor, trauma, hemorrhagic transformation)  + Primary IVH  + Historical mRS > 1  + Refused to enroll | sICH all location | Secondary neurological deterioration  30-day mortality  3-mo functional outcome | GCS decerase >3 points within 48h  Mortality  mRS  0-3 good  4-6 poor | Inflammatory score | Box plot, number of cohort with known score | Not stated | Not reporting data of biomarker range |
| 176 | Daubail et al. (2012) France | Daubail B, Jacquin A, Guilland JC, et al. Serum 25-hydroxyvitamin D predicts severity and prognosis in stroke patients. Eur J Neurol. 2013;20(1):57-61. doi:10.1111/j.1468-1331.2012.03758.x | **Total**  382 patients | **Inclusion:**  + Patients with ischaemic or hemorrhagic stroke | Ischemic and hemorrhagic stroke | Functional outcome at discharge | NIHSS  Rankin  0-2 good  3-6 poor | Serum vitamin D | Cut off 25.7nM | Not stated | Included ischemic stroke patients |
| 177 | Delgado et al. (2008)  Spain | Delgado P, Cuadrado E, Rosell A, et al. Fas system activation in perihematomal areas after spontaneous intracerebral hemorrhage. Stroke. 2008;39(6):1730-1734. doi:10.1161/STROKEAHA.107.500876 | **Total**  78 patients | **Inclusion:**  + Spontaneous supratentorial ICH  + Admitted within the first 24h  **Exclusion:**  + Patients with ICH related to vascular malformation, coagulation  + oral anticoagulant intake  + Traumatic brain injury  + Hemorrhagic infarction  + Tumoral bleeding  + Underwent surgical procedure | sICH all site | Neurological status (admission, 24h, 48h, 7 days, 3mo)  Mortality  Functional outcome | NIHSS  Mortality  mRS  + 0-2 good  + 3-6 poor | Soluble Fas | Dot plot towards hematoma growth | Not stated | No biomarker data for the reported outcomes |
| 178 | Feng et al. (2021)  China | Feng H, Wang X, Wang W, Zhao X. Lipid Levels and 3-Month Prognosis After Spontaneous Intracerebral Hemorrhage in Women. Front Neurol. 2021;12:690194. Published 2021 Jun 17. doi:10.3389/fneur.2021.690194 | **Total**  206 patients | **Inclusion:**  + Female whose time from onset of hemorrhage to enrolment was not more than 24h  + TG, TC, LDLC, HDLC, non-HDLC were documented in the database  + > 18 years  **Exclusion:**  + Primary ventricular hemorrhage  + Diagnosis of secondary ICH (head trauma, brain tumor, aneurysm, hemangioma, arteriovenous malformation)  + Surgical intervention (EVD, craniotomy, hematoma puncture, aspiration)  + Anticoagulat therapy befor onset of symptoms | sICH all site | 3-mo functional outcome | mRS  + 0-2 good  + 3-6 poor | + Glucose  + WBC  + Platelets  + INR  + Creatinine  + In hospital TG  + In hospital TC  + In hospital LDLC  + In hospital HDLC  + In hospital non-HDLC | Median (range) | + Age  + Hypertension  + DM  + Hyperlipidemia  + Smoking  + Alcohol  + Antiplatelet use  + Statin use  + NIHSS score on admission  + GCS Score on admission  + Location  + IVH |  |
| 179 | Garton et al. (2017) USA | Garton ALA, Gupta VP, Christophe BR, Connolly ES Jr. Biomarkers of Functional Outcome in Intracerebral Hemorrhage: Interplay between Clinical Metrics, CD163, and Ferritin. J Stroke Cerebrovasc Dis. 2017;26(8):1712-1720. doi:10.1016/j.jstrokecerebrovasdis.2017.03.035 | **Total**  41 patients | **Inclusion:**  + Serum sample collected on both day 1 and day 7  + Serum samples <5 years  + Follow-up data at 3 and 12 mo post discharge  + Non traumatic ICH  **Exclusion:** Not stated | Nontraumatic ICH | 3-mo functional outcome  12-mo functional outcome | mRS score | + CD163 day 1  + CD163 day 7  + Ferritin day 1  + Ferritin day 7  + Hepcidin day 1  + Hepcidin day 7 | Correlation to clinical metrics | Not stated | Not reporting data of biomarker range |
| 180 | Gendron et al. (2020)  USA | Gendron TF, Badi MK, Heckman MG, et al. Plasma neurofilament light predicts mortality in patients with stroke. Sci Transl Med. 2020;12(569):eaay1913. doi:10.1126/scitranslmed.aay1913 | **Total**  ACI 227  aSAH 58  ICH 29 | **Inclusion:**  + Stroke patients whom plasma was collected | All stroke aetiology | + Neurological outcome  + Functional outcome  + Cognitive status | + NIHSS  + mRS  + Mini Mental State Examination | + Plasma NFL | Association with mRS score | Not stated | No biomarker data for the reported outcomes |
| 181 | Godoy et al. (2010)  Argentina | Godoy DA, Papa F, Campi V, del Valle M, Piñero G, et al. (2010) Relationship between Baseline White Blood Cell and C-Reactive Protein with Mortality in Patients with Spontaneous Intracerebral Hemorrhage. J Neurol Neurophysiol 1:104. doi:10.4172/2155-9562.1000104 | **Total:** 175 | **Inclusion:**  + Patients admitted to ICU within 24h after onset  **Exclusion:**  + Pregnant  + Hemorrhage secondary to brain tumor  + Trauma  + Hemorrhagic transformation of cerebral infarct  + Aneurysm or vascular malformation + First evaluation >24h after symptom onset  + Referral from another hospital after diagnosis and initial evaluation  + Prior infection within 4 weeks  + Concurrent major renal or hepatic disase  + Cancer  + Acute inflammatory disease  + Surgery or major trauma in previous month | sICH | 30-day Mortality  30-day Functional outcome | Mortality  GOS + 4-5 good  + 2-3 poor | + Plasma glucose  + WBC  + CRP | Median (IQR) | + Age  + Sex  + HTN  + DM  + Alcohol  + Smoking  + Hypercholesterolemia  + GCS admission + Location  + Volume  + Midline shift  + IVH  + Graeb score  + Hydrocephalus |  |
| 182 | Gregorio et al. (2019)  Portugal | Gregorio T, Albuquerque I, Neves V, et al. NT-pro-BNP correlates with disease severity and predicts outcome in cerebral haemorrhage patients: Cohort study. J Neurol Sci. 2019;399:51-56. doi:10.1016/j.jns.2019.02.014 | **Total** 201 | **Inclusion**  + Previously independent (mRS <2)  + Age > 18 years  + non-comatose  + Not medically trated  + Non traumatic ICH  **Exclusion:**  + Previous cerebral hemorrhage  + cerebral haemorrhage secondary to trauma,  thrombolysis or cerebral angiographic procedures, cerebral haemorrhage secondary to  haemorrhagic transformation of arterial or venous brain infarcts, brain tumour associated ICH  + ICH occurring after hospital admission for another diagnosis  + patients transferred to/from another institution | sICH all location | Discharge mortality  3-mo functional outcome | Mortality  mRS  + 0-2 good  + 3-6 poor | NT-pro-BNP  Glucose  Creatinine clearance | Correlation to mRS | + GCS Score  + Hypertension  + DM  + Chronic heart disase  + Anticoagulant  + Antiplatelet  + Hematoma size  + Location | Not reporting data of biomarker range |
| 183 | Glyndenholm et al. (2022)  Denmark | Gyldenholm T, Hvas CL, Hvas AM, Hviid CVB. Serum glial fibrillary acidic protein (GFAP) predicts outcome after intracerebral and subarachnoid hemorrhage. Neurol Sci. 2022;43(10):6011-6019. doi:10.1007/s10072-022-06274-7 | **Total** 40 samples | **Inclusion:**  + Adult patients with MRI or CT sith sICH or aSAH  **Exclusion:**  + Pregnancy  + Antithrombotic medication  + Active cancer  + Tumor  + Chemotherapy within 3 mo  + Bleeding disorder  + Liver cirrhosis  + Hemosiderosis  + Antibiotic treatment at time of admission  + ICH due to arterio-venous malformation, trauma, perimesencephalic bleeding | sICH and aSAH | 30-day functional outcome | mRS score  0-3 good  4-6 poor | GFAP level  Neuroglobin level | Mean (SD) | Not stated |  |
| 184 | He et al. (2023) | He J, Zhang Y, Li T, et al. Glucose-albumin ratio as new biomarker for predicting mortality after intracerebral hemorrhage. Neurosurg Rev. 2023;46(1):94. Published 2023 Apr 19. doi:10.1007/s10143-023-02002-7 | **Total** | **Inclusion**  + Diagnosed with ICH  **Exclusion** + Prior history of ICH  + ICH due to vascular malformation, aneurysm, head trauma, hemorrhagic infarction, tumoral hemorrhage  + Lack of neutrophil, lymphocyte albumin, platelet, glucose data  + household registration were not found in the Household Registration Administration System of Sichuan province | All sICH | 90-day mortality | Mortality | + Glucose  + Neutrophil  + Platelet  + Lymphocyte  + Albumin | ROC curve to 90-day mortality | Not stated | Biomarkers not reported in mean or range median |
| 185 | Howe et al. (2019)  USA | Howe MD, Furr JW, Zhu L, Edwards NJ, McCullough LD, Gonzales NR. Sex-specific Association of Matrix Metalloproteinases with Secondary Injury and Outcomes after Intracerebral Hemorrhage. J Stroke Cerebrovasc Dis. 2019;28(6):1718-1725. doi:10.1016/j.jstrokecerebrovasdis.2019.02.014 | **Total** 55 | **Inclusion**  + Hypertensive hemorrhagic injury  **Exclusion:**  + Hemorrhagic due to traumatic brain injury, cerebral amyloid, angiopathy, vascular lesion | Basal ganglia and thalamus sICH | 90-day functional outcome  IVH score  Hematoma volume | mRS | MMP-2  MMP-9  MMP-1  MMP-3 | Correlation | Not stated | Biomarkers not reported in mean or range median |
| 186 | Hu et al. (2012)  China | Hu HT, Xiao F, Yan YQ, Wen SQ, Zhang L. The prognostic value of serum tau in patients with intracerebral hemorrhage. Clin Biochem. 2012;45(16-17):1320-1324. doi:10.1016/j.clinbiochem.2012.06.003 | **Total** 110 patients | **Inclusion:**  + Patients with acute spontaneous basal ganglia hemorrhage  + Evaluated within 6h  **Exclusion**  + Previous neurological disase  + Head trauma  + Presence of other prior systemic diseases (uremia, liver cirrhosis, malignancy, chronic heart or lung disease)  + surgical hematoma evacuation | Basal ganglia hemorrhage | Early neurological deterioration  3-mo functional outcome | NIHSS  mRS  +0-2 good  +3-6 poor | CRP  D-Dimer  Serum tau  WBC  Hemoglobin  Platelet  Prothrombin time  Thrombin time  Partial thromboplastin time  Fibrinogen | Mean (SD) | + Age  + Gender  + Hypertension  + DM  + NIHSS score  + Hematoma volume  + IVH  + Hemorrhage growth  + END |  |
| 187 | Huang et al. (2013)  China | Huang Q, Dai WM, Jie YQ, Yu GF, Fan XF, Wu A. High concentrations of visfatin in the peripheral blood of patients with acute basal ganglia hemorrhage are associated with poor outcome. Peptides. 2013;39:55-58. doi:10.1016/j.peptides.2012.11.006 | **Total** 128 patients | **Inclusion**  + Patients with acute sICH basal ganglia  + Presented within 6h  **Exclusion** + Neurological disase  + Head trauma  + Presence of other prior systemic disases (uremia, liver cirrhosis, malignancy, chronic heart lung disease)  + Hematoma evacuation | Basal ganglia hemorrhage | 6-mo mortality  6-mo functional outcome | Mortality  mRS  0-2 good  3-6 poor | + Triglyceride  + Cholesterol  + Creatinkinase  + Glucose  + CRP  + D-dimer  + WBC  + Hemoglobin  + Platelet  + Prothrombin  + Thrombin time  + Thromboplastin time  + Fibrinogen level  + VIsfatin | Mean (SD) | + Age  +BMI  + HTN  + DM  + NIHSS score  + Hematoma volume  + IVH  + Hemorrhage growth  + END |  |
| 188 | Huang et al. (2022)  China | Huang X, Wang D, Zhang Q, et al. Development and Validation of a Clinical-Based Signature to Predict the 90-Day Functional Outcome for Spontaneous Intracerebral Hemorrhage. Front Aging Neurosci. 2022;14:904085. Published 2022 May 9. doi:10.3389/fnagi.2022.904085 | **Total** 1,122 | **Inclusion**  + Age > 18 years  + Presented with sICH  + Baseline CT 6h  **Exclusion:** + Secondary ICH (tumor, trauma, aneurysm, hemorrhagic transformation, AV)  + Primary IVH  + SAH  + Volume of hematoma <1mL  + Inadequate data | All sICH | 90-day functional outcome | mRS  0-3 good  4-6 poor | + Glu  + TG  +WBC  + NE  + LY  + NLR  + HGB  + INR | Mean (SD) | + Age  + Sex  + Location  + SAH  + IVH  + PHE volume  + ICH volume  + Smoking | Included mRS 3 as good outcome criteria |
| 189 | Huangfu et al. (2020)  China | Huangfu XQ, Wang LG, Le ZD, Tao B. Utility of serum amyloid A as a potential prognostic biomarker of acute primary basal ganglia hemorrhage. Clin Chim Acta. 2020;505:43-48. doi:10.1016/j.cca.2020.02.022 | **Total** 159 | **Inclusion** + Primary basal ganglia hemorrhage  + Admitted within 24h after onset  **Exclusion** + Underwent surgical procedure  + Infection within 4 weeks  + Coexisting autoimmune disase  + malignancy  + Neurological disease (previous ischemic, hemorrhagic stroke, head trauma) | Basal ganglia hemorrhage | 90-day mortality  90-day functional outcome | Mortality  mRS 0-2 good  3-6 poor | + Hemoglobin  + Platelet count  + Fibrinogen  + D-dimer  + Glucose  + CRP  + WBC count  + Serum SAA | Median (IQR) | + IVH  + HTN  + DM  + Hyperlipidemia  + Alcohol  + Age  + Sex |  |
| 190 | Jiang et al. (2020)  China | Jiang W, Jin P, Bao Q, Wei W, Jiang W. Prognostic significance of serum translocator protein in patients with spontaneous intracerebral hematoma：preliminary findings. Neurol Res. 2021;43(5):412-417. doi:10.1080/01616412.2020.1866372 | **Total** 97 patients | **Inclusion** + Patient with cerebral hematoma  + Age 16-80 years  + Vital signs were stable  **Exclusion**  + History of brain injury, neurologic disase, hemorrhagic infarction, bleeding disorder  + Severe cardiac and renal insufficiency  + Severe pulmonary infection | Cerebral hematoma | 3-mo functional | mRS  0-2 good  3-6 poor | + TSPO | Lower vs higher | + HTN  + DM  + Smoking  + Hematoma volume  + Edema  + Location | Not stating aetiology of hemorrhage |
| 191 | Katsuki et al. (2020)  Japan | Katsuki M, Kakizawa Y, Nishikawa A, Yamamoto Y, Uchiyama T. Lower total protein and absence of neuronavigation are novel poor prognostic factors of endoscopic hematoma removal for intracerebral hemorrhage. J Stroke Cerebrovasc Dis. 2020;29(9):105050. doi:10.1016/j.jstrokecerebrovasdis.2020.105050 | **Total** 75 | **Inclusion**  + Hypertensive ICH  + Basal ganglia, subcortex, cerebellum  + Indicated for surgery  + Interval between onset and hematoma removal <24h  **Exclusion**  + Secondary ICH  + Thalamic or caudate head with IVH treated by flexible neuroendoscope | All site hypertensive ICH | Neurological function 7-day  6-mo functional outcome | GCS  mRS  0-2 good  3-6 poor | + Lymphocyte count  + Platelet  + Total protein  + Albumin  + Total cholesterol  +LDL  + Glucose  + PT-INR  + aPTT | Mean (SD) | + Age  + Sex  + Hematoma location  + IVH  + Hydrocephalus  + GCS score  + NIHSS score  + Drinking  + HTN  + Dyslipidemia  + DM  + Antithrombotic drugs  + Antiplatelet  + Anticoagulant  + CVD  + CKD  + Cancer  + history of stroke |  |
| 192 | Kuznietsov et al. (2021)  Ukraine | Kuznietsov AA. Prognostic value of serum concentration of matrix metalloproteinase-9 in patients in acute period of spontaneous supratentorial intracerebral hemorrhage. *UMJ*. Doi: 10.32471/umj.1680-3051.142.204800 | **Total**  105 | **Inclusion** + sICH hypertensive supratentorial  + Admission within first 24^th^ h upon the onset of the disease  + Signed consent  **Exclusion** + Acute cerebrovascular disorder  + Secondary ICH due to brain tumor, infarction, anticoagulant therapy  + Acute focal lesion of cerebral structure  + indications for surgical treatment  + Diseases of inflammatory genesis (including infectious) upon hospitalization | Supratentorial hypertensive ICH | Early neurological deterioration  21-day functional outcome | FOUR coma scale >2 or NIHSS > 4 within 48h from admission  mRS  0-3 good  4-6 poor | + MMP-9 | ROC curve | Not stated | Biomarkers not reported in mean or median |
| 193 | Landreneau et al. (2018)  USA | Landreneau MJ, Mullen MT, Messé SR, et al. CCL2 and CXCL10 are associated with poor outcome after intracerebral hemorrhage. Ann Clin Transl Neurol. 2018;5(8):962-970. Published 2018 Jul 3. doi:10.1002/acn3.595 | **Total**  115 | **Inclusion:**  + Age >18 years  + Presented with sICH within 24h  **Exclusion**  + Secondary ICH (AVM, AVF, aneurysm, venous sinus thrombosis, trauma)  + Systemic malignancy  + Immunosuppresion  + Autoimmune disease  + Pre-stroke disability (mRS > 2) | sICH all site | 90-day functional outcome | mRS  0-3 good  4-6 poor | + CCl2  + G-CSF  + GM-CSF  + Cx3Cl1  + IL-10  + CCl7  + CCl22  + IL-1ra  + IL-1beta  + IL-4  + IL-6  + IL-8  + CxCl10  + TNF | Odds ratio | Not stated | No biomarker data for the reported outcomes |
| 194 | Leasure et al. (2021)  USA | Leasure AC, Kuohn LR, Vanent KN, et al. Association of Serum IL-6 (Interleukin 6) With Functional Outcome After Intracerebral Hemorrhage. Stroke. 2021;52(5):1733-1740. doi:10.1161/STROKEAHA.120.032888 | **Total**  552 | **Inclusion:**  + Patients with sICH presenting within 3h from symptom onset  + Age > 18 years  **Exclusion**  + Secondary ICH  + Existing disability  + Haemophilia | sICH all site | 90-day functional outcome | mRS  0-3 good  4-6 poor | IL-6 | Odds Ratio | Not stated | Biomarkers not reported in mean or median |
| 195 | Li et al. (2021)  China | Li S, Wang W, Zhang Q, Wang Y, Wang A, Zhao X. Association Between Alkaline Phosphatase and Clinical Outcomes in Patients With Spontaneous Intracerebral Hemorrhage. Front Neurol. 2021;12:677696. Published 2021 Aug 30. doi:10.3389/fneur.2021.677696 | **Total** 939 | **Inclusion** + ICH patients diagnosed by the WHO standard and confirmed by CT scan  +First ever acute onset ICH  + Age > 18 years  + In hospital 72h after onset  **Exclusion**  + Complicated with major comorbidities or late-stage disease (liver failure, end-stage kidney disase, heart failure, malignant tumor  + Secondary ICH (aneurysm, cerebrovascular malformation, venous thrombosis, trauma, tumor, hemorrhagic transformation of ischemic stroke)  + Primary ventricular hemorrhage  + Lack of serum ALP data | sICH all site | 30-day functional outcome  90-day functional outcome  1-year functional outcome | mRS  0-2 good  3-6 poor | Serum ALP | Cut off quartiles  Odds Ratio | Not stated | Biomarkers not reported in mean or median |
| 196 | Li et al. (2021)  China | Li J, Yuan Y, Liao X, Yu Z, Li H, Zheng J. Prognostic Significance of Admission Systemic Inflammation Response Index in Patients With Spontaneous Intracerebral Hemorrhage: A Propensity Score Matching Analysis. Front Neurol. 2021;12:718032. Published 2021 Sep 24. doi:10.3389/fneur.2021.718032 | **Total** | **Inclusion**  + Patients ICH  **Exclusion**  + Age <18 years  + Secondary ICH (aneurysm, AV malformation, tumor)  + Incomplete baseline data  + ABsence of CT angiography and follow-up CT within 24h  + History of infectious disease, cancer, rheumatic disease  + Loss to follow-up | sICH all location | 90-day functional outcome | mRS  0-2 good  3-6 poor | PLT  PT  APTT  INR  Neutrophil  Lymphocyte  Monocyte  NLR  SIRI | Mean (SD) | + Age  + Sex  + GCS  + HTN  + DM  + Smoking  + Alcohol  + Previous stroke  + Antiplatelet or anticoagulation + Hematoma volume  + Location  + IVH  + Surgery |  |
| 197 | Li et al. (2021)  China | Li Y, Wen D, Cui W, et al. The Prognostic Value of the Acute Phase Systemic Immune-Inflammation Index in Patients With Intracerebral Hemorrhage. Front Neurol. 2021;12:628557. Published 2021 May 25. doi:10.3389/fneur.2021.628557 | **Total** 291 | **Inclusion**  + > 18 years  + Admission ICH diagnosis based on CT  + <24h from onset to admission  + Available clinical data  + Neuro-image  **Exclusion**  + Secondary ICH (aneurysm, vascular malformation, tumor)  + Possible disease that affect result (leukemia, lymphoma, thrombocytopenia)  + Unavailability of outcome data  + Coagulopathy or anticoagulant therapy  + Active infection or autoimmune disease | sICH all location | 90-day functional outcome | mRS  0-3 good  4-6 poor | Systemic immune inflammation index | Score | + Age  + Sex  + GCS  + Craniotomy  + ICH volume  +IVH  + Location | Biomarkers not reported in mean or median |
| 198 | Li et al. (2023)  China | Li W, Lv X, Ma Y, Cai Y, Zhu S. Prognostic significance of serum NLRC4 in patients with acute supratentorial intracerebral hemorrhage: A prospective longitudinal cohort study. Front Neurol. 2023;14:1125674. Published 2023 Mar 10. doi:10.3389/fneur.2023.1125674 | **Total** 148 | **Inclusion**  + Non-traumatic supratentorial ICH  + Age >18 years  + First time stroke  + Conservative treatment of hematoma  + Hospital admission within first 24h  **Exclusion**  + Secondary ICH  + Primary IVH  + Oncopathology of cerebral and cerebral location  + Specific or severe disease | Supratentorial sICH | Early neurological deterioration  6-mo functional outcome  6-mo mortality | NIHSS > 4 in 24h  mRS  0-2 good  3-6 poor  Mortality | NLRC4` level | Odds ratio  ROC curve | + Age  + Sex  + BMI  + HTN  + DM  + Hyperlipidemia  + Coronary heart disease  + Cigarette  + Alcohol  + Statin  + Anticoagulant  + Antiplatelet  + Lobar hemorrhage  +IVH  +SAH  + Hematoma volume |  |
| 199 | Lin et al. (2017)  China | Lin Q, Cai JY, Lu C, et al. Macrophage migration inhibitory factor levels in serum from patients with acute intracerebral hemorrhage: Potential contribution to prognosis. Clin Chim Acta. 2017;472:58-63. doi:10.1016/j.cca.2017.07.016 | **Total** 120 ICH  120 control | **Inclusion**  + Patients with acute spontaneous basal ganglia hemorrhage  **Exclusion**  + Infection within 1 month  + Previous ICH  + Ischemic stroke or sever head trauma  + Use of antiplatelet or anticoagulant medication  + Presence of other prior systemic disase (autoimunne, uremia, liver cirrhosis, malignancy, chronic heart or lung disease)  + Underwent surgical procedure | Basal ganglia sICH | END  6-mo functional outcome | NIHSS > 4 within 24h  mRS  0-2 good  3-6 poor | Plasma CRP  Blood glucose  Serum MIF | Hazard Ratio | + Age  + Sex  + HTN  + DM  + Thalamic and caudate hemorrhage  + NIHSS Score  + Hematoma volume  + IVH  + Hemorrhage growth  + END | Biomarkers not reported in mean or median |
| 200 | Lin et al. (2022)  China | Lin H, Shen J, Zhu Y, et al. Serum CCL23 emerges as a biomarker for poor prognosis in patients with intracerebral hemorrhage. *Clin Chim Acta*. 2022;537:188-193. doi:10.1016/j.cca.2022.10.012 | **Total** 94 patients | **Inclusion**  + Admitted within 24h for hemorrhage  **Exclusion**  + Age >18 years  + Infection within 1 month  + Previous ICH  + Ischemic stroke or sever head trauma  + Use of antiplatelet or anticoagulant medication  + Presence of other prior systemic disase (autoimunne, uremia, liver cirrhosis, malignancy, chronic heart or lung disease)  + Underwent surgical procedure | Primary ICH all site | 6-month functional outcome  6-mo mortality | mRS  0-2 good  3-6 poor  Mortality | CCl23  Glucose  CRP  RBC  WBC  Neutrophil | Cut-off 62.95pg/mL  Median (IQR) | + Age  + Sex  + HTN  + DM  + Coronary artery disease  + Smoking  + Alcohol  + NIHSS  + ICH Score  + GCS Score  + Hematoma volume  + Subtentorial hemorrhage | Biomarkers reported not related to outcome |
| 201 | Lou et al. (2020)  China | Lou XH, Cai YY, Yang XQ, et al. Serum netrin-1 concentrations are associated with clinical outcome in acute intracerebral hemorrhage. *Clin Chim Acta*. 2020;508:154-160. doi:10.1016/j.cca.2020.05.032 | **Total** 126 ICH patients  126 controls | **Inclusion**  + Patients with ICH admitted within 24 h  **Exclusion**  + Secondary ICH (head trauma, rupture AVM, cerebral aneurysm, venous sinus thrombosis, coagulation, cerebral infarction, tumor)  + Infection within 1 month  + Previous stroke | Non-traumatic sICH all site | 90-day functional outcome  90-day mortality | mRS  0-2 good  3-6 poor  Mortality | Serum netrin-1 level | Box plot | Not stated | Biomarker not reported in other studies |
| 202 | Machin et al. (2021)  Indonesia | Machin A, Aulia NN, Setyowatie S. Malodialdehyde levels and clinical outcomes assessed by the modified Rankin scale in patients with acute intracerebral hemorrhagic stroke. *Gac Med Caracas* 2021; 129 (Suppl2): S367-72. Doi: 10.47307/GMC.2021.129.s2.14 | **Total** 34 | **Inclusion:**  + Age > 20 years  + Patients with acute ICH  **Exclusion**  + Patients with acute ICH stroke with IVH or SAH  + Having active infection  + Patients with DM, acute coronary heart disase, history of malignancy, Parkinson disease | All cause ICH | Discharge functional outcome | mRS | Malondhyaldehyde | Cut off 494.95ng/mL | Age  GCS  Hemorrhage volume | Biomarkers not reported in mean or median |
| 203 | Marti-Fabregas et al. (2014)  Germany and Spain | Martí-Fàbregas J, Delgado-Mederos R, Marín R, et al. Prognostic value of plasma β-amyloid levels in patients with acute intracerebral hemorrhage. Stroke. 2014;45(2):413-417. doi:10.1161/STROKEAHA.113.002838 | **Total 160** | **Inclusion**  + Patients with sICH within 24 h  **Exclusion**  + Tumor or AVM | All cause ICH | 3-mo functional outcome | mRS  0-2 good  3-6 poor | Serum alpha-beta amyloid | Mean (SD) | + Age  + Sex  + HTN  + DM  + Hypercholesterolemia  + Ischemic heart disase  + Prior ischemic stroke  + Prior ICH  + Glucose  + NIHSS  + IVH  + Deep location  + Aetiology  + Hematoma volume | Biomarker is not reported in other studies |
| 204 | Masomi-Bornwasser et al.(2021)  Germany | Masomi-Bornwasser J, Kurz E, Frenz C, et al. The Influence of Oxidative Stress on Neurological Outcomes in Spontaneous Intracerebral Hemorrhage. Biomolecules. 2021;11(11):1615. Published 2021 Nov 1. doi:10.3390/biom11111615 | **Total** | **Inclusion**  + Prsent with ICH >30mL  + Nescessitating EVD  + Age > 18 years  + sICH non-traumatic  + Diagnosed within 4h  **Exclusion**  + Bleeding  + Infection  + Inflammation | All site sICH | 6-mo neurological function  6-mo functional outcome | GCS  mRS | GSH-Px | Correlation | Not stated | Not reporting biomarker in mean or range |
| 205 | Muresan et al. (2022)  Romania | Mureșan EM, Golea A, Vesa ȘC, Lenghel M, Csutak C, Perju-Dumbravă L. Emergency department point-of-care biomarkers and day 90 functional outcome in spontaneous intracerebral hemorrhage: A single-center pilot study. Exp Ther Med. 2022;23(3):200. doi:10.3892/etm.2022.11123 | **Total** | **Inclusion**  + Presenting with acute sICH <8h from onset  + Admission GCS >8  **Exclusion** + Secondary ICH  + Thromboembolic/ischemic disase  + Seizure  + Severe pre-ICH disability (mRS > 4)  + Coagulopathy  + Pregnancy/breastfeeding  +Schedulced neurosurgical/hemostatic treatment | All site sICH | 90-day functional outcome | mRS  0-3 good  4-6 poor  Barthel Index <60 | Hb  RBC  RDWa  WBC  GRA Lym  MID  PLT  NLR  LMR  PLR  SII  hs-CRP  cTnI  D-dimer  Glucose | Median (range) | + Age  + Gender  + HTN  + DM  + Dyslipidemia  + Statin use  + Antiplatelet  + Smoking  + GCS + NIHSS score  + AF  + Hematoma location  + Hematoma volume  + IVH  + MLS  + Mass effect  + CVC  + ECV  + Periventricular leucoaraiosis  + Lacunarism | mRS criteria not the same with other studies |
| 206 | Muresan et al. (2022)  Romania | Muresan EM, Golea A, Vesa SC, Givan I, Perju-Dumbrava L. Admission Emergency Department Point-of-care Biomarkers for Prediction of Early Mortality in Spontaneous Intracerebral Hemorrhage. In Vivo. 2022;36(3):1534-1543. doi:10.21873/invivo.12864 | **Total** 219 | **Inclusion**  + Age > 18 years  + Diagnosed with sICH  **Exclusion**  + Secondary ICH (traumatic, AV malformation, aneurysm, tumor, infection, hemorrhagic transformation of ischemic stroke) | All site sICH | In hospital mortality  7-day mortality | Mortality | + Hb  + RBC  + WBC  + GRA  + LYM  + MID  + PLT  + NLR  + LMR  + PLR  + SII  + SIRI  + Glucose  + ASAT  + ALAT  + BUN  + Crea  + hs-CRP  + cTnI  + D-dimer | Median (range) | Not stated` |  |
| 207 | Mustanoja et al. (2013)  Finland | Mustanoja S, Strbian D, Putaala J, et al. Association of prestroke statin use and lipid levels with outcome of intracerebral hemorrhage. Stroke. 2013;44(8):2330-2332. doi:10.1161/STROKEAHA.113.001829 | **Total 964** | **Inclusion**  + Patients with ICH | All cause ICH | In hospital mortality | Mortality | Lipid level |  |  | Included ICH from various aetiologies |
| 208 | Naidech et al. (2009)  USA | Naidech AM, Jovanovic B, Liebling S, et al. Reduced platelet activity is associated with early clot growth and worse 3-month outcome after intracerebral hemorrhage. Stroke. 2009;40(7):2398-2401. doi:10.1161/STROKEAHA.109.550939 | **Total** 68 | **Inclusion**  + Patients with ICH  **Exclusion**  + Patients with secondary ICH (trauma,ruptured aneurysm, AVM, vasculitis) | Non traumatic all site ICH | 3-mo functional outcome  3-mo mortality | mRS  0-2 no disability  3-5 severe disability  6 death | Aspirin reaction units | Cut off 551 | Not stated | Not reporting biomarker in mean or range |
| 209 | Navaro-Oviedo et al. (2020)  Spain | Navarro-Oviedo M, Muñoz-Arrondo R, Zandio B, et al. Circulating TIMP-1 is associated with hematoma volume in patients with spontaneous intracranial hemorrhage. Sci Rep. 2020;10(1):10329. Published 2020 Jun 25. doi:10.1038/s41598-020-67250-9 | **Total** 105 | **Inclusion**  + Patients with nontraumatic spontaneous supratentorial ICH  **Exclusion**  + Infratentorial ICH  + Secondary ICH (vascular malformation, aneurysm, tumors, ischemic stroke with hemorrhagic transformation)  + Subdural hematoma, epidural bleed, SAH | Supratentorial ICH | 90-day functional outcome | mRS | MMP-1  MMP-2  MMP-7  MMP-9  MMP-10  TIMP-1  Glucose | Correlation | Age  Sex  HTN  Diabetes  Dyslipidemia  Hematoma lobar  mRS admission | Not reporting biomarkers mean or median |
| 210 | Roy O’Reilly et al. (2017) | Roy-O'Reilly M, Zhu L, Atadja L, et al. Soluble CD163 in intracerebral hemorrhage: biomarker for perihematomal edema. Ann Clin Transl Neurol. 2017;4(11):793-800. Published 2017 Oct 19. doi:10.1002/acn3.485 | **Total** 51 | **Inclusion**  + Patients with basal ganglia or thalamus hypertensive ICH  **Exclusion**  + Coagulopathic  + Subacute hematoma volume increases >48h postictus  + Secondary ICH | Deep supratentorial | Functional outcome at discharge  90-day functional outcome | mRS | CD-163 | Not reported in relation to functional outcome |  | The reported biomarker is not related to functional outcome |
| 211 | Qi et al. (2018)  China | Qi H, Wang D, Deng X, Pang X. Lymphocyte-to-Monocyte Ratio Is an Independent Predictor for Neurological Deterioration and 90-Day Mortality in Spontaneous Intracerebral Hemorrhage. Med Sci Monit. 2018;24:9282-9291. Published 2018 Dec 20. doi:10.12659/MSM.911645 | **Total** 558 | **Inclusion** + Patients with ICH admitted within 24h  **Exclusion**  + Received surgery  + Immunomodulatory treatment  + Aneurysm  + Hepatitis  + AVM  + Heart failure  + Seizure  + COPD  + Brain tumor hemorrhage  + IVH | All site ICH | Neurological deterioration  90-day mortality | NIHSS >4  Mortality | WBC  ALC  ANC  AMC  NLR LMR | ROC | Not stated | Biomarkers are not reported in mean or median |
| 212 | Qin et al. (2019)  China | Qin J, Li Z, Gong G, et al. Early increased neutrophil-to-lymphocyte ratio is associated with poor 3-month outcomes in spontaneous intracerebral hemorrhage. PLoS One. 2019;14(2):e0211833. Published 2019 Feb 7. doi:10.1371/journal.pone.0211833 | **Total** | **Inclusion**  + Age 18-60 years  + Immediate routine blood sampling and CT within 24h  + Absence of secondary causes of ICH (trauma, rupture malformed cerebral vessels, hemorrhagic transformation after cerebral procedure)  + Absence of infection within 14 days  + no history of stroke for 6 mo  + Absence of cancer, autoimmune disase, severe hepatic or renal disease  + No use of steroid immunomodulatory | All site sICH | 3-mo functional outcome | mRS  0-2 good  3-6 poor | Neutrophil to lymphocyte ratio day 0, 1, 3, 7, 14 | Correlation | + Age  + Sex  + Hyperlipidemia  + ICU admission  + Endotracheal intubation  + Nasogastric feeding  + Operation  + Infection + Infratentorial hemorrhage  + IVH  + ICH vol >30  + Admission NIHSS  + GCS | Biomarkers are not reported in mean or median |
| 213 | Qin et al. (2019)  China | Qin J, Li Z, Gong G, et al. Early increased neutrophil-to-lymphocyte ratio is associated with poor 3-month outcomes in spontaneous intracerebral hemorrhage. PLoS One. 2019;14(2):e0211833. Published 2019 Feb 7. doi:10.1371/journal.pone.0211833 | **Total** 123 | **Inclusion**  + Age 18-60 years  + Immediate routine blood sampling and CT within 24h  + Absence of secondary causes of ICH (trauma, rupture malformed cerebral vessels, hemorrhagic transformation after cerebral procedure)  + Absence of infection within 14 days  + no history of stroke for 6 mo  + Absence of cancer, autoimmune disase, severe hepatic or renal disease  + No use of steroid immunomodulatory | All site sICH | 3-mo functional outcome | mRS  0-2 good  3-6 poor | Systemic immune inflammation index  Lymphocyte to monocyte ratio | Cut-off |  | Not reporting biomarkers mean or median |
| 214 | Qiu et al. (2016)  China | Qiu SZ, Wang HX, Shen J, et al. The prognostic value of serum signal peptide-Cub-Egf domain-containing protein-1 concentrations in acute intracerebral hemorrhage. Clin Chim Acta. 2016;461:103-109. doi:10.1016/j.cca.2016.08.001 | **Total** 128 | **Inclusion**  + Age > 18 years  + Acute spontaneous basal ganglia hemorrhage  **Exclusion** + Patients with secondary ICH (brain tumor, AVM, trauma)  + Acute or chronic infection within 4 weeks before ICH  + Previous ischemic or hemorrhagic stroke  + Severe head trauma  + Use of antiplatelet or anticoagulant medication  + Con current comorbidities | Basal ganglia hemorrhage | 6-mo functional outcome | mRS  0-2 good  3-6 poor | Peptide-Cub-EGF domain-containing protein 1 | Odds ratio  ROC | Not stated | Not reported biomarker mean or median |
| 215 | Qiu et al. (2016)  China | Qiu SZ, Zheng GR, Ma CY, et al. High Serum S100A12 Levels Predict Poor Outcome After Acute Primary Intracerebral Hemorrhage. Neuropsychiatr Dis Treat. 2021;17:3245-3253. Published 2021 Nov 2. doi:10.2147/NDT.S337041 | **Total** | **Inclusion**  + Age > 18 years  + Admitted within 24h of stroke  + Not undergo surgical evacuation of hematoma  **Exclusion**  + Secondary ICH  + Infection within 1 month  + Previous neurological diseases  _ Use of antiplatelet or anticoagulant  + Autoimmune disase  + Malignancies  + Severe systemic disease | All site ICH | END  3-month functional outcome | NIHSS > 4 at 24h  mRS  0-2 good  3-6 poor | S100A  Leukocyte count  Glucose  CRP | Median (range) | + Age  + Gender  + HTN  + DM  + Alcohol  + Infratentorial hemorrhage  + IVH  + Hematoma volume |  |
| 216 | Rodriguez-Luna et al. (2011)  Spain | Rodriguez-Luna D, Rubiera M, Ribo M, et al. Serum low-density lipoprotein cholesterol level predicts hematoma growth and clinical outcome after acute intracerebral hemorrhage. Stroke. 2011;42(9):2447-2452. doi:10.1161/STROKEAHA.110.609461 | **Total** | **Inclusion**  + Patients with acute primary supratentorial ICH admitted within 6h  **Exclusion**  + Anticoagulant treatment  + GCS <8  + Underwent surgical procedure | Supratentorial sICH primary | END  3-mo functional outcome  3-mo mortality | NIHSS >4 within 24h  mRS  0-2 good  3-6 poor  Mortality | Glucose  Creatinine  Hb  Leukocyte  Platelet  PT  aPTT  Fibrinogen  Albumin  Total-C  LDL-C  HDL-C  Triglyceride | Median (range) | + Age  + Sex  + Antiplatelet  + Statin  + GCS median  + NIHSS  + ICH volume  + IVH  + Lobar ICH |  |
| 217 | Saxena et al. (2016)  Australia | Saxena A, Anderson CS, Wang X, et al. Prognostic Significance of Hyperglycemia in Acute Intracerebral Hemorrhage: The INTERACT2 Study. Stroke. 2016;47(3):682-688. doi:10.1161/STROKEAHA.115.011627 | **Total** | **Inclusion**  + Patients with spontaneous ICH within 6h  + Systolic BP 150-220mmHg  **Exclusion**  + Contraindication to intensive BP lowering (eg, severe carotid, vertebral or cerebral arterial stenosis, known Moya Moya disease or Takayasu's arteritis, High-grade stenotic valvular heart disease or severe renal failure)  + Indication to intensive BP lowering (eg, systolic BP >220 mmHg, hypertensive encephalopathy, or aortic dissection)  + the ICH is secondary to a structural abnormality in the brain or recent thrombolysis  + ischaemic stroke within the last 30 days  + high likelihood of death within the next 24 hours  existing dementia or significant pre-stroke disability  + planned early surgical evacuation of the haematoma  + concomitant illness that may interfere with outcome assessments and follow-up  + previously participated in INTERACT2 or currently participating in another investigational drug trial  + high likelihood of not adhering to the study treatment and follow-up | Spontaneous ICH | 90-day functional outcome  END | mRS  0-2 good  3-6 poor  NIHSS > 4  GCS > 2 | Glucose level | Odds Ratio | Not stated | Biomarkers are not reported in mean or median |
| 218 | Semerano et al. (2020)  Italy | Semerano A, Strambo D, Martino G, et al. Leukocyte Counts and Ratios Are Predictive of Stroke Outcome and Hemorrhagic Complications Independently of Infections. Front Neurol. 2020;11:201. Published 2020 Apr 3. doi:10.3389/fneur.2020.00201 | **Total** | **Inclusion**  + Patients diagnosed with ischemic stroke within 4.5h from symptom onset  **Exclusion**  + Patients with history of pre-existing recent infections, hematological malignancies, recent surgery | Ischemic stroke | 3-mo functional outcome | mRS  0-2 good  3-6 poor | Total WBC  Neutrophil  Lymphocyte  NLR  Eosinophil  ELR  Monocyte | Odds ratio | Not stated | Cases of ischemic stroke not hemorrhagic stroke |
| 219 | Shen et al. (2017)  China | Shen J, Chen B, Zheng GR, et al. Detection of high serum concentration of CXC chemokine ligand-12 in acute intracerebral hemorrhage. Clin Chim Acta. 2017;471:55-61. doi:10.1016/j.cca.2017.05.022 | **Total** 105 ICH  105 controls | **Inclusion**  + Patients with first-ever acute sICH basal ganglia  + Admitted within 24h after symptom  **Exclusion** + Infection within a month  + Secondary cause ICH  + History of other neurological disease  + Use of antiplatelet or anticoagulant  + Surgical procedure  + Concurrent comorbidities (autoimmune, uremia, liver cirrhosis, malignancy, chronic heart or lung disease) | sICH basal ganglia | END  6-mo functional outcome  6-mo Mortality | NIHSS >4  mRS  0-2 good  3-6 poor  Mortality | CxCl12 | Dot plot | + Age  + Sex  + HTN  + DM  + NIHSS >9  + Hematoma volume >30  + IVH  + END | Biomarker is not reported in other studies |
| 220 | Song et al. (2022)  China | Song X, Liu J, Wang Y, Zheng L, Liu M. Serum microRNA miR-491-5p/miR-206 Is Correlated with Poor Outcomes/Spontaneous Hemorrhagic Transformation after Ischemic Stroke: A Case Control Study. Brain Sci. 2022;12(8):999. Published 2022 Jul 28. doi:10.3390/brainsci12080999 | **Total** 215 | **Inclusion**  + Patients with acute ischemic stroke  **Exclusion**  + Did not have blood samples at admission  + Had ICH at admission  + Received reperfusion therapies | Ischemic stroke | 3-mo functional outcome | mRS  0-2 good  3-6 poor | miR-21-5p  miR-491-5p  miR-312  miR-206 | Median (range) | + Age  + Sex  + GCS  + NIHSS  + HTN  + DM  + Hyperlipidemia  + AF  + Acut MI  + Transient ischemic attack  _ Antiplatelet  + Anticoagulant | Cases of ischemic not hemorrhagic stroke |
| 221 | Sun et al. (2017)  China | Sun DB, Xu MJ, Chen QM, Hu HT. Significant elevation of serum caspase-3 levels in patients with intracerebral hemorrhage. Clin Chim Acta. 2017;471:62-67. doi:10.1016/j.cca.2017.05.021 | **Total** 112 | **Inclusion**  + First ever acute spontaneous basal ganglia hemorrhage  + Admitted within 24h of hemorrhage onset  + Did not undergo surgical hematoma evacuation  **Exclusion**  + Previous neurological disases  + Infection within 1 month  + Prior usage of anticoagulant  + Presence of other prior systemic disease | Spontaneous basal ganglia | END  6-mo functional outcome  6-mo mortality | NIHSS > 4 within 24h  mRS  0-2 good  3-6 poor  Mortality | Serum caspase-3  Glucose  CRP  D-dimer  WBC count | Dot plot  Odds ratio | + Sex  + Age  + HTN  + DM  + IVH  + Hemorrhage growth  + END  + NIHSS score  + Hematoma volume | Biomarkers are not reported in mean or median |
| 222 | Tang et al. (2014)  China | Tang SC, Yeh SJ, Tsai LK, et al. Association between plasma levels of hyaluronic acid and functional outcome in acute stroke patients. J Neuroinflammation. 2014;11:101. Published 2014 Jun 10. doi:10.1186/1742-2094-11-101 | **Total** 206 | **Inclusion**  + Acute stroke  + Admitted within 24h after stroke onset  + Initially blood drawn within 48h after stroke onset  **Exclusion**  + Patients with known active infection  + Cancer  _ Autoimmune disorder  + Current steroid treatment | Ischemic or ICH | 3-mo functional outcome | mRS  0-2 good  3-6 poor | WBC  Hemoglobin  Glucose  Creatinine | Mean (SD) | + Age  + Sex  + BMI  + DM  + Hypertension  + Hyperlipidemia  _+ AF | Not stating sICH patients aetiology |
| 223 | Tan et al. (2016)  China | Tan G, Hao Z, Lei C, et al. Subclinical change of liver function could also provide a clue on prognosis for patients with spontaneous intracerebral hemorrhage. Neurol Sci. 2016;37(10):1693-1700. doi:10.1007/s10072-016-2656-0 | **Total** 639 | **Inclusion**  + Patients with sICH admitted within 7 days from stroke onset  **Exclusion**  + Primary SAH  + Secondary ICH by trauma, brain tumor, transformation of ischemic stroke  + Age <18 years  + Received anticoagulant or antiplatelet  + Severe liver disease, end-stage renal disease, hematologic disease | Spontaneous ICH | 30-day mortality  90-day mortality  90-day functional outcome | Mortality  mRS  0-2 good  3-6 poor | ALT  AST  ALP  GGT  BIL  ALB  INR | Mean (SD) | + Age  + Sex  + HTN  + DM  + Dyslipidemia  + Drinking  + Smoking  + GCS  + Hematoma location  + Surgery | Patients admitted >72 h since symptoms onset |
| 224 | Tony et al. (2018)  Egypt | Tony AA, Tony EA, Kholef EF, Mohammed WS. Vitamin D status and spontaneous intra cerebral hemorrhage: Would it be able to help?  *Med J Cairo Univ* 2018; 86(7): 3863-72 | **Total** 125 | **Inclusion** + First ever acute onset ICH  + Acute stroke with manifestation beginning within 24-48 h  + Age 25-87  **Exclusion**  + Patients with ischemic stroke  + History of past stroke  + Cerebral amyloid angiopathy  + Vascular malformation and aneurysm  + IVH or HC  + Brain neoplasm  + Endocrinal disorder  + Hepatic or renal disability  + Past fracture  + Bone disease  + Vitamin D or Ca supplementation  + Steroid therapy | sICH all site | In hospital mortality | Mortality | Total calcium  Phosphorus  Parathormone  Vitamin D | Mean (SD) | Not stated | No defined time for the outcome |
| 225 | Urwyler et al. (2010)  Germany | Urwyler SA, Schuetz P, Fluri F, et al. Prognostic value of copeptin: one-year outcome in patients with acute stroke. *Stroke*. 2010;41(7):1564-1567. doi:10.1161/STROKEAHA.110.584649 | **Total** 362 | **Inclusion**  + Patients with ischemic stroke  + Completed 1 year follow-up  **Exclusion**  + | Ischemic stroke | 1-year functional outcome  1-year mortality | mRS 0-2 good  3-6 poor | Copeptine | Cut-off | Not stated | Cases of ischemic stroke not hemorrhagic stroke |
| 226 | Walsh et al. (2015)  USA | Walsh KB, Sekar P, Langefeld CD, et al. Monocyte Count and 30-Day Case Fatality in Intracerebral Hemorrhage. Stroke. 2015;46(8):2302-2304. doi:10.1161/STROKEAHA.115.009880 | **Total** 240 | **Inclusion**  + Non-Hispanich white, black, Hispanic ICH patients aged > 18 years  + Resident within 75 miles of 1 of the 19 recruitement centers  + Spontaneous ICH  **Exclusion**  + Secondary ICH (malignancy, coagulopathy, dural venous sinus thrombosis, vascular malformation, aneurysm, tumor, hemorrhagic conversion of ischemic stroke | sICH all site | 30-day case fatality | Mortality | Total WBC  Neutrophil  Monocyte | Odds ratio | + Age  + GCS  + Volume  + Location  + IVH | Biomarkers not reported in mean or median |
| 227 | Wang et al. (2011)  China | Wang KW, Cho CL, Chen HJ, et al. Molecular biomarker of inflammatory response is associated with rebleeding in spontaneous intracerebral hemorrhage. Eur Neurol. 2011;66(6):322-327. doi:10.1159/000332027 | **Total 59** | **Inclusion**  + Patients with sICH  **Exclusion**  + Patients with history of stroke  + Infection within 3 months  + Concurrent major cardiac, renal, hepatic, autoimmune, cancerous disease  + Previous head trauma  + Taking immunosuppressant, anti-inflamation, bleeding tendency | sICH all aetiologies all site | 1-mo and 6-mo neurological outcome | GOS  0-3 good  4-5 poor | ICAM-1  C3  IL-10  Glucose | Median (range) | + Age  + Sex  + Type ICH  + HC + HTN  Aetiology  + DM  + Renal disase  + Liver  + Hematoma volume >30 | Include all aetiology of ICH |
| 228 | Wang et al. (2014)  China | Wang CL, Lin HY, Xu JW, et al. Blood levels of adrenomedullin on admission predict outcomes after acute intracerebral hemorrhage. Peptides. 2014;54:27-32. doi:10.1016/j.peptides.2014.01.005 | **Total** 104 ICH patients  112 controls | **Inclusion**  + Patients with spontaneous basal ganglia hemorrhage  **Exclusion**  + Previous troke  + Severe head trauma  + Use of antiplatelet or anticoagulant  + Presence of other prior systemic disease  + Surgical hematoma evacuation | Basal ganglia sICH | END  3-mo functional outcome | NIHSS > 4  mRS  0-2 good  3-6 poor | Glucose  CRP  D-Dimer  WBC count  Hb  Platelet  Prothrombin time  Thrombin time  pTT  Fibrinogen  Adrenomedulin | Mean (SD) | + Age  + Sex  + HTN  + DM  + NIHSS + Hematoma volume  + IVH  +Hemorrhage growth  + END |  |
| 229 | Wang et al. (2018)  China | Wang LG, Huangfu XQ, Tao B, Zhong GJ, Le ZD. Serum tenascin-C predicts severity and outcome of acute intracerebral hemorrhage. Clin Chim Acta. 2018;481:69-74. doi:10.1016/j.cca.2018.02.033 | **Total** 162 | **Inclusion**  + Patients with acute primary basal ganglia hemorrhage  + Admitted within 6h  **Exclusion**  + Using antiplatelet or anticoagulant  + Underwent surgical procedure  + Comorbidities  + Neurological diseases | Primary basal ganglia sICH | END  90-day functional outcome | NIHSS > 4  mRS  0-2 good  3-6 poor | Hb level  Platelet count  Fibrinogen  D-Dimer  Glucose  CRP  WBC  TNC level | Odds ratio | + Age  + Sex  + Smoking  + Alcohol  + Statin  + HTN  + DM  + NIHSS  + Hematoma volume  + IVH  + Hemorrhage growth  + END | Biomarkers not reported in mean or median |
| 230 | Wang et al. (2022)  China | Wang D, Cao Z, Li Z, et al. Homocysteine and Clinical Outcomes in Intracerebral Hemorrhage Patients: Results from the China Stroke Center Alliance. Neuropsychiatr Dis Treat. 2022;18:2837-2846. Published 2022 Dec 7. doi:10.2147/NDT.S391618 | **Total** 55,793 | **Inclusion**:  + Age >18 years  + Primary diagnosis of ICH by CT or MRI  + Admitted within 7 days  **Exclusion:**  + Hcy data were unavailable  + Patient had a history of stroke | All site ICH all aetiologies | In-hospital mortality  Functional outcome at discharge | Mortality  mRS  0-2 good  3-5 poor | Homocysteine level | Odds ratio | + Sex  + Age  + HTN  + DM | Included all cases of stroke aetiology |
| 231 | Wang et al. (2022)  China | Wang CL, Xu YW, Yan XJ, Zhang CL. Usability of serum annexin A7 as a biochemical marker of poor outcome and early neurological deterioration after acute primary intracerebral hemorrhage: A prospective cohort study. Front Neurol. 2022;13:954631. Published 2022 Aug 8. doi:10.3389/fneur.2022.954631 | **Total** 126 patients and 126 controls | **Inclusion** + Non traumatic first ever ICH  **Exclusion**  + Age < 18 years  + Hospital admission >24h  + Surgical evacuation of hematoma  + Primary IVH  + Bleeding of other cause (intracranial tumor, cerebral AVM, hemorrhagic transformation of cerebral infarction, moyamoya disease | All site ICH | END  90-day functional outcome | NIHSS > 4  mRS  0-2 good  3-6 poor | Leukocyte  Glucose  CRP  Annexin A7 | Median (range) | + Age  + Gender  + BMI  + HTN  + DM  + Hyperlipidemia  + Smoking  + Alcohol  + Statin  + Anticoagulant  + Antiplatelet  + Lobar hemorrhage  + location  + SAH  + NIHSS  + Hematoma volume  + GCS score |  |
| 232 | Wang et al. (2023)  China | Wang J, Du Y, Wang A, et al. Systemic inflammation and immune index predicting outcomes in patients with intracerebral hemorrhage. *Neurol Sci*. 2023;44(7):2443-2453. doi:10.1007/s10072-023-06632-z | **Total** 640 | **Inclusion**  + Age < 18 years  + Known major comorbidities  + Late stage diseases (heart failure, liver failure, end stage kidney disease, malignan tumors)  + Secondary ICH (AVM, trauma, aneurysma, coagulation disorder)  + Missing or extreme values of hematoma volume  + Extreme value of blood cell counte  + Missing data | All site ICH | Functional outcome at 1-mo, 3-mo, 1-year | mRS  0-3 good  4-6 poor | SIRI | Odds ratio | Not stated | Biomarkers reported not related to functional outcome |
| 233 | Wu et al. (2022)  China | Wu A, Yue H, Huang F, et al. Serum β2-microglobulin is closely associated with 3-month outcome of acute intracerebral hemorrhage: a retrospective cohort study [published online ahead of print, 2022 Sep 28]. Ir J Med Sci. 2022;10.1007/s11845-022-03170-z. doi:10.1007/s11845-022-03170-z | **Total** 231 | **Inclusion**  + Patients with ICH confirmed based on CT admitted within 24h  **Exclusion**  + Previous history of stroke in 6 month  + Traumatic brain injury  + Tumors  + Long-term use of immunosuppressant  + Severe hematological and metabolic diseases  + History of severe liver and kidney function | ICH | Functional outcome 3-month | mRS  0-2 good  3-6 poor | BUN  Crea  Uric  B2-microglobulin | Mean (SD) | + Age  + Sex  + HTN  + DM  + Smoking  + Drinking  + IVH  + Infratentorial  + Hematoma volume  + NIHSS Score  + GCS Score | Aetiologies of the ICH was not mentioned |
| 234 | Xiong et al. (2015)  China | Xiong L, Yang Y, Zhang M, Xu W. The use of serum glial fibrillary acidic protein test as a promising tool for intracerebral hemorrhage diagnosis in Chinese patients and prediction of the short-term functional outcomes. Neurol Sci. 2015;36(11):2081-2087. doi:10.1007/s10072-015-2317-8 | **Total** | **Inclusion**  + 2-6h from symptom onset to hospital admission  + Definite diagnosis of ICH or IS  **Exclusion**  + Negative findings in repeated brain imaging  + History of brain injury, ICH, IS, brain tumor  + Renal failure  + Other neurological diseases | ICH and IS | 90-day functional outcome | mRS  0-2 good  3-6 poor | GFAP | Boxplot | Not stated | Biomarker is not reported in other studies |
| 235 | Yan et al. (2016)  China | Yan XJ, Yu GF, Jie YQ, Fan XF, Huang Q, Dai WM. Role of galectin-3 in plasma as a predictive biomarker of outcome after acute intracerebral hemorrhage. *J Neurol Sci*. 2016;368:121-127. doi:10.1016/j.jns.2016.06.071 | **Total** | **Inclusion**  + Patients with acute spontaneous basal ganglia hemorrhage  + Admitted within 24h from onset to stroke  **Exclusion**  + Prior ischemic or hemorrhagic stroke  + Severe head trauma  + Use of antiplatelet or anticoagulant  + Presence of other systemic disease  + Surgical procedure  + Missing of follow-up | All aetiology basal ganglia hemorrhage | 7-day mortality  6-mo mortality  6-mo functional outcome | Mortality  mRS  0-2 good  3-6 poor | Glucose  CRP  Galectin-3 | Odds ratio | + Age  + Sex  + HTN  + DM + NIHSS score  + Hematoma volume  + IVH  + hemorrhage growth  + END | Biomarkers not reported in mean or median |
| 236 | Yan et al. (2022)  China | Yan T, Wang ZF, Wu XY, et al. Plasma SIRT3 as a Biomarker of Severity and Prognosis After Acute Intracerebral Hemorrhage: A Prospective Cohort Study. Neuropsychiatr Dis Treat. 2022;18:2199-2210. Published 2022 Sep 26. doi:10.2147/NDT.S376717 | **Total** | **Inclusion**  + First-ever stroke  + Time from onset to admission 24h  + Age > 18 years  **Exclusion**  + Hematoma from congenital or acquired coagulation abnormalities  + Hemorrhagic transformation of cerebral infarction, moya-moya, aneurysm, AVM tumor  + Surgical evacuation of hematoma  + Primary IVH  + Previous nurological disease  + Specific disease or conditions | All site ICH | 90-day functional outcome | GOS  0-3 good  4-5 poor | Glucose  Potassium  CRP  SIRT3 | Median (range) | + Age  + Sex  + HTN  + DM  + Hyperlipidemia  + Smoking  + Alcohol  + Statin  + Antiplatelet  + Anticoagulation |  |
| 237 | You et al. (2017)  China | You S, Zhong C, Zheng D, et al. Monocyte to HDL cholesterol ratio is associated with discharge and 3-month outcome in patients with acute intracerebral hemorrhage. J Neurol Sci. 2017;372:157-161. doi:10.1016/j.jns.2016.11.022 | **Total** | **Inclusion**  + Patients with CT confirmed ICH  **Exclusion**  + Patients with trauma, brain tumor, hemorrhagic transformation of ischemic stroke, VCM  + Surgery  + Time from onset to admission > 48h  + no data | All site ICH | 90-day functional outcome | mRS  0-2 good  3-6 poor | TG  TC  LDL-C  HDL-C  GLU  WBC  Monocyt  MLR | Odds ratio | + Age  + Sex  + Smoking  + HTN  + DM  + AF  + Hematoma volume  + Location  + IVH | Biomarkers not reported in median or mean |
| 238 | Zhao et al. (2013)  China | Zhao DQ, Wang K, Zhang HD, Li YJ. Significant reduction of plasma gelsolin levels in patients with intracerebral hemorrhage. *Clin Chim Acta*. 2013;415:202-206. doi:10.1016/j.cca.2012.10.048 | **Total** | **Inclusion**  + Patients with acute sICH basal ganglia  + Admitted within 6h from symptoms  **Exclusion**  + Existing previous neurological disease  + Head trauma  + Presence of other prior systemic disease  + Underwent surgical hematoma evacuation | Basal ganglia hemorrhage | END  6-mo mortality  6-mo functional outcome | NIHSS >4  Mortality  mRS  0-2 good  3-6 poor | Glucose  CRP  D-Dimer  Gelsolin  WBC Hemoglobin  Platelet  Prothrombin  Thrombin  pTT Fibrinogen | Mean (SD) | + Age  + Sex  + HTN  + DM  + Statin  + Calcium channel blocker  + NIHSS score  + Hematoma volume  + IVH  + Hemorrhage growth  + END + HC |  |
| 239 | Zhao et al. (2022)  China | Zhao Y, Xie Y, Li S, Hu M. The predictive value of neutrophil to lymphocyte ratio on 30-day outcomes in spontaneous intracerebral hemorrhage patients after surgical treatment: A retrospective analysis of 128 patients. *Front Neurol*. 2022;13:963397. Published 2022 Aug 22. doi:10.3389/fneur.2022.963397 | **Total** | **Inclusion**  + Age >18  + Diagnosed with sICH confirmed by CT 24h  + Underwent craniotomy or MIPD within 24h  **Exclusion**  + Infection for 2 weeks  + Comorbidity  + Immunomodulatory tratments  + GCS < 5  + Dissatisfied effect of EVD | sICH treated with surgery | 30-day functional outcome | mRS  0-2 good  3-6 poor | Neutrophil to leukocyte ratio | ROC curve | + Age  + Sex  _ GCS admission  + DM  + HTN  + Alcohol  + Smoking  + Hematoma volume  + Location  + IVH | Biomarkers not reported in mean or median |
| 240 | Zheng et al. (2018)  China | Zheng GR, Chen B, Shen J, et al. Serum myeloperoxidase concentrations for outcome prediction in acute intracerebral hemorrhage. Clin Chim Acta. 2018;487:330-336. doi:10.1016/j.cca.2018.10.026 | **Total** | **Inclusion**  + Patients with acute non-traumatic basal ganglia hemorrhage  + PResentd within 24h  + Age > 18 years  + Not secondary cause hemorrhage  **Exclusion**  + Acute or chronic infection within 4 weeks before ICH  + Previous ischemic or hemorrhagic stroke  + Severe head trauma  + Use of antiplatelet or anticoagulant medication  + Concurrent comorbidities  + underwent surgical procedure | sICH all site | END  7-day mortality  6-mo mortality  6-mo functional outcome | NIHSS > 4  Mortality  mRS  0-2 good  3-6 poor | Myeloperoxidase | Dot plot | Not stated | Biomarker is not reported in other tudies |
| 241 | Zheng et al. (2023)  China | Zheng P, Wang X, Chen J, Wang X, Shi SX, Shi K. Plasma Neurofilament Light Chain Predicts Mortality and Long-Term Neurological Outcomes in Patients with Intracerebral Hemorrhage. Aging Dis. 2023;14(2):560-571. Published 2023 Apr 1. doi:10.14336/AD.2022.21020 | **Total** | **Inclusion**  + Age > 18 years  + First- episode ICH within 24h  **Exclusion**  + Primary SAH  + Hemorrhage transformation of CI  + Hemorrhage after thrombolysis  + Traumatic cerebral hemorrhage  + Subdural hematoma  + Neurological diseases | All cause ICH | 1, 3, 6, 12-mo functional outcome | mRS  0-2 good  3-6 poor | Neurofilament light chain | ROC curve | Not stated | Biomarkers not reported in mean or median |
| 242 | Zhou et al. (2022)  China | Zhou Y, Dong W, Wang L, Ren S, Wei W, Wu G. Lower serum cystatin C level predicts poor functional outcome in patients with hypertensive intracerebral hemorrhage independent of renal function. *J Clin Hypertens (Greenwich)*. 2023;25(1):86-94. doi:10.1111/jch.14609 | **Total** | **Inclusion**  + Definitive history of hypertension  + Typical bleeding sites of HICH basal ganglia, thalamus, brainstem, cerebrum, cerebellum  + Admission within 72h  + Estimated eGFR > 60mL/min/1.73m^2^  + Age > 18 years  **Exclusion**  + Any other type of stroke  + Systemic comordbidities  + Chronic diseases  + Severe organic brain disorder | All cause ICH | 90-day functional outcome | mRS  0-2 good  3-6 poor | Serum Cystatin C | Odds ratio | Not stated | Biomarkers not reported in mean or median |
| 243 | Zhuge et al. (2022)  China | Zhuge CJ, Zhan CP, Wang KW, Yan XJ, Yu GF. Serum Sulfonylurea Receptor-1 Levels After Acute Supratentorial Intracerebral Hemorrhage: Implication for Prognosis. *Neuropsychiatr Dis Treat*. 2022;18:1117-1126. Published 2022 Jun 3. doi:10.2147/NDT.S368123 | **Total** | **Inclusion**  + First ever supratentorial ICH  + Age 18 year or more  + Admission within 24h  **Exclusion** + Secondary brain bleeding  + Surgical evacuation of hematoma  + Primary IVH  + Presence of previous neurological diseases  + Coexistence of severe systemic disease | Supratentorial ICH | 90-day functional outcome | mRS  0-2 good  3-6 poor | WBC  Glucose  CRP  SUR1 | Median (range) | + Age  + Sex  + HTN  + DM  + Hyperlipidemia  + Smoking  + Alcohol  + Statin  + Anticoagulation drugs  + Antiplatelet |  |
| 244 | Zweifell et al. (2021)  Switzerland | Zweifel C, Katan M, Schuetz P, et al. Growth hormone and outcome in patients with intracerebral hemorrhage: a pilot study. Biomarkers. 2011;16(6):511-516. doi:10.3109/1354750X.2011.599074 | **Total** | **Inclusion**  + Pressumed sICH stroke  **Exclusion**  + Traumatic, SAH | ICH all site | 30-day mortality  90-day functional outcome | Mortality  mRS  0-2 good  3-6 poor | Glucose  WBC  CRP  GH | Median (Range) | + Age  + Sex  + HTN  + DM  + | Not excluding secondary ICH |
| 245 | Amer et al. (2023) Egypt | Amer HA, El-Jaafary SIM, Sadek HMAE, Fouad AM, Mohammed SS. Clinical and paraclinical predictors of early neurological deterioration and poor outcome in spontaneous intracerebral hemorrhage. *Egypt J Neurol Psychiatr Neurosurg*. 2023;59(1):74. doi:10.1186/s41983-023-00675-x | **Total** 70 | **Inclusion:**  + Age > 18 years  + CT evidence of spontaneous ICH  + First symptom onset within 72h  **Exclusion:**  + Post-traumatic hematoma  + Intracranial space-occupying lesion with bleeds  + Hemorrhagic transformation of ischemic stroke + SAH  + Subdural and extradural hematoma  + CNS infection  + Presented after 72h of onset after ICH  + Diagnosed with corona virus infection | All sites | Presence of END  90-day functional outcome | mRS 0-2 good  3-6 poor | N/L Ratio  Urea  Creatinine  AST  Cholesterol  HDL-C | Mean (SD) | Age  GCS Score  NIHSS  GCS FU  NIHSS FU  ICH  ICH FUNC  Hematoma size |  |
| 246 | Feng et al. (2023) | Feng H, Wang X, Wang W, Zhao X. Risk factors and a prediction model for the prognosis of intracerebral hemorrhage using cerebral microhemorrhage and clinical factors. *Front Neurol*. 2023;14:1268627. Published 2023 Nov 23. doi:10.3389/fneur.2023.1268627 | **Total** 269 | **Inclusion:**  + Patient age > 18 years  + Firstly presenting with onset of ICH  + Admitted within 24 hour  + Diagnosis of ICh based on CT  + Available information regarding CM, clinical factor, mRS at 3 and 12 months  **Exclusion:**  + Primary IVH  + Diagnosed with secondary ICH including head trauma, brain tumor, aneurysm, cavernous hemangioma, AVM, acute thrombolysis, coagulopathy, moya moya disease  + Incomplete information of CM or outcome | Lobar  Basal ganglia  Thalamus  Brainstem  Ecencephalon | 90-day functional outcome | mRS  0-2 good  3-6 poor | WBC  Platelet  Glucose  INR  Creatinine  BUN  TC  TG  HDL  LDL  ALT AST  ALP | Median (range) | + Age  + Sex  + Smoking  + Alcohol  + Antiplatelet  + Antihypertensive  + Lipid lowering  + Antidiabetic  + NIHSS  + Location |  |
| 247 | Hernandez et al. (2024) | Hernandez EF, Go CJT, Collantes MEV. Admission Neutrophil-to-Lymphocyte Ratio as a Predictive Factor in the Outcome of Acute Spontaneous Intracerebral Hemorrhage. *Acta Med Philipp*. 2024;58(15):61-66. Published 2024 Aug 30. doi:10.47895/amp.vi0.7456 | **Total** 151 | **Inclusion** + Patient age >18 years  + sICH  + Admitted within 24hours  **Exclusion** + Myeloploriverative disease  + History of head trauma  + Immunocompromised state  + Autoimmune disorder  + Heart failure  + Anticoagulant  + Liver cirrhosis  + Renal dysfunction  + Secondary ICH due to AVM, aneurysm, etc. | All sites | 7-day mortality | Mortality | NLR | Cutoff (7) | + Hypertension  + DM  + Alcohol  + Smoking  + Blood pressure  + GCS + NIHSS  + SAH + IVH + ICH volume | Biomarkers not reported in mean or median |
| 248 | Huang et al. (2024) | Huang J, Shao F, Chen B, Zheng G, Shen J, Qiu S. Serum Secreted Protein Acidic and Rich in Cysteine-Like 1 as a Biochemical Predictor for Prognosticating Clinical Outcomes After Acute Supratentorial Intracerebral Hemorrhage: A Prospective Cohort Study [published correction appears in Neuropsychiatr Dis Treat. 2024 May 29;20:1179-1180. doi: 10.2147/NDT.S480071]. *Neuropsychiatr Dis Treat*. 2023;19:2709-2728. Published 2023 Dec 5. doi:10.2147/NDT.S444671 | **Total** 156 | **Inclusion**  + Age > 18 years  + Fist time ICH  + Supratentorial bleedings  + Primary ICH but not primary IVH  + Non-surgical treatment for intracerebral hematoma  + Hospitalization within 24h  **Exclusion** + Other neurological diseases  + Other sever diseases in other organs  + Pregnancies | Supratentorial | 6-month functional score | mRS 0-2 good  3-6 poor | Leukocyte  Glucose  SPARC1 | Median (range) | Age  Gender  HT DM  Dyslipidemia  Cigarette  Alcohol  Statin  Anticoagulant  Antiplatelet  Hospital admission  IVH  SAH |  |
| 249 | Ismail et al. (2024) | Ismail MH, Ibrahim GAE, Elaidy SA. Serum biomarkers predicting prognosis of spontaneous intracerebral hemorrhage. Zagazig Univ Med J 2024; 30(7): 3298-3307. DOI: 10.21608/ZUMJ.2024.303983.3473 | **Total** 138 | **Inclusion** + Age >18 years  + ICH diagnosed with CT  **Exclusion**  + Traumatic brain hemorrhage  + Aneurysm  + AVM  + Brain tumors  + Systemic disease | All sites | 3-month functional score | mRS 0-2 good  3-6 poor | Platelet count  NPR  Cholesterol  LDL-C  INR | Median (range) | Gender  BMI  Hypertension  Diabetes  Dyslipidemia  IHD  Smoking  GCS  NIHSS mRS admission  Hemorrhage volume |  |
| 250 | Kim et al. (2023) | Kim Y, Sohn JH, Kim C, Park SY, Lee SH. The Clinical Value of Neutrophil-to-Lymphocyte Ratio and Platelet-to-Lymphocyte Ratio for Predicting Hematoma Expansion and Poor Outcomes in Patients with Acute Intracerebral Hemorrhage. *J Clin Med*. 2023;12(8):3004. Published 2023 Apr 20. doi:10.3390/jcm12083004 | **Total** 520 | **Inclusion**  + Age > 18 years  + Brain CT  **Exclusion** + Secondary ICH to brain tumor, trauma, Moyamoya AVM, aneurysmal  +Coagulopathy  + Drug abuse  + Hemorrhagic transformation  + Immunomodulatory management before ICH + Preexisting autoimmune or infectious disease | All sites | 1-month mortality  3-month functional outcome | Mortality  mRS 0-2 good  3-6 poor | INR | Odds ratio | None | Biomarkers not reported in mean or median |
| 251 | Lee et al. (2023) | Lee SH, Sohn JH, Kim C, et al. Pre-stroke glycemic variability estimated by glycated albumin predicts hematoma expansion and poor outcomes in patients with spontaneous intracerebral hemorrhage. *Sci Rep*. 2023;13(1):12848. Published 2023 Aug 8. doi:10.1038/s41598-023-40109-5 | **Total** 343 | **Inclusion** + | All sites | Hematoma expansion  END  1-month mortality  3-month functional outcome | mRS  0-3 good  4-6 poor | Glycated albumin  LDL  Glucose  HbA1c | Odds ratio | Age  Gender  GCS  NIHSS Prior stroke  HT DM  Antithrombotic | Biomarkers not reported in mean or median |
| 252 | Li et al. (2024) | Li S, Zhang J, Hou X, et al. Prediction Model for Unfavorable Outcome in Spontaneous Intracerebral Hemorrhage Based on Machine Learning. *J Korean Neurosurg Soc*. 2024;67(1):94-102. doi:10.3340/jkns.2023.0118 | **Total** 227 | **Inclusion**  + ICH patients identified by CT or MRI  + Treated with surgery (including minimally invasive aspiration and craniotomies)  **Exclusion:**  + Secondary Ich by aneurysm, AVM, tumor  + Severe end-organ failure  + Infections  + Blood disorders  + Lost to follow-up | All sites | 6-month functional outcome | mRS  0-2 good  3-6 poor | None | None | Age  Sex  BMI  DM  HT Hyperlipidemia  GCS  Location  Midline shift  Hematoma volume  Operation type | Not reporting biomarkers |
| 253 | Liang et al. (2023) | Liang Z, Liu H, Xue L, et al. A retrospective study about association of dynamic systemic immune-inflammation index (SII) with 180-day functional outcome after basal ganglia intracerebral hemorrhage. *Heliyon*. 2023;9(6):e16937. Published 2023 Jun 8. doi:10.1016/j.heliyon.2023.e16937 | **Total** 245 | **Inclusion**  + Age > 18 years  + Basal ganglia ICH based on CT scans  + Admitted < 24h  **Exclusion**  + Aneurysm, AVM, tumor fistula  + Infection, autoimmune, hematologic disease  + Missing data  + Coagulopathy  + Active infection | Basal ganglia | 6-month functional outcome | mRS  0-3 good  4-6 poor | Neutrophil  Lymphocyte  Platelet  Monocyte  LMR  NLR  PLR SII index | Median (range) | Age  Sex  GCS  HT DM  Smoking  Hyperlipidemia  Drinking  Surgery  ICH Volume  IVH | mRS outcome criteria is not the same with other studies |
| 254 | Liu et al. (2024) | Liu Y, Qiu T, Fu Z, et al. Systemic immune-inflammation index and serum glucose-potassium ratio predict poor prognosis in patients with spontaneous cerebral hemorrhage: An observational study. *Medicine (Baltimore)*. 2024;103(29):e39041. doi:10.1097/MD.0000000000039041 | **Total** 105 | **Inclusion**  + Age > 18 years  + Onset time < 12 h  + Blood test within 12 h  **Exclusion** + Traumatic brain injury  + Secondaray ICH (AVM, tumor, aneurysm)  + Metabolic disease  + Hematologic disease  + Surgical treatment | All sites | 3-month functional outcome | mRS  0-3 good  4-6 poor | Hb  RBC  Neutrophil  Lymphocyte  Monocyte  Platelet  CRP  Glucose  K  Na  Ca  Mg  SII score  GPR  NLR  LMR  PLR | Median (range | Sex  Age  BMI  Smoking  Drinking  HT  Stroke  Statin  Antiplatelet  Anticoagulant  Hematoma volume  IVH  Site  GCS  NIHSS | mRS outcome criteria is not the same with other studies |
| 255 | Lv et al. (2023) | Lv XN, Shen YQ, Li ZQ, et al. Neutrophil percentage to albumin ratio is associated with stroke-associated pneumonia and poor outcome in patients with spontaneous intracerebral hemorrhage. *Front Immunol*. 2023;14:1173718. Published 2023 Jun 14. doi:10.3389/fimmu.2023.1173718 | **Total** 918 | **Inclusion**  + Age > 18 years  + Acute sICH  + Baseline CT  **Exclusion**  + Primary IVH  + Secondary ICH  + Traumatic ICH + Lost to follow up  + Surgical treatment | All sites | 3-month functional outcome | mRS  0-3 good  4-6 poor | Hb  WBC  Neutrophil percentage  NPR | Mean (SD)  Median (IQR) | Age  Sex  Smoking  Alcohol  HT DM  Stroke history  IVH  Basal ganglia  Pneumonia | mRS outcome criteria is not the same with other studies |
| 256 | Ma et al. (2024) | Ma Y, Wang J, Tang C, Li W, Lv X, Zhu S. Serum IRAK3 may serve as a prognostic biomarker in acute supratentorial intracerebral hemorrhage: findings from a prospective observational cohort study. *Front Neurol*. 2024;15:1436997. Published 2024 Sep 13. doi:10.3389/fneur.2024.1436997 | **Total** 152 | **Inclusion**  + Age > 18 years  + Admitted within 24h  + New onset hemorrhagic stroke  + Non secondary brain hemorrhage  + Hemorrhage at supratentorial cavity  **Exclusion** + History of concurrent neurological disase  + Severe comorbidities  Such as leukemia, COPD, liver cirrhosis, heart failure, renal disease  + Specific conditions or medications | Supratentorial | 6-month functional outcome | mRS | IRAK3 | Boxplot | None | Biomarker is not reported at other studies |
| 257 | Mishra et al. (2024) | Misra S, Kawamura Y, Singh P, et al. Prognostic biomarkers of intracerebral hemorrhage identified using targeted proteomics and machine learning algorithms. *PLoS One*. 2024;19(6):e0296616. Published 2024 Jun 3. doi:10.1371/journal.pone.0296616 | **Total** 150 | **Inclusion**  + Patients age > 18 years  + Admitted within 24hours | All sites | 90-day functional outcome  180-day functional outcome  90-day mortality  180-day mortality | mRS 0-2 good  3-6 poor | RBS TLC Hemoglobin  Platelets  BUN  Creatinine  Potassium  Sodium  Bilirubin | Median (IQR) | Age  Sex  Admission time  Surgery  HT  DM  ACE inhibitor  ARB  Beta blocker  Calcium channel blocker  Diuretic  Dyslipidemia  MI  AF  Smoking  Alcohol  No exercise  Obesity  ICH volume  IVH  Location  NIHSS GCS | Included cases of traumatic and secondary ICH |
| 258 | Pereira et al. (2023) | Pereira M, Batista R, Marreiros A, Nzwalo H. Neutrophil-to-leukocyte ratio and admission glycemia as predictors of short-term death in very old elderlies with lobar intracerebral hemorrhage. *Brain Circ*. 2023;9(2):94-98. Published 2023 Jun 30. doi:10.4103/bc.bc_5_23 | **Total** | **Inclusion**  + Patients with sICH lobar  + Age > 75 years  **Exclusion**  + Non lobar location + Macrovascular causes sICH (DVM, cavernoma, tumor, malformation, hemorrhagics transformation)  + Neurosurgical treatments | Lobar | 30-day mortality | Mortality | Glucose  RDS Neutrophils  Lymphocyte  NLR Platelet  MPV  PDW  Sodium  Potassium  BUN  Creatinine  Troponin I  CRP | Mean (SD) | Dyslipidemia  HT DM  AF  Hypocoagulation | Included patients with infections |
| 259 | Ray et al. (2024) | Ray S, Kumar V, Biswas R, et al. Neutrophil-to-Lymphocyte Ratio as a Prognostic Marker of Functional Outcome in Patients With Intracerebral Hemorrhage (ICH) and Its Comparison With ICH Score: A Hospital-Based Study. *Cureus*. 2024;16(9):e69350. Published 2024 Sep 13. doi:10.7759/cureus.69350 | **Total** 120 | **Inclusion**  + Patients with acute non-traumatic ICH  + Age > 18 years  + Presented within 24h of onset  + Hemorrhage confirmed by CT  **Exclusion**  + Head trauma  + Fever  + Signs of infection within 4 weeks  + Presence of subdural or epidural hematoma  + Known AVM | All sites | 90-day functional outcome | mRS 0-2 goos  3-6 poor | NLR | AUC | ICH score | Biomarker is not reported in meanor median |
| 260 | Wang et al. (2023) | Wang JY, Wilson M, Andreev A, Tarsia J, Selim M, Lioutas VA. The role of hyperglycemia in the outcome of intracerebral hemorrhage: A causative analysis. *J Stroke Cerebrovasc Dis*. 2023;32(12):107439. doi:10.1016/j.jstrokecerebrovasdis.2023.107439 | **Total** 410 | **Inclusion**  + Patients with primary ICH  + Admitted within 24h  **Exclusion**  + Baseline CT scan > 24h | All sites | 90-day functional outcome | mRS  0-2 good  3-6 poor | Glucose | Regression | Age  Hematoma volume  IVE  Hematoma expansion  Location  GCS | Biomarker is not reported in meanor median |
| 261 | Wang et al. (2024) | Wang D, Zhang J, Dong H, et al. Enhancing Outcome Prediction in Intracerebral Hemorrhage Through Deep Learning: A Retrospective Multicenter Study. *Acad Radiol*. Published online August 1, 2024. doi:10.1016/j.acra.2024.07.025 | **Total** 1098 | **Inclusion**  + ICH patients  + Admitted within 6h  + CT scan within 6h  **Exclusion**  + Surgery before CT  + Secondary ICH  + Tumor  + Trauma  +Cerebral aneurysm  + Infection  + Problematic image quality | Deep  Lobar | 90-day functional outcome | mRS  0-3 good  4-6 poor | Glucose  Triglyceride  WBC Neutrophil  Lymphocyte  NLR HGB  INR | Odds ratio | Sex  Age  Location  Midline shift  IVH  SAH  Hypodensities  IVH  SAH  ICH volume  PHE volume  Temperature  Smoking  SBP  GCS | Biomarkers are not reported in mean or median |
| 262 | Wang et al. (2024) | Wang J, Chen D, Tang Y, Sherchan P, Wang J, Zhang P, et al. Relationships between NT-pro-BNP and other serological indicators and the prognosis of spontaneous intracerebral hemorrhage, Brain Hemorrhages, https://doi.org/10.1016/j | **Total** 121 | **Inclusion**  + Acute sICH confirmed by CT  + Admission within 48 hourse  + Age between 18-85  + Survival for at least 7 days after admission  **Exclusion**  + Trauma  + Secondary ICH  + SAH  + Brain tumor  + Craniotomy  + Pregnancy  + History of heart diseases  + History of renal failure  + Multiple organ dysnfunction | All sites | 90-day functional outcome | mRS  0-2 good  3-6 poor | NT-pro BNP  Glucose  WBC  Hb  ALT  AST  Creatinine  CRP  IL-6  CTNI | Median (IQR) | Sex  Age  Hematoma vol  GCS  Temperature  HR  SBP  DBP |  |
| 263 | Witsch (2023) | Witsch J, Roh D, Oh S, et al. Association Between Soluble Intercellular Adhesion Molecule-1 and Intracerebral Hemorrhage Outcomes in the FAST Trial. *Stroke*. 2023;54(7):1726-1734. doi:10.1161/STROKEAHA.123.042466 | **Total** 507 | **Inclusion**  + Age > 18 years  + sICH on CT within 3 hrs of symptoms | All sites | 90-days functional outcome | mRS 0-3 good  4-6 Poor | SICAM-1  IL-6  MMP-3  MMP-9  TNF-alpha | Mean (SD) | Age  Sex  HT  DM  Hyperlipidemia  CAD  AF  Prior ischemic stroke or TIA  ICH vol  PHE vol  IVH vol  Presence of IVH  Location  Expansion | mRS criteria is not the same as other studies |
| 264 | Wu et al. 2023 | Wu M, Chen K, Jiang M, et al. High plasma complement C4 levels as a novel predictor of clinical outcome in intracerebral hemorrhage. *Front Aging Neurosci*. 2023;15:1103278. Published 2023 Feb 20. doi:10.3389/fnagi.2023.1103278 | **Total** 83 | **Inclusion**  + Age >18 years  + Admitted within 24h  **Exclusion**  + surgical treatment  + Secondary ICH  + Pre-ICH mRS > 2  + Severe infections  + Malignancies  +Autoimmune | All sites | Hematoma volume  NIHSS score  GCS score |  | Glucose  Potassium  Leukocyte  C4 | Regression | Age  Sex  HT  DM  Smoking  Alcohol | Not reporting outcome of interest |
| 265 | Wu et a. 2024 | Wu M, Chen K, Jiang M, et al. High plasma complement C4 levels as a novel predictor of clinical outcome in intracerebral hemorrhage. Front Aging Neurosci. 2023;15:1103278. Published 2023 Feb 20. doi:10.3389/fnagi.2023.1103278 | **Total** 148 | **Inclusion**  + Acute sICH confirmed by CT  + > 18 years  + Hospitalized within 24h  **Exclusion**  + Secondary ICH  + Coagulation disorders  + Severe organ complications  + Lost to follow-up | All sites | 90-day functional outcome | mRS  0-2 good  3-6 poor | ALT  AST  TG  TC LDL-C APTT PT  INR  Fibrinogen  WBC  NLR  Platelet  Glucose  ALP | Mean (SD) | Age  Sex  Smoking  Alcohol  HT DM  Hyperlipidemia  HF  Artery disease  Location  IVE |  |
| 266 | Xu et al. (2024) | Xu M, Wang J, Zhan C, et al. Association of follow-up neutrophil-to-lymphocyte ratio and systemic inflammation response index with stroke-associated pneumonia and functional outcomes in cerebral hemorrhage patients: a case-controlled study. *Int J Surg*. 2024;110(7):4014-4022. Published 2024 Jul 1. doi:10.1097/JS9.0000000000001329 | **Total** 451 | **Inclusion**  + sICH with CT within 6-72 h  **Exclusion**  + Secondary ICH  + Severe organ diseases  + Immunosuppressants > 3 months  + Diagnosed with pneumonia or active infection  + Lack of determined primary endpoint | All sites | Patient with SAP |  | Leukocyte  Platelet  Neu  Mono  Lymp | Mean (SD) | Age  Sex  Smoking  Alcohol  HT  DM  Previous stroke  Clinical status  GCS score  Bradden score | Not reporting outcome of interest |
| 267 | Geng et al. (2023) | Geng Y, Wang T, Liu Y, et al. How to predict the outcome of primary brainstem hemorrhage: Six-year results of a single-center retrospective analysis. *Medicine (Baltimore)*. 2023;102(37):e35131. doi:10.1097/MD.0000000000035131 | **Total** 63 | **Inclusion**  + Patients with primary brainstem hemorrhage | Brainstem | 30-day mortality  90-day functional recovery | Mortality  mRS  0-3 good  4-6 poor | WBC  NLR high | Mean (SD) | Age  Sex  SBP  Smoking  MV  HT  DM  Volume  IVH  Surgery |  |
| 268 | Yu et al. (2023) | Yu W, Peng J, Chen Z, et al. Association of plasma MMP-2 levels and prognosis of patients with intracerebral hemorrhage: a prospective cohort study. *Front Neurol*. 2023;14:1259339. Published 2023 Nov 27. doi:10.3389/fneur.2023.1259339 | **Total** 93 | **Inclusion**  + Diagnosed with acute sICH by CT within 24h  **Exclusion**  + Age < 20 years  + Needes surgical treatment  + Infratentorial bleeding  + Secondary ICH  + Coagulation disorder  + Pregnant  + Had pre-ICH by mRS >2 before ICH  + Sever infections  + Severe organ diseases | Supratentorial | Edema volume  NIHSS  GCS |  | MMP-2 MMP-14 | Regression |  | Not reporting outcome of interest |
