# Supplementary material for: Potential blood biomarkers that can be used as prognosticators of spontaneous intracerebral hemorrhage: A systematic review and meta-analysis
Source: PLoS One. 2025 Feb 19;20(2):e0315333. doi: 10.1371/journal.pone.0315333 (PMC11838903; doi:10.1371/journal.pone.0315333)
Supplement: S2 Table — (DOCX) [file pone.0315333.s004.docx]

**Supplement Table 2.** GRADE Profile Evidence

**Evidence for Mortality**

**Inflammatory Biomarkers**

1. C-Reactive Protein

| **Certainty assessment** | | | | | | | **№ of patients** | | **Effect** | | **Certainty** | **Importance** |
| --- | --- | --- | --- | --- | --- | --- | --- | --- | --- | --- | --- | --- |
| **№ of studies** | **Study design** | **Risk of bias** | **Inconsistency** | **Indirectness** | **Imprecision** | **Other considerations** | **Dead** | **Alive** | **Relative (95% CI)** | **Absolute (95% CI)** |  |  |
| **7-day mortality** | | | | | | | | | | | | |
| 5 | non-randomised studies | not serious | serious^a^ | not serious | not serious | strong association | 149 | 480 | - | MD **6.01 higher** (1.68 higher to 10.34 higher) | ⨁⨁◯◯ Low |  |
| **30-day mortality** | | | | | | | | | | | | |
| 6 | non-randomised studies | not serious | not serious | not serious | not serious | strong association | 278 | 779 | - | MD **2.63 higher** (2.22 higher to 3.04 higher) | ⨁⨁⨁◯ Moderate |  |
| **3-month mortality** | | | | | | | | | | | | |
| 4 | non-randomised studies | not serious | serious^a^ | not serious | not serious | strong association | 147 | 478 | - | MD **3.48 higher** (1.33 higher to 5.62 higher) | ⨁⨁◯◯ Low |  |
| **6-month mortality** | | | | | | | | | | | | |
| 4 | non-randomised studies | not serious | not serious | not serious | not serious | none | 165 | 337 | - | MD **3.23 higher** (1.66 higher to 4.8 higher) | ⨁⨁◯◯ Low |  |

a. Heterogeneity > 75% but the direction of the effect is the same

**Coagulation Parameters**

1. Prothrombin Time

| **Certainty assessment** | | | | | | | **№ of patients** | | **Effect** | | **Certainty** | **Importance** |
| --- | --- | --- | --- | --- | --- | --- | --- | --- | --- | --- | --- | --- |
| **№ of studies** | **Study design** | **Risk of bias** | **Inconsistency** | **Indirectness** | **Imprecision** | **Other considerations** | **Dead** | **Alive** | **Relative (95% CI)** | **Absolute (95% CI)** |  |  |
| **7-day mortality** | | | | | | | | | | | | |
| 5 | non-randomised studies | not serious | not serious | not serious | extremely serious^a^ | none | 127 | 504 | - | MD **0.15 lower** (0.76 lower to 0.46 higher) | ⨁◯◯◯ Very low |  |
| **6-month mortality** | | | | | | | | | | | | |
| 3 | non-randomised studies | not serious | not serious | not serious | extremely serious^a^ | none | 131 | 279 | - | MD **0.11 higher** (0.38 lower to 0.59 higher) | ⨁◯◯◯ Very low |  |

a. Number of cohort < 30% Optimum Information Size

1. Thrombin Time

| **Certainty assessment** | | | | | | | **№ of patients** | | **Effect** | | **Certainty** | **Importance** |
| --- | --- | --- | --- | --- | --- | --- | --- | --- | --- | --- | --- | --- |
| **№ of studies** | **Study design** | **Risk of bias** | **Inconsistency** | **Indirectness** | **Imprecision** | **Other considerations** | **Dead** | **Alive** | **Relative (95% CI)** | **Absolute (95% CI)** |  |  |
| **Thrombin 7-day** | | | | | | | | | | | | |
| 3 | non-randomised studies | not serious | not serious | not serious | extremely serious^a^ | none | 67 | 207 | - | MD **0.49 higher** (0.23 lower to 1.22 higher) | ⨁◯◯◯ Very low |  |
| **Thrombin 6-month mortality** | | | | | | | | | | | | |
| 3 | non-randomised studies | not serious | not serious | not serious | extremely serious^a^ | none | 131 | 279 | - | MD **0.02 higher** (0.68 lower to 0.71 higher) | ⨁◯◯◯ Very low |  |

a. Number of cohort < 30% Optimum Information Size

1. Activated Partial Thromboplastin Time

| **Certainty assessment** | | | | | | | **№ of patients** | | **Effect** | | **Certainty** | **Importance** |
| --- | --- | --- | --- | --- | --- | --- | --- | --- | --- | --- | --- | --- |
| **№ of studies** | **Study design** | **Risk of bias** | **Inconsistency** | **Indirectness** | **Imprecision** | **Other considerations** | **Dead** | **Alive** | **Relative (95% CI)** | **Absolute (95% CI)** |  |  |
| **7-day mortality** | | | | | | | | | | | | |
| 5 | non-randomised studies | not serious | serious^a^ | not serious | extremely serious^b^ | none | 127 | 504 | - | MD **0.13 higher** (1.21 lower to 1.47 higher) | ⨁◯◯◯ Very low |  |
| **6-month mortality** | | | | | | | | | | | | |
| 4 | non-randomised studies | not serious | very serious^c^ | not serious | extremely serious^b^ | none | 273 | 618 | - | MD **0.34 higher** (1.11 lower to 1.78 higher) | ⨁◯◯◯ Very low |  |

a. Heterogeneity > 50% with different directions between studies

b. Number of cohort < 30% Optimum Information Size

c. Heterogeneity > 75% with different directions between studies

1. Fibrinogen

| **Certainty assessment** | | | | | | | **№ of patients** | | **Effect** | | **Certainty** | **Importance** |
| --- | --- | --- | --- | --- | --- | --- | --- | --- | --- | --- | --- | --- |
| **№ of studies** | **Study design** | **Risk of bias** | **Inconsistency** | **Indirectness** | **Imprecision** | **Other considerations** | **Dead** | **Alive** | **Relative (95% CI)** | **Absolute (95% CI)** |  |  |
| **7-day mortality** | | | | | | | | | | | | |
| 6 | non-randomised studies | not serious | not serious | not serious | not serious | none | 132 | 528 | - | MD **0.32 higher** (0.03 lower to 0.68 higher) | ⨁⨁◯◯ Low |  |
| **3-month mortality** | | | | | | | | | | | | |
| 5 | non-randomised studies | not serious | serious^a^ | not serious | not serious | none | 174 | 537 | - | MD **0.33 higher** (0.17 lower to 0.83 higher) | ⨁◯◯◯ Very low |  |
| **6-month mortality** | | | | | | | | | | | | |
| 3 | non-randomised studies | not serious | not serious | not serious | extremely serious^b^ | none | 131 | 279 | - | MD **0.08 higher** (0.42 lower to 0.58 higher) | ⨁◯◯◯ Very low |  |

a. Heterogeneity > 75% but the direction of the effect is the same

b. Number of cohort < 30% Optimum Information Size

1. D-Dimer

| **Certainty assessment** | | | | | | | **№ of patients** | | **Effect** | | **Certainty** | **Importance** |
| --- | --- | --- | --- | --- | --- | --- | --- | --- | --- | --- | --- | --- |
| **№ of studies** | **Study design** | **Risk of bias** | **Inconsistency** | **Indirectness** | **Imprecision** | **Other considerations** | **Dead** | **Alive** | **Relative (95% CI)** | **Absolute (95% CI)** |  |  |
| **7-day mortality** | | | | | | | | | | | | |
| 4 | non-randomised studies | not serious | not serious | not serious | not serious | strong association | 158 | 504 | - | MD **0.84 higher** (0.64 higher to 1.03 higher) | ⨁⨁⨁◯ Moderate |  |
| **3-month mortality** | | | | | | | | | | | | |
| 2 | non-randomised studies | not serious | not serious | not serious | not serious | none | 88 | 243 | - | MD **0.39 higher** (0.12 higher to 0.65 higher) | ⨁⨁◯◯ Low |  |
| **6-month mortality** | | | | | | | | | | | | |
| 3 | non-randomised studies | not serious | not serious | not serious | not serious | none | 121 | 231 | - | MD **0.5 higher** (0.2 higher to 0.79 higher) | ⨁⨁◯◯ Low |  |

**Blood Counts**

1. White Blood Cells

| **Certainty assessment** | | | | | | | **№ of patients** | | **Effect** | | **Certainty** | **Importance** |
| --- | --- | --- | --- | --- | --- | --- | --- | --- | --- | --- | --- | --- |
| **№ of studies** | **Study design** | **Risk of bias** | **Inconsistency** | **Indirectness** | **Imprecision** | **Other considerations** | **Dead** | **Alive** | **Relative (95% CI)** | **Absolute (95% CI)** |  |  |
| **7-day mortality** | | | | | | | | | | | | |
| 7 | non-randomised studies | not serious | not serious | not serious | not serious | none | 194 | 625 | - | MD **1.61 higher** (0.61 higher to 2.61 higher) | ⨁⨁◯◯ Low |  |
| **30-day mortality** | | | | | | | | | | | | |
| 6 | non-randomised studies | not serious | not serious | not serious | not serious | strong association | 260 | 494 | - | MD **2.49 higher** (2.12 higher to 2.87 higher) | ⨁⨁⨁◯ Moderate |  |
| **3-month mortality** | | | | | | | | | | | | |
| 8 | non-randomised studies | not serious | serious^a^ | not serious | not serious | none | 401 | 1227 | - | MD **1.13 higher** (0.23 higher to 2.04 higher) | ⨁◯◯◯ Very low |  |
| **6-mo mortality** | | | | | | | | | | | | |
| 5 | non-randomised studies | not serious | serious^a^ | not serious | not serious | none | 289 | 684 | - | MD **1.56 higher** (0.12 higher to 3.01 higher) | ⨁◯◯◯ Very low |  |

a. Heterogeneity > 75% but the direction of the effect is the same

1. Neutrophil

| **Certainty assessment** | | | | | | | **№ of patients** | | **Effect** | | **Certainty** | **Importance** |
| --- | --- | --- | --- | --- | --- | --- | --- | --- | --- | --- | --- | --- |
| **№ of studies** | **Study design** | **Risk of bias** | **Inconsistency** | **Indirectness** | **Imprecision** | **Other considerations** | **Dead** | **Alive** | **Relative (95% CI)** | **Absolute (95% CI)** |  |  |
| **3-month Mortality** | | | | | | | | | | | | |
| 3 | non-randomised studies | not serious | very serious^a^ | not serious | not serious | none | 158 | 472 | - | MD **2.06 higher** (0.98 lower to 5.09 higher) | ⨁◯◯◯ Very low |  |

a. Heterogeneity > 75% with different directions between studies

1. Lymphocyte

| **Certainty assessment** | | | | | | | **№ of patients** | | **Effect** | | **Certainty** | **Importance** |
| --- | --- | --- | --- | --- | --- | --- | --- | --- | --- | --- | --- | --- |
| **№ of studies** | **Study design** | **Risk of bias** | **Inconsistency** | **Indirectness** | **Imprecision** | **Other considerations** | **Dead** | **Alive** | **Relative (95% CI)** | **Absolute (95% CI)** |  |  |
| **30-day Mortality** | | | | | | | | | | | | |
| 2 | non-randomised studies | not serious | not serious | not serious | extremely serious^a^ | none | 43 | 135 | - | MD **0.08 lower** (0.26 lower to 0.11 higher) | ⨁◯◯◯ Very low |  |
| **3-month mortality** | | | | | | | | | | | | |
| 2 | non-randomised studies | not serious | very serious^b^ | not serious | not serious | none | 107 | 272 | - | MD **0.2 lower** (0.58 lower to 0.18 higher) | ⨁◯◯◯ Very low |  |

a. Number of cohort < 30% Optimum Information Size

b. Heterogeneity > 75% with different directions between studies

1. Monocyte

| **Certainty assessment** | | | | | | | **№ of patients** | | **Effect** | | **Certainty** | **Importance** |
| --- | --- | --- | --- | --- | --- | --- | --- | --- | --- | --- | --- | --- |
| **№ of studies** | **Study design** | **Risk of bias** | **Inconsistency** | **Indirectness** | **Imprecision** | **Other considerations** | **Dead** | **Alive** | **Relative (95% CI)** | **Absolute (95% CI)** |  |  |
| **3-month mortality** | | | | | | | | | | | | |
| 2 | non-randomised studies | not serious | not serious | not serious | not serious | none | 107 | 272 | - | MD **0.04 higher** (0.02 higher to 0.06 higher) | ⨁⨁◯◯ Low |  |

1. Platelet

| **Certainty assessment** | | | | | | | **№ of patients** | | **Effect** | | **Certainty** | **Importance** |
| --- | --- | --- | --- | --- | --- | --- | --- | --- | --- | --- | --- | --- |
| **№ of studies** | **Study design** | **Risk of bias** | **Inconsistency** | **Indirectness** | **Imprecision** | **Other considerations** | **Dead** | **Alive** | **Relative (95% CI)** | **Absolute (95% CI)** |  |  |
| **7-day mortality** | | | | | | | | | | | | |
| 7 | non-randomised studies | not serious | serious^a^ | not serious | not serious | none | 200 | 671 | - | MD **8.96 higher** (0.37 lower to 18.3 higher) | ⨁◯◯◯ Very low |  |
| **30-day mortality** | | | | | | | | | | | | |
| 3 | non-randomised studies | not serious | serious^a^ | not serious | extremely serious^b^ | none | 91 | 189 | - | MD **0.49 higher** (34.5 lower to 35.47 higher) | ⨁◯◯◯ Very low |  |
| **3-month mortality** | | | | | | | | | | | | |
| 7 | non-randomised studies | not serious | very serious^c^ | not serious | not serious | none | 290 | 871 | - | MD **13.4 higher** (12.05 lower to 38.84 higher) | ⨁◯◯◯ Very low |  |
| **6-month mortality** | | | | | | | | | | | | |
| 3 | non-randomised studies | not serious | not serious | not serious | extremely serious^b^ | none | 131 | 279 | - | MD **1.56 lower** (14.96 lower to 11.85 higher) | ⨁◯◯◯ Very low |  |

a. Heterogeneity > 50% with different directions between studies

b. Number of cohort < 30% Optimum Information Size

c. Heterogeneity > 75% with different directions between studies

**Others**

1. Glucose

| **Certainty assessment** | | | | | | | **№ of patients** | | **Effect** | | **Certainty** | **Importance** |
| --- | --- | --- | --- | --- | --- | --- | --- | --- | --- | --- | --- | --- |
| **№ of studies** | **Study design** | **Risk of bias** | **Inconsistency** | **Indirectness** | **Imprecision** | **Other considerations** | **Dead** | **Alive** | **Relative (95% CI)** | **Absolute (95% CI)** |  |  |
| **7-day mortality** | | | | | | | | | | | | |
| 9 | non-randomised studies | not serious | serious^a^ | not serious | not serious | none | 231 | 822 | - | MD **1.99 higher** (1 higher to 2.98 higher) | ⨁◯◯◯ Very low |  |
| **30-day mortality** | | | | | | | | | | | | |
| 8 | non-randomised studies | not serious | serious^a^ | not serious | not serious | strong association | 314 | 903 | - | MD **3.1 higher** (2.39 higher to 3.81 higher) | ⨁⨁◯◯ Low |  |
| **3-month mortality** | | | | | | | | | | | | |
| 9 | non-randomised studies | not serious | not serious | not serious | not serious | none | 674 | 1726 | - | MD **1.84 higher** (1.31 higher to 2.37 higher) | ⨁⨁◯◯ Low |  |
| **6-mo mortality** | | | | | | | | | | | | |
| 4 | non-randomised studies | not serious | not serious | not serious | not serious | none | 165 | 337 | - | MD **2.57 higher** (1.62 higher to 3.51 higher) | ⨁⨁◯◯ Low |  |

a. Heterogeneity > 75% but the direction of the effect is the same

1. Sodium

| **Certainty assessment** | | | | | | | **№ of patients** | | **Effect** | | **Certainty** | **Importance** |
| --- | --- | --- | --- | --- | --- | --- | --- | --- | --- | --- | --- | --- |
| **№ of studies** | **Study design** | **Risk of bias** | **Inconsistency** | **Indirectness** | **Imprecision** | **Other considerations** | **Dead** | **Alive** | **Relative (95% CI)** | **Absolute (95% CI)** |  |  |
| **30-day mortality** | | | | | | | | | | | | |
| 2 | non-randomised studies | not serious | not serious | not serious | extremely serious^a^ | none | 83 | 154 | - | MD **2.3 higher** (2.11 lower to 6.71 higher) | ⨁◯◯◯ Very low |  |
| **3-month mortality** | | | | | | | | | | | | |
| 2 | non-randomised studies | not serious | very serious^b^ | not serious | not serious | none | 146 | 482 | - | MD **0.92 higher** (0.2 higher to 1.64 higher) | ⨁◯◯◯ Very low |  |
| **7-day Mortality** | | | | | | | | | | | | |
| 2 | non-randomised studies | not serious | not serious | not serious | serious^c^ | none | 47 | 77 | - | MD **0.41 higher** (1.36 lower to 2.19 higher) | ⨁◯◯◯ Very low |  |

a. Number of cohort < 30% Optimum Information Size

b. Heterogeneity > 75% with different directions between studies

c. Number of cohort < 50% Optimum Information Size

1. Potassium

| **Certainty assessment** | | | | | | | **№ of patients** | | **Effect** | | **Certainty** | **Importance** |
| --- | --- | --- | --- | --- | --- | --- | --- | --- | --- | --- | --- | --- |
| **№ of studies** | **Study design** | **Risk of bias** | **Inconsistency** | **Indirectness** | **Imprecision** | **Other considerations** | **Dead** | **Alive** | **Relative (95% CI)** | **Absolute (95% CI)** |  |  |
| **7-day mortality** | | | | | | | | | | | | |
| 2 | non-randomised studies | not serious | not serious | not serious | extremely serious^a^ | none | 47 | 77 | - | MD **0.2 higher** (0 to 0.4 higher) | ⨁◯◯◯ Very low |  |

a. Number of cohort < 30% Optimum Information Size

1. Haemoglobin

| **Certainty assessment** | | | | | | | **№ of patients** | | **Effect** | | **Certainty** | **Importance** |
| --- | --- | --- | --- | --- | --- | --- | --- | --- | --- | --- | --- | --- |
| **№ of studies** | **Study design** | **Risk of bias** | **Inconsistency** | **Indirectness** | **Imprecision** | **Other considerations** | **Dead** | **Alive** | **Relative (95% CI)** | **Absolute (95% CI)** |  |  |
| **7-day mortality** | | | | | | | | | | | | |
| 3 | non-randomised studies | not serious | not serious | not serious | extremely serious^a^ | none | 101 | 306 | - | MD **3.06 lower** (8.77 lower to 2.64 higher) | ⨁◯◯◯ Very low |  |
| **3-month mortality** | | | | | | | | | | | | |
| 3 | non-randomised studies | not serious | very serious^b^ | not serious | extremely serious^a^ | none | 116 | 323 | - | MD **0.06 higher** (6.82 lower to 6.94 higher) | ⨁◯◯◯ Very low |  |
| **6-month mortality** | | | | | | | | | | | | |
| 3 | non-randomised studies | not serious | not serious | not serious | extremely serious^a^ | none | 131 | 279 | - | MD **2.43 lower** (7.01 lower to 2.15 higher) | ⨁◯◯◯ Very low |  |

a. Number of cohort < 30% Optimum Information Size

b. Heterogeneity > 75% with different directions between studies

1. Creatinine

| **Certainty assessment** | | | | | | | **№ of patients** | | **Effect** | | **Certainty** | **Importance** |
| --- | --- | --- | --- | --- | --- | --- | --- | --- | --- | --- | --- | --- |
| **№ of studies** | **Study design** | **Risk of bias** | **Inconsistency** | **Indirectness** | **Imprecision** | **Other considerations** | **Dead** | **Alive** | **Relative (95% CI)** | **Absolute (95% CI)** |  |  |
| **30-day mortality** | | | | | | | | | | | | |
| 2 | non-randomised studies | not serious | serious^a^ | not serious | not serious | none | 94 | 143 | - | MD **0.46 higher** (0.52 lower to 1.43 higher) | ⨁◯◯◯ Very low |  |
| **3-month mortality** | | | | | | | | | | | | |
| 2 | non-randomised studies | not serious | not serious | not serious | not serious | none | 54 | 147 | - | MD **0.21 higher** (0.08 lower to 0.5 higher) | ⨁⨁◯◯ Low |  |

a. Heterogeneity > 75% but the direction of the effect is the same

1. Copeptin

| **Certainty assessment** | | | | | | | **№ of patients** | | **Effect** | | **Certainty** | **Importance** |
| --- | --- | --- | --- | --- | --- | --- | --- | --- | --- | --- | --- | --- |
| **№ of studies** | **Study design** | **Risk of bias** | **Inconsistency** | **Indirectness** | **Imprecision** | **Other considerations** | **Dead** | **Alive** | **Relative (95% CI)** | **Absolute (95% CI)** |  |  |
| **3-month mortality** | | | | | | | | | | | | |
| 2 | non-randomised studies | not serious | not serious | not serious | not serious | very strong association | 40 | 271 | - | MD **28.45 higher** (26.11 higher to 30.78 higher) | ⨁⨁⨁⨁ High |  |

1. S100β

| **Certainty assessment** | | | | | | | **№ of patients** | | **Effect** | | **Certainty** | **Importance** |
| --- | --- | --- | --- | --- | --- | --- | --- | --- | --- | --- | --- | --- |
| **№ of studies** | **Study design** | **Risk of bias** | **Inconsistency** | **Indirectness** | **Imprecision** | **Other considerations** | **Dead** | **Alive** | **Relative (95% CI)** | **Absolute (95% CI)** |  |  |
| **7-day mortality** | | | | | | | | | | | | |
| 2 | non-randomised studies | not serious | not serious | not serious | not serious | very strong association | 47 | 77 | - | MD **95.43 higher** (72.65 higher to 118.21 higher) | ⨁⨁⨁⨁ High |  |

**Evidence for Functional Outcomes**

**Angiogenic Factors**

1. Ang-1

| **Certainty assessment** | | | | | | | **№ of patients** | | **Effect** | | **Certainty** | **Importance** |
| --- | --- | --- | --- | --- | --- | --- | --- | --- | --- | --- | --- | --- |
| **№ of studies** | **Study design** | **Risk of bias** | **Inconsistency** | **Indirectness** | **Imprecision** | **Other considerations** | **Good Outcome** | **Poor Outcome** | **Relative (95% CI)** | **Absolute (95% CI)** |  |  |
| **3-month Functional Outcome** | | | | | | | | | | | | |
| 2 | non-randomised studies | not serious | not serious | not serious | extremely serious^a^ | none | 89 | 65 | - | MD **2.59 higher** (6.88 lower to 12.05 higher) | ⨁◯◯◯ Very low |  |

a. Number of cohort < 30% Optimum Information Size

1. VEGF

| **Certainty assessment** | | | | | | | **№ of patients** | | **Effect** | | **Certainty** | **Importance** |
| --- | --- | --- | --- | --- | --- | --- | --- | --- | --- | --- | --- | --- |
| **№ of studies** | **Study design** | **Risk of bias** | **Inconsistency** | **Indirectness** | **Imprecision** | **Other considerations** | **Good Outcome** | **Poor Outcome** | **Relative (95% CI)** | **Absolute (95% CI)** |  |  |
| **3-month Functional Outcome** | | | | | | | | | | | | |
| 2 | non-randomised studies | not serious | not serious | not serious | not serious | none | 89 | 65 | - | MD **62.11 higher** (9.24 higher to 114.98 higher) | ⨁⨁◯◯ Low |  |

**Inflammatory Biomarkers**

1. C-Reactive Protein

| **Certainty assessment** | | | | | | | **№ of patients** | | **Effect** | | **Certainty** | **Importance** |
| --- | --- | --- | --- | --- | --- | --- | --- | --- | --- | --- | --- | --- |
| **№ of studies** | **Study design** | **Risk of bias** | **Inconsistency** | **Indirectness** | **Imprecision** | **Other considerations** | **Good Outcome** | **Poor Outcome** | **Relative (95% CI)** | **Absolute (95% CI)** |  |  |
| **30-day functional outcome** | | | | | | | | | | | | |
| 2 | non-randomised studies | not serious | not serious | not serious | not serious | none | 186 | 212 | - | MD **0.84 lower** (1.29 lower to 0.4 lower) | ⨁⨁◯◯ Low |  |
| **3-mo Functional Outcome** | | | | | | | | | | | | |
| 12 | non-randomised studies | not serious | serious^a^ | not serious | not serious | none | 1470 | 1567 | - | MD **3.72 lower** (8.12 lower to 0.67 higher) | ⨁◯◯◯ Very low |  |
| **6-mo Functional Outcome** | | | | | | | | | | | | |
| 6 | non-randomised studies | not serious | not serious | not serious | not serious | none | 325 | 415 | - | MD **2.3 lower** (3.03 lower to 1.57 lower) | ⨁⨁◯◯ Low |  |
| **1-year functional outcome** | | | | | | | | | | | | |
| 2 | non-randomised studies | not serious | not serious | not serious | not serious | none | 806 | 668 | - | MD **7.27 lower** (16.19 lower to 1.66 higher) | ⨁⨁◯◯ Low |  |

a. Heterogeneity > 75% but the direction of the effect is the same

1. IL-6

| **Certainty assessment** | | | | | | | **№ of patients** | | **Effect** | | **Certainty** | **Importance** |
| --- | --- | --- | --- | --- | --- | --- | --- | --- | --- | --- | --- | --- |
| **№ of studies** | **Study design** | **Risk of bias** | **Inconsistency** | **Indirectness** | **Imprecision** | **Other considerations** | **Good Outcome** | **Poor Outcome** | **Relative (95% CI)** | **Absolute (95% CI)** |  |  |
| **3-month functional outcome** | | | | | | | | | | | | |
| 4 | non-randomised studies | not serious | not serious | not serious | not serious | Strong association | 164 | 190 | - | MD **7.69 lower** (9.40 lower to 5.98 lower) | ⨁⨁⨁◯ Moderate |  |

a. Heterogeneity > 75% but the direction of the effect is the same

1. TNF-α

| **Certainty assessment** | | | | | | | **№ of patients** | | **Effect** | | **Certainty** | **Importance** |
| --- | --- | --- | --- | --- | --- | --- | --- | --- | --- | --- | --- | --- |
| **№ of studies** | **Study design** | **Risk of bias** | **Inconsistency** | **Indirectness** | **Imprecision** | **Other considerations** | **Good Outcome** | **Poor Outcome** | **Relative (95% CI)** | **Absolute (95% CI)** |  |  |
| **3-month functional outcome** | | | | | | | | | | | | |
| 3 | non-randomised studies | not serious | not serious | not serious | not serious | strong association | 115 | 118 | - | MD **3.35 lower** (5.17 lower to 1.54 lower) | ⨁⨁⨁◯ Moderate |  |

**Coagulation Parameters**

1. Prothrombin Time

| **Certainty assessment** | | | | | | | **№ of patients** | | **Effect** | | **Certainty** | **Importance** |
| --- | --- | --- | --- | --- | --- | --- | --- | --- | --- | --- | --- | --- |
| **№ of studies** | **Study design** | **Risk of bias** | **Inconsistency** | **Indirectness** | **Imprecision** | **Other considerations** | **Good Outcome** | **Poor Outcome** | **Relative (95% CI)** | **Absolute (95% CI)** |  |  |
| **3-month Functional Outcome** | | | | | | | | | | | | |
| 8 | non-randomised studies | not serious | not serious | not serious | extremely serious^a^ | none | 504 | 938 | - | MD **0.03 lower** (0.20 lower to 0.13 higher) | ⨁◯◯◯ Very low |  |
| **6-mo functional outcome** | | | | | | | | | | | | |
| 4 | non-randomised studies | not serious | not serious | not serious | very serious^b^ | none | 427 | 426 | - | MD **0.21 lower** (0.63 lower to 0.2 higher) | ⨁◯◯◯ Very low |  |

a. Number of cohort < 30% Optimum Information Size

b. Number of cohort < 40% Optimum Information Size

1. Thrombin Time

| **Certainty assessment** | | | | | | | **№ of patients** | | **Effect** | | **Certainty** | **Importance** |
| --- | --- | --- | --- | --- | --- | --- | --- | --- | --- | --- | --- | --- |
| **№ of studies** | **Study design** | **Risk of bias** | **Inconsistency** | **Indirectness** | **Imprecision** | **Other considerations** | **Good Outcome** | **Poor Outcome** | **Relative (95% CI)** | **Absolute (95% CI)** |  |  |
| **Thrombin 3-mo functional outcome** | | | | | | | | | | | | |
| 3 | non-randomised studies | not serious | not serious | not serious | extremely serious^a^ | none | 186 | 276 | - | MD **0.4 lower** (0.87 lower to 0.08 higher) | ⨁◯◯◯ Very low |  |
| **Thrombin 6-mo functional outcome** | | | | | | | | | | | | |
| 3 | non-randomised studies | not serious | not serious | not serious | extremely serious^a^ | none | 150 | 222 | - | MD **0.09 lower** (0.66 lower to 0.48 higher) | ⨁◯◯◯ Very low |  |

a. Number of cohort < 30% Optimum Information Size

1. Activated Partial Thromboplastin Time

| **Certainty assessment** | | | | | | | **№ of patients** | | **Effect** | | **Certainty** | **Importance** |
| --- | --- | --- | --- | --- | --- | --- | --- | --- | --- | --- | --- | --- |
| **№ of studies** | **Study design** | **Risk of bias** | **Inconsistency** | **Indirectness** | **Imprecision** | **Other considerations** | **Good Outcome** | **Poor Outcome** | **Relative (95% CI)** | **Absolute (95% CI)** |  |  |
| **3-month functional outcome** | | | | | | | | | | | | |
| 12 | non-randomised studies | not serious | not serious | not serious | extremely serious^b^ | none | 1282 | 2012 | - | MD **0.03 higher** (0.37 lower to 0.31 higher) | ⨁◯◯◯ Very low |  |
| **6-mo functional outcome** | | | | | | | | | | | | |
| 5 | non-randomised studies | not serious | not serious | not serious | extremely serious^b^ | none | 449 | 479 | - | MD **0.47 lower** (1.62 lower to 0.68 higher) | ⨁◯◯◯ Very low |  |

a. Heterogeneity > 50% with different directions between studies

b. Number of cohort < 30% Optimum Information Size

c. Heterogeneity > 75% with different directions between studies

1. Fibrinogen

| **Certainty assessment** | | | | | | | **№ of patients** | | **Effect** | | **Certainty** | **Importance** |
| --- | --- | --- | --- | --- | --- | --- | --- | --- | --- | --- | --- | --- |
| **№ of studies** | **Study design** | **Risk of bias** | **Inconsistency** | **Indirectness** | **Imprecision** | **Other considerations** | **Good Outcome** | **Poor Outcome** | **Relative (95% CI)** | **Absolute (95% CI)** |  |  |
| **3-month functional outcome** | | | | | | | | | | | | |
| 13 | non-randomised studies | not serious | not serious | not serious | not serious | none | 895 | 1064 | - | MD **0.09 lower** (0.22 lower to 0.04 lower) | ⨁⨁◯◯ Low |  |
| **6-mo functional outcome** | | | | | | | | | | | | |
| 3 | non-randomised studies | not serious | not serious | not serious | extremely serious^b^ | none | 150 | 222 | - | MD **0.04 higher** (0.48 lower to 0.55 higher) | ⨁◯◯◯ Very low |  |

a. Heterogeneity > 75% but the direction of the effect is the same

b. Number of cohort < 30% Optimum Information Size

1. D-Dimer

| **Certainty assessment** | | | | | | | **№ of patients** | | **Effect** | | **Certainty** | **Importance** |
| --- | --- | --- | --- | --- | --- | --- | --- | --- | --- | --- | --- | --- |
| **№ of studies** | **Study design** | **Risk of bias** | **Inconsistency** | **Indirectness** | **Imprecision** | **Other considerations** | **Good Outcome** | **Poor Outcome** | **Relative (95% CI)** | **Absolute (95% CI)** |  |  |
| **3-month functional outcome** | | | | | | | | | | | | |
| 5 | non-randomised studies | not serious | serious^a^ | not serious | not serious | none | 511 | 535 | - | MD **0.54 lower** (0.86 lower to 0.21 lower) | ⨁◯◯◯ Very low |  |
| **6-mo functional outcome** | | | | | | | | | | | | |
| 4 | non-randomised studies | not serious | not serious | not serious | not serious | none | 191 | 273 | - | MD **2.23 lower** (3.02 lower to 1.45 lower) | ⨁⨁◯◯ Low |  |

a. Heterogeneity > 75% but the direction of the effect is the same

**Blood Counts**

1. White Blood Cells

| **Certainty assessment** | | | | | | | **№ of patients** | | **Effect** | | **Certainty** | **Importance** |
| --- | --- | --- | --- | --- | --- | --- | --- | --- | --- | --- | --- | --- |
| **№ of studies** | **Study design** | **Risk of bias** | **Inconsistency** | **Indirectness** | **Imprecision** | **Other considerations** | **Good Outcome** | **Poor Outcome** | **Relative (95% CI)** | **Absolute (95% CI)** |  |  |
| **30-day Functional Outcome** | | | | | | | | | | | | |
| 4 | non-randomised studies | not serious | not serious | not serious | not serious | very strong association | 328 | 385 | - | MD **2.11 lower** (2.35 lower to 1.86 lower) | ⨁⨁⨁⨁ High |  |
| **3-mo functional outcome** | | | | | | | | | | | | |
| 24 | non-randomised studies | not serious | serious^a^ | not serious | not serious | none |  |  | - | MD **1.36 lower** (1.90 lower to 0.83 lower) | ⨁◯◯◯ Very low |  |
| **6-mo Functional Outcome** | | | | | | | | | | | | |
| 7 | non-randomised studies | not serious | not serious | not serious | not serious | none |  |  | - | MD **1.47 lower** (2.46 lower to 0.48 lower) | ⨁⨁◯◯ Low |  |

a. Heterogeneity > 75% but the direction of the effect is the same

1. Neutrophil

| **Certainty assessment** | | | | | | | **№ of patients** | | **Effect** | | **Certainty** | **Importance** |
| --- | --- | --- | --- | --- | --- | --- | --- | --- | --- | --- | --- | --- |
| **№ of studies** | **Study design** | **Risk of bias** | **Inconsistency** | **Indirectness** | **Imprecision** | **Other considerations** | **Good Outcome** | **Poor Outcome** | **Relative (95% CI)** | **Absolute (95% CI)** |  |  |
| **30-day Functional Outcome** | | | | | | | | | | | | |
| 2 | non-randomised studies | not serious | not serious | not serious | not serious | none | 142 | 173 | - | MD **2.58 lower** (3.31 lower to 1.84 lower) | ⨁⨁◯◯ Low |  |
| **3-month Functional Outcome** | | | | | | | | | | | | |
| 5 | non-randomised studies | not serious | very serious^a^ | not serious | not serious | none | 802 | 1038 | - | MD **1.68 lower** (2.55 lower to 0.81 lower) | ⨁◯◯◯ Very low |  |

a. Heterogeneity > 75% with different directions between studies

1. Lymphocyte

| **Certainty assessment** | | | | | | | **№ of patients** | | **Effect** | | **Certainty** | **Importance** |
| --- | --- | --- | --- | --- | --- | --- | --- | --- | --- | --- | --- | --- |
| **№ of studies** | **Study design** | **Risk of bias** | **Inconsistency** | **Indirectness** | **Imprecision** | **Other considerations** | **Good Outcome** | **Poor Outcome** | **Relative (95% CI)** | **Absolute (95% CI)** |  |  |
| **30-day Functional Outcome** | | | | | | | | | | | | |
| 2 | non-randomised studies | not serious | serious^a^ | not serious | not serious | none | 142 | 173 | - | MD **0.44 higher** (0.11 higher to 0.76 higher) | ⨁◯◯◯ Very low |  |
| **3-month Functional Outcome** | | | | | | | | | | | | |
| 5 | non-randomised studies | not serious | serious^a^ | not serious | not serious | none | 744 | 1158 | - | MD **0.32 higher** (0.15 higher to 0.5 higher) | ⨁◯◯◯ Very low |  |

1. Heterogeneity > 75% but the direction of the effect is the same
2. Neutrophil to Lymphocyte Ratio

| **Certainty assessment** | | | | | | | **№ of patients** | | **Effect** | | **Certainty** | **Importance** |
| --- | --- | --- | --- | --- | --- | --- | --- | --- | --- | --- | --- | --- |
| **№ of studies** | **Study design** | **Risk of bias** | **Inconsistency** | **Indirectness** | **Imprecision** | **Other considerations** | **Neutrofil to Lymphocyte Ratio** | **placebo** | **Relative (95% CI)** | **Absolute (95% CI)** |  |  |
| **30-day Functional Outcome** | | | | | | | | | | | | |
| 2 | non-randomised studies | not serious | not serious | not serious | not serious | strong association | 142 | 173 | - | MD **4.33 lower** (5.31 lower to 3.36 lower) | ⨁⨁⨁◯ Moderate |  |
| **3-month Functional Outcome** | | | | | | | | | | | | |
| 8 | non-randomised studies | not serious | serious^a^ | not serious | not serious | none | 958 | 1387 | - | MD **3.08 lower** (4.22 lower to 1.94 lower) | ⨁◯◯◯ Very low^a^ |  |

1. Heterogeneity > 50% but the direction of the effect is the same
2. Monocyte

| **Certainty assessment** | | | | | | | **№ of patients** | | **Effect** | | **Certainty** | **Importance** |
| --- | --- | --- | --- | --- | --- | --- | --- | --- | --- | --- | --- | --- |
| **№ of studies** | **Study design** | **Risk of bias** | **Inconsistency** | **Indirectness** | **Imprecision** | **Other considerations** | **Good Outcome** | **Poor Outcome** | **Relative (95% CI)** | **Absolute (95% CI)** |  |  |
| **3-mo functional outcome** | | | | | | | | | | | | |
| 3 | non-randomised studies | not serious | not serious | not serious | not serious | none | 344 | 620 | - | MD **0.05 lower** (0.09 lower to 0.01 lower) | ⨁⨁◯◯ Low |  |

1. Platelet

| **Certainty assessment** | | | | | | | **№ of patients** | | **Effect** | | **Certainty** | **Importance** |
| --- | --- | --- | --- | --- | --- | --- | --- | --- | --- | --- | --- | --- |
| **№ of studies** | **Study design** | **Risk of bias** | **Inconsistency** | **Indirectness** | **Imprecision** | **Other considerations** | **Good Outcome** | **Poor Outcome** | **Relative (95% CI)** | **Absolute (95% CI)** |  |  |
| **3-month functional outcome** | | | | | | | | | | | | |
| 14 | non-randomised studies | not serious | serious^a^ | not serious | extremely serious^b^ | none | 862 | 1256 | - | MD **1.85 lower** (10.16 lower to 6.47 higher) | ⨁◯◯◯ Very low |  |
| **6-mo functional outcome** | | | | | | | | | | | | |
| 4 | non-randomised studies | not serious | not serious | not serious | very serious^d^ | none | 172 | 275 | - | MD **4.16 higher** (1.84 lower to 10.16 higher) | ⨁◯◯◯ Very low |  |

a. Heterogeneity > 50% with different directions between studies

b. Number of cohort < 30% Optimum Information Size

c. Number of cohort < 40% Optimum Information Size

**Others**

1. Glucose

| **Certainty assessment** | | | | | | | **№ of patients** | | **Effect** | | **Certainty** | **Importance** |
| --- | --- | --- | --- | --- | --- | --- | --- | --- | --- | --- | --- | --- |
| **№ of studies** | **Study design** | **Risk of bias** | **Inconsistency** | **Indirectness** | **Imprecision** | **Other considerations** | **Good Outcome** | **Poor Outcome** | **Relative (95% CI)** | **Absolute (95% CI)** |  |  |
| **30-day functional outcome** | | | | | | | | | | | | |
| 2 | non-randomised studies | not serious | not serious | not serious | not serious | very strong association | 186 | 212 | - | MD **2 lower** (2.36 lower to 1.63 lower) | ⨁⨁⨁⨁ High |  |
| **3-month Functional Outcome** | | | | | | | | | | | | |
| 29 | non-randomised studies | not serious | very serious^a^ | not serious | not serious | none | 2334 | 2403 | - | MD **0.89 lower** (1.26 lower to 0.52 lower) | ⨁◯◯◯ Very low |  |
| **6-mo Functional Outcome** | | | | | | | | | | | | |
| 9 | non-randomised studies | not serious | not serious | not serious | not serious | none | 452 | 587 | - | MD **1.68 lower** (2.19 lower to 1.17 lower) | ⨁⨁◯◯ Low |  |
| **1-year functional outcome** | | | | | | | | | | | | |
| 2 | non-randomised studies | not serious | very serious^a^ | not serious | not serious | none | 645 | 829 | - | MD **1.21 higher** (1.99 lower to 4.41 higher) | ⨁◯◯◯ Very low |  |

a. Heterogeneity > 75% with different directions between studies

1. Low Density Lipoprotein

| **Certainty assessment** | | | | | | | **№ of patients** | | **Effect** | | **Certainty** | **Importance** |
| --- | --- | --- | --- | --- | --- | --- | --- | --- | --- | --- | --- | --- |
| **№ of studies** | **Study design** | **Risk of bias** | **Inconsistency** | **Indirectness** | **Imprecision** | **Other considerations** | **Good Outcome** | **Poor Outcome** | **Relative (95% CI)** | **Absolute (95% CI)** |  |  |
| **3-month functional outcome** | | | | | | | | | | | | |
| 7 | non-randomised studies | not serious | serious^a^ | not serious | extremely serious^b^ | none | 1461 | 1370 | - | MD **0.03**  (0.06 lower to 0.12 higher) | ⨁◯◯◯ Very low |  |

a. Heterogeneity > 50% with different directions between studies

b. Number of cohort < 30% Optimum Information Size

1. High Density Lipoprotein

| **Certainty assessment** | | | | | | | **№ of patients** | | **Effect** | | **Certainty** | **Importance** |
| --- | --- | --- | --- | --- | --- | --- | --- | --- | --- | --- | --- | --- |
| **№ of studies** | **Study design** | **Risk of bias** | **Inconsistency** | **Indirectness** | **Imprecision** | **Other considerations** | **Good Outcome** | **Poor Outcome** | **Relative (95% CI)** | **Absolute (95% CI)** |  |  |
| **3-month functional outcome** | | | | | | | | | | | | |
| 4 | non-randomised studies | not serious | serious^a^ | not serious | extremely serious^b^ | none | 330 | 323 | - | MD **0.02 lower** (0.11 lower to 0.14 higher) | ⨁◯◯◯ Very low |  |

a. Heterogeneity > 50% with different directions between studies

b. Number of cohort < 30% Optimum Information Size

1. Triglycerides

| **Certainty assessment** | | | | | | | **№ of patients** | | **Effect** | | **Certainty** | **Importance** |
| --- | --- | --- | --- | --- | --- | --- | --- | --- | --- | --- | --- | --- |
| **№ of studies** | **Study design** | **Risk of bias** | **Inconsistency** | **Indirectness** | **Imprecision** | **Other considerations** | **Good Outcome** | **Poor Outcome** | **Relative (95% CI)** | **Absolute (95% CI)** |  |  |
| **3-month functional outcome** | | | | | | | | | | | | |
| 6 | non-randomised studies | not serious | serious^a^ | not serious | not serious | none | 1306 | 1268 | - | MD **0.11 higher** (0.04 lower to 0.18 higher) | ⨁◯◯◯ Very low |  |

a. Heterogeneity > 50% with different directions between studies

1. Total Cholesterol

| **Certainty assessment** | | | | | | | **№ of patients** | | **Effect** | | **Certainty** | **Importance** |
| --- | --- | --- | --- | --- | --- | --- | --- | --- | --- | --- | --- | --- |
| **№ of studies** | **Study design** | **Risk of bias** | **Inconsistency** | **Indirectness** | **Imprecision** | **Other considerations** | **Good Outcome** | **Poor Outcome** | **Relative (95% CI)** | **Absolute (95% CI)** |  |  |
| **3-month functional outcome** | | | | | | | | | | | | |
| 5 | non-randomised studies | not serious | serious^a^ | not serious | not serious | none | 1034 | 1046 | - | MD **0.12 higher** (0.09 lower to 0.33 higher) | ⨁◯◯◯ Very low |  |
| **6-mo functional outcome** | | | | | | | | | | | | |
| 2 | non-randomised studies | not serious | not serious | not serious | extremely serious^b^ | none | 74 | 129 | - | MD **0.25 higher** (0.09 lower to 0.59 higher) | ⨁◯◯◯ Very low |  |

a. Heterogeneity > 50% with different directions between studies

b. Number of cohort < 30% Optimum Information Size

1. Calcium

| **Certainty assessment** | | | | | | | **№ of patients** | | **Effect** | | **Certainty** | **Importance** |
| --- | --- | --- | --- | --- | --- | --- | --- | --- | --- | --- | --- | --- |
| **№ of studies** | **Study design** | **Risk of bias** | **Inconsistency** | **Indirectness** | **Imprecision** | **Other considerations** | **Good Outcome** | **Poor Outcome** | **Relative (95% CI)** | **Absolute (95% CI)** |  |  |
| **3-month Functional Outcome** | | | | | | | | | | | | |
| 2 | non-randomised studies | not serious | not serious | not serious | not serious | none | 591 | 782 | - | MD **0.13 higher** (0.09 higher to 0.16 higher) | ⨁⨁◯◯ Low |  |

1. Potassium

| **Certainty assessment** | | | | | | | **№ of patients** | | **Effect** | | **Certainty** | **Importance** |
| --- | --- | --- | --- | --- | --- | --- | --- | --- | --- | --- | --- | --- |
| **№ of studies** | **Study design** | **Risk of bias** | **Inconsistency** | **Indirectness** | **Imprecision** | **Other considerations** | **Good Outcome** | **Poor Outcome** | **Relative (95% CI)** | **Absolute (95% CI)** |  |  |
| **3-month functional outcome** | | | | | | | | | | | | |
| 2 | non-randomised studies | not serious | not serious | not serious | extremely serious^a^ | none | 95 | 102 | - | MD **0.06 higher** (0.08 lower to 0.21 higher) | ⨁◯◯◯ Very low |  |

a. Number of cohort < 30% Optimum Information Size

1. Haemoglobin

| **Certainty assessment** | | | | | | | **№ of patients** | | **Effect** | | **Certainty** | **Importance** |
| --- | --- | --- | --- | --- | --- | --- | --- | --- | --- | --- | --- | --- |
| **№ of studies** | **Study design** | **Risk of bias** | **Inconsistency** | **Indirectness** | **Imprecision** | **Other considerations** | **Good Outcome** | **Poor Outcome** | **Relative (95% CI)** | **Absolute (95% CI)** |  |  |
| **3-mo functional outcome** | | | | | | | | | | | | |
| 8 | non-randomised studies | not serious | not serious | not serious | extremely serious^a^ | none | 457 | 640 | - | MD **1.02 higher** (1.11 lower to 3.14 higher) | ⨁◯◯◯ Very low |  |
| **6-mo functional outcome** | | | | | | | | | | | | |
| 3 | non-randomised studies | not serious | not serious | not serious | extremely serious^a^ | none | 150 | 222 | - | MD **1.07 higher** (3.4 lower to 5.54 higher) | ⨁◯◯◯ Very low |  |

a. Number of cohort < 30% Optimum Information Size

1. Creatinine

| **Certainty assessment** | | | | | | | **№ of patients** | | **Effect** | | **Certainty** | **Importance** |
| --- | --- | --- | --- | --- | --- | --- | --- | --- | --- | --- | --- | --- |
| **№ of studies** | **Study design** | **Risk of bias** | **Inconsistency** | **Indirectness** | **Imprecision** | **Other considerations** | **Good Outcome** | **Poor Outcome** | **Relative (95% CI)** | **Absolute (95% CI)** |  |  |
| **3-month functional outcome** | | | | | | | | | | | | |
| 6 | non-randomised studies | not serious | not serious | not serious | extremely serious^a^ | none | 429 | 404 | - | MD **0**  (0.04 lower to 0.04 higher) | ⨁◯◯◯ Very low |  |

1. Number of cohort < 30% Optimum Information Size
2. ALT

| **Certainty assessment** | | | | | | | **№ of patients** | | **Effect** | | **Certainty** | **Importance** |
| --- | --- | --- | --- | --- | --- | --- | --- | --- | --- | --- | --- | --- |
| **№ of studies** | **Study design** | **Risk of bias** | **Inconsistency** | **Indirectness** | **Imprecision** | **Other considerations** | **AST ALT** | **placebo** | **Relative (95% CI)** | **Absolute (95% CI)** |  |  |
| **3-month functional outcome ALT** | | | | | | | | | | | | |
| 3 | non-randomised studies | not serious | not serious | not serious | extremely serious^a^ | none | 291 | 247 | - | MD **0.08 lower** (2.99 lower to 2.83 higher) | ⨁◯◯◯ Very low^a^ |  |

1. AST

| **Certainty assessment** | | | | | | | **№ of patients** | | **Effect** | | **Certainty** | **Importance** |
| --- | --- | --- | --- | --- | --- | --- | --- | --- | --- | --- | --- | --- |
| **№ of studies** | **Study design** | **Risk of bias** | **Inconsistency** | **Indirectness** | **Imprecision** | **Other considerations** | **AST ALT** | **placebo** | **Relative (95% CI)** | **Absolute (95% CI)** |  |  |
| **3-month Functional Outcome AST** | | | | | | | | | | | | |
| 4 | non-randomised studies | not serious | not serious | not serious | not serious | none | 311 | 297 | - | MD **2.28 lower** (4.9 lower to 0.34 higher) | ⨁⨁◯◯ Low |  |

1. Copeptin

| **Certainty assessment** | | | | | | | **№ of patients** | | **Effect** | | **Certainty** | **Importance** |
| --- | --- | --- | --- | --- | --- | --- | --- | --- | --- | --- | --- | --- |
| **№ of studies** | **Study design** | **Risk of bias** | **Inconsistency** | **Indirectness** | **Imprecision** | **Other considerations** | **Good Outcome** | **Poor Outcome** | **Relative (95% CI)** | **Absolute (95% CI)** |  |  |
| **3-mo functional outcome** | | | | | | | | | | | | |
| 2 | non-randomised studies | not serious | not serious | not serious | not serious | very strong association | 207 | 104 | - | MD **17.96 lower** (19.28 lower to 16.64 lower) | ⨁⨁⨁⨁ High |  |
