# Supplementary material for: Potential blood biomarkers that can be used as prognosticators of spontaneous intracerebral hemorrhage: A systematic review and meta-analysis
Source: PLoS One. 2025 Feb 19;20(2):e0315333. doi: 10.1371/journal.pone.0315333 (PMC11838903; doi:10.1371/journal.pone.0315333)
Supplement: S3 Table — (DOCX) [file pone.0315333.s005.docx]

**Supplemental Table 3.** Effect Size and Optimum Information Size Calculation

**Mortality**

| **DATA ENTRY** |  |  |  |  |  |  |  |  |  |  | **STANDARDISED EFFECT SIZE** | | | | | |  | **IMPRECISION** | | | |  |
| --- | --- | --- | --- | --- | --- | --- | --- | --- | --- | --- | --- | --- | --- | --- | --- | --- | --- | --- | --- | --- | --- | --- |
| Outcome measure | Treatment group | | | Control group | | | Mean Difference | p-value for mean diff (2-tailed T-test) | Confidence Interval for Difference | | Effect Size | Bias corrected (Hedges) | Standard Error of E.S. estimate | Confidence Interval for Effect Size | | Interpretation | OIS | 30% OIS | 40% OIS | 50% OIS | Interpretation |  |
|  | mean | n | SD | mean | n | SD |  |  | lower | upper |  |  |  | lower | upper |  |  |  |  |  |  |  |
| CRP 7-day mortality | 14.08 | 149 | 8.22 | 8.09 | 480 | 4.47 | 5.99 | 0.00 | 4.96 | 7.02 | 1.07 | 1.07 | 0.10 | 0.88 | 1.26 | Large | 13.67 | 4.10 | 5.47 | 6.83 | not serious |  |
| CRP 30-day mortality | 10.56 | 278 | 1.46 | 7.93 | 779 | 2.43 | 2.63 | 0.00 | 2.33 | 2.93 | 1.19 | 1.19 | 0.07 | 1.04 | 1.33 | Large | 11.16 | 3.35 | 4.46 | 5.58 | not serious |  |
| CRP 3-month mortality | 13.73 | 147 | 2.26 | 10.25 | 478 | 2.81 | 3.48 | 0.00 | 2.98 | 3.98 | 1.29 | 1.29 | 0.10 | 1.09 | 1.49 | Large | 9.40 | 2.82 | 3.76 | 4.70 | not serious |  |
| CRP 6-month mortality | 11.83 | 165 | 5.78 | 8.60 | 337 | 3.54 | 3.23 | 0.00 | 2.41 | 4.05 | 0.73 | 0.73 | 0.10 | 0.54 | 0.92 | Medium | 29.16 | 8.75 | 11.67 | 14.58 | not serious |  |
| Prothrombin time 7-day mortality | 12.94 | 127 | 1.40 | 13.10 | 504 | 1.69 | -0.16 | 0.34 | -0.47 | 0.16 | -0.10 | -0.10 | 0.10 | -0.29 | 0.10 | Small | 1718.99 | 515.70 | 687.59 | 859.49 | extremely serious |  |
| Prothrombin time 6-month mortality | 13.15 | 131 | 1.76 | 13.05 | 279 | 1.70 | 0.11 | 0.56 | -0.25 | 0.46 | 0.06 | 0.06 | 0.11 | -0.15 | 0.27 | Small | 4193.11 | 1257.93 | 1677.25 | 2096.56 | extremely serious |  |
| Thrombin 7-day mortality | 17.44 | 67 | 2.49 | 16.94 | 207 | 2.59 | 0.49 | 0.17 | -0.22 | 1.20 | 0.19 | 0.19 | 0.14 | -0.08 | 0.47 | Small | 426.11 | 127.83 | 170.44 | 213.05 | extremely serious |  |
| Thrombin 6-month mortality | 17.47 | 131 | 2.83 | 17.45 | 279 | 2.66 | 0.02 | 0.95 | -0.55 | 0.58 | 0.01 | 0.01 | 0.11 | -0.20 | 0.21 | Small | 419478.78 | 125843.63 | 167791.51 | 209739.39 | extremely serious |  |
| aPTT 7-day mortality | 30.93 | 127 | 3.43 | 30.80 | 504 | 4.60 | 0.13 | 0.77 | -0.73 | 0.99 | 0.03 | 0.03 | 0.10 | -0.17 | 0.22 | Small | 17929.01 | 5378.70 | 7171.60 | 8964.50 | extremely serious |  |
| aPTT 6-month mortality | 33.93 | 273 | 4.20 | 33.59 | 618 | 4.94 | 0.34 | 0.33 | -0.34 | 1.01 | 0.07 | 0.07 | 0.07 | -0.07 | 0.21 | Small | 3093.93 | 928.18 | 1237.57 | 1546.96 | extremely serious |  |
| Fibrinogen 7-day mortality | 3.57 | 132 | 1.15 | 3.25 | 528 | 0.97 | 0.32 | 0.00 | 0.13 | 0.51 | 0.32 | 0.32 | 0.10 | 0.13 | 0.51 | Small | 152.57 | 45.77 | 61.03 | 76.29 | not serious |  |
| Fibrinogen 3-month mortality | 3.32 | 174 | 0.85 | 2.99 | 537 | 1.30 | 0.33 | 0.00 | 0.12 | 0.53 | 0.27 | 0.27 | 0.09 | 0.1 | 0.44 | Small | 212.42 | 63.72 | 84.97 | 106.21 | not serious |  |
| Fibrinogen 6-month mortality | 3.76 | 131 | 2.23 | 3.68 | 279 | 2.35 | 0.08 | 0.75 | -0.4 | 0.56 | 0.03 | 0.03 | 0.11 | -0.17 | 0.24 | Small | 13415.71 | 4024.71 | 5366.29 | 6707.86 | extremely serious |  |
| D-Dimer 7-day mortality | 1.89 | 158 | 0.90 | 1.06 | 504 | 0.92 | 0.84 | 0.00 | 0.67 | 1 | 0.92 | 0.92 | 0.09 | 0.73 | 1.1 | Large | 18.58 | 5.58 | 7.43 | 9.29 | not serious |  |
| D-Dimer 3-month mortality | 2.56 | 88 | 0.67 | 2.17 | 243 | 0.49 | 0.39 | 0.00 | 0.25 | 0.52 | 0.71 | 0.71 | 0.13 | 0.46 | 0.96 | Medium | 30.90 | 9.27 | 12.36 | 15.45 | not serious |  |
| D-Dimer 6-month mortality | 2.70 | 121 | 1.36 | 2.20 | 231 | 1.07 | 0.50 | 0.00 | 0.24 | 0.76 | 0.42 | 0.42 | 0.11 | 0.2 | 0.64 | Small | 87.91 | 26.37 | 35.17 | 43.96 | not serious |  |
| WBC 7-day mortality | 11.55 | 194 | 4.73 | 9.94 | 625 | 3.09 | 1.61 | 0.00 | 1.03 | 2.18 | 0.45 | 0.45 | 0.08 | 0.29 | 0.61 | Small | 76.55 | 22.97 | 30.62 | 38.28 | not serious |  |
| WBC 30-day mortality | 10.01 | 260 | 1.65 | 7.52 | 494 | 1.85 | 2.49 | 0.00 | 2.22 | 2.76 | 1.40 | 1.40 | 0.08 | 1.23 | 1.56 | Large | 8.02 | 2.41 | 3.21 | 4.01 | not serious |  |
| WBC 3-month mortality | 9.88 | 401 | 3.70 | 8.75 | 1227 | 2.58 | 1.13 | 0.00 | 0.81 | 1.46 | 0.39 | 0.39 | 0.06 | 0.28 | 0.5 | Small | 102.66 | 30.80 | 41.06 | 51.33 | not serious |  |
| WBC 6-month mortality | 10.49 | 289 | 4.68 | 8.92 | 684 | 4.51 | 1.57 | 0.00 | 0.94 | 2.2 | 0.34 | 0.34 | 0.07 | 0.21 | 0.48 | Small | 132.69 | 39.81 | 53.08 | 66.35 | not serious |  |
| Neutrophil 3-month mortality | 9.29 | 158 | 3.43 | 7.23 | 472 | 2.73 | 2.06 | 0.00 | 1.53 | 2.58 | 0.7 | 0.70 | 0.09 | 0.52 | 0.89 | Medium | 31.64 | 9.49 | 12.66 | 15.82 | not serious |  |
| Lymphocyte 30-day mortality | 1.20 | 43 | 0.37 | 1.28 | 135 | 0.69 | -0.08 | 0.49 | -0.29 | 0.14 | -0.12 | -0.12 | 0.18 | -0.46 | 0.22 | Small | 1063.42 | 319.03 | 425.37 | 531.71 | extremely serious |  |
| Lymphocyte 3-month mortality | 0.92 | 107 | 0.24 | 1.11 | 272 | 0.27 | -0.2 | 0.00 | -0.26 | -0.14 | -0.74 | -0.74 | 0.12 | -0.97 | -0.51 | Medium | 28.46 | 8.54 | 11.38 | 14.23 | not serious |  |
| Monocyte 3-month mortality | 0.39 | 107 | 0.09 | 0.35 | 272 | 0.06 | 0.04 | 0.00 | 0.03 | 0.06 | 0.6 | 0.59 | 0.12 | 0.37 | 0.82 | Medium | 44.30 | 13.29 | 17.72 | 22.15 | not serious |  |
| Platelet 7-day mortality | 175.24 | 200 | 32.04 | 166.28 | 671 | 33.87 | 8.97 | 0.00 | 3.68 | 14.3 | 0.27 | 0.27 | 0.08 | 0.11 | 0.43 | Small | 218.61 | 65.58 | 87.44 | 109.30 | not serious |  |
| Platelet 30-day mortality | 208.18 | 91 | 71.08 | 207.70 | 189 | 70.69 | 0.48 | 0.96 | -17.3 | 18.3 | 0.01 | 0.01 | 0.13 | -0.24 | 0.26 | Small | 339147.48 | 101744.25 | 135658.99 | 169573.74 | extremely serious |  |
| Platelet 3-month mortality | 206.14 | 290 | 60.38 | 192.72 | 871 | 50.01 | 13.4 | 0.00 | 6.4 | 20.4 | 0.25 | 0.25 | 0.07 | 0.12 | 0.39 | Small | 242.70 | 72.81 | 97.08 | 121.35 | not serious |  |
| Platelet 6-month mortality | 165.67 | 131 | 50.38 | 167.22 | 279 | 46.48 | -1.55 | 0.76 | -11.5 | 8.39 | -0.03 | -0.03 | 0.11 | -0.24 | 0.18 | Small | 14873.45 | 4462.03 | 5949.38 | 7436.72 | extremely serious |  |
| Glucose 7-day mortality | 10.47 | 231 | 3.20 | 8.48 | 822 | 2.43 | 1.99 | 0.00 | 1.61 | 2.37 | 0.76 | 0.76 | 0.08 | 0.61 | 0.91 | Medium | 27.17 | 8.15 | 10.87 | 13.59 | not serious |  |
| Glucose 30-day mortality | 10.57 | 314 | 2.41 | 7.47 | 903 | 2.50 | 3.1 | 0.00 | 2.78 | 3.42 | 1.25 | 1.25 | 0.07 | 1.11 | 1.39 | Large | 10.01 | 3.00 | 4.00 | 5.01 | not serious |  |
| Glucose 3-month mortality | 10.95 | 674 | 3.15 | 9.11 | 1726 | 2.78 | 1.84 | 0.00 | 1.58 | 2.1 | 0.64 | 0.64 | 0.05 | 0.54 | 0.73 | Medium | 38.87 | 11.66 | 15.55 | 19.44 | not serious |  |
| Glucose 6-month mortality | 13.73 | 165 | 5.70 | 11.16 | 337 | 3.38 | 2.57 | 0.00 | 1.77 | 3.37 | 0.6 | 0.60 | 0.10 | 0.41 | 0.79 | Medium | 43.71 | 13.11 | 17.48 | 21.86 | not serious |  |
| Sodium 7-day mortality | 139.66 | 47 | 5.41 | 139.25 | 77 | 3.69 | 0.41 | 0.61 | -1.2 | 2.03 | 0.09 | 0.09 | 0.19 | -0.27 | 0.46 | Small | 1784.41 | 535.32 | 713.76 | 892.20 | extremely serious |  |
| Sodium 30-day mortality | 140.13 | 83 | 4.07 | 137.82 | 154 | 4.88 | 2.3 | 0.00 | 1.07 | 3.54 | 0.5 | 0.50 | 0.14 | 0.23 | 0.77 | Small | 62.98 | 18.90 | 25.19 | 31.49 | not serious |  |
| Sodium 3-month mortality | 138.82 | 146 | 3.66 | 137.90 | 482 | 4.46 | 0.92 | 0.02 | 0.13 | 1.72 | 0.22 | 0.21 | 0.09 | 0.03 | 0.4 | Small | 338.94 | 101.68 | 135.58 | 169.47 | serious |  |
| Potassium 7-day mortality | 4.00 | 47 | 0.62 | 3.80 | 77 | 0.42 | 0.2 | 0.04 | 0.01 | 0.39 | 0.39 | 0.39 | 0.19 | 0.02 | 0.76 | Small | 101.80 | 30.54 | 40.72 | 50.90 | serious |  |
| Hemoglobin 7-day mortality | 130.18 | 101 | 25.00 | 133.25 | 306 | 23.02 | -3.07 | 0.26 | -8.37 | 2.24 | -0.13 | -0.13 | 0.11 | -0.36 | 0.09 | Small | 923.21 | 276.96 | 369.28 | 461.60 | extremely serious |  |
| Hemoglobin 3-month mortality | 124.60 | 116 | 14.19 | 124.54 | 323 | 14.67 | 0.06 | 0.97 | -3.03 | 3.16 | 0 | 0.00 | 0.11 | -0.21 | 0.22 | Small | 852985.11 | 255895.53 | 341194.04 | 426492.55 | extremely serious |  |
| Hemoglobin 6-month mortality | 125.14 | 131 | 22.50 | 127.56 | 279 | 20.83 | -2.43 | 0.28 | -6.88 | 2.02 | -0.11 | -0.11 | 0.11 | -0.32 | 0.09 | Small | 1217.13 | 365.14 | 486.85 | 608.57 | extremely serious |  |
| Creatinine 30-day mortality | 1.35 | 94 | 0.76 | 0.89 | 143 | 0.28 | 0.46 | 0.00 | 0.32 | 0.59 | 0.87 | 0.87 | 0.14 | 0.6 | 1.14 | Large | 20.75 | 6.22 | 8.30 | 10.37 | not serious |  |
| Creatinine 3-month mortality | 1.03 | 54 | 0.55 | 0.82 | 147 | 0.30 | 0.21 | 0.00 | 0.09 | 0.33 | 0.55 | 0.55 | 0.16 | 0.23 | 0.87 | Medium | 51.24 | 15.37 | 20.50 | 25.62 | not serious |  |
| S100beta 7-day mortality | 247.57 | 47 | 66.64 | 152.14 | 77 | 55.75 | 95.4 | 0.00 | 73.4 | 117 | 1.59 | 1.58 | 0.21 | 1.17 | 1.99 | Very Large | 6.22 | 1.87 | 2.49 | 3.11 | not serious |  |
| Copeptine 3-month mortality | 41.16 | 40 | 5.62 | 12.72 | 271 | 10.74 | 28.4 | 0.00 | 25 | 31.9 | 2.78 | 2.77 | 0.20 | 2.37 | 3.17 | Very Large | 2.03 | 0.61 | 0.81 | 1.02 | not serious |  |

**Functional Outcome**

| **DATA ENTRY** |  |  |  |  |  |  |  |  |  |  | **STANDARDISED EFFECT SIZE** | | | | | |  | **IMPRECISION** | | | | |
| --- | --- | --- | --- | --- | --- | --- | --- | --- | --- | --- | --- | --- | --- | --- | --- | --- | --- | --- | --- | --- | --- | --- |
| Outcome measure | Treatment group | | | Control group | | | Mean Difference | p-value for mean diff (2-tailed T-test) | Confidence Interval for Difference | | Effect Size | Bias corrected (Hedges) | Standard Error of E.S. estimate | Confidence Interval for Effect Size | | Interpretation | OIS | 30% OIS | 40% OIS | 50% OIS | Interpretation |  |
|  | mean | n | SD | mean | n | SD |  |  | lower | upper |  |  |  | lower | upper |  |  |  |  |  |  |  |
| Ang-1 3-mo functional | 25.39 | 89 | 13.79 | 22.81 | 65 | 12.86 | 2.59 | 0.24 | -1.73 | 6.91 | 0.19 | **0.19** | 0.16 | -0.13 | 0.51 | Small | 421.72 | 126.52 | 168.69 | 210.86 | extremely serious |  |
| VEGF 3-mo functional | 295.96 | 89 | 154.92 | 233.84 | 65 | 148.52 | 62.11 | 0.01 | 13.03 | 111.19 | 0.41 | **0.41** | 0.16 | 0.08 | 0.73 | Small | 94.32 | 28.30 | 37.73 | 47.16 | not serious |  |
| CRP 30-day functional | 8.02 | 186 | 2.34 | 8.86 | 212 | 1.99 | -0.84 | 0.00 | -1.27 | -0.42 | -0.39 | **-0.39** | 0.10 | -0.59 | -0.19 | Small | 103.30 | 30.99 | 41.32 | 51.65 | not serious |  |
| CRP 3-month functional | 8.51 | 1470 | 4.36 | 12.23 | 1567 | 8.21 | -3.72 | 0.00 | -4.19 | -3.25 | -0.56 | **-0.56** | 0.04 | -0.63 | -0.49 | Medium | 49.89 | 14.97 | 19.96 | 24.95 | not serious |  |
| CRP 6-month functional | 8.28 | 325 | 3.43 | 10.58 | 415 | 5.50 | -2.30 | 0.00 | -2.98 | -1.61 | -0.49 | **-0.49** | 0.08 | -0.63 | -0.34 | Small | 65.87 | 19.76 | 26.35 | 32.93 | not serious |  |
| CRP 1-year functional | 6.61 | 806 | 11.31 | 13.87 | 668 | 16.30 | -7.26 | 0.00 | -8.68 | -5.85 | -0.53 | **-0.53** | 0.05 | -0.63 | -0.42 | Medium | 56.60 | 16.98 | 22.64 | 28.30 | not serious |  |
| IL-6 3-month functional | 10.41 | 164 | 5.55 | 18.10 | 190 | 6.42 | -7.69 | 0.00 | -8.96 | -6.43 | -1.27 | **-1.27** | 0.12 | -1.50 | -1.04 | Large | 9.66 | 2.90 | 3.87 | 4.83 | not serious |  |
| TNF 3-month functional | 8.93 | 115 | 3.92 | 12.29 | 118 | 4.18 | -3.35 | 0.00 | -4.40 | -2.31 | -0.83 | **-0.82** | 0.14 | -1.09 | -0.56 | Large | 22.96 | 6.89 | 9.18 | 11.48 | not serious |  |
| Prothrombin time 3-month functional | 12.12 | 504 | 0.95 | 12.16 | 938 | 0.90 | -0.03 | 0.49 | -0.13 | 0.06 | -0.04 | **-0.04** | 0.06 | -0.15 | 0.07 | Small | 10937.79 | 3281.34 | 4375.11 | 5468.89 | extremely serious |  |
| Prothrombin time 6-month functional | 12.33 | 427 | 1.88 | 12.54 | 426 | 1.77 | -0.21 | 0.09 | -0.46 | 0.03 | -0.12 | **-0.12** | 0.07 | -0.25 | 0.02 | Small | 1174.66 | 352.40 | 469.86 | 587.33 | very serious |  |
| Thrombin time 3-month functional | 16.17 | 186 | 2.42 | 16.57 | 276 | 2.76 | -0.39 | 0.11 | -0.88 | 0.09 | -0.15 | -0.15 | 0.09 | -0.34 | 0.04 | Small | 694.64 | 208.39 | 277.86 | 347.32 | extremely serious |  |
| Thrombin time 6-month functional | 17.41 | 150 | 2.66 | 17.50 | 222 | 2.86 | -0.09 | 0.76 | -0.67 | 0.49 | -0.03 | -0.03 | 0.11 | -0.24 | 0.17 | Small | 14722.25 | 4416.67 | 5888.90 | 7361.12 | extremely serious |  |
| aPTT 3-month functional | 27.54 | 1282 | 3.12 | 27.57 | 2012 | 3.30 | -0.03 | 0.78 | -0.26 | 0.19 | -0.01 | -0.01 | 0.04 | -0.08 | 0.06 | Small | 152538.42 | 45761.53 | 61015.37 | 76269.21 | extremely serious |  |
| aPTT 6-month functional | 32.51 | 449 | 4.94 | 32.97 | 479 | 4.76 | -0.47 | 0.14 | -1.09 | 0.16 | -0.10 | -0.10 | 0.07 | -0.23 | 0.03 | Small | 1678.48 | 503.55 | 671.39 | 839.24 | extremely serious |  |
| Fibrinogen 3-month functional | 3.18 | 895 | 0.82 | 3.28 | 1064 | 0.97 | -0.09 | 0.02 | -0.17 | -0.01 | -0.10 | -0.10 | 0.05 | -0.19 | -0.01 | Small | 1510.23 | 453.07 | 604.09 | 755.12 | not serious |  |
| Fibrinogen 6-month functional | 3.84 | 150 | 2.39 | 3.80 | 222 | 2.23 | 0.04 | 0.88 | -0.44 | 0.51 | 0.02 | 0.02 | 0.11 | -0.19 | 0.22 | Small | 59061.74 | 17718.52 | 23624.70 | 29530.87 | extremely serious |  |
| D-Dimer 3-month functional | 1.36 | 511 | 0.87 | 1.90 | 535 | 1.53 | -0.53 | 0.00 | -0.69 | -0.38 | -0.43 | -0.43 | 0.06 | -0.55 | -0.30 | Small | 86.44 | 25.93 | 34.57 | 43.22 | not serious |  |
| D-Dimer 6-month functional | 7.13 | 191 | 2.85 | 9.37 | 273 | 5.46 | -2.23 | 0.00 | -3.08 | -1.39 | -0.49 | -0.49 | 0.10 | -0.68 | -0.30 | Small | 65.72 | 19.72 | 26.29 | 32.86 | not serious |  |
| WBC 30-day functional | 7.01 | 328 | 1.01 | 9.12 | 385 | 1.69 | -2.11 | 0.00 | -2.32 | -1.90 | -1.49 | -1.48 | 0.08 | -1.65 | -1.32 | Very Large | 7.11 | 2.13 | 2.85 | 3.56 | not serious |  |
| WBC 3-month functional | 8.38 | 1941 | 3.27 | 9.74 | 2353 | 4.04 | -1.36 | 0.00 | -1.58 | -1.14 | -0.37 | -0.37 | 0.03 | -0.43 | -0.31 | Small | 117.19 | 35.16 | 46.88 | 58.60 | not serious |  |
| WBC 6-month functional | 8.24 | 548 | 4.95 | 9.72 | 606 | 5.36 | -1.47 | 0.00 | -2.07 | -0.87 | -0.28 | -0.28 | 0.06 | -0.40 | -0.17 | Small | 193.56 | 58.07 | 77.42 | 96.78 | not serious |  |
| Neutrophil 30-day functional | 5.87 | 142 | 2.51 | 8.45 | 173 | 3.82 | -2.58 | 0.00 | -3.31 | -1.84 | -0.78 | -0.78 | 0.12 | -1.01 | -0.55 | Medium | 25.66 | 7.70 | 10.26 | 12.83 | not serious |  |
| Neutrophil 3-month functional | 6.14 | 802 | 2.11 | 7.82 | 1038 | 2.65 | -1.68 | 0.00 | -1.91 | -1.46 | -0.69 | -0.69 | 0.05 | -0.79 | -0.60 | Medium | 32.89 | 9.87 | 13.16 | 16.45 | not serious |  |
| Lymphocyte 30-day functional | 1.67 | 142 | 0.59 | 1.23 | 173 | 0.61 | 0.44 | 0.00 | 0.30 | 0.57 | 0.73 | 0.73 | 0.12 | 0.50 | 0.96 | Medium | 29.20 | 8.76 | 11.68 | 14.60 | not serious |  |
| Lymphocyte 90-day functional | 1.47 | 744 | 0.90 | 1.15 | 1158 | 0.78 | 0.32 | 0.00 | 0.24 | 0.40 | 0.39 | 0.39 | 0.05 | 0.29 | 0.48 | Small | 105.10 | 31.53 | 42.04 | 52.55 | not serious |  |
| Monocyte 3-month functional | 0.38 | 344 | 0.21 | 0.43 | 620 | 0.31 | -0.05 | 0.01 | -0.09 | -0.02 | -0.19 | -0.19 | 0.07 | -0.32 | -0.05 | Small | 453.70 | 136.11 | 181.48 | 226.85 | not serious |  |
| Platelet 3-month functional | 177.49 | 862 | 49.61 | 179.32 | 1256 | 55.98 | -1.83 | 0.44 | -6.47 | 2.81 | -0.03 | -0.03 | 0.04 | -0.12 | 0.05 | Small | 13436.01 | 4030.80 | 5374.41 | 6718.01 | extremely serious |  |
| Platelet 6-month functional | 73.30 | 172 | 28.18 | 69.14 | 275 | 21.53 | 4.16 | 0.08 | -0.48 | 8.80 | 0.17 | 0.17 | 0.10 | -0.02 | 0.36 | Small | 536.12 | 160.84 | 214.45 | 268.06 | very serious |  |
| Glucose 30-day functional | 6.68 | 186 | 1.15 | 8.68 | 212 | 1.61 | -2.00 | 0.00 | -2.28 | -1.72 | -1.41 | -1.41 | 0.11 | -1.63 | -1.19 | Very Large | 7.85 | 2.36 | 3.14 | 3.93 | not serious |  |
| Glucose 3-month functional | 7.40 | 2334 | 2.22 | 8.30 | 2403 | 2.86 | -0.89 | 0.00 | -1.04 | -0.75 | -0.35 | -0.35 | 0.03 | -0.41 | -0.29 | Small | 128.81 | 38.64 | 51.52 | 64.40 | not serious |  |
| Glucose 6-month functionl | 9.61 | 452 | 3.17 | 11.29 | 587 | 4.72 | -1.68 | 0.00 | -2.19 | -1.17 | -0.41 | -0.41 | 0.06 | -0.53 | -0.28 | Small | 94.27 | 28.28 | 37.71 | 47.13 | not serious |  |
| Glucose 1-year functional | 9.81 | 645 | 3.77 | 8.60 | 829 | 2.76 | 1.21 | 0.00 | 0.88 | 1.54 | 0.37 | 0.37 | 0.05 | 0.27 | 0.48 | Small | 112.88 | 33.86 | 45.15 | 56.44 | not serious |  |
| LDL 3-month functional | 2.75 | 1461 | 0.54 | 2.72 | 1370 | 0.60 | 0.03 | 0.16 | -0.01 | 0.07 | 0.05 | 0.05 | 0.04 | -0.02 | 0.13 | Small | 5548.13 | 1664.44 | 2219.25 | 2774.06 | extremely serious |  |
| HDL 3-month functional | 1.25 | 330 | 0.39 | 1.23 | 323 | 0.48 | 0.02 | 0.62 | -0.05 | 0.08 | 0.04 | 0.04 | 0.08 | -0.11 | 0.19 | Small | 10189.11 | 3056.73 | 4075.64 | 5094.55 | extremely serious |  |
| Triglyceride 3-month functional | 1.91 | 1306 | 0.60 | 1.80 | 1268 | 0.57 | 0.11 | 0.00 | 0.07 | 0.16 | 0.19 | 0.19 | 0.04 | 0.11 | 0.27 | Small | 444.09 | 133.23 | 177.64 | 222.05 | not serious |  |
| Cholesterol 3-month functional | 4.79 | 1322 | 1.00 | 4.69 | 1383 | 0.96 | 0.10 | 0.01 | 0.02 | 0.17 | 0.10 | 0.10 | 0.04 | 0.02 | 0.17 | Small | 1584.01 | 475.20 | 633.61 | 792.01 | not serious |  |
| Cholesterol 6-month functional | 5.57 | 74 | 1.12 | 5.32 | 129 | 1.27 | 0.25 | 0.16 | -0.10 | 0.60 | 0.21 | 0.21 | 0.15 | -0.08 | 0.49 | Small | 366.79 | 110.04 | 146.72 | 183.40 | extremely serious |  |
| Calcium 3-month functional | 2.43 | 591 | 0.22 | 2.31 | 782 | 0.21 | 0.13 | 0.00 | 0.10 | 0.15 | 0.60 | 0.60 | 0.06 | 0.49 | 0.71 | Medium | 42.99 | 12.90 | 17.20 | 21.49 | not serious |  |
| Potasium 3-month functional | 3.77 | 95 | 0.34 | 3.71 | 102 | 0.52 | 0.06 | 0.31 | -0.06 | 0.19 | 0.14 | 0.14 | 0.14 | -0.14 | 0.42 | Small | 752.98 | 225.89 | 301.19 | 376.49 | extremely serious |  |
| Hemoglobin 3-month functional | 129.10 | 457 | 17.95 | 128.09 | 640 | 14.68 | 1.01 | 0.30 | -0.92 | 2.95 | 0.06 | 0.06 | 0.06 | -0.06 | 0.18 | Small | 3970.72 | 1191.21 | 1588.29 | 1985.36 | extremely serious |  |
| Hemoglobin 6-month functional | 126.15 | 150 | 21.09 | 125.08 | 222 | 22.26 | 1.07 | 0.64 | -3.46 | 5.60 | 0.05 | 0.05 | 0.11 | -0.16 | 0.26 | Small | 6508.49 | 1952.55 | 2603.40 | 3254.24 | extremely serious |  |
| Creatinine 3-month functional | 0.67 | 429 | 0.24 | 0.67 | 404 | 0.25 | 0.00 | 0.94 | -0.03 | 0.04 | 0.01 | 0.01 | 0.07 | -0.13 | 0.14 | Small | 518186.80 | 155456.04 | 207274.72 | 259093.40 | extremely serious |  |
| Copeptine 3-month functional | 11.22 | 207 | 4.25 | 29.18 | 104 | 5.85 | -17.97 | 0.00 | -19.11 | -16.82 | -3.71 | -3.70 | 0.19 | -4.08 | -3.33 | Very Large | 1.14 | 0.34 | 0.46 | 0.57 | not serious |  |
| NLR 30-day functional | 3.16 | 142 | 2.16 | 7.50 | 173 | 5.60 | -4.33 | 0.00 | -5.31 | -3.35 | -0.98 | -0.98 | 0.12 | -1.22 | -0.75 | Large | 16.19 | 4.86 | 6.48 | 8.09 | not serious |  |
| NLR 3-month functional | 4.92 | 958 | 5.11 | 8.00 | 1387 | 8.37 | -3.08 | 0.00 | -3.67 | -2.48 | -0.43 | -0.43 | 0.04 | -0.51 | -0.34 | Small | 86.30 | 25.89 | 34.52 | 43.15 | not serious |  |
| AST 3-month functional | 21.58 | 311 | 11.10 | 23.86 | 297 | 13.95 | -2.28 | 0.03 | -4.28 | -0.28 | -0.18 | -0.18 | 0.08 | -0.34 | -0.02 | Small | 477.79 | 143.34 | 191.12 | 238.90 | not serious |  |
| ALT 3-month functional | 22.09 | 291 | 18.68 | 22.16 | 247 | 14.15 | -0.08 | 0.96 | -2.93 | 2.77 | 0.00 | 0.00 | 0.09 | -0.17 | 0.16 | Small | 718693.78 | 215608.13 | 287477.51 | 359346.89 | extremely serious |  |
